# Supplementary material for: Identification and initial response to children’s exposure to intimate partner violence: a qualitative synthesis of the perspectives of children, mothers and professionals
Source: BMJ Open. 2018 Apr 28;8(4):e019761. doi: 10.1136/bmjopen-2017-019761 (PMC5931305; doi:10.1136/bmjopen-2017-019761)
Supplement: Supplementary data [file bmjopen-2017-019761supp001.pdf]

Supplementary file 1

Search strategy

Full Boolean search strategy for MEDLINE

Date range of search 1 April 2013 to 28 April 2016.

Date of search 28 April 2016.

----

1 Child Welfare/ or Child, Preschool/ or Mother-Child Relations/ or Father-Child Relations/ or Child Behavior/ or "Child of Impaired Parents"/ or Child/ or Parent-Child Relations/ or Child Psychology/ or Child Reactive Disorders/ or Child Psychiatry/ or Adolescent Psychiatry/ or Adolescent Behavior/ or Adolescent/ or Adolescent Health Services/ or Adolescent Psychology/ or Adolescent Development/ (2612601)

2 (adolesc* or preadolesc* or pre-adolesc* or boy* or girl* or child* or infant* or preschool* or juvenil* or minors or school* or pediatri* or paediatri* or pubescen* or pre-pubescen* or puberty or student* or teen* or young or youth* or school* or high-school or "high school" or college or undergrad* or campus* or classroom*).mp. [mp=title, abstract, original title, name of substance word, subject heading word, keyword heading word, protocol supplementary concept word, rare disease supplementary concept word, unique identifier] (4168331)

3 1 or 2 (4168331)

4 Domestic Violence/ or Spouse Abuse/ or Battered Women/ (12097)

5 (abuse* adj3 (wom*n or partner or spous* or m*n or wife or wives or husband*)).tw. (5458)

6 (battered adj3 (wom*n or partner or spous* or m*n or wife or wives or husband*)).tw. (695)

7 (violen* adj3 (wom*n or partner or spous* or m*n or wife or wives or husband*)).tw. (7703)

8 (marital adj3 (violen* or abuse*)).tw. (297)

9 (famil* adj3 (violen* or abuse*)).tw. (2930)

10 domestic violen*.tw. (4517)

11 (intimate adj3 partner adj3 (violen* or abuse*)).tw. (4273)

12 (interparental adj3 (violen* or abuse*)).tw. (97)

13 (violen* adj2 (home*1 or household*)).tw. (206)

14 (parent* adj3 (violen* or abuse*)).tw. (1868)

15 or/4-14 (22293)

16 (expose* or exposure).mp. (927096)

17 witnes*.mp. (18418)

18 growing up.tw. (1791)

19 ((child* or adolesc*) adj3 "living with").tw. (1380)

20 16 or 17 or 18 or 19 (946981)

21 3 and 15 and 20 (2401)

22 limit 1 to ed=20130401-20160428 (284370)

23 21 and 22 (426)
