# Supplementary material for: Identification and initial response to children’s exposure to intimate partner violence: a qualitative synthesis of the perspectives of children, mothers and professionals
Source: BMJ Open. 2018 Apr 28;8(4):e019761. doi: 10.1136/bmjopen-2017-019761 (PMC5931305; doi:10.1136/bmjopen-2017-019761)
Supplement: Supplementary data [file bmjopen-2017-019761supp003.pdf]

Supplementary file 3

Quality appraisal for the included studies (n = 11)

Quality appraisal criteria	Yes	Studies
A. Appropriateness of research methodology and design		
1. Does the research seek to interpret or illuminate the actions and/or subjective experiences of research participants?	11	[33 35 37 40-47]
2. Is qualitative research the right methodology for addressing the research goal?	11	[33 35 37 40-47]
3. Is the research design appropriate to address the aims of the research?	11	[33 35 37 40-47]
4. Has the researcher justified the research design	5	[33 42 43 45 46]
B. Ethical considerations		
5. Did the researcher use two or more of the following strategies to ensure ethical issues have been taken into consideration (is there are sufficient details of):<br>a. how the research was explained to participants for the reader to assess whether ethical standards were maintained;<br>b. did the researcher discuss issues raised by the study, such as issues around informed consent or confidentiality or how they have handled the effects of the study on the participants during and after the study;<br>c. was approval sought from an ethics committee)?	8	[33 35 37 41-45]
C. Credibility (akin to internal validity)<br>Do participants and those with similar experiences recognize the experiences contained with the study?		
6. Did the research use one or more of the following strategies to establish credibility:<br>a. has the researcher discussed saturation of data;<br>b. attempt to triangulate data by using different data collection methods;<br>c. member checking to see if participants agreed with the interpretations of the researcher;<br>d. peers or consultants experienced in qualitative research review their coding process;<br>e. full descriptions of member's words in their final paper?	11	[33 35 37 40-47]
D. Transferability (akin to external validity)		

Quality appraisal criteria	Yes	Studies
How does one determine the extent to which the findings of the study are applicability in other contexts or with other participant types?		
7. Did the researchers use any of the following strategies to establish transferability (use of dense description of the population studied through descriptions of demographics and geographic boundaries of the study? Note: the author must describe at least two specific sample descriptors (e.g. age range, gender, setting from which sample was selected, SES, etc.)	8	[33 35 37 42-44 46 47]
E. Consistency (akin to reliability)<br>Can another researcher follow the decision trail used by the researcher?		
8. Did the researcher use any of the following strategies to establish the purpose of the research:<br>a. was there a clear statement of aims of the research;<br>b. what was the goal of the research;<br>c. why was it thought important<br>d. its relevance?	11	[33 35 37 40-47]
9. Was the recruitment strategy appropriate to the aims of the research? (e.g. does the population from which the sample was selected resonate with the research objectives, was the sample selection ethically implemented).	10	[33 35 37 40-42 44-47]
10. Did the researcher explain how participants were selected?	9	[32 37 40 42-47]
11. Did they explain why the participants they selected were the most appropriate to provide access to the type of knowledge sought by the study	4	[32 37 42 45]
12. Were there any discussions around recruitment (e.g. why some people chose not to take part)?	4	[41 44-46]
13. Was the setting for data collection justified?	8	[35 37 40 42-46]
14. Is it clear how data were collected (e.g. focus group, semi-structured interview etc.)?	11	[33 35 37 40-47]
15. Did the researcher justify the methods chosen?	3	[33 44 45]
16. Did the researcher make the methods explicit (e.g. for interview method, is there an indication of how interviews were conducted, or did they use a topic guide)?	10	[33 35 37 41-47]
17. If the form of data is clear (e.g. tape recordings, video material, notes etc.)?	11	[33 35 37 40-47]
18. Did the researcher explain how the data were reduced or transformed for analysis?	11	[33 35 37 40-47]

**Quality appraisal criteria**	**Yes**	**Studies**
19. Did they discuss their interpretation and presentation of their findings?	11	[33 35 37 40-47]
F. Neutrality (akin to objectivity)<br>Did overall credibility, transferability, and consistency occur?		
20. Did the researcher use one or more of the following strategies to ensure neutrality (has the relationship between researcher and participants been adequately considered; has the researcher critically examined their own role, potential bias and influence during the formulation of the research questions or data collection, including sample recruitment and choice of location; did the researcher discuss how they responded to events during the study and whether they considered the implications of any changes in the research design; did the researcher employ field notes to record their personal reactions and biases after each interview/focus group; did they make a conscious effort to follow rather than lead the direction of interviews/focus groups)?	2	[41 45]

Note. Quality was appraised using the modified version of the Critical Appraisal Skill Programme (M-CASP), score range 0-20.[17]
