# Supplementary material for: Identification and initial response to children’s exposure to intimate partner violence: a qualitative synthesis of the perspectives of children, mothers and professionals
Source: BMJ Open. 2018 Apr 28;8(4):e019761. doi: 10.1136/bmjopen-2017-019761 (PMC5931305; doi:10.1136/bmjopen-2017-019761)
Supplement: Supplementary data [file bmjopen-2017-019761supp004.pdf]

Supplementary file 4

Descriptive themes

Descriptive theme with subthemes		Stakeholder group, study		
		Children	Parents	Professionals
**1. Experiences of identification of children's exposure to IPV**				
1.1. Approach	1.1.1. How to ask	All children felt that HCPs should initiate a conversation about safety and well-being by giving permission, space and time to discuss sensitive matters.[45]	All mothers felt that HCPs should initiate a conversation about safety and well-being by giving permission, space and time to discuss sensitive matters.[45]	
	1.1.2. When to ask	Most children found it acceptable when HCP initiates enquiry about 'safety at home' from the presenting problem.[45]		Most HCPs described a positive experience of enquiring from the presenting symptoms of children's exposure to IPV or following the disclosure of IPV by mother.[37 41 42 47]
		One young man preferred when HCP asks about 'safety at home' regardless of the presenting problem.[45]		Several practitioners suggested screening for IPV as part of routine pediatric exam.[41]
	1.1.4. Police notification to children's social services of IPV incidents when		Most mothers were not aware of the police notification.[35]	Some SSPs from children's social services thought that not all incidents of IPV with children present/ resided in the household need to be referred to

Descriptive theme with subthemes		Stakeholder group, study		
		Children	Parents	Professionals
	children were present/ resided in the household			them. However, they thought that they should be informed of such incidents.[35]
				Some SSPs from children's social services found police notifications inadequate due to the lack of details on children's exposure to IPV.[35]
				SSPs from children's social services found that "filtering out" police notifications to assess whether the family requires further intervention is too time- and labour-consuming.[35]
**2. Experiences of initial response to children's exposure to IPV**				
	2.1. Focus			Most HCPs felt that they should treat the mother-child dyad as a single entity where the needs of the mother were consistent with the child's health and safety needs.[37 41]
				Some HCPs felt conflicted when they had to prioritise the child's needs over mother's needs and confidentiality.[37 41]
				Some HCPs and most SSPs felt that if a child is at risk, they have to prioritise his/ her safety over the mothers' needs and confidentiality.[35 37]
	2.2. Emotional support		Mothers valued when SSPs provided a lot of encouragement.[46]	Some HCPs were positive about providing emotional support and 'leaving the door open' to

Descriptive theme with subthemes		Stakeholder group, study		
		Children	Parents	Professionals
				children to come back with any problems.[37 39 47]
	2.3. Education		Mothers valued information materials on the impact of IPV on children and IPV resources.[43]	HCPs and SSPs emphasised the importance of educating mothers about the impact of IPV on children and professional responsibilities in cases of children's exposure to IPV. Professionals used education to increase mother's engagement in services.[35 37 41 42 46]
				HCPs emphasised the importance of educating mothers about the purpose of the home visitation programme and children's social services. Professionals saw education as a pathway to easing mothers' fears of child removal as a result of professionals' involvement.[42]
				SSPs expressed mixed views on the acceptability of sending parents materials about impact of IPV on children in response to the police notification without any other intervention to families.[35]
2.4. Safety	2.4.1. Understanding of safety		Mothers thought that involvement of SSPs resulted in increased risk of abuse escalation and child removal.[32 44 46]	SSPs understood women's and children's safety as being out of the abusive relationship and believed that their involvement resulted in increased safety for both mothers and children.[44 46]

Descriptive theme with subthemes	Stakeholder group, study		
	Children	Parents	Professionals
		Some actions that mothers perceived as preventing abuse escalation and increasing child safety (e.g., staying with perpetrator, not seeking help) were seen by SSPs as increasing risk for children.[44 46]	Some actions that mothers perceived as increasing child safety were seen by SSPs as increasing risk for children.[44 46]
		Mothers did not feel safer after they complied with SSPs' requirements and left the abusive partner.[44 46]	
		Women wanted to talk with children's social workers about safety and safety planning.[46]	
2.4.2. Assessment of risk	One child suggested that HCPs need to ask children about their safety in all the environments that a child inhabits (e.g., all households post-separation, school).[45]		
			SSPs and HCPs favoured varied approaches to risk assessment (e.g., observation, information gathering from mother and other professionals, gut feeling, previous experiences).[35 37 46]

Descriptive theme with subthemes		Stakeholder group, study		
		Children	Parents	Professionals
				Some SSPs found standardised tools helpful for risk assessment (e.g., the Barnardo's Domestic Violence Risk toll).[35]
				Some SSPs saw the existing risk assessment as a bureaucratic exercise and believed that it does not measure real risk from the perpetrator[35 40 46]
	2.4.3. Safety planning		Most mothers were not involved in safety planning with their SSPs therefore could not comment on its acceptability.[46]	SSPs found undertaking safety planning with both children and mothers to be acceptable.[35]
				Although SSPs understood that they could not change women, they believed that leaving an abusive partner is the most effective form of safety planning.[46]
				Some SSPs felt that they monitor the risk instead of doing direct safety planning with mothers and children and wanted more direct engagement with them.[35 40]
				Some HCPs found it acceptable to undertake safety planning with women who were not ready to leave the perpetrator.[41]

Descriptive theme with subthemes		Stakeholder group, study		
		Children	Parents	Professionals
	2.5. Signposting	Children appreciated when social workers signposted them to other services.[35]	Mothers appreciated when health visitors signposted them to other services.[35]	Two GPs felt comfortable when giving children information about services they could access.[37]
				Some HCPs and SSPs felt comfortable providing mothers with information about IPV resources. They saw signposting as a mechanism to facilitate engagement in services.[35 37 46]
	2.6. Documenting			Most HCPs reported uncertainty and confusion about recording policies and practices on children's exposure to IPV. The uncertainty was reported at local, region and national levels.[36 37 41 42]
				HCPs feared possible breach confidentiality of parents exposed to IPV by recording children's exposure in child or parent medical records.[36 37]
	2.7. Reporting			HCPs described uncertainty and confusion about their reporting responsibilities especially in cases of child exposure to psychological IPV and non-direct physical IPV.[37 41 42]

Descriptive theme with subthemes		Stakeholder group, study		
		Children	Parents	Professionals
			Mothers felt uncertain about reporting responsibilities of HCPs and feared that HCPs could initiate child removal.[42]	HCPs emphasised the importance of explaining their reporting responsibilities and procedures to mothers. Professionals use such education to ease mothers' fears of child removal, to increase engagement with services, and to justify breach of the mothers' confidentiality after IPV disclosure.[37 42]
**3. Factors that facilitated identification and initial response to children's exposure to IPV**				
	3.1. Mother's readiness to disclose and engage		Mothers reported past experiences when they were not ready to acknowledge the impact of IPV on their children, disclose IPV and engage with services.[35 43 44]	HCPs and SSPs believed that the approach to identification and initial response should match mother's readiness to disclose IPV and engage with services.[35 41 46]
	3.2. Patient-professional relationship	Most children emphasised the importance of 'knowing and being known' to the doctor, which made them feel comfortable to talk about personal issues.[45]	Most mothers emphasised the importance of a long-term trusting relationship with the same professional for IPV disclosure and engagement with services.[32 44-46]	Most HCPs and SSPs emphasised the importance of a long-term trusting relationship with mother for IPV disclosure and engagement with services.[41 46]
			Mothers suggested that they could be role models for their children in developing trust and building	Only one HCP suggested that existing relationships could enable proactive and direct engagement with children.[37 39]

Descriptive theme with subthemes		Stakeholder group, study		
		Children	Parents	Professionals
			relationship between child and doctor.[45]	
				Some HCPs thought that referral to children's social service can worsen their relationship with a parent who discloses IPV.[37 39 41]
				Some HCPs feared that professionals with strong relationships with their patients are more likely to develop vicarious trauma.[41]
	3.3. Positive professional attitude	Children described preferred professionals as good at listening, making them feel that they were welcome back, helping them practically.[34 35 45]	Mother appreciated HCPs and SSPs who took them seriously, validated their accounts, ensured confidentiality and helped practically.[32-35 42 44 46]	HCPs emphasised the importance of nonthreatening and non-judgemental attitudes with frequent reassurance of confidentiality when responding to IPV disclosure.[37 41 42]
3.4. Engaging directly with [illegible]	3.4.1. Talking to children at consultation	Children appreciated when professionals talk directly to them, instead of to their mothers.[34 35 45]	Mothers stressed the importance of giving each child a voice and addressing each child's needs at a medical consultation.[45]	Most HCPs expressed concerns in children's competence as informants and believed parents more than children.[37 39]
				Some HCPs expressed concern about their lack of competence in communicating directly with children.[37]

Descriptive theme with subthemes		Stakeholder group, study		
		Children	Parents	Professionals
				A few HCPs who directly engaged with children were female general practitioners in affluent areas with high levels of IPV service provision.[39]
				SSPs felt confident talking to children when assessing and intervening in families with IPV.[35]
				SSPs felt that they should listen to the child more when assessing and intervening in families with IPV.[40]
	3.4.2. Appropriate age for talking to children at consultation	Most children regardless of age preferred their mother (trusted adult) to be present in a medical consultation.[45]	Mothers found it acceptable if doctors speak to children from the age of five. They believed that children exposed to IPV mature quicker than non-exposed children.[45]	There was no age above which clinicians consistently agreed it would be acceptable to talk to children about IPV; some were hesitant about even talking to teenagers.[37 39]
			Mothers preferred doctors to use non-verbal methods when communicating with young children.[45]	
			Mothers of adolescents believed that their children would find it difficult to talk to doctors because of teenage behavioural traits.[45]	

Descriptive theme with subthemes		Stakeholder group, study		
		Children	Parents	Professionals
	3.4.3. Seeing children alone	Some children felt that they should be of adolescent-age before speaking alone with the doctor about their safety and well-being.[45]		Most general practice clinicians felt that they should not see children alone at consultation.[37 39]
		Most children wanted their mothers (trusted adult) to be present at the medical consultation.[45]		Few general practice clinicians who would see children alone thought that it is acceptable to seek permission from the non-abusive parent and from the child without describing how it should be done.[37]
				SSPs regularly saw children alone when assessing risk, although they found it challenging to talk to children alone.[35]
**4. Reasons for not identifying/ disclosing children's exposure to IPV and not engaging with services**				
	4.1. Awareness of children's exposure to IPV		Mothers recognised that it took long time to acknowledge that IPV had an impact on their children.[32 35 43 44 46]	Most HCPs felt uncertain and confused about what constitutes children's exposure to IPV in cases of psychological IPV and non-direct physical IPV and how to respond to such cases.[37 42]
			Mothers felt that SSPs are unaware of the dynamics of IPV and risks involved.[32 34 35 44 46]	SSPs believed that mothers' underestimating of the impact of IPV on children was a barrier to disclosure and engagement with services.[34 35 46]

Descriptive theme with subthemes		Stakeholder group, study		
		Children	Parents	Professionals
4.2. Perceived role	4.2.1. Someone else's	Most children believed that HCPs' role is to treat illness and monitor their health and well-being, although health was narrowly defined as physical (e.g., injuries, vaccination).[45]	Mothers believed that HCPs' role is to treat illness and monitor their health and well-being, although health was narrowly defined as physical (e.g., injuries, vaccination).[45]	HCPs believed that Identifying and responding to children's exposure to IPV is someone else's role (e.g., health visitor, social worker, school nurse).[35 37]
		Two children recognised a potential role for HCPs in caring about children beyond physical illness.[45]		
				HCPs and SSPs believed that it is a mother's role to seek help and stop IPV.[35 37 40 42 44 46 47]
	4.2.2. Threatening and punitive	Children perceived children's social service's role as threatening and punitive.[34 35]	Mothers perceived children's social service's role as threatening and punitive.[34 35 42 46]	HCPs and SSPs perceived children's social service's role as threatening and punitive and recognised that mothers and children felt the same.[34 35 42]
				SSPs perceived health visitors as less threatening and more acceptable to families.[35]
	4.2.3. Uncertainty		Some mothers felt confused and uncertain about the role of children's social services which led to anxiety and avoidance of HCPs and SSPs.[34 35]	

Descriptive theme with subthemes		Stakeholder group, study		
		Children	Parents	Professionals
	4.2.4. Mandatory reporting			HCPs felt confused and uncertain about their role as mandated reporters of children's exposure to IPV, especially in cases of psychological IPV and non-direct physical IPV.[42]
			Mothers perceived all HCPs as mandated reporters of children's exposure to IPV which led to anxiety and avoidance of HCPs.[42]	HCPs recognised that their role as mandated reporters prevented mothers from disclosing IPV and engaging with services.[42]
	4.3. Fears	Children feared being removed from the home and taken into care.[34 35 40]	Mothers feared the removal of children by social services.[32-35 42 44 46]	HCPs and SSPs recognised that varied fears prevented mothers and children from disclosing IPV and engaging with services.[35 42 46]
		Children feared the negative consequences of IPV disclosure (e.g., escalation of abuse, involvement of children's social services).[40 45]	Mothers feared the negative consequences of IPV disclosure (e.g., escalation of abuse, involvement of police and children's social service).[44 45]	HCPs feared the negative consequences of identifying and responding to children's exposure to IPV (e.g., escalation of abuse, misunderstanding, involvement of children's social service, threats from parents, damage to the doctor-patient relationship, legal consequences).[35-37 39 41 47]
			Mothers feared HCPs because of their mandated reporter responsibilities.[42]	HCPs recognised that mothers feared them because of their mandated reporter responsibilities.[42]

Descriptive theme with subthemes		Stakeholder group, study		
		Children	Parents	Professionals
	4.4. Stigma	Children recognised the stigma, shame, and embarrassment associated with disclosing IPV.[34 35]	Mothers recognised the stigma, shame and embarrassment associated with disclosing IPV.[34 35 43 45]	
4.5. Influences from the wider environment	4.5.1 Social and cultural factors		Mothers who were non-native speakers expressed the need for information on children's exposure to IPV in their native language.[43]	HCPs and SSPs recognised that language and cultural norms and stereotypes make it difficult to engage with mothers from ethnic minorities.[35 41]
			Immigrant mothers wanted materials on children's exposure to IPV that address the immigration issues and culture-specific fears of IPV disclosure.[43]	
	4.5.2. Healthcare and social care system factors			HCPs and SSPs perceived the lack of contact time with patients/ clients as a major barrier to identifying and responding to children's exposure to IPV.[37 40 41]
				HCPs did not perceive children's exposure to IPV as their top priority.[37]
				HCPs and SSPs highlighted that their work with children was negatively affected by the cutbacks and constant reforms in home visitation services and social services.[35 37]

Descriptive theme with subthemes		Stakeholder group, study		
		Children	Parents	Professionals
				HCPs and SSPs highlighted the lack of services for all family members which prevented them from asking about and responding to children's exposure to IPV.[35 37 41]
		A few children suggested that police and social workers should communicate better in cases of IPV to ensure children's safety and adequate support.[34 35]		HCPs and SSPs were concerned with the lack of communication between the different providers and expressed a need for better communication and a more coordinated response to children's exposure to IPV.[35 37 40 41]
**5. Psychological consequences of identification and initial response to children's exposure to IPV**				
	5.1. Emotional burden	Children felt disappointed and powerless as a result of negative experiences with SSPs.[34 35]	Parents felt disappointed, stressed, hopeless and frustrated as a result of negative experiences with SSPs.[32-35 44]	
		Children thought that involvement with SSPs was 'stressful' for their mothers.[35]		
			Mothers felt embarrassed and guilty when acknowledging the impact of IPV on their children and when disclosing IPV to professionals.[34 35 43 45]	HCPs and SSPs felt frustrated and conflicted towards mothers who did not follow their advice.[41 46 47]

Descriptive theme with subthemes		Stakeholder group, study		
		Children	Parents	Professionals
				HCPs felt conflicted when changing the focus from the mother-child dyad to the individual child.[37 41]
				HCPs and SSPs felt uncomfortable about pressuring mothers to leave the perpetrator to protect children, but thought that they had few options in situations where mothers and children continued to live with the perpetrator.[37 41]
	5.2. Vicarious trauma			HCPs feared the possibility of burnout and hardening towards women and children.[41 47]
		**6. Training and resources suggested**		
	6.1. Professional training			HCPs preferred interactive training built upon real cases.[37 38]
				HCPs asked for training on communication with children.[37 38]
				HCPs and SSPs wanted to learn about other services' roles and responsibilities in responding to children's exposure to IPV.[35 37 38]
	6.2. Information materials		Mothers favoured single posters in pediatric settings about signs of IPV, effect of IPV on children and local IPV services.[43]	HCPs found IPV posters in health care settings to be acceptable in facilitating IPV disclosure and self-referral to IPV services.[41]

Descriptive theme with subthemes	Stakeholder group, study		
	Children	Parents	Professionals
		Mothers who were non-native speakers favoured information materials in their native language.[43]	HCPs wanted local signposting materials on IPV services.[37 38]
		Mothers preferred information materials with positive non-judgemental message, representing ethnic and social diversity[43]	HCPs wanted materials on policies and procedures about mandatory reporting of children's exposure to IPV.[42]

Note. IPV – intimate partner violence. HCPs – health care professionals. SSPs – social service professionals..... – converging perspectives. .... – diverging perspectives. ... - conflicting perspectives.
