# Supplementary material for: Identification and initial response to children’s exposure to intimate partner violence: a qualitative synthesis of the perspectives of children, mothers and professionals
Source: BMJ Open. 2018 Apr 28;8(4):e019761. doi: 10.1136/bmjopen-2017-019761 (PMC5931305; doi:10.1136/bmjopen-2017-019761)
Supplement: Supplementary data [file bmjopen-2017-019761supp005.pdf]

Supplementary file 5

Interim analytical themes and their definitions

Interim analytical theme with subthemes	Stakeholder group, study			
	Children	Mothers	HCPs	SSPs
**1. Converging perspectives on satisfactory strategies for identification and initial response to children's exposure to IPV**				
**1.1. Facilitators of acceptable identification and initial response**				
**1.1.1. Patient-professional relationship.** Some children, most mothers and most professionals highlighted that central to any work around children's exposure to IPV were aspects of the patient-professional relationship. When describing a good relationship, stakeholders talked about the long-term process of relationship building, continuity of contact, trust and effective communication.	[45]	[32 44-46]	[37 41]	[46]
**1.1.2. Positive professional attitudes and skills.** Patients/ clients and HCPs described how a non-judgemental non-threatening attitude, showing respect, validation of patients' accounts and reassurance of confidentiality facilitated disclosure and engagement with services.	[35 45]	[32 35 42 44 46]	[37 41 42]	
**1.1.3. Safety & comfort.** Good patient-professional relationships enabled children and mothers to feel safe and comfortable when discussing sensitive issues.	[45]	[45]		
**1.1.4. Individual mother's readiness.** Mothers and professionals suggested that the individual mother's readiness to disclose IPV and seek help should be considered when asking about IPV and responding to disclosure.		[43 44]	[41]	[35 46]
**1.1.5. Patient materials.** Mothers and HCPs thought that printed materials for patients about the effect of IPV on children and local IPV resources should be displayed in health-care settings to facilitate IPV disclosure by mothers and provide information for self-help.		[43]	[41]	

Interim analytical theme with subthemes	Stakeholder group, study			
	Children	Mothers	HCPs	SSPs
**1.2. Identification process**				
**1.2.1. Consultation atmosphere.** Children and mothers desired permission, space and time to discuss sensitive issues with HCPs.	[45]	[45]		
**1.2.2. Approach to enquiry.** Most children and HCPs preferred a case-based approach (from presenting symptoms).	[45]		[37 41 42 45 47]	
**1.2.3. Phased inquiry.** Patients and professionals were positive about HCPs initiating the enquiry. They preferred professionals to ask about children's exposure to IPV as a 'safety-at-home' issue – starting from the presenting symptoms, through questions about general well-being and the family situation to questions about feeling safe at home.	[45]	[45]	[37 41 42 47]	
**1.3. Initial response process**				
**1.3.1. Emotional support.** Mothers and professionals were positive about providing a lot of encouragement and emotional support to both children and mothers.		[46]	[37 47]	
**1.3.2. Patient/client education.** Mothers and professionals were positive about patient/client education about the impact of IPV on children, IPV dynamics, professional roles and duties. Professionals highlighted the importance of considering women's safety when sending letters about the impact of IPV on children.		[43]	[37 41 42]	[35 46]
**1.3.3. Signposting.** Mothers, children, and professionals were positive about signposting mothers and children to IPV services.	[35]	[35]	[37]	[35 46]
**2. Converging perspectives on barriers to satisfactory identification and initial response to children's exposure to IPV and proposed solutions**				
**2.1. Fears.** All stakeholder groups shared varied fears. The fear of abuse escalation, involvement of children's social services and child removal were mentioned by all groups. Additional professional fears of	[35 40 45]	[32 35 42 44 46]	[37 41 42 47]	[35 46]

Interim analytical theme with subthemes	Stakeholder group, study			
	Children	Mothers	HCPs	SSPs
negative consequences included threats from parents, negative impact on the therapeutic relationship with parents and legal consequences. Professionals used education to ease mothers' fears.				
**2.2. Gaps in knowledge and skills**				
**2.1.1. Low awareness.** All stakeholder groups demonstrated low awareness about children's exposure to IPV, especially psychological and non-direct physical IPV and delegated responsibilities of identifying and responding to somebody else.	[35 45]	[32 35 43-46]	[37 42]	[35 46]
**2.1.2. Communicating with children.** HCPs wanted to improve their communication skills in talking directly to children during medical consultations.			[37]	
**2.1.3. Documenting.** Professional described uncertainty and confusion regarding how and where to document children's exposure to IPV.			[36 41 42]	
**2.1.4. Mandatory reporting.** Mothers' uncertainty and confusion about HCPs' duties as mandated reporters mirrored professional uncertainty and confusion, especially in cases of children's exposure to psychological and non-direct physical IPV.		[42]	[37 41 42]	
**2.2. Emotional burdens.** All stakeholders were emotionally involved in the work of identifying and responding to IPV. Clients reported feeling threatened and pressured by SSPs to leave the perpetrator without adequate support provided. Professionals described psychological distress caused by ambivalent feelings towards mothers when dealing with children's exposure to IPV and were concerned that involvement in helping abused women and their children could cause vicarious trauma.	[35]	[32 35 46]	[37 41 47]	[35]
**2.3. System level factors**				
**2.3.1. Language and culture.** Stakeholders described mothers' immigration status, community norms and poor English as additional barriers to identifying and responding to children's exposure to IPV.		[43]	[41]	[35]

Interim analytical theme with subthemes	Stakeholder group, study			
	Children	Mothers	HCPs	SSPs
**2.3.2. Inter-agency work.** Stakeholders were similarly concerned with the lack of communication between different providers and expressed a need for a better communication, information sharing and a more coordinated approach when responding to children's exposure to IPV.	[35]		[37 41]	[35 40]
**2.3.3. Stretched services.** Professionals repeatedly mentioned: (i) lack of contact time with patients/clients, (ii) high demand and competing priorities, (iii) constant reforms to health care and social services, and (iv) shortage of services for all family members.			[37 41]	[35 40]
**3. Suggested training and resources** Professional training and resources. Professionals favoured a case-based training, wanted to improve their communication skills, learn about the roles of and responsibilities of other services and highlighted the importance of inter-agency collaboration. HCPs requested clear instructions on mandatory reporting and local signposting materials			[36 37 41 42]	[35]
**4. Diverging perspectives on satisfactory initial response**				
**4.1. Safety.** Mothers and professionals disagreed on the understanding of safety for the child. Actions perceived by mothers as increasing children's safety were seen by SSPs as jeopardising it. Most SSPs believed that their involvement increased children's safety and required mothers to leave the perpetrator. In contrast, most mothers thought that SSPs' involvement posed a threat to the child through leading to IPV escalation and child removal. Women did not feel safer after leaving the perpetrator because of potential escalation of abuse and child contact.		[32 44 46]		[44 46]
**4.2. Talking directly to children.** While most recipients of care were positive about professionals talking directly to children from a young age and addressing their individual needs, most HCPs did not see children as patients on their own and assessed children's needs through a proxy adult. Children and HCPs had	[34 35 45]	[45]	[37]	[35 40]

Interim analytical theme with subthemes	Stakeholder group, study			
	Children	Mothers	HCPs	SSPs
conflicting views about the appropriate age for talking to children about IPV and about appropriateness of seeing children alone.				
**5. Conflicting perspectives on satisfactory identification and initial response**				
**5.1 Shifting focus.** HCPs and SSPs had conflicting perspectives on the focus of their responses – child vs mother-child dyad/family. Although all agreed that the focus should switch to the child when he/she is at risk of harm, some felt conflicted when prioritising children's safety and needs over mother's' safety.			[37 41]	[35]
**5.2. Assessment of risk.** While children wanted to be involved in risk assessment and some SSPs found it acceptable to talk to children when assessing the risk, most professionals were not satisfied with the existing risk assessment process and saw it as a ticking box exercise.	[45]		[37]	[35 40 46]
**5.3. Safety planning.** While some HCPs found it acceptable to undertake safety planning with women who were not ready to leave the abusive partner, most SSPs saw leaving the abusive partner as the best safety planning for the child. SSPs had mixed views on the current safety planning process. Mothers wanted better communication with SSPs when undertaking safety planning.			[41]	[35 40 46]

Note. IPV – intimate partner violence. HCPs – health care professionals, SSPs – social service professionals.
