# Supplementary material for: Identification and initial response to children’s exposure to intimate partner violence: a qualitative synthesis of the perspectives of children, mothers and professionals
Source: BMJ Open. 2018 Apr 28;8(4):e019761. doi: 10.1136/bmjopen-2017-019761 (PMC5931305; doi:10.1136/bmjopen-2017-019761)
Supplement: Supplementary data [file bmjopen-2017-019761supp006.pdf]

Supplementary file 6

Final analytical themes supported by raw qualitative data from included studies

Theme	Subtheme	Participant quotation	Authors interpretation quotation
1. Precursors for acceptable identification and response to children's exposure to IPV	1.1. Satisfying and sustainable relationship	"Have confidence around them...I guess it depends how much you know your doctor. It's just depending on how well you know 'em. (Zoe, 12)"[45]<br><br>"Well in domestic violence I find that it's always the relationship that we have with the client, with the mom in this case — the stronger our relationship with the mom and the more we can understand her, understand her world through her eyes instead of imposing our view, we get them more on board. Once we can get that trust, then we can work together. [Worker #11]"[46]	"In common with participants in the wider study, they [parents] placed a lot of value on the type of relationship they could build up with workers [child protection workers] and the degree of trust that developed thereafter."[32]
	1.2. Desired professional attitudes and skills	"My doctor was really good, like once I went there and she was like really concerned and she sent me to someone else and then she told me to come back in a week so she could check on me and then, you know…like she kept making appointments so she could see me again and again. So it's good when doctors care like that…Makes you want to then	"Where service users reported positive experiences with the child protection services, it was normally when they felt they had been listened to fairly and believed. They also appreciated it when workers took direct action."[32]

Theme	Subtheme	Participant quotation	Authors interpretation quotation
		maybe go and um always go to them. (Claire, 21)"[45]<br><br>"I'm grateful that they actually heard me, heard my voice and listened to me (Client#11)"[46]	
	1.3. Considering mother's readiness	"[I] lost the children—my three youngest girls plus my son. Because the Department saw that I—which, looking back now, I understand what they're saying— that I put my children in a dangerous situation … they could very well have gotten hurt. But I don't think I deserved to lose them—I needed help to get out of that situation and I asked for help to get out of that situation."[44]	"It was suggested that a comprehensive system of support could be achieved through coordination of education, safety, and support services with the stage of change of the caregiver (P 1 and 14). Whereas victims in the early stages needed information, victims in later stages needed shelter, support groups, or legal services (P 11 and 16)."[41]
	1.4. Patient materials	"Doctors should ask about exposure. We can invite the conversation through literature and posters. Some believe that we should not ask. It is not the doctor's role. Some people [victims] are not ready to do anything. For those people, you just say, "Here is some info. (P 14)"[41]	"We found that women with histories of IPV support positive educational interventions in the PED [Pediatric Emergency Department] and recommend interventions that educate about resources, signs of IPV, and the effects of childhood IPV exposure in a non-judgmental and positive manner."[43]
	1.5. Professional training	"…other agencies, they don't actually understand what our role is, you know…what would be really, really good is people should shadow…and they should do some training on what the role, the roles	"Participants voiced a need to deconstruct the misperception that CEDV [children's exposure to domestic violence] is harmless and to educate

Theme	Subtheme	Participant quotation	Authors interpretation quotation
		of different agencies are and what different agencies can actually offer. (Child protection social worker 4)"[35]	professionals, health care providers, judges, police officers, and school staff on the hazards of CEDV (P 19)."[41]
	1.6. Professional resources	"I think you need to have in your literature about mandated reporting because, as nurses, we are mandated reporters and we need to know, again, with that role it needs to be spelled out very clearly because in a court of law that's what would be thrown at us, if we have fulfilled our responsibilities in what way. (Nurse)"[42]	
	1.7. Professional supervision	"[...] I don't feel much impact, because, it's like, we get so many [children], recently there have been a lot, and so before we used to get more [impacted], but these days it is hard to go through one month in which you don't have one or two cases of poor treatment, sexual abuse [...] I have already had so many [cases] that we are beginning to become accustomed with the situation [...] (N15)."[47]	"Conversely, providers with strong relationships with their patients may be more susceptible to frustration, burnout, or hardening toward victims who are not ready to leave abusive relationships." (P 19).[41]
	1.8. Addressing systems' barriers	"I know people can be very protective about information sharing. 'Well *you're* working with the adult' and '*You're* working with the child', so there is	"Some of the reasons given for not exploring the possibility of DVA [domestic violence and abuse] when child safeguarding concerns arose included DVA not being 'first on your radar or list of things to

Theme	Subtheme	Participant quotation	Authors interpretation quotation
		not always that joined-up thinking about risks as a whole. (Specialist Social Worker: 1)"[40]	ask about' (GP31) and the problem of 'finding the time to do [it] all' (GP28)."[37]
2. Acceptable identification	2.1. Space and time	"It makes it a lot easier when they say that you can take your time. Like even for the first session…you don't have to do anything, you can just try and get used to being there and stuff. (Amelia, 15)"[45]<br><br>"Five years, same woman, all same—then I start to tell them everything then they said why you didn't tell me before? Then I tell them you know I was so scared that time like I feel safe with them to talk about everything then my children aid worker she find out shelter for me. [Client #9]"[46]<br><br>"The sense that it's okay to come and talk to you about anything that worries them. (GP21)"[37]	
	2.2. Vocabulary	"Ask the kids if they're OK…(Lisa, Claire's mother) Yeah, I mean that's exactly right (agreeing with Lisa in the focus group).<br>(Doctor) 'Are_you_OK?' (Patient) 'Oh no'. (Doctor) 'Why? Would you like to talk about it?' I think, and they're simple little words…(Penelope, mother)"[45]	

Theme	Subtheme	Participant quotation	Authors interpretation quotation
	2.3. Phased approach	"I brought up the fact that I wasn't sleeping like normal people and that it was affecting my school work because I really, really want to do well at school. So he kind of asked a few questions and stuff to find out what else is affecting me… (Amelia, 15)"[45]<br><br>"But there might be other things like sleeping issues, so then the doctor might go, 'OK, so why aren't you sleeping, do you think?' And then that might lead to the child opening up, 'Well this is happening and…Mum and Dad are fighting all the time and…(Lisa, mother)"[45]<br><br>"If they start talking about domestic violence then at that point engage them in the discussion about, "Well, do you realize that that is a form of child abuse? Not that I'm going to report you today or anything but I want you to be aware of that. (Community stakeholder)"[42]	
3. Acceptable initial response	3.1. Shifting focus	"We are child focused so this is what you need to do to keep your children safe, but obviously you need to be safe to do that, so it sort of interlinks, but	"Throughout the interviews, it became apparent that although the project focused on children, practitioners treated the mother–child dyad as a

Theme	Subtheme	Participant quotation	Authors interpretation quotation
		certainly the children are our first concern. I mean, you know, if the victim is unable to protect their child, then we need to look at whether there is more action that we need to do, something a bit sort of formal as opposed to giving advice. (Initial assessment manager 4)"[35]<br><br>"I'd have to tell her that, because of the children, I would basically be needing to involve child protection services. I would reassure and inform her as much as possible as to what that would involve, but yes, I'd have to make her aware that, you know, it wasn't actually up to her whether I, because I often get into arguments about 'oh no, but they'll take the children… (GP30)"[37]	single entity where the needs of the mother were consistent with the child's health and safety needs."[41]
	3.2. Emotional support	"But, within my capabilities, what I did was intensify surveillance, and try my best to help the child emotionally [...] (N4)."[47]<br><br>"she used to give me a lot of encouragement, tell me I was doing a good job in spite of you know all the circumstances?" "just thinking of her words does	

Theme	Subtheme	Participant quotation	Authors interpretation quotation
		help you know, when I'm being a bit hard on myself as a mom (Client #8)"[46]	
	3.3. Education	"Initial care of the mother–child consisted of gathering information, helping the mother to view the relationship honestly and objectively, educating the mother on potentially dangerous situations and ways to avoid or prevent abuse. Documenting provider efforts in the patient chart facilitated follow-up. (P 15)"[41]<br><br>"There's already a lot of anxiety if you know talk about Children Services. . . . Hopefully [CPS] could be seen as more of a positive support for this mom and the children. . . . One thing is just to educate and talk to moms about what role Children Services can play, and seeing it more as a positive not just a negative experience. . . .Educate that, you know, Children Services is here to help, Children's Services isn't here to remove your kids. (Community stakeholder)"[42]	
	3.4. Signposting	"She was really helpful, …, she was the one that gave us the number for the NSPCC. She was just good at	"Two examples were given of GPs overcoming the lack of time and giving young people information

Theme	Subtheme	Participant quotation	Authors interpretation quotation
		listening to us and that. (Nicola, Young People's Focus Group 1)" [35]	directly about relevant services they could access."[37]
4. Conflicting perspectives on acceptable identification and response	4.1. Engaging directly with children	"It depends on how they (doctors) approach them. And you've got to treat them as an individual, not as a (sibling) group." (Focus group participants all agree).(Charlie, mother)"[45]<br><br>". . . for me it's opening the opportunities for children to speak. Because key to it is understanding the children's view of it. The parents can say, 'Oh I love him, I'll never do it again'. . . . What's happening for the kid within all this? (Senior Social Worker: 2)"[40]<br><br>"When you complete initial assessments you do see the children alone and often they are quite young, and direct questions are very difficult. You are only in the house for an hour, an hour and a half doing your initial assessment. They usually know why this lady is coming to the house and they have been told not to say anything. You always ask if there is anything they would like to talk about, how are things at home, you know, what happens if they are naughty, do they go	"Lisa and Penelope suggest five as a suitable age to engage directly with children about their safety based on the level of awareness that their own children had at that age."[45]<br><br>"Whilst not precluding the doctor from addressing a child directly in a consultation where their mother is present, all the children and young people suggested an older age range [15-18] for being seen on their own."[45]<br><br>"Few clinicians would routinely seek to directly engage with the children concerned. Some might in some circumstance, after undertaking other actions such as talking to others (e.g. health visitors); or if an opportunity arose or excuse could be made. More than half would not seek to engage directly with children."[37]

Theme	Subtheme	Participant quotation	Authors interpretation quotation
		on the naughty stair, or, you know, but it's very difficult. (Initial assessment worker 3)"[35]	
	4.2. Management of safety	"AM: And in what way can doctors and nurses help families?<br>By checking if they're sick. Checking… if they're OK, feeling safe and all that.<br>AM: OK, what would be the best way that they could ask you if you were feeling OK?<br>Um…are you safe at home and at school and that…and at Dad's. (Fred, 9)"[45]<br><br>"We talk a lot about options if she is ready to leave [the abusive situation]. The options, if she is not ready to leave, a lot has to do with education, what are the dynamics of domestic violence, helping her to understand what is going on in her relationship, helping her to look at the cycle of violence, and to begin to recognize what is going on. A lot of what we do, if somebody is not ready to leave, has to do with safety planning and not safety planning about leaving, but safety planning while you are in the relationship. . . . If a violent incident happened, where can you tell the kids to go? Can the kids go	"Although formally asked about safety plans, many women either had not been involved in safety planning (or did not recall doing so) with their workers."[46]<br><br>"Similar to the victims who decided against leaving after approaching CPS [child protection service] for support, most victims who remained silent altogether believed they were acting in the best interest of their children at the time…. Some of the non-help-seeking decisions of victims are therefore well-informed decisions, often based on victims' individual risk assessment and their perception of how they can best ensure their children's safety at that particular point in time."[44]<br><br>"A common theme across the interviews with practitioners was the need for a shift towards more direct working with children and their parents. There was a widespread belief that current policy focused on monitoring risk, rather than addressing risk

Theme	Subtheme	Participant quotation	Authors interpretation quotation
		upstairs? Can you teach them to dial 911? Can you teach them to leave and go to a neighbor's house? Who can the kids talk to? How do you talk to your kids about what is going on? And then, how do you get yourself safe? Not staying in the kitchen, not being in the bathroom, not staying in a place where you don't have an exit, not wearing scarves around your neck because they can be used to choke you. There are lots and lots . . . and for every person the safety plan is going to look a little bit different. (P 2D)"[41]	through systematic safety planning with adult victims and direct safety planning with children."[40]
