# Supplementary material for: Identification and initial response to children’s exposure to intimate partner violence: a qualitative synthesis of the perspectives of children, mothers and professionals
Source: BMJ Open. 2018 Apr 28;8(4):e019761. doi: 10.1136/bmjopen-2017-019761 (PMC5931305; doi:10.1136/bmjopen-2017-019761)
Supplement: Supplementary data [file bmjopen-2017-019761supp002.pdf]

Supplementary file 2

Characteristics of the 11 studies (16 papers) synthesised (in chronological order)

N	Study<br>Paper(s)<br>Country	Aim	Setting	Participants, n	Intervention(s)	Data collection	Data analysis	Primary themes relevant to the synthesis	M-CASP score/ Yes-No-Not sure
1	Buckley 2008[a]<br>Buckley 2008 (report) [33]<br>Buckley 2011 (article)<br>[32]<br>Republic of Ireland	Service users' experiences of child protection services	Women's refuges, advocacy groups, family support services, treatment services, counselling service, public redress bodies, community youth project, and statutory child protection service in 16 counties	27 parents<br>20 female, 7 male<br>18 survivors of IPV	Notification by other services<br>Self-disclosure<br>Self-referral<br>First contact and initial response from child protection services	Interviews	Grounded-theory	Positive and negative experiences with child protection services.<br>Impact of those experiences on parents and children.	17-3-0

N	Study<br>Paper(s)<br>Country	Aim	Setting	Participants, n	Intervention(s)	Data collection	Data analysis	Primary themes relevant to the synthesis	M-CASP score/ Yes-No-Not sure
2	Black 2010 [41],<br>US	Recommended practices and obstacles to practices to improve interventions for families suffering from IPV	Varied health care services in one large urban area	Mixed sample of professionals including 24 HCPs and SSPs<br>20 female, 4 male	IPV screening<br>case finding of children's exposure to IPV<br>Initial care of the mother–child dyad	Interviews	Not stated	Individual, social, and system levels obstacles to identifying and responding to children's exposure to IPV.<br>Participants recommendations on how to improve identification and response to children's exposure to IPV.<br>Focus on supporting the mother–child dyad.<br>Challenges of focusing on the	14-6-0

N	Study Paper(s) Country	Aim	Setting	Participants, n	Intervention(s)	Data collection	Data analysis	Primary themes relevant to the synthesis	M-CASP score/ Yes-No-Not sure
								mother-child dyad.	
3	Stanley 2010[a]<br>Stanley 2010 (report) [35]<br>Stanley 2012 (article) [34]<br>UK	Experiences and views of police notifications to children's social services of IPV incidents where children were present/resided in the household	IPV services for women, services for children affected by IPV, children's social services in two local authorities in the North and South of England	19 children: age 10-19; 8 male, 11 female; 16 white British, 1 white/ Asian, 1 white/ black Caribbean, 1 white/ black African.<br>11 parents survivors of IPV: 1 male, 10 female; mean age 38 (25-48); most black minority ethnic (BME), 4 white British.<br>25 SSPs: age 23-65; 4 male, 21 female; 14 front-line, 22 white British, 1 black British, 1 black African, 1 "other";	Notification by police<br>Response from children's social services to the police notifications	Focus groups; interviews	Grounded theory	Clients' positive and negative experiences with children's social services and health professionals when disclosing IPV.<br>Barriers and facilitators to disclosing and acknowledging IPV.<br>Individual, organisational and system level barriers to responding to the police	14-6-0

N	Study<br>Paper(s)<br>Country	Aim	Setting	Participants, n	Intervention(s)	Data collection	Data analysis	Primary themes relevant to the synthesis	M-CASP score/ Yes-No-Not sure
				job experience 1.5-30 years				notifications of children's exposure to IPV.	
4	Meyer 2011 [44]<br>Australia	Help-seeking decisions of IPV victims with dependent children	IPV services, family counselling services in three areas in Southeast Queensland	29 parents survivors of IPV<br>All female<br>Age 21-62 (mean 38, SD 11.3).<br>21 Australian-born, 2 New Zealand-born, 3 UK-born, 1 South African, 1 Malaysian, 1 Filipino.<br>27 had children. 25 children had witnessed the abuse.<br>16 secondary education, 3 in tertiary education, 10 completed tertiary education.	Initial response from child protection services	Interviews	Grounded theory	Gap between client and professional understanding of the dynamics of IPV and response needed.<br>Parents' fear of harm and loss of custody.	17-3-0

N	Study Paper(s) Country	Aim	Setting	Participants, n	Intervention(s)	Data collection	Data analysis	Primary themes relevant to the synthesis	M-CASP score/ Yes-No-Not sure
				7 employed, 2 unemployed, 2 volunteers, 14 home duties, 3 students, 1 retired.<br>26 received government benefits.					
5	Randell 2012 [43]<br>US	Important information to communicate about IPV and how it should be presented in a pediatric emergency department	IPV service in a racially and ethnically mixed city	99 parents survivors of IPV<br>All female<br>36 White, 32 Hispanic, 22 African American, 5 other, 4 not reported.<br>52 high school, 21 degree, 26 not reported.<br>45 currently employed	Identification and response in health care setting	Focus groups	Grounded theory	Content and format of posters about children's exposure to IPV.	15-5-0

N	Study Paper(s) Country	Aim	Setting	Participants, n	Intervention(s)	Data collection	Data analysis	Primary themes relevant to the synthesis	M-CASP score/ Yes-No-Not sure
6	Davidov 2012 [42] US	Issues related to the mandatory reporting of children's exposure to IPV	Community based Nurse-Family Partnership (NFP) programme in four sites	20 NFP mothers survivors of IPV; mean age 21.3; 50% White, 25% Black, 15% Hispanic; 90% single/ never married; years of school 11.7; 80% income<$24,000<br>47 HCPs:<br>25 NFP nurses; mean age 46.2; 92%>=bachelor's degree; nursing experience 19.7 (5-38); years in NFP 4.4.<br>22 community stakeholders including 4 from hospital or public health services; work	Mandatory reporting of children's exposure to IPV by NFP nurses	A secondary analysis of focus groups and interviews	Retrospective interpretation	Professional and patient uncertainty regarding reportable children's exposure to IPV.<br>Mothers' fears of losing custody of children.<br>Strategies nurses can use to ease mothers' fear.<br>Negative client experiences with child protection services.	17-3-0

N	Study<br>Paper(s)<br>Country	Aim	Setting	Participants, n	Intervention(s)	Data collection	Data analysis	Primary themes relevant to the synthesis	M-CASP score/ Yes-No-Not sure
				experience 9.2 years; mean age 50.5 years.					
7	Angelo 2013<br>[47]<br>Brazil	Experience of the nurses in their care of child victims of IPV	Pediatric emergency, intensive care and inpatient units in São Paulo	15 HCPs<br>14 female, 1 male; ages 27-48, time since graduation 3-12 years	Case finding<br>Initial response	Interviews	Motivational theory	Identification of children's exposure to IPV through attention to signs and symptoms followed by verification.<br>Ambivalent feelings towards parents of children exposed to IPV.<br>Protective professional attitude towards children.	13-7-0
8	Jenney 2014<br>[46]<br>Canada	How the voices of women with experience of	Child protection services in five Ontario child	17 CPS workers: 12 females, 5 males; job experience >6 years;	Notification by other services	Focus group; interviews	Grounded theory	The process of interaction between worker	16-4-0

N	Study<br>Paper(s)<br>Country	Aim	Setting	Participants, n	Intervention(s)	Data collection	Data analysis	Primary themes relevant to the synthesis	M-CASP score/ Yes-No-Not sure
		IPV are translated into child protection service workers' interventions	welfare agencies	qualifications - BA (3), BSW (8), CYW (1), and MSW (5); 9 did not have DVA training, 4 had training in both risk assessment and safety planning.<br>11 parent survivors of IPV: all female; age 26-49 years; 5 immigrant/refugee; 8 on < $25,000 annually; 5 College/University education, 2 high school, 4 grades 9–11.	Self-referral<br>Response from child protection services			and client during child protection service investigation.<br>Gap between client and professional understanding of the concept of safety and the impact of IPV on children.	
9	Interview study within the RESPONDS Project[a]	Responses to disclosure of IPV when children are exposed and the dilemmas	General practices in six sites across the north and south	69 HCPs<br>42 general practitioners, 12 practice nurses and	Case-finding<br>Notification by other services	Interviews	Thematic analysis using framework	Engaging directly with children at medical consultation.	16-4-0

N	Study<br>Paper(s)<br>Country	Aim	Setting	Participants, n	Intervention(s)	Data collection	Data analysis	Primary themes relevant to the synthesis	M-CASP score/<br>Yes-No-Not sure
	Szilassy 2015 (report) [37]<br>Larkins 2015 (paper) [39]<br>Szilassy 2016 (paper) [38]<br>Drinkwater 2016 (paper) [36]<br><br>UK	and challenges general practice clinicians face when confronted with children's exposure to IPV	of England and the Midlands	15 practice managers<br>48 female, 21 male<br>Age: 21-34 – 10; 35-44 – 13; 45-54 – 30; 55-64 – 10; not known – 10<br>Experience managing domestic violence (number of cases): >5 – 5; a few – 14; 1 – 2, none – 26; none but aware of case at surgery - 7<br>IPV service provision: sparse – 28; established – 41<br>Location: metropolitan – 19; urban – 27; semi-rural – 23	Initial response from general practice clinicians			Professional uncertainty about responding to children's exposure to IPV.<br>Poor inter-agency work.	

N	Study Paper(s) Country	Aim	Setting	Participants, n	Intervention(s)	Data collection	Data analysis	Primary themes relevant to the synthesis	M-CASP score/ Yes-No-Not sure
				Region: north – 21; Midlands – 33; south – 33					
10	Clarke 2015 [40] UK	Practitioners' perceptions of children's experiences of IPV. Experiences of responding to the needs of children	Varied services in Wales	Mixed sample of 54 professionals from social services, health, education, police, probation, housing, IPV services and third sector organisations 5 parent survivors of IPV: all female	HCPs and SSPs responses to disclosure	Focus groups; interviews; case studies; meetings observations	Thematic analysis	Organisational and system level barriers to engaging directly with children and inter-agency work.	12-8-0
11	Morris 2015 (thesis) [45] Australia	Perspectives on children's safety and resilience when they live with IPV	General practices in Victoria	18 parent survivors of IPV: all female; mean age 43 (28-56) years; 2 born overseas. 23 children to 16 mothers survivors of IPV: mean age 14 (8-	Screening Case-finding Initial response from general practice clinicians	Interviews; focus groups	Hermeneutic Phenomenological analysis	Raising matters of safety or psychological well-being in the consultation. The type of relationships children and mothers expect	19-1-0

N	Study<br>Paper(s)<br>Country	Aim	Setting	Participants, n	Intervention(s)	Data collection	Data analysis	Primary themes relevant to the synthesis	M-CASP score/ Yes-No-Not sure
				24) years; 2 born overseas				to have with health professionals if they are to feel comfortable discussing family violence.<br>When, with whom and how safety conversations could be initiated.	

Note. a Indicates where more than one paper relates to a single study. IPV – intimate partner violence. HCPs – health care professionals. SSPs – social service professionals; NFP – Nurse-Family Partnership. CPS – child protection service; M-CASP – the modified version of the Critical Appraisal Skill Programme.[21 22] For studies reported in multiple papers, the total score was derived based on data extracted from all papers.
