# Supplementary material for: Identification and initial response to children’s exposure to intimate partner violence: a qualitative synthesis of the perspectives of children, mothers and professionals
Source: BMJ Open. 2018 Apr 28;8(4):e019761. doi: 10.1136/bmjopen-2017-019761 (PMC5931305; doi:10.1136/bmjopen-2017-019761)
Supplement: Author's manuscript [file bmjopen-2017-019761.draft_revisions.pdf]

BMJ Open

BMJ Open is committed to open peer review. As part of this commitment we make the peer review history of every article we publish publicly available.

When an article is published we post the peer reviewers' comments and the authors' responses online. We also post the versions of the paper that were used during peer review. These are the versions that the peer review comments apply to.

The versions of the paper that follow are the versions that were submitted during the peer review process. They are not the versions of record or the final published versions. They should not be cited or distributed as the published version of this manuscript.

BMJ Open is an open access journal and the full, final, typeset and author-corrected version of record of the manuscript is available on our site with no access controls, subscription charges or pay-per-view fees (http://bmjopen.bmj.com).

If you have any questions on BMJ Open's open peer review process please email info.bmjopen@bmj.com

BMJ Open

IDENTIFICATION AND INITIAL RESPONSE TO CHILDREN'S EXPOSURE TO INTIMATE PARTNER VIOLENCE: A QUALITATIVE SYNTHESIS OF THE PERSPECTIVES OF CHILDREN, PARENTS AND PROFESSIONALS

Journal:	*BMJ Open*
Manuscript ID	bmjopen-2017-019761
Article Type:	Research
Date Submitted by the Author:	22-Sep-2017
Complete List of Authors:	Lewis, Natalia; University of Bristol, Centre for Academic Primary Care, Population Health Sciences, Bristol Medical School; NIHR CLAHRC North Thames, Queen Mary University of London, Centre for Primary Care and Public Health<br>Feder, Gene; University of Bristol, Centre for Academic Primary Care, Population Health Sciences, Bristol Medical School<br>Howarth, Emma; University of Cambridge, NIHR Collaboration for Leadership in Applied Health Research & Care (CLAHRC) East of England<br>Szilassy, Eszter; University of Bristol, Centre for Academic Primary Care, Population Health Sciences, Bristol Medical School<br>McTavish, Jill; McMaster University, Department of Psychiatry and Behavioural Neurosciences<br>MacMillan, Harriet; McMaster University, Departments of Psychiatry and Behavioural Neurosciences, and of Pediatrics<br>Wathen, Nadine; Western University, Faculty of Information and Media Studies, and Centre for Research & Education on Violence Against Women & Children
**Primary Subject Heading**:	Public health
Secondary Subject Heading:	Health services research, Qualitative research
Keywords:	Child protection < PAEDIATRICS, PUBLIC HEALTH, QUALITATIVE RESEARCH, SOCIAL MEDICINE

SCHOLARONE™
Manuscripts

IDENTIFICATION AND INITIAL RESPONSE TO CHILDREN'S EXPOSURE TO INTIMATE PARTNER VIOLENCE: A QUALITATIVE SYNTHESIS OF THE PERSPECTIVES OF CHILDREN, PARENTS AND PROFESSIONALS

Corresponding author: Natalia V. Lewis, Canynge Hall, 39 Whatley Road, Bristol, BS8 2PS, UK; (44)1179287246, Nat.Lewis@bristol.ac.uk

Natalia V. Lewis, Centre for Academic Primary Care, Population Health Sciences, Bristol Medical School, University of Bristol, Bristol, UK and Centre for Primary Care and Public Health, Queen Mary University of London, National Institute for Health Research Collaboration for Leadership in Applied Health Research and Care North Thames at Bart's Health NHS Trust, London, UK

Gene S. Feder, Centre for Academic Primary Care, Population Health Sciences, Bristol Medical School, University of Bristol, Bristol, UK

Emma Howarth, National Institute for Health Research Collaboration for Leadership in Applied Health Research and Care East of England, Cambridge Institute of Public Health, University of Cambridge, Cambridge, UK

Eszter Szilassy, Centre for Academic Primary Care, Population Health Sciences, Bristol Medical School, University of Bristol, UK

Jill R. McTavish, Department of Psychiatry and Behavioural Neurosciences, McMaster University, Hamilton, ON, Canada

Harriet L. MacMillan, Departments of Psychiatry and Behavioural Neurosciences, and of Pediatrics, McMaster University, Hamilton, ON, Canada

Nadine Wathen, Faculty of Information and Media Studies, and Centre for Research & Education on Violence Against Women & Children, Western University, London, ON, Canada

Word count: 4393 (excluding title page, abstract, tables, figures & references).

For peer review only

ABSTRACT

Objectives: to synthesise research evidence on the acceptability of identification of and initial responses to children's exposure to intimate partner violence (IPV) in healthcare and social care settings.

Design: thematic synthesis of qualitative studies. We appraised the included studies with the modified Critical Appraisal Skills Programme checklist and undertook a sensitivity analysis on a subsample of the studies scored in the top tertile.

Data sources: we searched eight electronic databases, checked references and citations, and contacted authors of the included studies.

Eligibility criteria: qualitative research where participants were healthcare or social care professionals, or children, or parents and which described experiences of identification or initial responses to children's exposure to IPV.

Results: searches identified 1951 records; 16 papers reporting on 11 studies with 42 children, 220 parents and 251 professionals were included. Seven studies scored in the top tertile. Integrated perspectives of participants showed that sufficient training and support for professionals, good patient-professional relationship and supportive environment for patient/clients need to be in place before enquiry/disclosure of children's exposure to IPV should occur. Providers and recipients of care favour a phased enquiry about IPV initiated by healthcare professionals, which focuses on "safety at home" and is integrated into the context of the consultation or visit. Participants agreed that an acceptable initial response prioritises child safety and includes emotional support, education about IPV and signposting to IPV services. Participants had conflicting perspectives on what constitutes acceptable engagement with children and management of safety. Sensitivity analysis produced similar results.

Conclusions: Healthcare and social care professionals should receive sufficient training and on-going individual and system-level support to provide acceptable identification of and initial response to children's exposure to IPV. Ideal identification and responses should use a phased approach to enquiry and WHO LIVES principles integrated into a trauma- and violence-informed model of care.

For peer review only - http://bmjopen.bmj.com/site/about/guidelines.xhtml

STRENGTHS AND LIMITATIONS OF THIS STUDY

- This is the first synthesis of qualitative studies focusing on the integrated perspectives of patients/clients and healthcare and social care professionals on the acceptable identification and initial response to children's exposure to IPV.
- We retrieved relevant studies through a comprehensive search strategy, including electronic searches, citation and reference checking and contacting experts.
- Involvement of two reviewers throughout screening, data abstraction and critical appraisal of each study ensured methodological rigour of this review. Reviewers' backgrounds in different disciplines broadened and enriched the interpretation of data.
- Thematic synthesis allowed us to: (i) integrate perspectives of all participant groups and generate new interpretations going beyond the findings from primary studies, (ii) identify gaps in evidence within and across participant groups; (ii) establish areas of conflicting perspectives which can be targeted in development of interventions and future research.
- Exclusion of papers published in languages other than English and exclusion of non-peer reviewed reports could result in missing some relevant studies.

For peer review only

For peer review only - http://bmjopen.bmj.com/site/about/guidelines.xhtml

INTRODUCTION

Intimate partner violence (IPV) is a violation of human rights and widespread public health problem that affects approximately one third of women globally. IPV has detrimental effects on physical, mental and reproductive health of women and has a negative impact on their children.[1 2] Children's exposure to IPV can occur through direct involvement and witnessing or through indirect exposure (e.g., being aware of the violence between parents/caregivers, financial consequences, parenting affected by IPV).[3 4] The prevalence of witnessing a parent assaulting another parent in the United States is 5.8% in the past year and 25% over the life-time.[5] Children's exposure to IPV is strongly associated with child maltreatment, and has similar negative impact on children's physical and mental health, behaviour and academic attainment.[6] Healthcare professionals (HCPs) and social care professionals (SCPs) have an important role in identifying and responding to adult patients/clients and their children exposed to IPV.[7-9]

Identification of children's exposure to IPV in healthcare or social care settings can occur when the abused parent or caregiver seeks help, when children undergo assessment for behavioural problems, or when other services notify healthcare and social care providers about IPV occurrence. The WHO IPV guidelines recommend a case-finding approach to identifying women exposed to IPV: healthcare providers asking those women who present with indicators or clinical associations of IPV about safety in their relationship and at home.[10] There is no equivalent guidance on effective and acceptable approaches to identifying and responding to children's exposure to IPV and limited evidence on which to base that guidance.[6]

This review is one of a series undertaken for the **V**iolence, **E**vidence, **G**uidance and **A**ction (VEGA) Project, informing pan-Canadian public health guidance on family violence.[11] The objectives of the present systematic review were to identify, appraise and synthesise research evidence on the acceptability of the identification and initial responses to children's exposure to IPV in healthcare and social care settings. The synthesis addressed the following research questions:

1. What approaches to identification of children's exposure to IPV are acceptable to children, non-abusing parents and professionals?
2. What initial responses to children identified as being exposed to IPV are acceptable to children, non-abusing parents and professionals?

For peer review only - http://bmjopen.bmj.com/site/about/guidelines.xhtml

METHODS

We focused on qualitative evidence because we wanted to understand *how* providers and recipients of care perceive approaches to identification and responses to children's exposure to IPV and *why* they find them acceptable or otherwise.[12] This systematic review follows the Centre for Reviews and Dissemination (CRD) guidance[13] and adheres to the ENTREQ statement for reporting synthesis of qualitative research (see online Supplementary file 1).[14]

Eligibility criteria

Inclusion criteria are summarised in Table 1. Multiple papers from the same study were included if they each reported new data relevant to the research questions. Studies reported in books, conference papers, editorials, letters or general comment papers were excluded.

Table 1. Inclusion criteria for selecting studies

**Category**	**Inclusion criteria**
Population (further called stakeholders)	• Providers of health care or social care (further called healthcare professionals (HCPs) and social care professionals (SCPs) respectively OR<br>• Recipients of health care or social service (further called patients or clients respectively):<br>– Children (however defined in primary studies) who have been exposed to IPV OR<br>– Non-abusing parents of children who have been exposed to IPV
Intervention	• Identification of children' exposure to IPV by any methods(s) – screening, case-finding, notification by other services, self-disclosure OR<br>• Initial response to children's exposure to IPV that followed the identification and occurred before referral to another professional or service
Phenomena of interest	• Views of and direct experiences with identification and initial response to children's exposure to IPV
Types of studies	• Publication date: database inception to April 28th, 2016 AND<br>• English language AND<br>• Empirical qualitative (stand alone or components of mixed methods research) AND

For peer review only - http://bmjopen.bmj.com/site/about/guidelines.xhtml

Category	Inclusion criteria
	• Qualitative methods for data collection and analysis (e.g. interviews, focus groups, observations) AND<br>• Verbatim quotations from participants AND<br>• Papers that have undergone formal peer-review

Information sources

We aimed to retrieve relevant studies in the field through a comprehensive search and sampling strategy,[15] building on an earlier review by Howarth and colleagues in the IMPROVE 2013 evidence synthesis.[16] First, we retrieved full-text reports assessed in the IMPROVE synthesis. Second, we re-ran IMPROVE searches (28.04.2016) in eight medical, social science, social care and nursing databases (see online Supplementary file 2). Finally, the first reviewer completed forward and backward citation chaining of all included papers and emailed corresponding authors of the included papers (12.08.2016, 24.08.2016) to confirm peer-reviewed status of reports and signpost to additional relevant papers.

Studies selection

Two reviewers independently screened titles and abstracts of all references. The first reviewer screened all full text papers and the second reviewers screened a 10% subset. Disagreement was resolved through discussion and consensus.

Quality appraisal

Two reviewers independently assessed each study for methodological validity using the Critical Appraisal Skills Programme Qualitative checklist[17] modified for the purpose of the VEGA Project (M-CASP, score range 0-20).[18] Disagreements not resolvable between reviewers were resolved by a third reviewer. We did not exclude studies on reporting quality;[19] we conducted a secondary sensitivity analysis by restricting the synthesis to studies in the top tertile of methodological quality.[20]

Data extraction

We adapted a data extraction form from IMPROVE.[16] The first reviewer extracted study details; second reviewers checked the extracts. The papers were then treated as primary text transcripts. Two reviewers independently extracted raw qualitative data[21] relevant to the views and direct experience of identification and initial response to children's exposure to IPV from Results sections

For peer review only - http://bmjopen.bmj.com/site/about/guidelines.xhtml

of the included papers. These data could be in the form of participants' quotes or authors' interpretations of participants' voices. For each study, the reviewer entered data extracts into the form separately for children, parents and professionals.

Synthesis

Our choice of synthesis method was guided by the practical aims of this review, the "thickness" of the data reported in the primary studies, and the expertise of the team.[22 23] We applied thematic synthesis[21 24] in three stages:

I. *Line-by-line coding* alongside data extraction. Two reviewers independently coded the data extracts for themes relevant to the acceptability of the identification approach and initial response to children's exposure to IPV[25] subsequently meeting to compare and combine their codes. The first reviewer produced a final table of codes with supporting verbatim text for each participant group in each study.
II. *Developing descriptive themes.* The first reviewer grouped the codes into themes and sub-themes with accompanying verbatim to capture consistency and range of views within each participant group and across the studies.[25] Second reviewers commented on the table leading to the final version.
III. *Generating analytical themes.* The first reviewer used the constant comparison method[26] to integrate perspectives across child, parent and professional groups. The integrated stakeholder perspectives were categorised by level of agreement within and across the groups.[27] When perspectives on a theme were consistent, it was categorised as convergent. When stakeholders' views on a theme were consistent within groups, but differed between them, it was categorised as divergent. Finally, themes with wide variation, within and between the groups, were categorised as conflicting. This integration through categorisation produced interim analytical themes which were further refined in relation to the research questions. Our approach allowed us to: (i) integrate perspectives of all participant groups and generate new interpretations beyond findings from primary studies, (ii) identify gaps in evidence within and across participant groups; and (ii) establish areas of conflicting perspectives which can be targeted in future research and interventions. Throughout this stage, the first reviewer developed diagrams and tables with interim analytical themes which were refined during group discussions at three meetings with all second reviewers.

For peer review only - http://bmjopen.bmj.com/site/about/guidelines.xhtml

RESULTS

Searches

Sixteen papers reporting 11 studies were included (Figure 1, Supplementary file 3); three were reported in multiple papers. Two papers were from a study on parents' experiences with Irish child protection services.[28 29] Two papers drew on a study of police notifications to children's social services of IPV incidents where children were present/resided in the household.[30 31] Four papers were from the RESPONDS study on general practice clinicians' perspectives on child safeguarding in IPV cases.[32-34]

Critical appraisal

The quality of studies was good overall (see online Supplementary file 4), except for lack of justification for study design,[31 35-39] chosen methods[31 35-37 39-42], participant selection[31 35-38 41 42] and recruitment.[29 31 35 37 39 40 42] Only two studies described strategies for establishing neutrality.[36 43] Of 11 studies, seven were in the top tertile (scored >=15) (Fig. 2).[29 38-43]

Study characteristics

The 11 included studies are summarised in the online Supplementary file 5. Ten studies were based in high-income countries: UK,[31 34 37] US,[36 40 42] Australia,[38 43] Ireland,[28] and Canada.[41] One study was based in a middle-income country, Brazil.[35]

Voices of children were reported in two studies,[31 43] parents in eight studies[28 31 37 38 40-43] and professionals in seven studies.[31 34-37 40 41] Overall, the studies involved 42 children and young people aged 8 to 24, 220 parents (212 mothers), and 251 professionals (113 health care, 42 children's social services, and 96 mixed samples). All parents were IPV survivors.

Synthesis of research findings

I-II. Line-by-line coding and descriptive themes

Initial line-by-line coding generated 75 codes. We grouped them into 22 descriptive themes with 13 subthemes related to: (1) experiences of identifying children's exposure to IPV; (2) experiences of the initial response to children's exposure; (3) factors enabling identification and initial response to children's exposure ; (4) reasons for not identifying or disclosing children's exposure or not engaging with services; (5) psychological consequences of individuals' involvement in identification and initial

For peer review only - http://bmjopen.bmj.com/site/about/guidelines.xhtml

response to children's exposure; and (6) suggested training and resources (see online Supplementary file 6). All relevant parents' quotations were from mothers. Not all participant groups contributed equally to the descriptive themes: children's voices contributed to 10, mothers' voices to 17; professionals' perspectives were present across all 22 themes.

III. Analytical themes

Categorisation of participant views by agreement/disagreement within and across the three stakeholder groups showed converging, diverging and conflicting perspectives on satisfactory identification and responses to children's exposure to IPV. These perspectives were expressed in five interim analytical themes (see online Supplementary file 7) which were refined further, when possible resolving conflicting perspectives within and across the studies. Finally, we articulated the final analytical themes as descriptions of an ideal identification and initial response:

1. Precursors for acceptable identification and response,
2. Acceptable identification,
3. Acceptable initial response, and
4. Conflicting perspectives on engagement with children and management of safety.

Quotes from each stakeholder group supporting the final analytical themes are collated in the online Supplementary file 8.

For peer review only

For peer review only - http://bmjopen.bmj.com/site/about/guidelines.xhtml

Table 2. Analytical themes and their definitions

Final analytical themes with subthemes	Definition	Study
**1. Precursors for acceptable identification and response**		
**1.1. Satisfying and sustainable relationship**	Patients know and trust professionals with whom they develop good long-term relationships. Trusting relationships enable patients to feel safe and comfortable to discuss sensitive issues.	[28 36 38 39 41 43]
**1.2. Desired professional attitudes and skills**	When interacting with patients/clients, professionals demonstrate non-judgemental, non-threatening attitudes, show respect, actively listen, validate patient's accounts, reassure confidentiality, and provide practical help.	[28 31 36 38-41 43]
**1.3. Considering mother's readiness**	Professionals acknowledge individual mothers' readiness to disclose IPV and engage with services, work towards increasing mothers' readiness and match their approaches to the stage of mothers' readiness.	[31 36 38 41 42]
**1.4. Patient materials**	Culturally sensitive materials on IPV and children's exposure to IPV in different languages are displayed in healthcare settings.	[36 42]
**1.5. Professional training**	Professionals receive adequate training on communication with children, indicators of children's exposure to IPV, especially psychological and non-direct physical IPV, professionals' role in identifying and responding, documenting and reporting, inter-agency work.	[31 32 36 39-41]
**1.6. Professional resources**	Professionals have clear guidance on local IPV resources, what constitutes children's exposure to IPV, what is reportable, and how to document children's exposure to IPV in a way that keeps the child safe and ensures the safety and confidentiality of the mother.	[36 39 40]
**1.7. Professional supervision and support**	Professionals have skilled supervision and ongoing support for coping with psychological consequences of working with children and mothers exposed to IPV and preventing vicarious trauma.	[35 36 39 41]
**1.8. Addressing systems' barriers**	Professionals' work of identifying and responding to children's exposure to IPV fits into the organisational, local and national context of increased demands on health care and social services without commensurate resources.	[31 36 37 39]
**2. Acceptable identification**		
**2.1. Space and time**	It is ideal to give patients permission, space and time to discuss sensitive matters.	[43]

For peer review only - http://bmjopen.bmj.com/site/about/guidelines.xhtml

Final analytical themes with subthemes	Definition	Study
**2.2. Vocabulary**	It is preferable for HCPs to phrase questions about children's exposure to IPV as a "safety-at-home" matter.	[43]
**2.2. Phased approach**	When asking about children's exposure to IPV, it is ideal for HCPs to initiate the enquiry, adapt it to the context of the consultation, and use a phased approach – from presenting symptoms to general safety and well-being, then to safety at home.	[35 36 39 40 43]
**3. Acceptable initial response**		
**3.1. Shifting focus**	Professionals first focus their responses on the mother-child dyad and shift to the child if he/she is at risk of harm. Professionals need assistance with managing emotional burdens caused by the shift.	[31 36 39]
**3.2. Emotional support**	When responding to disclosure, it is ideal to provide children and parents with encouragement and emotional support.	[35 39 41]
**3.3. Education**	It is acceptable to educate mothers about the impact of IPV on children, IPV dynamics, professionals' roles and duties in responding. However, education should not jeopardise patient safety (e.g., through sending materials home where the perpetrator can find them).	[31 36 39-42]
**3.4. Signposting**	It is acceptable for professionals to give children and mothers information about local IPV services.	[31 39 41]
**4. Conflicting perspectives on engagement with children and management of safety**		
**4.1. Engaging directly with children**	Stakeholders' perspectives on the acceptability of talking directly to children exposed to IPV and seeing them alone are conflicting. Children are absent in the patient-professional communication. Recipients of care want direct engagement with children. Professionals do not see children as patients on their own and feel ill equipped for communicating with children about IPV.	[31 37 39 43]
**4.2. Management of safety**	Stakeholder preferences regarding risk assessment and safety planning are conflicting. Recipients of care are absent in the management of safety and want to be involved. Providers are not satisfied with current risk assessment and safety planning approaches and want them to change.	[31 36 37 39 41 43]

For peer review only - http://bmjopen.bmj.com/site/about/guidelines.xhtml

I. Precursors for acceptable identification and response. This theme was developed from converging perspectives on facilitators and barriers to satisfactory identification and response. It captures areas of agreement within and between stakeholder groups on the enabling processes and conditions required before children's exposure to IPV is disclosed or discussed. Patients know and trust professionals who are non-judgemental and non-threatening, have good communication skills, can ensure confidentiality, and can offer practical help.[28 31 36 38-41 43] Good communication and trusting patient-professional relationship make children and mothers feel comfortable and safe in discussing sensitive issues and to return for support, if needed.[31 43] Professionals match their approaches to the individual mother's readiness to disclose and engage with services and work with the mother towards increasing her readiness.[31 36 38 41 42] Culturally sensitive materials in different languages on IPV and children's exposure to IPV are displayed in healthcare settings to prepare patients for enquiry and provide information for those who are not ready to disclose and engage.[36 42]

Professionals receive sufficient training and guidance built upon local policies on children's exposure to IPV and inter-agency work with children's social services and IPV services. Training fits into daily practice of HCPs and SCPs and prepares them to better communicate with children. Training and guidance clarify: (i) what constitutes children's exposure to IPV, especially psychological and non-direct physical, (ii) what are professionals' roles and reporting duties, (iii) how and where to document children's exposure to IPV, and (iv) how and where to signpost mothers and children. Training and guidance increase professional awareness, skills and confidence, which lead to more satisfactory identification and responses.[31 32 36 39-41]

Providers and recipients of care receive emotional support and help with managing the emotional burden of involvement in identifying and responding to IPV. For patients/clients, such support is provided through non-judgemental responses and confidentiality (or being clear when reports must be made). For professionals, ongoing emotional support and supervision need to be arranged.[28 31 35 36 38 41-43] This helps to maintain well-being and mental health of patients and professionals and leads to increased satisfaction.

The acceptability of any work related to identification of and response to children's exposure to IPV can be undermined by systems-level constraints, such as high demand on services, lack of resources and system support, and poor inter-agency collaboration. Therefore, the approaches to identification and responses must be aligned with the capabilities of under-resourced healthcare and social care services.[31 36 37 39]

For peer review only - http://bmjopen.bmj.com/site/about/guidelines.xhtml

II. Acceptable identification This theme was developed from converging perspectives on the acceptable approaches to identification. It captures areas of agreement within and between stakeholders on when and how professionals should ask about children's exposure to IPV. The enquiry is built upon a trusting patient-professional relationship, takes place in a safe and supportive environment and is integrated into the context of the consultation or visit. Acceptable identification involves a trained and well-supported HCP who is non-judgemental and has good communication skills. HCP uses a phased approach, i.e. starts from the presenting symptoms, then moves to general safety and well-being of the child and finally asks about feeling safe at home.[35 36 39 40 43]

III. Acceptable initial responses. This theme was developed from converging perspectives on what the acceptable initial response should include and how it should be delivered. It also captures conflicting professional perspectives on the focus of their responses, reported within and between three studies and resolved though justifying that child safety is prioritized over mother's safety and confidentiality. Due to the overlap between IPV and children's exposure to IPV, most HCPs first target their initial response to the mother-child dyad.[36 39] However, their focus shifts towards the child if they are at risk of harm.[31 39] Some professionals feel conflicted when prioritising the child's needs over the adult's and require assistance with managing the emotional burden.[36] Professionals educate the mother about the impact of IPV on children, as well as the roles and duties of different providers. This helps to relieve mothers' fears and increase their readiness to engage with services.[31 36 39-42] Importantly, the approach to patient education should not jeopardise their safety (e.g., by sending materials home).[31] Mothers and children must also be given information about local IPV services.[31 39 41]

IV. IV. Conflicting perspectives on engagement with children and management of safety. This theme captures divergent and conflicting perspectives on the appropriateness of engaging directly with children and satisfactory management of safety. The variations were reported within and between four studies and remain un-resolved in our review. While most children and mothers are positive about professionals talking directly to children and addressing their individual needs, a few patients held the opposite view.[31 43] In contrast, most professionals do not feel competent in communicating directly with children and prefer to assess children's needs through a proxy adult,[34 39] while some report always engaging with children and some think they should engage more.[31 37] Furthermore, all stakeholder groups have different views on the appropriate age for talking directly to children about IPV.[34 39 43] For example, some mothers think that HCPs can talk to their children about IPV from the age of five, while some doubt the acceptability of such conversations even with adolescents.[43] Finally, patients and HCPs do not agree on the appropriate age for seeing a doctor alone. Most children prefer their mother or trusted adult to be with them at the

For peer review only - http://bmjopen.bmj.com/site/about/guidelines.xhtml

consultation and indicate that at least teenagers should be seen alone.[43] Similarly, most HCPs indicate they would not see children alone, with some suggesting that if they were to do so, they would need to be adolescents. A few HCPs suggest that they would seek a parent's and child's permission to see the child alone, but they do not elaborate on the practicalities of obtaining such permission.[39]

Next, mothers and SCPs have divergent and conflicting views on the management of child safety and both perceive current practices as unsatisfactory. First, safety is understood differently by mothers and professionals. Actions perceived by mothers as increasing child's safety (e.g., staying with perpetrator for financial reasons, not seeking help to prevent escalation of abuse) are seen by SCPs as increasing the risk for the child.[38 41] While most mothers think that involvement of children's social services increases the risk for the child through potential escalation of abuse and child removal, most SCPs believe that their involvement results in greater safety. Whereas most SCPs think that women and children are safer out of the abusive relationship and feel frustrated when women do not follow their advice to leave, women do not feel safer after leaving the perpetrator because of potential escalation of abuse, child contact with perpetrator, and lack of support post-separation. However, mothers feel intimidated by SCPs and that they must adhere to their instructions to keep children in the home and minimize involvement of children's social services.[28 38 41]

Second, stakeholders' perspectives on acceptable risk assessment and safety planning vary and diverge. Children's preferences to be asked about feeling safe at all environments are represented by one boy's voice which does not match any of the professional or parent viewpoints.[43] Mothers report not being included in safety planning with SCPs and want their views to be considered.[41] Professionals have polarised perspectives on the acceptability of the current risk assessment and safety planning with most feeling dissatisfied with the inconsistent approaches and bureaucracy involved. A few SCPs suggest engaging with mothers and children more without elaborating on how this should be done. All stakeholders report feeling conflicted about the existing management of safety, but have to comply due to the imbalance of power and system requirements.[31 36 37 39 41]

Sensitivity analysis

Exclusion of four studies scoring below 15 on the M-CASP in sensitivity analysis[31 35-37] did not change the final analytical themes.

DISCUSSION

For peer review only - http://bmjopen.bmj.com/site/about/guidelines.xhtml

Main findings

This synthesis included 11 qualitative studies with 513 providers and recipients of health care and social care. We identified enabling precursors, ideal approaches, and areas of disagreement among children, non-abusing parents and professionals regarding the acceptable identification and initial response to children's exposure to IPV. Enabling precursor processes were linked to patient/client-professional relationship building, creating a safe and supporting environment, and changing and matching responses according to individual mothers' readiness to disclose and engage with services. Enabling conditions included sufficient training and information materials and embedding the work of identifying and responding to children's exposure to IPV into the context of under-resourced services. Acceptable identification involved a phased approach to enquiry. An ideal initial response included emotional support, education about IPV and signposting to IPV services. Areas of disagreement were related to the acceptability of engaging directly with children and managing child's safety.

An important finding of this synthesis is that participants' views on many factors related to satisfactory identification and initial response to children's exposure to IPV are strikingly consistent. Our first theme suggests that recipients of care need to have a trusting relationship with a professional who demonstrates certain attitudes and skills before enquiry and identification occurs. Combined with the theme of a phased approach to enquiry, our findings support a case-finding approach to identifying IPV. Our third theme supports the acceptability of providing mothers and children with emotional support, education about IPV and signposting to local IPV services. These essential components of good clinical practice on identification and response to IPV are supported in the literature [20 44 45] and highlight the importance of safe environments and trusting relationships as conditions for recognition of and response to mothers' and children's exposure to IPV.[46] Furthermore, the acceptability of a case-finding approach is consistent with the approach outlined by WHO on identification of child maltreatment in the recent mhGAP Intervention Guide.[47] The ideal initial responses to children's exposure to IPV are in line with the WHO LIVES principles for IPV response (**L**isten, **I**nquire about needs and concerns, **V**alidate, **E**nhance safety, and **S**upport).[10]

Another notable finding is the role of the wider context. Although most of the included studies were based in high-income countries, all professionals described how their ability to identify and respond to children's exposure to IPV was heavily influenced by health and social care systems constraints. Specifically, they felt they lacked time to engage with their patients about sensitive issues; they felt burdened by constant cuts and restructuring of health care and social services; and they expressed

For peer review only - http://bmjopen.bmj.com/site/about/guidelines.xhtml

frustration about poor access to referral pathways. These systemic factors were compounded by poor communication and coordination across organisations and absence of a single referral pathway, consistent with findings in research on child protection services.[48 49] This highlights the importance of targeting interventions on identification and initial response to IPV at both individual and system levels,[50-52] with professional guidance adaptable to the changing landscape of local services.

Our findings about the emotional burden of IPV work suggest that identifying and responding to children's exposure to IPV can have negative psychological impact on both providers and recipients of care (e.g., disempowerment, compassion fatigue and vicarious trauma). The common causes of distress are patients'/clients' feelings of shame and guilt linked to the acknowledgement of IPV and disclosure, professionals' ambiguous feelings towards mothers who did not follow their advice, tensions when shifting the focus from mother-child dyad to the child, and frustration with system-level obstacles. These findings, which are consistent with previous research,[53-55] emphasize the importance of assisting both patients/clients and professionals with managing psychological symptoms and preventing vicarious trauma.[55-57] The results also supports a trauma- and violence-informed approach to care of adults and children exposed to violence.[56-59]

One of the most striking findings of this review is the gap between including children's own needs and preferences, and the lack of attention paid by professionals to children as patient/clients on their own. Although professionals recognised the importance of working with a mother-child dyad exposed to IPV, most did not perceive children as individual patients/clients and used a proxy adult to assess and respond to the child's needs. In contrast, mothers and children were in favour of HCPs talking directly to children, treating each one as an individual and addressing the child's needs directly. The invisibility of children's own views has been previously reported[60-64] and can be explained by ethical and methodological challenges of undertaking research with children,[65] rigid professional attitudes, and lack of knowledge/competence on communicating with children. However, the finding is still concerning, especially given growing recognition of children's rights[66] and agency.[67] The gap between patient and professional preferences for talking directly to children suggests that professionals need training and guidance on communicating with children about sensitive issues. With regards to children's exposure to IPV specifically, professionals need help understanding safety requirements, such as asking children in a private, confidential environment about exposure to IPV.[47]

Patients/clients and professionals held conflicting views about the acceptable management of safety. Neither patients nor professionals were satisfied with current risk assessment and safety

For peer review only - http://bmjopen.bmj.com/site/about/guidelines.xhtml

planning. Addressing the needs of mother and child involves aligning professionals' and mothers' diverging perspectives about safety and risk. While professionals may think that leaving an abusive partner is a pre-requisite for safety, this is the time when women are at greatest risk of homicide;[68] furthermore, women themselves know when it is safest to leave.[69] In our synthesis, professionals favoured targeting the mother-child dyad, but found it acceptable to switch the focus to the child when they are at risk of harm. This is consistent with literature which suggests a hierarchy of needs for children and caregivers, recognising the needs of both mother and child, but prioritising the child given children's inherent vulnerability.[70]

Strengths and limitations

We used comprehensive strategies for retrieving papers, including systematic searches of bibliographic databases, citation searching, reference checking, and emailing topic experts. Involvement of two reviewers throughout screening, data abstraction and critical appraisal reduced potential bias. Reviewers' backgrounds in different disciplines broadened and enriched data interpretation. Bringing together perspectives of children, mothers and professionals gave the recipients of care a voice equal to the providers. Our analytical themes are easily understood and can be used by practitioners and policy makers as targets for interventions on identification and response to children's exposure to IPV.

This review has several limitations. Only papers published in English were included due to limited resources. This limitation alongside the exclusion of books and conference abstracts could result in missing studies relevant to the review questions. However, our decision to focus on papers that have been peer-reviewed and potentially of better methodological quality, increases the robustness of our findings. Only two of the eleven studies reported children's voices. Although small, the sample of children covered varied ages and settings.

Future studies should explore children's own experiences of encounters with health care and social services around identification and initial responses to IPV. Children's voices will provide important information on their values and preferences. Our fourth theme of conflicting perspectives should guide future research on the acceptable approaches to talking directly to children about IPV, assessing risk and undertaking risk assessment and safety planning.

Conclusion

Healthcare and social care professionals should receive sufficient training and on-going individual and system-level support to provide acceptable identification of and initial response to children's

For peer review only - http://bmjopen.bmj.com/site/about/guidelines.xhtml

For peer review only

exposure to IPV. Ideal identification and responses should use a phased approach to enquiry and WHO LIVES principles integrated into a trauma- and violence-informed model of care.

FUNDING

This work was supported by the Public Health Agency of Canada, through funding to the VEGA Project (HLM, NW). HLM was supported by the Chedoke Health Chair in Child Psychiatry. NVL was also supported by the Avon Primary Care Research Collaborative and National Institute for Health Research (NIHR) Collaboration for Leadership in Applied Health Research and Care (CLAHRC) North Thames at Bart's Health NHS Trust. The views expressed are those of the authors and not necessarily those of the NHS, the NIHR or the Department of Health.

COMPETING INTERESTS

We have read and understood BMJ policy on declaration of interests and declare that we have no competing interests.

ACKNOWLEDGEMENTS

The authors thank Dr Lisa Arai for performing the electronic searches and for helping with title and abstract screening. The authors are grateful to Khabo Piggott and Eleanor Wright who volunteered to help with data extraction, coding and critical appraisal.

CONTRIBUTORS

HLM and NW conceived the idea. GSF, NVL, EH, ES designed the study. NVL identified additional studies, carried out title and abstract screening, full text screening, quality appraisal, data extraction, coding and synthesis. She reported the research and drafted the paper. EH, ES, GSF conducted double full text screening, data extraction, coding and quality appraisal. They contributed to the development of descriptive and analytical themes. All authors contributed to the interpretation of the data and five revisions of the paper.

DATA SHARING STATEMENT

Data extracted from each of the 16 papers are stored in Word files and are available from the corresponding author upon reasonable request. Raw syntheses of these data are stored in Excel and Word files and are available from the corresponding author upon reasonable request.

For peer review only - http://bmjopen.bmj.com/site/about/guidelines.xhtml
For peer review only

List of figures

Figure 1. Flow of studies through the review.

Figure 2. Methodological quality of the studies as assessed by the modified Critical Appraisal Skills Programme (M-CASP, score range 0-20). Studies scored >=15 are in the top tertile.

List of Supplementary files

Supplementary file 1. ENTREQ checklist (Enhancing transparency in reporting the synthesis of qualitative research.

Supplementary file 2. Search strategy.

Supplementary file 3. List of excluded papers and reasons for exclusion.

Supplementary file 4. Quality appraisal for the included studies.

Supplementary file 5. Characteristics of the 11 studies (16 papers) synthesised (in chronological order).

Supplementary file 6. Descriptive themes.

Supplementary file 7. Interim analytical themes and their definitions.

Supplementary file 8. Final analytical themes supported by raw qualitative data from included studies.

REFERENCES

1. WHO. Global and regional estimates of violence against women: prevalence and health effects of intimate partner violence and nonpartner sexual violence. Italy: WHO, 2013:51.
2. García-Moreno C, Jansen H, Ellsberg M, Heise L, Watts C. WHO multi-country study on women's health and domestic violence against women : initial results on prevalence, health outcomes and women's responses. Geneva, Switzerland: WHO, 2005:118.
3. MacMillan HL, Wathen CN. Children's exposure to intimate partner violence. Child Adolesc Psychiatr Clin N Am 2014;**23**(2):295-308, viii-ix doi: 10.1016/j.chc.2013.12.008[published Online First: Epub Date]|.
4. Radford L, Hester M. *Mothering through domestic violence*. London ; Philadelphia: Jessica Kingsley Publishers, 2006.
5. Finkelhor D, Turner HA, Shattuck A, Hamby SL. Prevalence of Childhood Exposure to Violence, Crime, and Abuse: Results From the National Survey of Children's Exposure to Violence. JAMA Pediatr 2015;**169**(8):746-54 doi: 10.1001/jamapediatrics.2015.0676[published Online First: Epub Date]|.
6. McTavish JR, MacGregor JC, Wathen CN, MacMillan HL. Children's exposure to intimate partner violence: an overview. Int Rev Psychiatry 2016:1-15 doi: 10.1080/09540261.2016.1205001[published Online First: Epub Date]|.

For peer review only - http://bmjopen.bmj.com/site/about/guidelines.xhtml

7. Government HM. Working together to safeguard children. A guide to inter-agency working to safeguard and promote the welfare of children: HM Government, 2015:109.
8. NICE. Domestic violence and abuse: How social care, health services and those they work with can respond effectively. London: NICE, 2014.
9. WHO. Responding to Intimate Partner Violence and Sexual Violence Against Women: WHO Clinical and Policy Guidelines. Geneva: WHO, 2013.
10. WHO. Health care for women subjected to intimate partner violence or sexual violence. http://www.who.int/reproductivehealth/publications/violence/vaw-clinical-handbook/en/: WHO, 2014.
11. PreVAiL. VEGA (Violence, Evidence, Guidance and Action) Project: a public health response to family violence. Secondary VEGA (Violence, Evidence, Guidance and Action) Project: a public health response to family violence. https://projectvega.ca/.
12. Green J, Thorogood N. *Qualitative methods for health research*. Third edition. ed. Los Angeles: SAGE, 2014.
13. CRD. Systematic reviews: CRD's guidance for undertaking reviews in health care. In: Dissemination CfRa, ed. Centre for Reviews and Dissemination: University of York, 2009:294.
14. Tong A, Flemming K, McInnes E, Oliver S, Craig J. Enhancing transparency in reporting the synthesis of qualitative research: ENTREQ. BMC Med Res Methodol 2012;**12**:181 doi: 10.1186/1471-2288-12-181[published Online First: Epub Date]|.
15. Booth A. Searching for qualitative research for inclusion in systematic reviews: a structured methodological review. Syst Rev 2016;**5**(74)
16. Howarth E, Moore T, Welton N, et al. IMPRoving Outcomes for children exposed to domestic ViolencE (IMPROVE): an evidence synthesis. Public Health Res 2016;**4**(10)
17. CASP. Critical Appraisal Skills Programme. CASP Qualitative Research Checklist 31.05.13. Secondary Critical Appraisal Skills Programme. CASP Qualitative Research Checklist 31.05.13 2013. http://www.casp-uk.net/checklists.
18. McTavish J, Kimber M, Devries K, et al. Mandated reporters' experiences with reporting child maltreatment: a meta-synthesis of qualitative studies. BMJ Open 2017
19. Carroll C, Booth A. Quality assessment of qualitative evidence for systematic review and synthesis: Is it meaningful, and if so, how should it be performed? Res Synth Methods 2015;**6**(2):149-54 doi: 10.1002/jrsm.1128[published Online First: Epub Date]|.
20. Feder GS, Hutson M, Ramsay J, Taket AR. Women exposed to intimate partner violence: expectations and experiences when they encounter health care professionals: a meta-analysis of qualitative studies. Arch Intern Med 2006;**166**(1):22-37 doi: 10.1001/archinte.166.1.22[published Online First: Epub Date]|.
21. Thomas J, Harden A. Methods for the thematic synthesis of qualitative research in systematic reviews. BMC Med Res Methodol 2008;**8**:45 doi: 10.1186/1471-2288-8-45[published Online First: Epub Date]|.
22. Hannes K, Lockwood C. *Synthesising qualitative research : choosing the right approach*. Chichester, West Sussex, UK: John Wiley & Sons, 2012.
23. Noyes J, Hannes K, Booth A, et al. Chapter 20: Qualitative research and Cochrane reviews. In: Higgins J, Green S, eds. Cochrane handbook for systematic reviews of interventions. Version 5.3.0 (updated October 2015): The Cochrane Collaboration, 2015.
24. Barnett-Page E, Thomas J. Methods for the synthesis of qualitative research: a critical review. BMC Med Res Methodol 2009;**9**:59 doi: 10.1186/1471-2288-9-59[published Online First: Epub Date]|.
25. Braun V, Clarke V. Using thematic analysis in psychology. Qualitative Research in Psychology 2006;**3**(2):77-101 doi: 10.1191/1478088706qp063oa[published Online First: Epub Date]|.
26. Boeije H. A purposeful approach to the constant comparative method in the analysis of qualitative interviews. Qual Quant 2002;**36**(4):391-409

For peer review only - http://bmjopen.bmj.com/site/about/guidelines.xhtml

27. Shim WS, Haight WL. Supporting battered women and their children: Perspectives of battered mothers and child welfare professionals. Child Youth Serv Rev 2006;**28**(6):620-37 doi: 10.1016/j.childyouth.2005.06.005[published Online First: Epub Date]|.
28. Buckley H, Whelan S, Carr N. 'Like waking up in a Franz Kafka novel': Service users' experiences of the child protection system when domestic violence and acrimonious separations are involved. Child Youth Serv Rev 2011;**33**(1):126-33
29. Buckley H, Whelan S, Carr N, Murphy C. Service users' perceptions of the Irish Child Protection System. The Stationery Office, Dublin: Office of the Minister for Children and Youth Affairs, 2008:96.
30. Stanley N, Miller P, Richardson Foster H. Engaging with children's and parents' perspectives on domestic violence. Child & Family Social Work 2012;**17**(2):192-201
31. Stanley N, Miller P, Richardson Foster H, Thompson G. Children and families experiencing domestic violence: Police and children's social services' responses. London: NSPCC University of Lancashire, 2010:268.
32. Drinkwater J, Stanley N, Szilassy E, Larkins C, Hester M, Feder G. Juggling confidentiality and safety: A qualitative study of how general practice clinicians document domestic violence in families with children. BJGP 2017 doi: 10.3399/bjgp17X689353[published Online First: Epub Date]|.
33. Szilassy E, Drinkwater J, Hester M, et al. Making the links between domestic violence and child safeguarding: an evidence-based pilot training for general practice. Health Soc Care Community 2016 doi: 10.1111/hsc.12401[published Online First: Epub Date]|.
34. Larkins C, Drinkwater J, Hester M, Stanley N, Szilassy E, Feder G. General practice clinicians' perspectives on involving and supporting children and adult perpetrators in families experiencing domestic violence and abuse. Family practice 2015;**32**(6):701-05 doi: 10.1093/fampra/cmv070[published Online First: Epub Date]|.
35. Angelo M, Isidoro Prado S, Cascaes Cruz Ai, Oliveira Ribeiro M. Nurses' experiences caring for child victims of domestic violence: a phenomenological analysis. Texto & Contexto Enfermagem 2013;**22**(3):585-92 8p
36. Black S, Dempsey SH, Davis MB. Practitioner-recommended policies and procedures for children exposed to domestic violence. Health Promotion Practice 2010;**11**(6):900-7
37. Clarke A, Wydall S. From 'Rights to Action': practitioners' perceptions of the needs of children experiencing domestic violence. Child & Family Social Work 2015;**20**(2):181-90 doi: 10.1111/cfs.12066[published Online First: Epub Date]|.
38. Meyer S. 'Acting in the children's best interest?': Examining victims' responses to intimate partner violence. Journal of Child and Family Studies 2011;**20**(4):436-43
39. Szilassy E, Das J, Drinkwater J, et al. Researching Education to Strengthen Primary care ON Domestic violence & Safeguarding (RESPONDS). Final Report for the Department of Health, Policy Research Programme Project Bristol: University of Bristol, 2015.
40. Davidov DM, Jack SM, Frost SS, Coben JH. Mandatory reporting in the context of home visitation programs: intimate partner violence and children's exposure to intimate partner violence. Violence Against Women 2012;**18**(5):595-610
41. Jenney A, Mishna F, Alaggia R, Scott K. Doing the right thing? (Re) Considering risk assessment and safety planning in child protection work with domestic violence cases. Child Youth Serv Rev 2014;**47**:92-101 doi: 10.1016/j.childyouth.2014.07.015[published Online First: Epub Date]|.
42. Randell KA, Bledsoe LK, Shroff PL, Pierce MC. Educational Interventions for Intimate Partner Violence Guidance From Survivors. Pediatric Emergency Care 2012;**28**(11):1190-96 doi: 10.1097/PEC.0b013e318271be7f[published Online First: Epub Date]|.
43. Morris A. Safety and resiliency at home: Voices of children from a primary care population (Unpublished PhD thesis). The University of Melbourne, 2015.

For peer review only - http://bmjopen.bmj.com/site/about/guidelines.xhtml

44. Feder G, MacMillan HL. Intimate partner violence. In: Goldman L, Schafer AI, eds. Goldman's Cecil Medicine (25th ed). Philadelphia, PA: Elsevier Saunders, 2015.
45. MacMillan HL, Fleming J, Jamieson E. Psychiatric assessment of children and adolescents. In: Goldbloom R, ed. Pediatric Clinical Skills. (4th ed). New York: Churchill Linvingstone, 2010:2062–14.
46. VEGA. Trauma- and violence-informed care. Briefing note. Secondary Trauma- and violence-informed care. Briefing note. 2016. http://projectvega.ca/wp-content/uploads/2016/10/VEGA-TVIC-Briefing-Note-2016.pdf.
47. WHO. Update of the Mental Health Gap Action Programme (mhGAP) guidelines for mental, neurological and substance use disorders. http://www.who.int/mental_health/mhgap/guideline_2015/en/: WHO, 2015:72.
48. Stanley N. *Children experiencing domestic violence: a research review*: Dartington: Research in practice, 2011.
49. Stanley N, Humphreys C. *Domestic violence and protecting children : new thinking and approaches*. London ; Philadelphia: Jessica Kingsley Publishers, 2015.
50. Turner W, Hester M, Broad J, et al. Interventions to Improve the Response of Professionals to Children Exposed to Domestic Violence and Abuse: A Systematic Review. Child Abuse Rev 2017;**26**(1):19-39 doi: 10.1002/car.2385[published Online First: Epub Date]|.
51. Zaher E, Keogh K, Ratnapalan S. Effect of domestic violence training: systematic review of randomized controlled trials. Can Fam Physician 2014;**60**(7):618-24, e340-7
52. Garcia-Moreno C, Hegarty K, d'Oliveira AF, Koziol-McLain J, Colombini M, Feder G. The health-systems response to violence against women. Lancet 2015;**385**(9977):1567-79 doi: 10.1016/S0140-6736(14)61837-7[published Online First: Epub Date]|.
53. Dmytryshyn AL, Jack SM, Ballantyne M, Wahoush O, MacMillan HL. Long-term home visiting with vulnerable young mothers: an interpretive description of the impact on public health nurses. Bmc Nurs 2015;**14**(12)
54. Conrad D, Kellar-Guenther Y. Compassion fatigue, burnout, and compassion satisfaction among Colorado child protection workers. Child Abuse Neglect 2006;**30**(10):1071-80 doi: 10.1016/j.chiabu.2006.03.009[published Online First: Epub Date]|.
55. Slattery SM, Goodman LA. Secondary traumatic stress among domestic violence advocates: workplace risk and protective factors. Violence Against Women 2009;**15**(11):1358-79 doi: 10.1177/1077801209347469[published Online First: Epub Date]|.
56. Munro E. Department for Education. The Munro review of child protection: Final Report. A child-centred system. The Stationery Office Limited, 2011:189.
57. Tehrani N. *Managing trauma in the workplace : supporting workers and organisations*. Hove, East Sussex ; New York: Routledge, 2011.
58. Phillips H, Lyon E, Fabri M, Warshaw C. Promising practices and model programs: trauma-informed approaches to working with survivors of domestic and sexual violence and other trauma http://www.nationalcenterdvtraumamh.org/publications-products/promising-practices-and-model-programs/: National Center on Domestic Violence, Trauma & Mental Health, 2015:39.
59. Ponic P, Varcoe C, Smutylo T. Trauma- (and violence-) informed approaches to supporting victims of violence: policy and practice considerations. http://www.justice.gc.ca/eng/rp-pr/cj-jp/victim/rd9-rr9/p2.html: Government of Canada, Department of Justice, 2016.
60. Hester M. Children protection and domestic violence: findings from a Rowntree/ NSPCC study. In: Hanmer J, Itzin C, Quaid S, Wigglesworth D, eds. Home thruth about domestic violence: feminist influences on policy and practice: a reader. London and New York: Routledge, 2000:96-112.
61. McGee C. *Childhood experiences of domestic violence*. London ; Philadelphia: Jessica Kingsley Publishers, 2000.

For peer review only - http://bmjopen.bmj.com/site/about/guidelines.xhtml

62. Mullender A. *Children's perspectives on domestic violence*. London ; Thousand Oaks, Calif.: SAGE, 2002.
63. Radford L, Aitken R, Miller P, Ellis J, Roberts J, Firkic A. Meeting the needs of children living with domestic violence in London. Research report. London: NSPCC and Refuge, 2011:258.
64. Overlien C. Children Exposed to Domestic Violence Conclusions from the Literature and Challenges Ahead. J Soc Work 2010;**10**(1):80-97 doi: 10.1177/1468017309350663[published Online First: Epub Date]|.
65. Cater A, Øverlien C. Children exposed to domestic violence: a discussion about research ethics and researchers' responsibilities. Nordic Social Work Research 2014

4(1):67-79
66. UN. Convention on the Rights of the Child. Treaty Series 20 November 1989; 1577. http://www.refworld.org/docid/3ae6b38f0.html (accessed 21 June 2017).
67. Mayall B. *Towards a sociology for childhood : thinking from children's lives*. Buckingham ; Philadelphia: Open University Press, 2002.
68. Garcia L, Soria C, Hurwitz EL. Homicides and intimate partner violence. Trauma, Vilence & Abuse 2007;**8**(4):370-83 doi: 10.1177/1524838007307294[published Online First: Epub Date]|.
69. Reisenhofer S, Taft A. Women's journey to safety – the transtheoretical model in clinical practice when working with women experiencing intimate partner violence: a scientific review and clinical guidance. Patient Education and Counseling 2013;**93**(3): 536-48 doi: 10.1016/j.pec.2013.08.004[published Online First: Epub Date]|.
70. Humphreys C. Problems in the system of mandatory reporting of children living with domestic violence. Journal of Family Studies 2008;**14**(2-3)

For peer review only - http://bmjopen.bmj.com/site/about/guidelines.xhtml

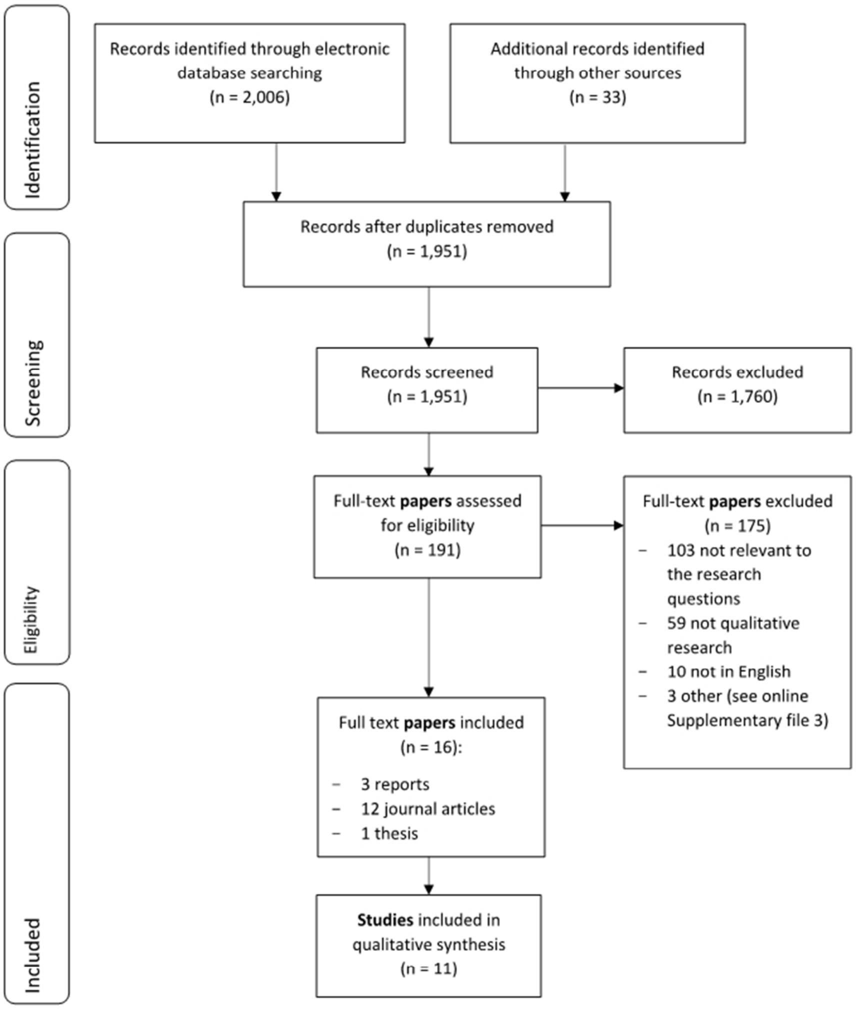

67x73mm (300 x 300 DPI)

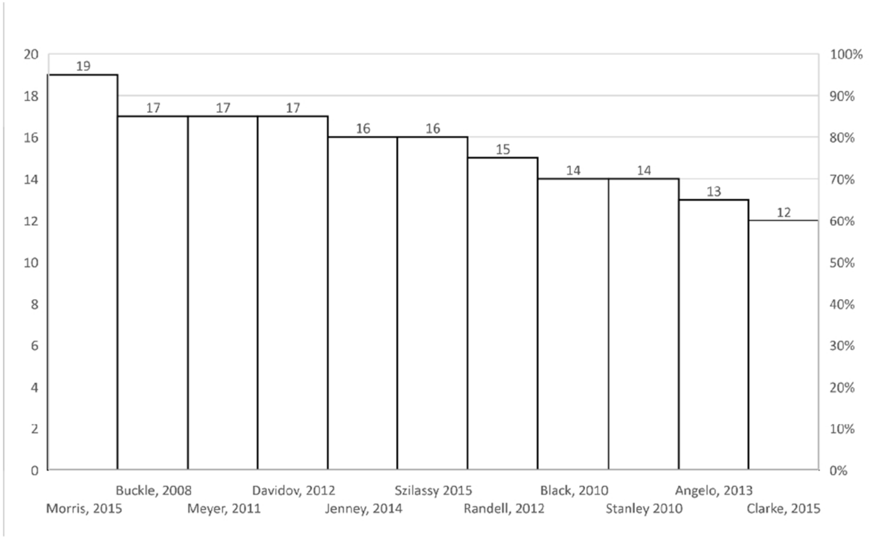

Methodological quality of the studies as assessed by the modified Critical Appraisal Skills Programme (M-CASP, score range 0-20). Studies scored >=15 are in the top tertile.

95x67mm (300 x 300 DPI)

Supplementary file 2

Search strategy

Full Boolean search strategy for MEDLINE

Date range of search 1 April 2013 to 28 April 2016.

Date of search 28 April 2016.

----

1 Child Welfare/ or Child, Preschool/ or Mother-Child Relations/ or Father-Child Relations/ or Child Behavior/ or "Child of Impaired Parents"/ or Child/ or Parent-Child Relations/ or Child Psychology/ or Child Reactive Disorders/ or Child Psychiatry/ or Adolescent Psychiatry/ or Adolescent Behavior/ or Adolescent/ or Adolescent Health Services/ or Adolescent Psychology/ or Adolescent Development/ (2612601)

2 (adolesc* or preadolesc* or pre-adolesc* or boy* or girl* or child* or infant* or preschool* or juvenil* or minors or school* or pediatri* or paediatri* or pubescen* or pre-pubescen* or puberty or student* or teen* or young or youth* or school* or high-school or "high school" or college or undergrad* or campus* or classroom*).mp. [mp=title, abstract, original title, name of substance word, subject heading word, keyword heading word, protocol supplementary concept word, rare disease supplementary concept word, unique identifier] (4168331)

3 1 or 2 (4168331)

4 Domestic Violence/ or Spouse Abuse/ or Battered Women/ (12097)

5 (abuse* adj3 (wom*n or partner or spous* or m*n or wife or wives or husband*)).tw. (5458)

6 (battered adj3 (wom*n or partner or spous* or m*n or wife or wives or husband*)).tw. (695)

7 (violen* adj3 (wom*n or partner or spous* or m*n or wife or wives or husband*)).tw. (7703)

8 (marital adj3 (violen* or abuse*)).tw. (297)

9 (famil* adj3 (violen* or abuse*)).tw. (2930)

10 domestic violen*.tw. (4517)

11 (intimate adj3 partner adj3 (violen* or abuse*)).tw. (4273)

12 (interparental adj3 (violen* or abuse*)).tw. (97)

For peer review only

13 (violen* adj2 (home*1 or household*)).tw. (206)

14 (parent* adj3 (violen* or abuse*)).tw. (1868)

15 or/4-14 (22293)

16 (expose* or exposure).mp. (927096)

17 witnes*.mp. (18418)

18 growing up.tw. (1791)

19 ((child* or adolesc*) adj3 "living with").tw. (1380)

20 16 or 17 or 18 or 19 (946981)

21 3 and 15 and 20 (2401)

22 limit 1 to ed=20130401-20160428 (284370)

23 21 and 22 (426)

For peer review only

Supplementary file 3.

List of excluded papers and reasons for exclusion

Paper is not relevant to the qualitative synthesis

1. Alaggia R, Gadalla TM, Shlonsky A, Jenney A, Daciuk J. Does differential response make a difference: examining domestic violence cases in child protection services. Child & Family Social Work. 2015;20(1):83-95.
2. Aymer SR: Adolescent males' coping responses to domestic violence: A qualitative study. Children and Youth Services Review 2008, 30(6):654-664.
3. Bennett L, Dawe D, Power J: Children's needs in shelters: mother's perceptions. Canadian Journal of Community Mental Health 1999, 18(1):57-76.
4. Bennett L: Adolescent girls' experience of witnessing marital violence: a phenomenological study. Journal of advanced nursing 1991, 16(4):431-438.
5. Berman H: Health in the aftermath of violence: a critical narrative study of children of war and children of battered women. Canadian Journal of Nursing Research 1999, 31(3):89-109.
6. Berman H: The Relevance of Narrative Research with Children Who Witness Woman Abuse. Journal of Aggression, Maltreatment & Trauma 2000, 3(1):107-125.
7. Berman HA: Growing up amid violence: a critical narrative analysis of children of war and children of battered women. WAYNE STATE UNIVERSITY; 1996.
8. Berman HA: Growing up amid violence: A critical narrative analysis of children of war and children of battered women. Dissertation Abstracts International: Section B: The Sciences and Engineering 1997, 57(12-B):7447.
9. Bertram C, Hall J, Fine M, Weis L: Where the girls (and women) are. American Journal of Community Psychology 2000, 28(5):731-755.
10. Bradbury-Jones C, Taylor J: Domestic abuse as a transgressive practice: understanding nurses' responses through the lens of abjection. Nursing Philosophy 2013, 14(4):295-304 210p.
11. Brewer AKJ: Designing treatment for children exposed to domestic violence: An exploratory study. Dissertation Abstracts International: Section B: The Sciences and Engineering 2009, 69(7-B):4410.
12. Buckley H, Holt S, Whelan S: Listen to me! Children's experiences of domestic violence. Child Abuse Review 2007, 16(5):296-310.
13. Bunce DA: Children living in a battered women's shelter: Attitudes, beliefs, behaviors, and contextual variables. Dissertation Abstracts International: Section B: The Sciences and Engineering 2000, 60(8-B):4205.
14. Byrne D, Taylor B: Children at risk domestic violence and their educational attainment: Perspectives of education welfare officiers, social workers and teachers. Child Care in Practice 2007, 13(3):185-201.
15. Byrskog U, Essen B, Olsson P, Klingberg-Allvin M: 'Moving on' Violence, wellbeing and questions about violence in antenatal care encounters. A qualitative study with Somali-born refugees in Sweden. Midwifery 2016, 40:10-17.
16. Byrskog U, Olsson P, Essen B, Allvin MK: Being a bridge: Swedish antenatal care midwives' encounters with Somali-born women and questions of violence; a qualitative study. Bmc Pregnancy and Childbirth 2015, 15.
17. Campbell JE: Supporting mother-infant relationships affected by intimate partner violence in Prince Edward Island: A pilot project.

18. Carlos DM, Carvalho Ferriani MdG, Iossi Silva MA, de Souza Teixeira Roque EM, Vendruscolo TS: Institutional shelter to protect adolescent victims of domestic violence: theory or practice?1. Revista Latino-Americana de Enfermagem (RLAE) 2013, 21(2):579-585 577p.
19. Chanmugam A: Perspectives on US domestic violence emergency shelters: What do young adolescent residents and their mothers say? Child Care in Practice 2011, 17(4):393-415.
20. DeVoe ER, Smith EL: Don't take my kids: Barriers to service delivery for battered mothers and their young children. Journal of Emotional Abuse 2003, 3(3-4):277-294.
21. DeVoe ER, Smith EL: The impact of domestic violence on urban preschool children: Battered mothers' perspectives. Journal of Interpersonal Violence 2002, 17(10):1075-1101.
22. Douglas H, Walsh T: Mothers, domestic violence, and child protection. Violence Against Women 2010, 16(5):489-508.
23. Dryden C, Doherty K, Nicolson P: Accounting for the hero: a critical psycho-discursive approach to children's experience of domestic violence and the construction of masculinities. British Journal of Social Psychology 2010, 49(Pt 1):189-205.
24. Eggert J: A qualitative analysis of mother-preschooler dyads exposed to trauma participating in child-parent psychotherapy. Dissertation Abstracts International: Section B: The Sciences and Engineering 2006, 66(12-B):6918.
25. Eisbart L: Mental health services for women and children residing in domestic violence shelters: A multicultural perspective. Dissertation Abstracts International: Section B: The Sciences and Engineering 2011, 71(8-B):5119.
26. Engnes K, Lidén E, Lundgren I: Women's experiences of important others in a pregnancy dominated by intimate partner violence. Scandinavian Journal of Caring Sciences 2013, 27(3):643-650 648p.
27. Ericksen JR, Henderson AD: Witnessing family violence - the children’s experience. Journal of advanced nursing 1992, 17(10):1200-1209.
28. Featherstone B, Fraser C: Working with fathers around domestic violence: Contemporary debates. Child Abuse Review 2012, 21(4):255-263.
29. Georgsson A, Almqvist K, Broberg AG: Naming the unmentionable: How children exposed to intimate partner violence articulate their experiences. Journal of Family Violence 2011, 26(2):117-129.
30. Gibson SM: Producing scripts: An ethnomethodological study of intimate partner violence child witness therapy. Dissertation Abstracts International Section A: Humanities and Social Sciences 2009, 69(10-A):4141.
31. Goldblatt H: Strategies of coping among adolescents experiencing interparental violence. Journal of Interpersonal Violence 2003, 18(5):532-552.
32. Gorin S, Jobe A: Young People Who Have Been Maltreated: Different Needs—Different Responses? British Journal of Social Work 2012, 43(7):1330-1346 1317p.
33. Grauwiler P: Voices of women: Perspectives on decision-making and the management of partner violence. Children and Youth Services Review 2008, 30(3):311-322.
34. Gray B: Working with families in tower hamlets: An evaluation of the Family Welfare Association's Gamily Support Services. Health & Social Care in the Community 2002, 10(2):112-122.
35. Hague G, Malos E, Mullender A, Kelly L, Imam U: Children, coping strategies and domestic violence. Community Practitioner 2002, 75(5):180-183.
36. Haight WL, Shim WS, Linn LM, Swinford L: Mothers' strategies for protecting children from batterers: the perspectives of battered women involved in child protective services. Child Welfare 2007, 86(4):41-62.

For peer review only

37. Hardesty JL, Campbell JC, McFarlane JM, Lewandowski LA: How children and their caregivers adjust after intimate partner femicide. Journal of Family Issues 2008, 29(1):100-124.
38. Henderson AD: Abused women's perceptions of their children's experiences. Canada's Mental Health 1993, 41(1):7-11.
39. Hillman LJ: Battered Women and Their Children: An Exploratory Study of the Relationships between Mothers Who Have Fled Abusive Relationships and the Children Who Have Witnessed Their Abuse.
40. Hilton ZN: Battered Women's Concerns about Their Children Witnessing Wife Assault. In.; 1992: 77-86.
41. Hooker L, Small R, Humphreys C, Hegarty K, Taft A. Applying normalization process theory to understand implementation of a family violence screening and care model in maternal and child health nursing practice: a mixed method process evaluation of a randomised controlled trial. Implement Sci. 2015;10:39.
42. Hooker L, Small R, Taft A. Understanding sustained domestic violence identification in maternal and child health nurse care: process evaluation from a 2-year follow-up of the MOVE trial. J Adv Nurs. 2016;72(3):533-44.
43. Horton E, Murray CE, Garr B, Notestine L, Flasch P, Johnson CH: Provider perceptions of safety planning with children impacted by intimate partner violence. Children and Youth Services Review 2014, 42:67-73.
44. Houghton C. Young People's Perspectives on Participatory Ethics: Agency, Power and Impact in Domestic Abuse Research and Policy-Making. Child Abuse Review. 2015;24(4):235-48.
45. Hughes J, Chau S, Poff DC: "They're not my favourite people": What mothers who have experienced intimate partner violence say about involvement in the child protection system. Children and Youth Services Review 2011, 33(7):1084-1089.
46. Hughes J, Chau S. Children's best interests and intimate partner violence in the Canadian family law and child protection systems. Crit Soc Policy. 2012;32(4):677-95.
47. Hughes J, Chau S: Children's best interests and intimate partner violence in the Canadian family law and child protection systems. Critical Social Policy 2012, 32(4):677-695.
48. Hughes J, Chau S: Making complex decisions: Child protection workers' practices and interventions with families experiencing intimate partner violence. Children and Youth Services Review 2013, 35(4):611-617.
49. Hultmann O, Möller J, Ormhaug S, Broberg A. Asking Routinely About Intimate Partner Violence in a Child and Adolescent Psychiatric Clinic: A Qualitative Study. Journal of Family Violence. 2014;29(1):67-78 12p.
50. Humphreys C, Mullender A, Thiara R, Skamballis A: 'Talking to My Mum': Developing Communication Between Mothers and Children in the Aftermath of Domestic Violence. Journal of Social Work 2006, 6(1):53-63.
51. Humphreys C, Thiara RK, Skamballis A: Readiness to change: Mother-child relationship and domestic violence intervention. British Journal of Social Work 2011, 41(1):166-184.
52. Humphreys J: The work of worrying: battered women and their children. Scholarly Inquiry for Nursing Practice 1995, 9(2):127-145.
53. Hutchings JB: A Qualitative Study of Adolescents Growing Up in Domestic Violence Homes.
54. Jack SM, Ford-Gilboe M, Wathen CND, Danielle M.; , McNaughton DB, Coben JH, Olds DL, Macmillan HL, Research TNI: Development of a nurse home visitation intervention for intimate partner violence. BMC Health Services Research 2012, 12:50.
55. Joest KS: Multiple Contexts of Violence in the Lives of Adolescent Females Who Have Been Exposed to Domestic Violence.

For peer review only

56. Joseph S, Govender K, Bhagwanjee A: "I can't see him hit her again, I just want to run away. . .hide and block my ears": A phenomenological analysis of a sample of children's coping responses to exposure to domestic violence. Journal of Emotional Abuse 2006, 6(4):23-45.
57. Katz E: Recovery-Promoters: Ways in which Children and Mothers Support One Another's Recoveries from Domestic Violence. British Journal of Social Work 2015, 45:153-169.
58. Kearney JA, Cushing E: A multi-modal pilot intervention with violence-exposed mothers in a child psychiatric trauma-focused treatment program. Issues in Mental Health Nursing 2012, 33(8):544-552.
59. Kearney JA: Women and children exposed to domestic violence: themes in maternal interviews about their children's psychiatric diagnoses. Issues in Mental Health Nursing 2010, 31(2):74-81.
60. Kubeka AM: Exposure to violence at home: a qualitative exploration of experiences and perceptions of black adolescents in South Africa. South African Review of Sociology 2008, 39(2):282-300.
61. Lemmey D, McFarlane J, Willson P, Malecha A: Intimate partner violence. Mothers' perspectives of effects on their children. MCN, American Journal of Maternal Child Nursing 2001, 26(2):98-103.
62. Leppakoski T, Paavilainen E, Astedt-Kurki P: Experiences of emergency care by the women exposed to acute physical intimate partner violence from the Finnish perspective. International emergency nursing 2011, 19(1):27-36.
63. Letourneau N, Morris CY, Stewart M, Hughes J, Critchley KA, Secco L: Social Support Needs Identified by Mothers Affected by Intimate Partner Violence. Journal of Interpersonal Violence 2013, 28(14):2873-2893.
64. Letourneau N, Young C, Secco L, Stewart M, Hughes J, Critchley K: Supporting mothering: service providers' perspectives of mothers and young children affected by intimate partner violence. Res Nurs Health 2011, 34(3):192-203.
65. Lisboa C, Koller SH, Ribas FF, Bitencourt K, Oliveira L, Porciuncula LP, De Marchi RB: Coping Strategies of Domestic Violence Victimized and Non Victimized Children. Psicologia: Reflexao e Critica 2002, 15(2):345-362.
66. Lorelle S, Grothaus T: Homeless children and their families' perspectives of agency services. Community Mental Health Journal 2015, 51(7):800-808.
67. Lucas MB: Sibling support as a protective process for children exposed to domestic violence. Dissertation Abstracts International: Section B: The Sciences and Engineering 2002, 62(12-B):5970.
68. McInnes E: The impact of violence on mothers' and children's needs during and after parental separation. Early Child Development and Care 2004, 174(4):357-368.
69. Mullender A, Debbonaire T, Hague G, Kelly L, Malos E: Working with children in women's refuges. Child & Family Social Work 1998, 3(2):87-98.
70. Munford R, Sanders J: Negotiating and constructing identity: social work with young people who experience adversity. British Journal of Social Work 2015, 45(5):1564-1580.
71. Munford R: Understanding service engagement: young people’s experience of service use. Journal of Social Work 2016, 16(3):283-302.
72. Overlien C, Hyden M: Children's actions when experiencing domestic violence. Childhood: A Global Journal of Child Research 2009, 16(4):479-496.
73. Overlien C: Abused women with children or children of abused women? A study of conflicting perspectives at women's refuges in Norway. Child & Family Social Work 2011, 16(1):71-80.
74. Overlien C: Narrating the good life-Children in shelters for abused women talk about the future. Qualitative Social Work: Research and Practice 2012, 11(5):470-485.

For peer review only

75. Overlien C: The children of patriarchal terrorism. Journal of Family Violence 2013, 28(3): 277-287.
76. Overlien C: Women's refuges as intervention arenas for children who experience domestic violence. Child Care in Practice 2011, 17(4):375-391.
77. Paris PR: The development and field testing of a multimodal intervention program for families involved in domestic violence. Dissertation Abstracts International: Section B: The Sciences and Engineering 1999, 60(2-B):0574.
78. Peled E, Edleson JL: Barriers to children's domestic violence counseling: A qualitative study. Families in Society 1999, 80(6):578-586.
79. Peled E, Edleson JL: Multiple perspectives on groupwork with children of battered women. Violence & Victims 1992, 7(4):327-346.
80. Peled E: The experience of living with violence for preadolescent children of battered women. Youth & Society 1998, 29(4):395-430.
81. Phillips B, Phillips DA: Learning from youth exposed to domestic violence: decentering DV and the primacy of gender stereotypes. Violence Against Women 2010, 16(3):291-312.
82. Pitter CP: Midwives' knowledge and attitudes when encountering Gender-Based Violence in their practice at a maternity-hospital in Kingston, Jamaica. International Journal of Qualitative Studies on Health and Well-Being 2016, 10.
83. Prosman G-J, Lo Fo Wong SH, Lagro-Janssen ALM: Why abused women do not seek professional help: a qualitative study. Scandinavian Journal of Caring Sciences 2014, 28(1):3-11 19p.
84. Ramachandran DV, Covarrubias L, Watson C, Decker MR: How You Screen is as Important as Whether You Screen: A Qualitative Analysis of Violence Screening Practices in Reproductive Health Clinics. Journal of Community Health 2013, 38(5):856-863.
85. Randell KA, Bledsoe LK, Shroff PL, Pierce MC: Mothers' Motivations for Intimate Partner Violence Help-Seeking. Journal of Family Violence 2012, 27(1):55-62.
86. Remington CM: An evaluation of interventions, codes and public policy for children who have witnessed family violence. Dissertation Abstracts International: Section B: The Sciences and Engineering 2000, 60(9-B):4905.
87. Rhodes KV, Cerulli C, Dichter ME, Kothari CL, Barg FK: "I didn't want to put them through that": The influence of children on victim decision-making in intimate partner violence cases. Journal of Family Violence 2010, 25(5):485-493.
88. Santos da Silva MR, Silva PAd, Luz GdS, Schek G, da Rosa Silveira Nunes MdP: FAMILY NEEDS AND PROFESSIONAL PRACTICES IN INTRAFAMILY DOMESTIC VIOLENCE. Ciencia, Cuidado e Saude 2014, 13(2):335-342 338p.
89. Sopczyk DL: The domestic violence experience of children: An ethnographic study. Dissertation Abstracts International Section A: Humanities and Social Sciences 2007, 68(6-A):2660.
90. Stanley N, Humphreys C. Multi-agency risk assessment and management for children and families experiencing domestic violence. Children and Youth Services Review. 2014;47:78-85.
91. Stephens DL: Battered women's views of their children. Journal of Interpersonal Violence 1999, 14(7):731-746.
92. Thompson E: The evolution of a children's domestic violence counseling group: Stages and processes. Journal for Specialists in Group Work 2011, 36(3):178-201.
93. Thompson EH: Child-Witnesses of Domestic Violence: The Evolution of a Counseling Group. 2009.
94. Tsantefski M, Humphreys C, Jackson AC. Infant risk and safety in the context of maternal substance use. Children and Youth Services Review. 2014;47:10-7.

For peer review only

95. Vaise MJ: Teaching resilience skills to children who have been exposed to domestic violence: An exploratory qualitative study. Dissertation Abstracts International: Section B: The Sciences and Engineering 2001, 61(10-B):5584.
96. van den Bosse S, McGinn MA: Child welfare professionals' experiences of childhood exposure to domestic violence. Child Welfare 2009, 88(6):47-63.
97. van der Wath A, van Wyk N, Janse van Rensburg E: Emergency nurses' experiences of caring for survivors of intimate partner violence. Journal of Advanced Nursing 2013, 69(10):2242-2252 2211p.
98. Vendruscolo TS, Ferriani MdGC, Silva MAI: Public care policies for child and adolescent victims of domestic violence. Revista Latino-Americana de Enfermagem 2007, 15 Spec No:812-819.
99. Weinehall KM: Growing Up in the Proximity of Violence: Teenagers' Stories of Violence in the Home. 1998.
100. Wiklund M, Malmgren-Olsson E-B, Bengs C, Ohman A: "He messed me up": Swedish adolescent girls' experiences of gender-related partner violence and its consequences over time. Violence Against Women 2010, 16(2):207-232.
101. Willis D, Hawkins JW, Pearce CW, Phalen J, Keet M, Singer C: Children who witness violence: what services do they need to heal? Issues in Mental Health Nursing 2010, 31(9):552-560.
102. Wood BL: Women's perceptions of their children's experiences in domestic violence. Dissertation Abstracts International: Section B: The Sciences and Engineering 1999, 60(6-B):2595.
103. Zannettino L, McLaren H. Domestic violence and child protection: towards a collaborative approach across the two service sectors. Child & Family Social Work. 2014;19(4):421-31.

Not qualitative research

1. Alzahrani TA, Abaalkhail BA, Ramadan IK: Prevalence of intimate partner violence and its associated risk factors among Saudi female patients attending the primary healthcare centers in Western Saudi Arabia. Saudi Medical Journal 2016, 37(1):96-99.
2. Ambuel B, Hamberger L, Guse C, Melzer-Lange M, Phelan M, Kistner A: Healthcare Can Change from Within: Sustained Improvement in the Healthcare Response to Intimate Partner Violence. Journal of Family Violence 2013, 28(8):833-847 815p.
3. Apostólico MR, Hino P, Egry EY: Possibilities for addressing child abuse in systematized nursing consultations. Revista da Escola de Enfermagem da USP 2013, 47(2):320-327 328p.
4. Bagshaw D, Chung D: The needs of children who witness domestic violence: a South Australian study. Children Australia 2001, 26(3):9-17.
5. Bair-Merritt M, Zuckerman B, Augustyn M, Cronholm PF: Silent Victims - An Epidemic of Childhood Exposure to Domestic Violence. New England Journal of Medicine 2013, 369(18):1673-1675.
6. Baker L, Cunningham A: Inter-parental violence: The pre-schooler's perspective and the educator's role. Early Childhood Education Journal 2009, 37(3):199-207.
7. Bazargan-Hejazi S, Kim E, Lin J, Ahmadi A, Khamesi MT, Teruya S: Risk factors associated with different types of intimate partner violence (ipv): an emergency department study. Journal of Emergency Medicine 2014, 47(6):710-719.
8. Campbell AM, Hibbard R: More than Words The Emotional Maltreatment of Children. Pediatric Clinics of North America 2014, 61(5): 959–970
9. Campbell AM, Thompson SL: The emotional maltreatment of children in domestically violent homes: Identifying gaps in education and addressing common misconceptions. Child Abuse & Neglect 2015, 48:39-49.

10. Campbell KA, Thomas AM, Cook LJ, Keenan HT: Resolution of intimate partner violence and child behavior problems after investigation for suspected child maltreatment. JAMA pediatrics 2013, 167(3):236-242.
11. Capucci R, Paganelli C, Carboni S, Cappadona R, Roberto M, Rinaldi G: Characteristics of Gender-Based Violence Determined from Emergency Room Visits. Violence and Gender 2015, 2(2):129-133.
12. Conners-Burrow NA, et a: Trauma-informed care training in a child welfare system: moving it to the front line. Children and Youth Services Review 2013, 35(11):1830-1835.
13. Coulter ML, Mercado-Crespo MC: Co-Occurrence of Intimate Partner Violence and Child Maltreatment: Service Providers' Perceptions. Journal of Family Violence 2015, 30(2):255-262.
14. Cruz M, Bair-Merrit MH: Screening and intervention for intimate partner violence. Contemporary Pediatrics 2013, 30(5):12-25 14p.
15. Cruz M, Cruz PB, Weirich C, McGorty R, McColgan MD: Referral patterns and service utilization in a pediatric hospital-wide intimate partner violence program. Child Abuse & Neglect 2013, 37(8):511-519.
16. Davidov DM, Jack SM: Nurse home visitors' perceived awareness of mandatory reporting requirements: pregnant women's and children's exposure to intimate partner violence. Journal of Advanced Nursing 2014, 70(8):1770-1779 1710p.
17. Diderich HM, Dechesne M, Fekkes M, Verkerk PH, Pannebakker FD, Velderman MK, Sorensen PJG, Buitendijk SE, Oudesluys-Murphy AM: Facilitators and barriers to the successful implementation of a protocol to detect child abuse based on parental characteristics. Child Abuse & Neglect 2014, 38(11):1822-1831.
18. Diderich HM, Fekkes M, Verkerk PH, Pannebakker FD, Velderman MK, Sorensen PJG, Baeten P, Oudesluys-Murphy AM: A new protocol for screening adults presenting with their own medical problems at the Emergency Department to identify children at high risk for maltreatment. Child Abuse & Neglect 2013, 37(12):1122-1131.
19. El-Radhi AS: Safeguarding the welfare of children: what is the nurse's role? British Journal of Nursing 2015, 24(15):769-773 765p.
20. Emerson D, Magnuson D: Child Protection Intervention in Domestic Violence: An Illustrative Case Narrative. Child & Youth Services 2013, 34(3):236-249 214p.
21. Ferrell NJ, Melton B, Banu S, Coverdale J, Valdez MR: The Development and Evaluation of a Trauma Curriculum for Psychiatry Residents. Academic Psychiatry 2014, 38(5):611-614.
22. Finklestein M, et a: Posttraumatic stress disorder and vicarious trauma in mental health professionals. Health and Social Work 2015, 40(2):e25-e31.
23. Frey-Angel J: Treating children of violent families: A sibling group approach. Social Work with Groups: A Journal of Community and Clinical Practice 1989, 12(1):95-107.
24. Green BL, Saunders PA, Power E, Dass-Brailsford P, Schelbert KB, Giller E, Wissow L, Hurtado-De-Mendoza A, Mete M: Tlioauma-Informed Medical Care: CME Communication Training for Primary Care Providers. Family Medicine 2015, 47(1):7-14.
25. Groves BM, Van Horn P, Lieberman AF: Deciding on fathers' involvement in their children's treatment after domestic violence. Edleson, Jeffrey L [Ed] 2007:65-84.
26. Hoytema van Konijnenburg EMM, Diderich HM, Teeuw AH, Klein Velderman M, Oudesluys-Murphy AM, van der Lee JH, Biezeveld MH, Brilleslijper-Kater SN, Edelenbos E, Flapper BC et al: Comparing policies for children of parents attending hospital emergency departments after intimate partner violence, substance abuse or suicide attempt. Child Abuse and Neglect 53 (pp 81-94), 2016, Date of Publication:March 01.

For peer review only

27. Jaffe P, Wilson SK, Wolfe D: Specific assessment and intervention strategies for children exposed to wife battering: Preliminary empirical investigations. Canadian Journal of Community Mental Health 1988, 7(2):157-163.
28. Jenny C, Crawford-Jakubiak JE, Comm Child Abuse N: The Evaluation of Children in the Primary Care Setting When Sexual Abuse Is Suspected. Pediatrics 2013, 132(2):E558-E567.
29. Joseph B, Khalil M, Zangbar B, Kulvatunyou N, Orouji T, Pandit V, O'Keeffe T, Tang A, Gries L, Friese RS et al: Prevalence of Domestic Violence Among Trauma Patients. Jama Surgery 2015, 150(12):1177-1183.
30. Kiely M, Gantz M, El-Khorazaty M, El-Mohandes A: Sequential screening for psychosocial and behavioural risk during pregnancy in a population of urban African Americans. BJOG: An International Journal of Obstetrics & Gynaecology 2013, 120(11):1395-1402 1398p.
31. Kodner C, Wetherton A: Diagnosis and management of physical abuse in children. American Family Physician 2013, 88(10):669-675 667p.
32. Kot S, Tyndall-Lind A: Intensive Play Therapy With Child Witnesses of Domestic Violence. Reddy, Linda A [Ed] 2005:31-49.
33. Kramer TL, et a: A statewide introduction of trauma-informed care in a child welfare system. Children and Youth Services Review 2013, 35(1):19-24.
34. Leppäkoski T, Paavilainen E: Interventions for women exposed to acute intimate partner violence: emergency professionals' perspective. Journal of Clinical Nursing 2013, 22(15/16):2273-2285 2213p.
35. Lisboa C, Koller SH, Ribas FF, Bitencourt K, Oliveira L, Porciuncula LP, De Marchi RB: Coping Strategies of Domestic Violence Victimized and Non Victimized Children. Psicologia: Reflexao e Critica 2002, 15(2):345-362.
36. Lopez-Bushnell K: New Mexico Nurses and Child Abuse Prevention. New Mexico Nurse 2016, 61(1):1-4 2p.
37. McCall-Hosenfeld JS, Winter M, Heeren T, Liebschutz JM: The association of interpersonal trauma with somatic symptom severity in a primary care population with chronic pain: Exploring the role of gender and the mental health sequelae of trauma. Journal of Psychosomatic Research 2014, 77(3):196-204.
38. McDowell BM: Ask the expert - Caring for child victims: Countering the effects of domestic violence. Journal for Specialists in Pediatric Nursing 2006, 11(2):129-132.
39. Mejdoubi J, van den Heijkant S, van Leerdam FJM, Heymans MW, Hirasing RA, Crijnen AAM: Effect of Nurse Home Visits vs. Usual Care on Reducing Intimate Partner Violence in Young High-Risk Pregnant Women: A Randomized Controlled Trial. Plos One 2013, 8(10).
40. Melchiorre R, Vis J-A: Engagement strategies and change: an intentional practice response for the child welfare worker in cases of domestic violence. Child & Family Social Work 2013, 18(4):487-495 489p.
41. Menashe A, Possick C, Buchbinder E: Between the maternal and the professional: the impact of being a child welfare officer on motherhood. Child and Family Social Work 2014, 19(4):391-400.
42. Minze LC, McDonald R, Rosentraub EL, Jouriles EN: Making sense of family conflict: intimate partner violence and preschoolers' externalizing problems. Journal of Family Psychology 2010, 24(1):5-11.
43. Moyer VA: Primary care interventions to prevent child maltreatment: U.S. Preventive Services Task Force recommendation statement. Annals of Internal Medicine 2013, 159(4):289-295 287p.
44. Moyer VA: Screening for intimate partner violence and abuse of elderly and vulnerable adults: u.s. Preventive services task force recommendation statement. Annals of Internal Medicine 2013, 158(6):478-486 479p.
45. Mullender A: Children living with domestic violence. Adoption and fostering 1996, 20(1):8-15.

For peer review only

46. O'Doherty L, Hegarty K, Ramsay J, Davidson LL, Feder G, Taft A: Screening women for intimate partner violence in healthcare settings. Cochrane Database of Systematic Reviews 2015(7).
47. Paavilainen E, Lepisto S, Flinck A: Ethical issues in family violence research in healthcare settings. Nursing Ethics 2014, 21(1):43-52.
48. Parade SH: Inter-parental violence in early childhood: Identification and intervention with exposed children. Brown University Child & Adolescent Behavior Letter 2013, 29(10):1-6 6p.
49. Peled E, Edleson JL: Predicting children's domestic violence service participation and completion. Research on Social Work Practice 1998, 8(6):698-712.
50. Prosman GJ, Wong S, Lagro-Janssen ALM: Support by trained mentor mothers for abused women: a promising intervention in primary care. Family Practice 2014, 31(1):71-80.
51. Riley CL, Sarani B, Sullivan JA, Upperman JS, Kane-Gill SL, Bailey H: Critical Violent Injury in the United States: A Review and Call to Action. Critical Care Medicine 2015, 43(11):2460-2467 2468p.
52. Roseby V, Johnston JR: Clinical interventions with latency-age children of high conflict and violence. American Journal of Orthopsychiatry 1995, 65(1):48-59.
53. Scribano P, Stevens J, Kaizar E: The Effects of Intimate Partner Violence Before, During, and After Pregnancy in Nurse Visited First Time Mothers. Maternal & Child Health Journal 2013, 17(2):307-318 312p.
54. Taft A, O'Doherty L, Hegarty K, Ramsay J, Davidson L, Feder G: Screening women for intimate partner violence in healthcare settings. Cochrane Database of Systematic Reviews 2015 Jul 22;(7):CD007007. doi: 10.1002/14651858.CD007007.pub3.
55. Taft AJ, Hooker L, Humphreys C, Hegarty K, Walter R, Adams C, Agius P, Small R: Maternal and child health nurse screening and care for mothers experiencing domestic violence (MOVE): a cluster randomised trial. Bmc Medicine 2015, 13.
56. van Dalen A, Glasserman M: My father, Frankenstein: A child's view of battering parents. Journal of the American Academy of Child & Adolescent Psychiatry 1997, 36(7):1005-1007.
57. van Konijnenburg E, Sieswerda-Hoogendoorn T, Brilleslijper-Kater SN, van der Lee JH, Teeuw AH: New hospital-based policy for children whose parents present at the ER due to domestic violence, substance abuse and/or a suicide attempt. European Journal of Pediatrics 2013, 172(2):207-214.
58. Vogus TJ, Cull MJ, Hengelbrok NE, Modell SJ, Epstein RA. Assessing safety culture in child welfare: Evidence from Tennessee. Children and Youth Services Review. 2016;65:94-103.
59. Weaver TL, Gilbert L, El-Bassel N, Resnick HS, Noursi S: Identifying and Intervening with Substance-Using Women Exposed to Intimate Partner Violence: Phenomenology, Comorbidities, and Integrated Approaches Within Primary Care and Other Agency Settings. Journal of Women's Health (15409996) 2015, 24(1):51-56 56p.

Full text not in English

1. Andrade EM, Nakamura E, de Paula CS, do Nascimento, R.; Bordin IA, Martin D: Health Professionals' Views About Domestic Violence Against Children and Teenagers: a qualitative study. Saude e Sociedade 2011, 20(1):147-155. [Article in Portuguese]
2. Cheseaux JJ, Marwood AD, Glassey NR: [Children's exposure to domestic violence. A multidisciplinary model of detection, assessment and management]. Rev Med Suisse 2013, 9(374):398-401. [Article in French]
3. da Silva AL, Vogel C, Virgilio MS: [Significance of care for children and adolescents victims of domestic violence]. Revista Brasileira de Enfermagem 2001, 54(1):48-62 [Article in Portuguese]

For peer review only

4. Gabatz RIB, Padoin SMdM, Neves ET, Terra MG: [Factors associated with institutionalization: perspectives for children who suffered domestic violence]. Revista Gaucha de Enfermagem 2010, 31(4):670-677. [Article in Portuguese]
5. Hoytema van Konijnenburg EM, van der Lee JH, Brilleslijper-Kater SN, van Leerdam FJ, Teeuw AH: [Domestic violence in the accident and emergency department: don't forget the children]. Ned Tijdschr Geneeskd 2014, 158:A7120. [Article in Dutch]
6. Kamo T: [Rebuilding of the nurturing relationship and parent-child interaction therapy of mothers and children affected by domestic violence]. Seishin Shinkeigaku Zasshi - Psychiatria et Neurologia Japonica 2010, 112(9):885-889. [Article in Japanese]
7. Keiko F: Midwifery care for victims of domestic violence that encourages recovery during the perinatal period. Journal of Japan Academy of Midwifery 2013, 27(2):247-256 210p. [Article in Japanese]
8. Kratzsch W, Menrath I, Steffens M: Impact of intimate partner violence on women and their children and the role of the pediatric. Padiatrische Praxis 2015, 83(4):539-550. [Article in German]
9. Lavergne C, Helle S, Malo C: Exposure to domestic violence: Profile of children reported to child welfare and response to families needs. Revue de Psychoeducation 2015, 44(2):245-267. [Article in French]
10. Olesen T, Nielsen AM: [The coordination group for children exposed to violence and their families in the County of Copenhagen. Experiences after 2 years work]. Ugeskrift for Laeger 1985, 147(16):1355-1359. [Article in Danish]

Other

1. Apostolico MR, Egry EY: Development of an e-learning for primary health care professionals to coping child domestic violence from the perspective of the professional competencies model. In: Iceri2014: 7th International Conference of Education, Research and Innovation. edn. Edited by Chova LG, Martinez AL, Torres IC; 2014: 5286-5291. – Conference Proceedings
2. Dodd J: Domestic violence and abuse; a psychosomatic issue. Archives of Women's Mental Health 2015, Conference:International Marce Society for Perinatal Mental Health Biennial Scientific Conference 2014 Swansea United Kingdom. Conference Start: 20140910 Conference End: 20140912. Conference Publication: (var.pagings). 20140918 (20140912) (pp 20140375). – Conference proceedings.
3. McKie L: Children and domestic violence in rural areas: A child-focused assessment of service provision. Journal of Social Policy 2005, 34:165-167. – Book review

For peer review only

Supplementary file 4

Quality appraisal for the included studies (n = 11)

**Quality appraisal criteria**	**Yes**	**Studies**
A. Appropriateness of research methodology and design		
1. Does the research seek to interpret or illuminate the actions and/or subjective experiences of research participants?	11	[29 31 35-43]
2. Is qualitative research the right methodology for addressing the research goal?	11	[29 31 35-43]
3. Is the research design appropriate to address the aims of the research?	11	[29 31 35-43]
4. Has the researcher justified the research design	5	[29 40-43]
B. Ethical considerations		
5. Did the researcher use two or more of the following strategies to ensure ethical issues have been taken into consideration (is there are sufficient details of):<br>a. how the research was explained to participants for the reader to assess whether ethical standards were maintained;<br>b. did the researcher discuss issues raised by the study, such as issues around informed consent or confidentiality or how they have handled the effects of the study on the participants during and after the study;<br>c. was approval sought from an ethics committee)?	8	[29 31 36 38-40 42 43]
C. Credibility (akin to internal validity)<br>Do participants and those with similar experiences recognize the experiences contained with the study?		
6. Did the research use one or more of the following strategies to establish credibility:<br>a. has the researcher discussed saturation of data;<br>b. attempt to triangulate data by using different data collection methods;<br>c. member checking to see if participants agreed with the interpretations of the researcher;<br>d. peers or consultants experienced in qualitative research review their coding process;<br>e. full descriptions of member's words in their final paper?	11	[29 31 35-43]
D. Transferability (akin to external validity)<br>How does one determine the extent to which the findings of the study are applicability in other contexts or with other participant types?		
7. Did the researchers use any of the following strategies to establish transferability (use of dense description of the population studied through descriptions of demographics and geographic boundaries of the study? Note: the author must describe at least two specific sample descriptors (e.g. age range, gender, setting from which sample was selected, SES, etc.)	8	[29 31 35 38-42]

For peer review only

Quality appraisal criteria	Yes	Studies
E. Consistency (akin to reliability)<br>Can another researcher follow the decision trail used by the researcher?		
8. Did the researcher use any of the following strategies to establish the purpose of the research:<br>a. was there a clear statement of aims of the research;<br>b. what was the goal of the research;<br>c. why was it thought important<br>d. its relevance?	11	[29 31 35-43]
9. Was the recruitment strategy appropriate to the aims of the research? (e.g. does the population from which the sample was selected resonate with the research objectives, was the sample selection ethically implemented).	10	[29 31 35-41 43]
10. Did the researcher explain how participants were selected?	9	[28 35 37-43]
11. Did they explain why the participants they selected were the most appropriate to provide access to the type of knowledge sought by the study	4	[28 39 40 43]
12. Were there any discussions around recruitment (e.g. why some people chose not to take part)?	4	[36 38 41 43]
13. Was the setting for data collection justified?	8	[31 37-43]
14. Is it clear how data were collected (e.g. focus group, semi-structured interview etc.)?	11	[29 31 35-43]
15. Did the researcher justify the methods chosen?	3	[29 38 43]
16. Did the researcher make the methods explicit (e.g. for interview method, is there an indication of how interviews were conducted, or did they use a topic guide)?	10	[29 31 35 36 38-43]
17. If the form of data is clear (e.g. tape recordings, video material, notes etc.)?	11	[29 31 35-43]
18. Did the researcher explain how the data were reduced or transformed for analysis?	11	[29 31 35-43]
19. Did they discuss their interpretation and presentation of their findings?	11	[29 31 35-43]
F. Neutrality (akin to objectivity)<br>Did overall credibility, transferability, and consistency occur?		
20. Did the researcher use one or more of the following strategies to ensure neutrality (has the relationship between researcher and participants been adequately considered; has the researcher critically examined their own role, potential bias and influence during the formulation of the research questions or data collection, including sample recruitment and choice of location; did the researcher discuss how they responded to events during the study and whether they considered the implications of any changes in the research design; did the researcher employ field notes to record their personal reactions and biases after each interview/focus group; did they make a conscious effort to follow rather than lead the direction of interviews/focus groups)?	2	[36 43]

Note. Quality was appraised using the modified version of the Critical Appraisal Skill Programme (M-CASP), score range 1-20.[17]

For peer review only

Supplementary file 5

Characteristics of the 11 studies (16 papers) synthesised (in chronological order)

N	Study<br>Paper(s)<br>Country	Aim	Setting	Participants, n	Intervention(s)	Data collection	Data analysis
1	Buckley 2008[a]<br>Buckley 2008 (report) [29]<br>Buckley 2011 (article)<br>[28]<br>Republic of Ireland	Service users' experiences of child protection services	Women's refuges, advocacy groups, family support services, treatment services, counselling service, public redress bodies, community youth project, and statutory child protection service in 16 counties	27 parents<br>20 female, 7 male<br>18 survivors of IPV	Notification by other services<br>Self-disclosure<br>Self-referral<br>First contact and initial response from child protection services	Interviews	Grounded-theory
2	Black 2010 [36],<br>US	Recommended practices and obstacles to practices to improve interventions for families suffering from IPV	Varied health care services in one large urban area	24 HCPs and SCPs<br>20 female, 4 male	IPV screening<br>case finding of children's exposure to IPV<br>Initial care of the mother–child dyad	Interviews	Not stated
3	Stanley 2010[a]	Expectations, views	IPV services for	19 children: age 10-19; 8	Notification by police	Focus groups;	Grounded theory

For peer review only - http://bmjopen.bmj.com/site/about/guidelines.xhtml

N	Study<br>Paper(s)<br>Country	Aim	Setting	Participants, n	Intervention(s)	Data collection	Data analysis
	Stanley 2010 (report) [31]<br>Stanley 2012 (article) [30]<br>UK	and experiences of children's social services	women, services for children affected by IPV, children's social services in two local authorities in the North and South of England	male, 11 female; 16 white British, 1 white/ Asian, 1 white/ black Caribbean, 1 white/ black African.<br>11 parents survivors of IPV: 1 male, 10 female; mean age 38 (25-48); most black minority ethnic (BME), 4 white British.<br>25 SCPs: age 23-65; 4 male, 21 female; 14 front-line, 22 white British, 1 black British, 1 black African, 1 "other"; job experience 1.5-30 years	Response from children's social services to the police notifications	interviews	
4	Meyer 2011 [38]<br>Australia	Help-seeking decisions of IPV victims with dependent children	IPV services, family counselling services in three areas in Southeast Queensland	29 parents survivors of IPV<br>All female<br>Age 21-62 (mean 38, SD 11.3).<br>21 Australian-born, 2 New Zealand-born, 3 UK-born, 1 South African, 1 Malaysian,	Initial response from child protection services	Interviews	Grounded theory

For peer review only

N	Study<br>Paper(s)<br>Country	Aim	Setting	Participants, n	Intervention(s)	Data collection	Data analysis
				1 Filipino.<br>27 had children. 25 children had witnessed the abuse.<br>16 secondary education, 3 in tertiary education, 10 completed tertiary education.<br>7 employed, 2 unemployed, 2 volunteers, 14 home duties, 3 students, 1 retired.<br>26 received government benefits.			
5	Randell 2012 [42]<br>US	Important information to communicate about IPV and how it should be presented in a pediatric emergency department	IPV service in a racially and ethnically mixed city	99 parents survivors of IPV<br>All female<br>36 White, 32 Hispanic, 22 African American, 5 other, 4 not reported.<br>52 high school, 21 degree, 26 not reported.<br>45 currently employed	Identification and response in health care setting	Focus groups	Grounded theory

For peer review only

N	Study<br>Paper(s)<br>Country	Aim	Setting	Participants, n	Intervention(s)	Data collection	Data analysis
6	Davidov 2012 [40]<br>US	Issues related to the mandatory reporting of children's exposure to IPV	Community based Nurse-Family Partnership (NFP) programme in four sites	20 NFP mothers survivors of IPV; mean age 21.3; 50% White, 25% Black, 15% Hispanic; 90% single/ never married; years of school 11.7; 80% income<$24,000<br>47 HCPs:<br>25 NFP nurses; mean age 46.2; 92%>=bachelor's degree; nursing experience 19.7 (5-38); years in NFP 4.4.<br>22 community stakeholders including 4 from hospital or public health services; work experience 9.2 years; mean age 50.5 years.	Mandatory reporting of children's exposure to IPV by NFP nurses	A secondary analysis of focus groups and interviews	Retrospective interpretation
7	Angelo 2013 [35]<br>Brazil	Experience of the nurses in their care of child victims of IPV	Pediatric emergency, intensive care and inpatient units in São Paulo	15 HCPs<br>14 female, 1 male; ages 27-48, time since graduation 3-12 years	Case finding<br>Initial response	Interviews	Motivational theory
8	Jenney 2014 [41]	How the voices of	Child protection	17 SCPs: 12 females, 5	Notification by other services	Focus group;	Grounded theory

For peer review only

N	Study<br>Paper(s)<br>Country	Aim	Setting	Participants, n	Intervention(s)	Data collection	Data analysis
	Canada	women with experience of IPV are translated into child protection service workers' interventions	services in five Ontario child welfare agencies	males; job experience >6 years; qualifications - BA (3), BSW (8), CYW (1), and MSW (5); 9 did not have DVA training, 4 had training in both risk assessment and safety planning.<br>11 parent survivors of IPV: all female; age 26-49 years; 5 immigrant/refugee; 8 on < $25,000 annually; 5 College/University education, 2 high school, 4 grades 9–11.	Self-referral<br>Response from child protection services	interviews	
9	Interview study within the RESPONDS Project[a]<br><br>Szilassy 2015 (report) [34]<br>Larkins 2015 (paper) [34]<br>Szilassy 2016 (paper)	Responses to disclosure of IPV when children are exposed and the dilemmas and challenges general practice clinicians face when confronted with children's exposure to	General practices in six sites across the north and south of England and the Midlands	69 HCPs<br>42 general practitioners, 12 practice nurses and 15 practice managers<br>48 female, 21 male<br>Age: 21-34 – 10; 35-44 – 13; 45-54 – 30; 55-64 – 10; not known – 10	Case-finding<br>Notification by other services<br>Initial response from general practice clinicians	Interviews	Thematic analysis using framework

For peer review only

N	Study<br>Paper(s)<br>Country	Aim	Setting	Participants, n	Intervention(s)	Data collection	Data analysis
	[33]<br>Drinkwater 2016 (paper) [32]<br><br>UK	IPV		Experience managing domestic violence (number of cases): >5 – 5; a few – 14; 1 – 2, none – 26; none but aware of case at surgery - 7<br>IPV service provision: sparse – 28; established – 41<br>Location: metropolitan – 19; urban – 27; semi-rural – 23<br>Region: north – 21; Midlands – 33; south – 33			
10	Clarke 2015 [37]<br>UK	Practitioners' perceptions of children's experiences of IPV.<br>Experiences of responding to the needs of children	Varied services in Wales	54 professionals including HCPs and SCPs<br>5 parent survivors of IPV<br>All female	HCPs and SCPs responses to disclosure	Focus groups; interviews; case studies; meetings observations	Thematic analysis
11	Morris 2015 (thesis) [43]<br>Australia	Perspectives on children's safety and resilience when they	General practices in Victoria	18 parent survivors of IPV: all female; mean age 43 (28-56) years; 2 born overseas.	Screening<br>Case-finding<br>Initial response from general	Interviews; focus groups	Hermeneutic Phenomenological analysis

For peer review only

N	Study<br>Paper(s)<br>Country	Aim	Setting	Participants, n	Intervention(s)	Data collection	Data analysis
		live with IPV		23 children to 16 mothers survivors of IPV: mean age 14 (8-24) years; 2 born overseas	practice clinicians		

Note. a Indicates where more than one paper relates to a single study. IPV – intimate partner violence. HCPs – health care professionals. SCPs – social care professionals; NFP – Nurse-Family Partnership. Quality appraisal using the modified CASP.[17 18]

For peer review only

Supplementary file 6

Descriptive themes

Descriptive theme with subthemes		Stakeholder group, study		
		Children	Parents	Professionals
1. Experiences of CE-IPV identification				
1.1. Approach	1.1.1. How to ask	All children felt that HCPs should initiate a conversation about safety and well-being by giving permission, space and time to discuss sensitive matters.[43]	All mothers felt that HCPs should initiate a conversation about safety and well-being by giving permission, space and time to discuss sensitive matters.[43]	
	1.1.2. When to ask	Most children found it acceptable when HCP initiates enquiry about 'safety at home' from the presenting problem.[43]		Most HCPs described a positive experience of enquiring from the presenting symptoms of children's exposure to IPV or following the disclosure of IPV by mother.[35 36 39 40]
		One young man preferred when HCP asks about 'safety at home' regardless of the presenting problem.[43]		Several practitioners suggested screening for IPV as part of routine pediatric exam.[36]
	1.1.4. Police notification to children's social services of IPV incidents when children were present/		Most mothers were not aware of the police notification.[31]	Some SCPs from children's social services thought that not all incidents of IPV with children present/ resided in the household need to be referred to them. However, they thought that they should be

Descriptive theme with subthemes		Stakeholder group, study		
		Children	Parents	Professionals
	resided in the household			informed of such incidents.[31]
				Some SCPs from children's social services found police notifications inadequate due to the lack of details on children's exposure to IPV.[31]
				SCPs from children's social services found that "filtering out" police notifications to assess whether the family requires further intervention is too time- and labour-consuming.[31]
**2. Experiences of initial response to CE-IPV**				
	2.1. Focus			Most HCPs felt that they should treat the mother-child dyad as a single entity where the needs of the mother were consistent with the child's health and safety needs.[36 39]
				Some HCPs felt conflicted when they had to prioritise the child's needs over mother's needs and confidentiality.[36 39]
				Some HCPs and most SCPs felt that if a child is at risk, they have to prioritise his/ her safety over the mothers' needs and confidentiality.[31 39]
	2.2. Emotional support		Mothers valued when SCPs provided a lot of encouragement.[41]	Some HCPs were positive about providing emotional support and 'leaving the door open' to children to come back with any problems.[34 35 39]

Descriptive theme with subthemes		Stakeholder group, study		
		Children	Parents	Professionals
	2.3. Education		Mothers valued information materials on the impact of IPV on children and IPV resources.[42]	HCPs and SCPs emphasised the importance of educating mothers about the impact of IPV on children and professional responsibilities in cases of children's exposure to IPV. Professionals used education to increase mother's engagement in services.[31 36 39-41]
				HCPs emphasised the importance of educating mothers about the purpose of the home visitation programme and children's social services. Professionals saw education as a pathway to easing mothers' fears of child removal as a result of professionals' involvement.[40]
				SCPs expressed mixed views on the acceptability of sending parents materials about impact of IPV on children in response to the police notification without any other intervention to families.[31]
2.4. Safety	2.4.1. Understanding of safety		Mothers thought that involvement of SCPs resulted in increased risk of abuse escalation and child removal.[28 38 41]	SCPs understood women's and children's safety as being out of the abusive relationship and believed that their involvement resulted in increased safety for both mothers and children.[38 41]
			Some actions that mothers perceived as preventing abuse escalation and	Some actions that mothers perceived as increasing child safety were seen by SCPs as increasing risk for

Descriptive theme with subthemes	Stakeholder group, study		
	Children	Parents	Professionals
		increasing child safety (e.g., staying with perpetrator, not seeking help) were seen by SCPs as increasing risk for children.[38 41]	children.[38 41]
		Mothers did not feel safer after they complied with SCPs' requirements and left the abusive partner.[38 41]	
		Women wanted to talk with children's social workers about safety and safety planning.[41]	
2.4.2. Assessment of risk	One child suggested that HCPs need to ask children about their safety in all the environments that a child inhabits (e.g., all households post-separation, school).[43]		
			SCPs and HCPs favoured varied approaches to risk assessment (e.g., observation, information gathering from mother and other professionals, gut feeling, previous experiences).[31 39 41]
			Some SCPs found standardised tools helpful for risk assessment (e.g., the Barnardo's Domestic Violence Risk toll).[31]

Descriptive theme with subthemes	Stakeholder group, study		
	Children	Parents	Professionals
			Some SCPs saw the existing risk assessment as a bureaucratic exercise and believed that it does not measure real risk from the perpetrator[31 37 41]
2.4.3. Safety planning		Most mothers were not involved in safety planning with their SCPs therefore could not comment on its acceptability.[41]	SCPs found undertaking safety planning with both children and mothers to be acceptable.[31]
			Although SCPs understood that they could not change women, they believed that leaving an abusive partner is the most effective form of safety planning.[41]
			Some SCPs felt that they monitor the risk instead of doing direct safety planning with mothers and children and wanted more direct engagement with them.[31 37]
			Some HCPs found it acceptable to undertake safety planning with women who were not ready to leave the perpetrator.[36]
2.5. Signposting		Mothers appreciated when health visitors signposted them to other services.[31]	Two GPs felt comfortable when giving children information about services they could access.[39]
			Some HCPs and SCPs felt comfortable providing

Descriptive theme with subthemes		Stakeholder group, study		
		Children	Parents	Professionals
				mothers with information about IPV resources. They saw signposting as a mechanism to facilitate engagement in services.[31 39 41]
	2.6. Documenting			Most HCPs reported uncertainty and confusion about recording policies and practices on children's exposure to IPV. The uncertainty was reported at local, region and national levels.[32 36 39 40]
				HCPs feared possible breach confidentiality of parents exposed to IPV by recording children's exposure in child or parent medical records.[32 39]
	2.7. Reporting			HCPs described uncertainty and confusion about their reporting responsibilities especially in cases of child exposure to psychological IPV and non-direct physical IPV.[36 39 40]
			Mothers felt uncertain about reporting responsibilities of HCPs and feared that HCPs could initiate child removal.[40]	HCPs emphasised the importance of explaining their reporting responsibilities and procedures to mothers. Professionals use such education to ease mothers' fears of child removal, to increase engagement with services, and to justify breach of the mothers' confidentiality after IPV disclosure.[39 40]
**3. Factors that facilitated identification and initial response to CE-IPV**				

Descriptive theme with subthemes	Stakeholder group, study		
	Children	Parents	Professionals
3.1. Mother's readiness to disclose and engage		Mothers reported past experiences when they were not ready to acknowledge the impact of IPV on their children, disclose IPV and engage with services.[31 38 42]	HCPs and SCPs believed that the approach to identification and initial response should match mother's readiness to disclose IPV and engage with services.[31 36 41]
3.2. Patient-professional relationship	Most children emphasised the importance of 'knowing and being known' to the doctor, which made them feel comfortable to talk about personal issues.[43]	Most mothers emphasised the importance of a long-term trusting relationship with the same professional for IPV disclosure and engagement with services.[28 38 41 43]	Most HCPs and SCPs emphasised the importance of a long-term trusting relationship with mother for IPV disclosure and engagement with services.[28 29 36 41]
		Mothers suggested that they could be role models for their children in developing trust and building relationship between child and doctor.[43]	Only one HCP suggested that existing relationships could enable proactive and direct engagement with children.[34 39]
			Some HCPs thought that referral to children's social service can worsen their relationship with a parent who discloses IPV.[34 36 39]
			Some HCPs feared that professionals with strong relationships with their patients are more likely to develop vicarious trauma.[36]
3.3. Positive professional	Children described preferred	Mother appreciated HCPs and SCPs who	HCPs emphasised the importance of

Descriptive theme with subthemes		Stakeholder group, study		
		Children	Parents	Professionals
	attitude	professionals as good at listening, making them feel that they were welcome back, helping them practically.[30 31 43]	took them seriously, validated their accounts, ensured confidentiality and helped practically.[28-31 38 40 41]	nonthreatening and non-judgemental attitudes with frequent reassurance of confidentiality when responding to IPV disclosure.[36 39 40]
3.4. Engaging directly with children	3.4.1. Talking to children at consultation	Children appreciated when professionals talk directly to them, instead of to their mothers.[30 31 43]	Mothers stressed the importance of giving each child a voice and addressing each child's needs at a medical consultation.[43]	Most HCPs expressed concerns in children's competence as informants and believed parents more than children.[34 39]
				Some HCPs expressed concern about their lack of competence in communicating directly with children.[39]
				A few HCPs who directly engaged with children were female general practitioners in affluent areas with high levels of IPV service provision.[34]
				SCPs felt confident talking to children when assessing and intervening in families with IPV.[31]
				SCPs felt that they should listen to the child more when assessing and intervening in families with IPV.[37]
	3.4.2. Appropriate age for talking to children at consultation	Most children regardless of age preferred their mother (trusted adult) to be present in a medical	Mothers found it acceptable if doctors speak to children from the age of five. They believed that children exposed to	There was no age above which clinicians consistently agreed it would be acceptable to talk to children about IPV; some were hesitant about

Descriptive theme with subthemes		Stakeholder group, study		
		Children	Parents	Professionals
		consultation.[43]	IPV mature quicker than non-exposed children.[43]	even talking to teenagers.[34 39]
			Mothers preferred doctors to use non-verbal methods when communicating with young children.[43]	
			Mothers of adolescents believed that their children would find it difficult to talk to doctors because of teenage behavioural traits.[43]	
	3.4.3. Seeing children alone	Some children felt that they should be of adolescent-age before speaking alone with the doctor about their safety and well-being.[43]		Most general practice clinicians felt that they should not see children alone at consultation.[34 39]
		Most children wanted their mothers (trusted adult) to be present at the medical consultation.[43]		Few general practice clinicians who would see children alone thought that it is acceptable to seek permission from the non-abusive parent and from the child without describing how it should be done.[39]
				SCPs regularly saw children alone when assessing risk, although they found it challenging to talk to children alone.[31]

Descriptive theme with subthemes		Stakeholder group, study		
		Children	Parents	Professionals
4. Reasons for not identifying/ disclosing CE-IPV and not engaging with services				
	4.1. Awareness of children's exposure to IPV		Mothers recognised that it took long time to acknowledge that IPV had an impact on their children.[28 31 38 41 42]	Most HCPs felt uncertain and confused about what constitutes children's exposure to IPV in cases of psychological IPV and non-direct physical IPV and how to respond to such cases.[39 40]
			Mothers felt that SCPs are unaware of the dynamics of IPV and risks involved.[28 30 31 38 41]	SCPs believed that mothers' underestimating of the impact of IPV on children was a barrier to disclosure and engagement with services.[30 31 41]
4.2. Perceived role	4.2.1. Someone else's	Most children believed that HCPs' role is to treat illness and monitor their health and well-being, although health was narrowly defined as physical (e.g., injuries, vaccination).[43]	Mothers believed that HCPs' role is to treat illness and monitor their health and well-being, although health was narrowly defined as physical (e.g., injuries, vaccination).[43]	HCPs believed that Identifying and responding to children's exposure to IPV is someone else's role (e.g., health visitor, social worker, school nurse).[31 39]
		Two children recognised a potential role for HCPs in caring about children beyond physical illness.[43]		
				HCPs and SCPs believed that it is a mother's role to seek help and stop IPV.[31 35 37-41]
	4.2.2. Threatening and punitive	Children perceived children's social service's role as threatening and	Mothers perceived children's social service's role as threatening and	HCPs and SCPs perceived children's social service's role as threatening and punitive and recognised

Descriptive theme with subthemes		Stakeholder group, study		
		Children	Parents	Professionals
		punitive.[30 31]	punitive.[30 31 40 41]	that mothers and children felt the same.[30 31 40]
				SCPs perceived health visitors as less threatening and more acceptable to families.[31]
	4.2.3. Uncertainty		Some mothers felt confused and uncertain about the role of children's social services which led to anxiety and avoidance of HCPs and SCPs.[30 31]	
	4.2.4. Mandatory reporting			HCPs felt confused and uncertain about their role as mandated reporters of children's exposure to IPV, especially in cases of psychological IPV and non-direct physical IPV.[40]
			Mothers perceived all HCPs as mandated reporters of children's exposure to IPV which led to anxiety and avoidance of HCPs.[40]	HCPs recognised that their role as mandated reporters prevented mothers from disclosing IPV and engaging with services.[40]
	4.3. Fears	Children feared being removed from the home and taken into care.[30 31 37]	Mothers feared the removal of children by social services.[28-31 38 40 41]	HCPs and SCPs recognised that varied fears prevented mothers and children from disclosing IPV and engaging with services.[31 40 41]
		Children feared the negative consequences of IPV disclosure (e.g., escalation of abuse, involvement of children's social	Mothers feared the negative consequences of IPV disclosure (e.g., escalation of abuse, involvement of police and children's social service).[38	HCPs feared the negative consequences of identifying and responding to CE-IPV (e.g., escalation of abuse, misunderstanding, involvement of children's social service, threats

Descriptive theme with subthemes		Stakeholder group, study		
		Children	Parents	Professionals
		services).[37 43]	43]	from parents, damage to the doctor-patient relationship, legal consequences).[31 32 34-36 39]
			Mothers feared HCPs because of their mandated reporter responsibilities.[40]	HCPs recognised that mothers feared them because of their mandated reporter responsibilities.[40]
	4.4. Stigma	Children recognised the stigma, shame, and embarrassment associated with disclosing IPV.[30 31]	Mothers recognised the stigma, shame and embarrassment associated with disclosing IPV.[30 31 42 43]	
4.5. Influences from the wider environment	4.5.1 Social and cultural factors		Mothers who were non-native speakers expressed the need for information on children's exposure to IPV in their native language.[42]	SCPs and HCPs recognised that language and cultural norms and stereotypes make it difficult to engage with mothers from ethnic minorities.[31 36]
			Immigrant mothers wanted materials on children's exposure to IPV that address the immigration issues and culture-specific fears of IPV disclosure.[42]	
	4.5.2. Healthcare and social care system factors			HCPs and SCPs perceived the lack of contact time with patients/ clients as a major barrier to identifying and responding to children's exposure to IPV.[36 37 39]
				HCPs did not perceive children's exposure to IPV as their top priority.[39]

Descriptive theme with subthemes	Stakeholder group, study		
	Children	Parents	Professionals
			HCPs and SCPs highlighted that their work with children was negatively affected by the cutbacks and constant reforms in home visitation services and social services.[31 39]
			HCPs and SCPs highlighted the lack of services for all family members which prevented them from asking about and responding to children's exposure to IPV.[31 36 39]
	A few children suggested that police and social workers should communicate better in cases of IPV to ensure children's safety and adequate support.[30 31]		HCPs and SCPs were concerned with the lack of communication between the different providers and expressed a need for better communication and a more coordinated response to children's exposure to IPV.[31 36 37 39]
**5. Psychological consequences of identification and initial response to CE-IPV**			
5.1. Emotional burden	Children felt disappointed and powerless as a result of negative experiences with SCPs.[30 31]	Parents felt disappointed, stressed, hopeless and frustrated as a result of negative experiences with SCPs.[28-31 38]	
	Children thought that involvement with SCPs was 'stressful' for their mothers.[31]		
		Mothers felt embarrassed and guilty	HCPs and SCPs felt frustrated and conflicted

For peer review only - http://bmjopen.bmj.com/site/about/guidelines.xhtml

Descriptive theme with subthemes	Stakeholder group, study		
	Children	Parents	Professionals
		when acknowledging the impact of IPV on their children and when disclosing IPV to professionals.[30 31 42 43]	towards mothers who did not follow their advice.[35 36 41]
			HCPs felt conflicted when changing the focus from the mother-child dyad to the individual child.[36 39]
			HCPs and SCPs felt uncomfortable about pressuring mothers to leave the perpetrator to protect children, but thought that they had few options in situations where mothers and children continued to live with the perpetrator.[36 39]
5.2. Vicarious trauma			HCPs feared the possibility of burnout and hardening towards women and children.[35 36]
**6. Training and resources suggested**			
6.1. Professional training			HCPs preferred interactive training built upon real cases.[33 39]
			HCPs asked for training on communication with children.[33 39]
			HCPs and SCPs wanted to learn about other services' roles and responsibilities in responding to children's exposure to IPV.[31 33 39]
6.2. Information materials		Mothers favoured single posters in	HCPs found IPV posters in health care settings to

Descriptive theme with subthemes		Stakeholder group, study		
		Children	Parents	Professionals
			pediatric settings about signs of IPV, effect of IPV on children and local IPV services.[42]	be acceptable in facilitating IPV disclosure and self-referral to IPV services.[36]
			Mothers who were non-native speakers favoured information materials in their native language.[42]	HCPs wanted local signposting materials on IPV services.[33 39]
			Mothers preferred information materials with positive non-judgemental message, representing ethnic and social diversity[42]	HCPs wanted materials on policies and procedures about mandatory reporting of children's exposure to IPV.[40]

Note. IPV – intimate partner violence. HCPs – health care professionals. SCPs – social care professionals….. – converging perspectives. … . – diverging perspectives. …. - conflicting perspectives.

For peer review only - http://bmjopen.bmj.com/site/about/guidelines.xhtml

Supplementary file 7

Interim analytical themes and their definitions

Interim analytical theme with subthemes	Stakeholder group, study			
	Children	Parents	HCPs	SCPs
**1. Converging perspectives on satisfactory strategies for identification and initial response to children's exposure to IPV**				
**1.1. Facilitators of acceptable identification and initial response**				
**1.1.1. Patient-professional relationship.** Some children, most mothers and most professionals highlighted that central to any work around children's exposure to IPV were aspects of the patient-professional relationship. When describing a good relationship, stakeholders talked about the long-term process of relationship building, continuity of contact, trust and effective communication.	[43]	[28 38 41 43]	[36 39]	[28 41]
**1.1.2. Positive professional attitudes and skills.** Patients/ clients and HCPs described how a non-judgemental non-threatening attitude, showing respect, validation of patients' accounts and reassurance of confidentiality facilitated disclosure and engagement with services.	[31 43]	[28 31 38 40 41]	[36 39 40]	
**1.1.3. Safety & comfort.** Good patient-professional relationships enabled children and mothers to feel safe and comfortable when discussing sensitive issues.	[43]	[43]		
**1.1.4. Individual mother's readiness.** Mothers and professionals suggested that the individual mother's readiness to disclose IPV and seek help should be considered when asking about IPV and responding to disclosure.		[38 42]	[36]	[31 41]
**1.1.5. Patient materials.** Mothers and HCPs thought that printed materials for patients about the effect of IPV on children and local IPV resources should be displayed in health-care settings to facilitate IPV disclosure by mothers and provide information for self-help.		[42]	[36]	

For peer review only

Interim analytical theme with subthemes	Stakeholder group, study			
	**Children**	**Parents**	**HCPs**	**SCPs**
**1.2. Identification process**				
**1.2.1. Consultation atmosphere.** Children and mothers desired permission, space and time to discuss sensitive issues with HCPs.	[43]	[43]		
**1.2.2. Approach to enquiry.** Most children and HCPs preferred a case-based approach (from presenting symptoms).	[43]		[35 36 39 40 43]	
**1.2.3. Phased inquiry.** Patients and professionals were positive about HCPs initiating the enquiry. They preferred professionals to ask about children's exposure to IPV as a 'safety-at-home' issue – starting from the presenting symptoms, through questions about general well-being and the family situation to questions about feeling safe at home.	[43]	[43]	[35 36 39 40]	
**1.3. Initial response process**				
**1.3.1. Emotional support.** Mothers and professionals were positive about providing a lot of encouragement and emotional support to both children and mothers.		[41]	[35 39]	
**1.3.2. Patient/client education.** Mothers and professionals were positive about patient/client education about the impact of IPV on children, IPV dynamics, professional roles and duties. Professionals highlighted the importance of considering women's safety when sending letters about the impact of IPV on children.		[42]	[36 39 40]	[31 41]
**1.3.3. Signposting.** Mothers and professionals were positive about signposting mothers and children to IPV services.		[31]	[39]	[31 41]
**2. Converging perspectives on barriers to satisfactory identification and initial response to children's exposure to IPV and proposed solutions**				
**2.1. Fears.** All stakeholder groups shared varied fears. The fear of abuse escalation, involvement of children's social services and child removal were mentioned by all groups. Additional professional fears of negative consequences included threats from parents, negative impact on the therapeutic relationship with parents	[31 37 43]	[28 31 38 40 41]	[35 36 39 40]	[31 41]

For peer review only

Interim analytical theme with subthemes	Stakeholder group, study			
	Children	Parents	HCPs	SCPs
and legal consequences. Professionals used education to ease mothers' fears.				
**2.2. Gaps in knowledge and skills**				
**2.1.1. Low awareness.** All stakeholder groups demonstrated low awareness about children's exposure to IPV, especially psychological and non-direct physical IPV and delegated responsibilities of identifying and responding to somebody else.	[31 43]	[28 31 38 41-43]	[39 40]	[31 41]
**2.1.2. Communicating with children.** HCPs wanted to improve their communication skills in talking directly to children during medical consultations.			[39]	
**2.1.3. Documenting.** Professional described uncertainty and confusion regarding how and where to document children's exposure to IPV.			[32 36 40]	
**2.1.4. Mandatory reporting.** Mothers' uncertainty and confusion about HCPs' duties as mandated reporters mirrored professional uncertainty and confusion, especially in cases of children's exposure to psychological and non-direct physical IPV.		[40]	[36 39 40]	
**2.2. Emotional burdens.** All stakeholders were emotionally involved in the work of identifying and responding to IPV. Clients reported feeling threatened and pressured by SCPs to leave the perpetrator without adequate support provided. Professionals described psychological distress caused by ambivalent feelings towards mothers when dealing with children's exposure to IPV and were concerned that involvement in helping abused women and their children could cause vicarious trauma.	[31]	[28 31 41]	[35 36 39]	[31]
**2.3. System level factors**				
**2.3.1. Language and culture.** Stakeholders described mothers' immigration status, community norms and poor English as additional barriers to identifying and responding to children's exposure to IPV.		[42]	[36]	[31]
**2.3.2. Inter-agency work.** Stakeholders were similarly concerned with the lack of communication between different providers and expressed a need for a better communication, information sharing and a more	[31]		[36 39]	[31 37]

For peer review only

Interim analytical theme with subthemes	Stakeholder group, study			
	Children	Parents	HCPs	SCPs
coordinated approach when responding to children's exposure to IPV.				
**2.3.3. Stretched services.** Professionals repeatedly mentioned: (i) lack of contact time with patients/clients, (ii) high demand and competing priorities, (iii) constant reforms to health care and social services, and (iv) shortage of services for all family members.			[36 39]	[31 37]
**3. Suggested training and resources** Professional training and resources. Professionals favoured a case-based training, wanted to improve their communication skills, learn about the roles of and responsibilities of other services and highlighted the importance of inter-agency collaboration. HCPs requested clear instructions on mandatory reporting and local signposting materials			[36 39]	[31]
**4. Diverging perspectives on satisfactory initial response**				
**4.1. Safety.** Mothers and professionals disagreed on the understanding of safety for the child. Actions perceived by mothers as increasing children's safety were seen by SCPs as jeopardising it. Most SCPs believed that their involvement increased children's safety and required mothers to leave the perpetrator. In contrast, most mothers thought that SCPs' involvement posed a threat to the child through leading to IPV escalation and child removal. Women did not feel safer after leaving the perpetrator because of potential escalation of abuse and child contact.		[28 38 41]		[38 41]
**4.2. Talking directly to children.** While most recipients of care were positive about professionals talking directly to children from a young age and addressing their individual needs, most HCPs did not see children as patients on their own and assessed children's needs through a proxy adult. Children and HCPs had conflicting views about the appropriate age for talking to children about IPV and about appropriateness of seeing children alone.	[31 43]	[43]	[71]	[31 37]
**5. Conflicting perspectives on satisfactory identification and initial response**				

For peer review only

Interim analytical theme with subthemes	Stakeholder group, study			
	Children	Parents	HCPs	SCPs
**5.1 Shifting focus.** HCPs and SCPs had conflicting perspectives on the focus of their responses – child vs mother-child dyad/family. Although all agreed that the focus should switch to the child when he/she is at risk of harm, some felt conflicted when prioritising children's safety and needs over mother's' safety.			[36 39]	[31]
**5.2. Assessment of risk.** While children wanted to be involved in risk assessment and some SCPs found it acceptable to talk to children when assessing the risk, most professionals were not satisfied with the existing risk assessment process and saw it as a ticking box exercise.	[43]		[39]	[31 37 41]
**5.3. Safety planning.** While some HCPs found it acceptable to undertake safety planning with women who were not ready to leave the abusive partner, most SCPs saw leaving the abusive partner as the best safety planning for the child. SCPs had mixed views on the current safety planning process. Mothers wanted better communication with SCPs when undertaking safety planning.			[36]	[31 37 41]

Note. IPV – intimate partner violence. HCPs – health care professionals, SCPs – social care professionals.

For peer review only

Supplementary file 8

Final analytical themes supported by raw qualitative data from included studies

Theme	Subtheme	Participant quotation	Authors interpretation quotation
1. Precursors for acceptable identification and response to children's exposure to IPV	1.1. Satisfying and sustainable relationship	"Have confidence around them...I guess it depends how much you know your doctor. It's just depending on how well you know 'em. (Zoe, 12)"[43]<br><br>"Well in domestic violence I find that it's always the relationship that we have with the client, with the mom in this case — the stronger our relationship with the mom and the more we can understand her, understand her world through her eyes instead of imposing our view, we get them more on board. Once we can get that trust, then we can work together. [Worker #11]"[41]	"In common with participants in the wider study, they [parents] placed a lot of value on the type of relationship they could build up with workers [child protection workers] and the degree of trust that developed thereafter."[28]
	1.2. Desired professional attitudes and skills	"My doctor was really good, like once I went there and she was like really concerned and she sent me to someone else and then she told me to come back in a week so she could check on me and then, you know...like she kept making appointments so she could see me again and again. So it's good when doctors care like that...Makes you want to then	"Where service users reported positive experiences with the child protection services, it was normally when they felt they had been listened to fairly and believed. They also appreciated it when workers took direct action."[28]

For peer review only

Theme	Subtheme	Participant quotation	Authors interpretation quotation
		maybe go and um always go to them. (Claire, 21)"[43]<br><br>"I'm grateful that they actually heard me, heard my voice and listened to me (Client#11)"[41]	
	1.3. Considering mother's readiness	"[I] lost the children—my three youngest girls plus my son. Because the Department saw that I—which, looking back now, I understand what they're saying— that I put my children in a dangerous situation … they could very well have gotten hurt. But I don't think I deserved to lose them—I needed help to get out of that situation and I asked for help to get out of that situation."[38]	"It was suggested that a comprehensive system of support could be achieved through coordination of education, safety, and support services with the stage of change of the caregiver (P 1 and 14). Whereas victims in the early stages needed information, victims in later stages needed shelter, support groups, or legal services (P 11 and 16)."[36]
	1.4. Patient materials	"Doctors should ask about exposure. We can invite the conversation through literature and posters. Some believe that we should not ask. It is not the doctor's role. Some people [victims] are not ready to do anything. For those people, you just say, "Here is some info. (P 14)"[36]	"We found that women with histories of IPV support positive educational interventions in the PED [Pediatric Emergency Department] and recommend interventions that educate about resources, signs of IPV, and the effects of childhood IPV exposure in a non-judgmental and positive manner."[42]
	1.5. Professional training	"…other agencies, they don't actually understand what our role is, you know…what would be really, really good is people should shadow…and they should do some training on what the role, the roles	"Participants voiced a need to deconstruct the misperception that CEDV [children's exposure to domestic violence] is harmless and to educate professionals, health care providers, judges, police

For peer review only

Theme	Subtheme	Participant quotation	Authors interpretation quotation
		of different agencies are and what different agencies can actually offer. (Child protection social worker 4)"[31]	officers, and school staff on the hazards of CEDV (P 19)."[36]
	1.6. Professional resources	"I think you need to have in your literature about mandated reporting because, as nurses, we are mandated reporters and we need to know, again, with that role it needs to be spelled out very clearly because in a court of law that's what would be thrown at us, if we have fulfilled our responsibilities in what way. (Nurse)"[40]	
	1.7. Professional supervision	"[...] I don't feel much impact, because, it's like, we get so many [children], recently there have been a lot, and so before we used to get more [impacted], but these days it is hard to go through one month in which you don't have one or two cases of poor treatment, sexual abuse [...] I have already had so many [cases] that we are beginning to become accustomed with the situation [...] (N15)."[35]	"Conversely, providers with strong relationships with their patients may be more susceptible to frustration, burnout, or hardening toward victims who are not ready to leave abusive relationships." (P 19).[36]
	1.8. Addressing systems' barriers	"I know people can be very protective about information sharing. 'Well *you're* working with the adult' and '*You're* working with the child', so there is not always that joined-up thinking about risks as a whole. (Specialist Social Worker: 1)"[37]	"Some of the reasons given for not exploring the possibility of DVA [domestic violence and abuse] when child safeguarding concerns arose included DVA not being 'first on your radar or list of things to ask about' (GP31) and the problem of 'finding the

For peer review only

Theme	Subtheme	Participant quotation	Authors interpretation quotation
			time to do [it] all' (GP28)."[39]
2. Acceptable identification	2.1. Space and time	"It makes it a lot easier when they say that you can take your time. Like even for the first session…you don't have to do anything, you can just try and get used to being there and stuff. (Amelia, 15)"[43]<br><br>"Five years, same woman, all same—then I start to tell them everything then they said why you didn't tell me before? Then I tell them you know I was so scared that time like I feel safe with them to talk about everything then my children aid worker she find out shelter for me. [Client #9]"[41]<br><br>"The sense that it's okay to come and talk to you about anything that worries them. (GP21)"[39]	
	2.2. Vocabulary	"Ask the kids if they're OK…(Lisa, Claire's mother) Yeah, I mean that's exactly right (agreeing with Lisa in the focus group).<br>(Doctor) 'Are_you_OK?' (Patient) 'Oh no'. (Doctor) 'Why? Would you like to talk about it?' I think, and they're simple little words…(Penelope, mother)"[43]	
	2.3. Phased approach	"I brought up the fact that I wasn't sleeping like normal people and that it was affecting my school	

For peer review only

Theme	Subtheme	Participant quotation	Authors interpretation quotation
		work because I really, really want to do well at school. So he kind of asked a few questions and stuff to find out what else is affecting me… (Amelia, 15)"[43]<br><br>"But there might be other things like sleeping issues, so then the doctor might go, 'OK, so why aren't you sleeping, do you think?' And then that might lead to the child opening up, 'Well this is happening and…Mum and Dad are fighting all the time and…(Lisa, mother)"[43]<br><br>"If they start talking about domestic violence then at that point engage them in the discussion about, "Well, do you realize that that is a form of child abuse? Not that I'm going to report you today or anything but I want you to be aware of that. (Community stakeholder)"[40]	
3. Acceptable initial response	3.1. Shifting focus	"We are child focused so this is what you need to do to keep your children safe, but obviously you need to be safe to do that, so it sort of interlinks, but certainly the children are our first concern. I mean, you know, if the victim is unable to protect their	"Throughout the interviews, it became apparent that although the project focused on children, practitioners treated the mother–child dyad as a single entity where the needs of the mother were consistent with the child's health and safety

For peer review only

Theme	Subtheme	Participant quotation	Authors interpretation quotation
		child, then we need to look at whether there is more action that we need to do, something a bit sort of formal as opposed to giving advice. (Initial assessment manager 4)"[31]<br><br>"I'd have to tell her that, because of the children, I would basically be needing to involve child protection services. I would reassure and inform her as much as possible as to what that would involve, but yes, I'd have to make her aware that, you know, it wasn't actually up to her whether I, because I often get into arguments about 'oh no, but they'll take the children… (GP30)"[39]	needs."[36]
	3.2. Emotional support	"But, within my capabilities, what I did was intensify surveillance, and try my best to help the child emotionally [...] (N4)."[35]<br><br>"she used to give me a lot of encouragement, tell me I was doing a good job in spite of you know all the circumstances?" "just thinking of her words does help you know, when I'm being a bit hard on myself as a mom (Client #8)"[41]	
	3.3. Education	"Initial care of the mother–child consisted of	

For peer review only

Theme	Subtheme	Participant quotation	Authors interpretation quotation
		gathering information, helping the mother to view the relationship honestly and objectively, educating the mother on potentially dangerous situations and ways to avoid or prevent abuse. Documenting provider efforts in the patient chart facilitated follow-up. (P 15)"[36]<br><br>"There's already a lot of anxiety if you know talk about Children Services. . . . Hopefully [CPS] could be seen as more of a positive support for this mom and the children. . . . One thing is just to educate and talk to moms about what role Children Services can play, and seeing it more as a positive not just a negative experience. . . .Educate that, you know, Children Services is here to help, Children's Services isn't here to remove your kids. (Community stakeholder)"[40]	
	3.4. Signposting	"She was really helpful, …, she was the one that gave us the number for the NSPCC. She was just good at listening to us and that. (Nicola, Young People's Focus Group 1)" [31]	"Two examples were given of GPs overcoming the lack of time and giving young people information directly about relevant services they could access."[39]
4. Conflicting perspectives on	4.1. Engaging directly with children	"It depends on how they (doctors) approach them. And you've got to treat them as an individual, not as	"Lisa and Penelope suggest five as a suitable age to engage directly with children about their safety

For peer review only

Theme	Subtheme	Participant quotation	Authors interpretation quotation
acceptable identification and response		a (sibling) group." (Focus group participants all agree).(Charlie, mother)"[43]<br><br>". . . for me it's opening the opportunities for children to speak. Because key to it is understanding the children's view of it. The parents can say, 'Oh I love him, I'll never do it again'. . . . What's happening for the kid within all this? (Senior Social Worker: 2)"[37]<br><br>"When you complete initial assessments you do see the children alone and often they are quite young, and direct questions are very difficult. You are only in the house for an hour, an hour and a half doing your initial assessment. They usually know why this lady is coming to the house and they have been told not to say anything. You always ask if there is anything they would like to talk about, how are things at home, you know, what happens if they are naughty, do they go on the naughty stair, or, you know, but it's very difficult. (Initial assessment worker 3)"[31]	based on the level of awareness that their own children had at that age."[43]<br><br>"Whilst not precluding the doctor from addressing a child directly in a consultation where their mother is present, all the children and young people suggested an older age range [15-18] for being seen on their own."[43]<br><br>"Few clinicians would routinely seek to directly engage with the children concerned. Some might in some circumstance, after undertaking other actions such as talking to others (e.g. health visitors); or if an opportunity arose or excuse could be made. More than half would not seek to engage directly with children."[39]
	4.2. Management of safety	"AM: And in what way can doctors and nurses help families?	"Although formally asked about safety plans, many women either had not been involved in safety

For peer review only

Theme	Subtheme	Participant quotation	Authors interpretation quotation
		By checking if they're sick. Checking… if they're OK, feeling safe and all that.<br>AM: OK, what would be the best way that they could ask you if you were feeling OK?<br>Um…are you safe at home and at school and that…and at Dad's. (Fred, 9)"[43]<br><br>"We talk a lot about options if she is ready to leave [the abusive situation]. The options, if she is not ready to leave, a lot has to do with education, what are the dynamics of domestic violence, helping her to understand what is going on in her relationship, helping her to look at the cycle of violence, and to begin to recognize what is going on. A lot of what we do, if somebody is not ready to leave, has to do with safety planning and not safety planning about leaving, but safety planning while you are in the relationship. . . . If a violent incident happened, where can you tell the kids to go? Can the kids go upstairs? Can you teach them to dial 911? Can you teach them to leave and go to a neighbor's house? Who can the kids talk to? How do you talk to your kids about what is going on? And then, how do you	planning (or did not recall doing so) with their workers."[41]<br><br>"Similar to the victims who decided against leaving after approaching CPS [child protection service] for support, most victims who remained silent altogether believed they were acting in the best interest of their children at the time…. Some of the non-help-seeking decisions of victims are therefore well-informed decisions, often based on victims' individual risk assessment and their perception of how they can best ensure their children's safety at that particular point in time."[38]<br><br>"A common theme across the interviews with practitioners was the need for a shift towards more direct working with children and their parents. There was a widespread belief that current policy focused on monitoring risk, rather than addressing risk through systematic safety planning with adult victims and direct safety planning with children."[37]

For peer review only

Theme	Subtheme	Participant quotation	Authors interpretation quotation
		get yourself safe? Not staying in the kitchen, not being in the bathroom, not staying in a place where you don't have an exit, not wearing scarves around your neck because they can be used to choke you. There are lots and lots . . . and for every person the safety plan is going to look a little bit different. (P 2D)"[36]	

For peer review only

Supplementary file 1.

ENTREQ checklist (Enhancing transparency in reporting the synthesis of qualitative research[14]

No.	Item	Guide and description	Reported on Page #
1	Aim	State the research question the synthesis addresses	Introduction, p.6-7
2	Synthesis methodology	Identify the synthesis methodology or theoretical framework which underpins the synthesis, and describe the rationale for choice of methodology (e.g. meta-ethnography, thematic synthesis, critical interpretive synthesis, grounded theory synthesis, realist synthesis, meta-aggregation, meta-study, framework synthesis)	Synthesis, p. 10
3	Approach to searching	Indicate whether the search was pre-planned (comprehensive search strategies to seek all available studies) or iterative (to seek all available concepts until they theoretical saturation is achieved)	Information sources, p. 8
4	Inclusion criteria	Specify the inclusion/exclusion criteria (e.g. in terms of population, language, year limits, type of publication, study type)	Eligibility criteria, p. 7-8 Table 1
5	Data sources	Describe the information sources used (e.g. electronic databases (MEDLINE, EMBASE, CINAHL, psycINFO), grey literature databases (digital thesis, policy reports), relevant organisational websites, experts, information specialists, generic web searches (Google Scholar) hand searching, reference lists) and when the searches conducted; provide the rationale for using the data sources	Information sources, p. 8-9
6	Electronic search strategy	Describe the literature search (e.g. provide electronic search strategies with population terms, clinical or health topic terms, experiential or social phenomena related terms, filters for qualitative research, and search limits)	Information sources, p. 8-9 Online appendix 2
7	Study screening methods	Describe the process of study screening and sifting (e.g. title, abstract and full text review, number of independent reviewers who screened studies)	Studies selection, p. 9
8	Study characteristics	Present the characteristics of the included studies (e.g. year of publication, country, population, number of participants, data collection, methodology, analysis, research questions)	Study characteristics p. 13-14 Online appendix 5
9	Study selection results	Identify the number of studies screened and provide reasons for study exclusion (e,g, for comprehensive searching, provide numbers of studies screened and reasons for exclusion indicated in a figure/flowchart; for iterative searching describe reasons for study exclusion	Searches p. 11 Figure 1

For peer review only

		and inclusion based on modifications to the research question and/or contribution to theory development)	
10	Rationale for appraisal	Describe the rationale and approach used to appraise the included studies or selected findings (e.g. assessment of conduct (validity and robustness), assessment of reporting (transparency), assessment of content and utility of the findings)	Quality appraisal p. 9
11	Appraisal items	State the tools, frameworks and criteria used to appraise the studies or selected findings (e.g. Existing tools: CASP, QARI, COREQ, Mays and Pope [25]; reviewer developed tools; describe the domains assessed: research team, study design, data analysis and interpretations, reporting)	Quality appraisal p. 9
12	Appraisal process	Indicate whether the appraisal was conducted independently by more than one reviewer and if consensus was required	Quality appraisal p. 9
13	Appraisal results	Present results of the quality assessment and indicate which articles, if any, were weighted/excluded based on the assessment and give the rationale	Critical appraisal p. 13<br>Figure 2
14	Data extraction	Indicate which sections of the primary studies were analysed and how were the data extracted from the primary studies? (e.g. all text under the headings "results /conclusions" were extracted electronically and entered into a computer software)	Data extraction p. 9
15	Software	State the computer software used, if any	Data extraction p. 9
16	Number of reviewers	Identify who was involved in coding and analysis	Synthesis p. 10
17	Coding	Describe the process for coding of data (e.g. line by line coding to search for concepts)	Synthesis p. 10
18	Study comparison	Describe how were comparisons made within and across studies (e.g. subsequent studies were coded into pre-existing concepts, and new concepts were created when deemed necessary)	Synthesis p. 10
19	Derivation of themes	Explain whether the process of deriving the themes or constructs was inductive or deductive	Synthesis p. 10
20	Quotations	Provide quotations from the primary studies to illustrate themes/constructs, and identify whether the quotations were participant quotations of the author's interpretation	Online appendix 8
21	Synthesis output	Present rich, compelling and useful results that go beyond a summary of the primary studies (e.g. new interpretation, models of evidence, conceptual models, analytical framework, development of a new theory or construct)	Synthesis of research findings p. 14-15<br>Online appendixes 6, 7<br>Table 2

For peer review only

BMJ Open

IDENTIFICATION AND INITIAL RESPONSE TO CHILDREN'S EXPOSURE TO INTIMATE PARTNER VIOLENCE: A QUALITATIVE SYNTHESIS OF THE PERSPECTIVES OF CHILDREN, MOTHERS AND PROFESSIONALS

Journal:	*BMJ Open*
Manuscript ID	bmjopen-2017-019761.R1
Article Type:	Research
Date Submitted by the Author:	12-Jan-2018
Complete List of Authors:	Lewis, Natalia; University of Bristol, Centre for Academic Primary Care, Population Health Sciences, Bristol Medical School; NIHR CLAHRC North Thames, Queen Mary University of London, Centre for Primary Care and Public Health<br>Feder, Gene; University of Bristol, Centre for Academic Primary Care, Population Health Sciences, Bristol Medical School<br>Howarth, Emma; University of Cambridge, NIHR Collaboration for Leadership in Applied Health Research & Care (CLAHRC) East of England<br>Szilassy, Eszter; University of Bristol, Centre for Academic Primary Care, Population Health Sciences, Bristol Medical School<br>McTavish, Jill; McMaster University, Department of Psychiatry and Behavioural Neurosciences<br>MacMillan, Harriet; McMaster University, Departments of Psychiatry and Behavioural Neurosciences, and of Pediatrics<br>Wathen, Nadine; Western University, Faculty of Information and Media Studies, and Centre for Research & Education on Violence Against Women & Children
**Primary Subject Heading**:	Public health
Secondary Subject Heading:	Health services research, Qualitative research
Keywords:	Child protection < PAEDIATRICS, PUBLIC HEALTH, QUALITATIVE RESEARCH, SOCIAL MEDICINE

SCHOLARONE™
Manuscripts

IDENTIFICATION AND INITIAL RESPONSE TO CHILDREN'S EXPOSURE TO INTIMATE PARTNER VIOLENCE: A QUALITATIVE SYNTHESIS OF THE PERSPECTIVES OF CHILDREN, MOTHERS AND PROFESSIONALS

Corresponding author: Natalia V. Lewis, Canynge Hall, 39 Whatley Road, Bristol, BS8 2PS, UK; (44)1179287246, Nat.Lewis@bristol.ac.uk

Natalia V. Lewis, Centre for Academic Primary Care, Population Health Sciences, Bristol Medical School, University of Bristol, Bristol, UK and Centre for Primary Care and Public Health, Blizard Institute, Barts and the London School of Medicine and Dentistry, Queen Mary University of London, UK

Gene S. Feder, Centre for Academic Primary Care, Population Health Sciences, Bristol Medical School, University of Bristol, Bristol, UK

Emma Howarth, National Institute for Health Research Collaboration for Leadership in Applied Health Research and Care East of England, Cambridge Institute of Public Health, University of Cambridge, Cambridge, UK

Eszter Szilassy, Centre for Academic Primary Care, Population Health Sciences, Bristol Medical School, University of Bristol, UK

Jill R. McTavish, Department of Psychiatry and Behavioural Neurosciences, McMaster University, Hamilton, ON, Canada

Harriet L. MacMillan, Departments of Psychiatry and Behavioural Neurosciences, and of Pediatrics, McMaster University, Hamilton, ON, Canada

Nadine Wathen, Faculty of Information and Media Studies, and Centre for Research & Education on Violence Against Women & Children, Western University, London, ON, Canada

Word count: 4740 (excluding title page, abstract, tables, figures & references).

For peer review only

For peer review only - http://bmjopen.bmj.com/site/about/guidelines.xhtml

ABSTRACT

Objectives. To synthesise evidence on the acceptable identification and initial response to children's exposure to intimate partner violence (IPV) from the perspectives of providers and recipients of healthcare and social services.

Design. We conducted a thematic synthesis of qualitative research, appraised the included studies with the modified Critical Appraisal Skills Programme checklist and undertook a sensitivity analysis of the studies scored above 15.

Data sources. We searched eight electronic databases, checked references and citations, and contacted authors of the included studies.

Eligibility criteria. We included qualitative studies with children, parents and providers of healthcare or social services about their experiences of identification or initial responses to children's exposure to IPV. Papers that have not been peer-reviewed were excluded as well as non-English papers.

Results: Searches identified 2039 records; 11 studies met inclusion criteria. Integrated perspectives of 42 children, 212 mothers and 251 professionals showed that sufficient training and support for professionals, good patient-professional relationship and supportive environment for patient/clients need to be in place before enquiry/disclosure of children's exposure to IPV should occur. Providers and recipients of care favour a phased enquiry about IPV initiated by healthcare professionals, which focuses on "safety at home" and is integrated into the context of the consultation or visit. Participants agreed that an acceptable initial response prioritises child safety and includes emotional support, education about IPV and signposting to IPV services. Participants had conflicting perspectives on what constitutes acceptable engagement with children and management of safety. Sensitivity analysis produced similar results.

Conclusions: Healthcare and social service professionals should receive sufficient training and on-going individual and system-level support to provide acceptable identification of and initial response to children's exposure to IPV. Ideal identification and responses should use a phased approach to enquiry and WHO LIVES principles integrated into a trauma- and violence-informed model of care.

For peer review only - http://bmjopen.bmj.com/site/about/guidelines.xhtml

STRENGTHS AND LIMITATIONS OF THIS STUDY

- This is the first synthesis of qualitative studies focusing on the integrated perspectives of patients/clients and healthcare and social service professionals on the acceptable identification and initial response to children's exposure to IPV.
- We retrieved relevant studies through a comprehensive search strategy, including electronic searches, citation and reference checking and contacting experts.
- Involvement of two reviewers throughout screening, data abstraction and critical appraisal of each study ensured methodological rigour of this review. Reviewers' backgrounds in different disciplines broadened and enriched the interpretation of data.
- Thematic synthesis allowed us to: (i) integrate perspectives of all participant groups and generate new interpretations going beyond the findings from primary studies, (ii) identify gaps in evidence within and across participant groups; (ii) establish areas of conflicting perspectives which can be targeted in development of interventions and future research.
- Exclusion of non-English papers and non-peer reviewed reports could result in missing some relevant studies; methodological limitations of the included studies weakened the reliability and objectivity of the evidence.

For peer review only

For peer review only - http://bmjopen.bmj.com/site/about/guidelines.xhtml

INTRODUCTION

Intimate partner violence (IPV) is a violation of human rights and widespread public health problem that is associated with impairment throughout the lifespan. It is defined as any behaviour by a current or former intimate partner associated with physical, sexual or psychological harm, including acts of physical aggression, sexual coercion, psychological abuse and controlling behaviours.[1] Although IPV is experienced by both men and women, the morbidity and mortality related to IPV is highest among women.[2] IPV has detrimental effects on physical, mental and reproductive health of women and has a negative impact on their children.[3 4] Children's exposure to IPV can occur through direct involvement and witnessing or through indirect exposure (e.g., being aware of the violence between parents/caregivers, financial consequences, parenting affected by IPV).[5 6] The prevalence of witnessing a parent assaulting another parent in the United States is 5.8% in the past year and 25% over the life-time.[7] Children's exposure to IPV is strongly associated with a broad range of emotional and behavioural problems, including internalising and externalising symptoms, as well as increased risk-taking behaviour and academic problems. Furthermore, such exposure among children can lead to physical health consequences, including injuries and death, when physical violence between caregivers directly involves children.[8 9] Health care professionals (HCPs) and social service professionals (SSPs) have an important role in identifying and responding to adult patients/clients and their children exposed to IPV.[10-12]

Identification of children's exposure to IPV in healthcare or social service settings can occur when the abused parent or caregiver seeks help, when children undergo assessment for behavioural problems, or when other services notify healthcare and social service providers about IPV occurrence. The WHO IPV guidelines recommend a case-finding approach to identifying women exposed to IPV: healthcare providers asking those women who present with indicators or clinical associations of IPV about safety in their relationship and at home.[1] There is no equivalent guidance on effective and acceptable approaches to identifying and responding to children's exposure to IPV and limited evidence on which to base that guidance.[8]

This review is one of a series undertaken for the **V**iolence, **E**vidence, **G**uidance and **A**ction (VEGA) Project, informing pan-Canadian public health guidance on family violence.[13] The objectives of the present systematic review were to identify, appraise and synthesise research evidence on the acceptability of the identification and initial responses to children's exposure to IPV in healthcare and social service settings. The synthesis addressed the following research questions:

1. What approaches to identification of children's exposure to IPV are acceptable to children, non-abusing parents and professionals?

For peer review only - http://bmjopen.bmj.com/site/about/guidelines.xhtml

2. What initial responses to children identified as being exposed to IPV are acceptable to children, non-abusing parents and professionals?

METHODS

We focused on qualitative evidence because we wanted to understand how providers and recipients of care perceive approaches to identification and responses to children's exposure to IPV and why they find them acceptable or otherwise.[14] Qualitative research explores peoples' own experiences and perspectives through analysing textual or visual material obtained while talking to people or observing them.[15] This systematic review follows the Centre for Reviews and Dissemination (CRD)[16] and PRISMA[17] guidance and adheres to the ENTREQ checklist for reporting synthesis of qualitative research.[18]

Search methods

We aimed to retrieve relevant studies in the field through a comprehensive search and sampling strategy,[19] building on an earlier review by Howarth and colleagues in the IMPROVE 2013 evidence synthesis.[20] First, we retrieved full-text reports assessed in the IMPROVE synthesis. Second, we re-ran IMPROVE searches (28.04.2016) in eight medical, social science, social care and nursing databases (Ovid MEDLINE, PsychINFO, The Cochrane Library, Embase, Web of Science Social Sciences Citation Index, Web of Science Conference Proceedings Citation Index- Social Science & Humanities, Social care online, CINAHL on EBSCO). For details of the MEDLINE search strategy see online Supplementary file 1. Finally, the first reviewer completed forward and backward citation chaining of all included papers and emailed corresponding authors of the included papers (12.08.2016, 24.08.2016) to confirm peer-reviewed status of reports and signpost to additional relevant papers.

Studies selection

Inclusion criteria are summarised in Table 1.

Table 1. Inclusion criteria for selecting studies

**Category**	**Inclusion criteria**

For peer review only

Category	Inclusion criteria
Population (further called stakeholders)	• Providers of health care (health care professionals (HCPs)) or social services (social service professionals (SSPs)) OR<br>• Recipients of health care or social services (further called patients or clients respectively):<br>– Children (however defined in primary studies) who have been exposed to IPV OR<br>– Non-abusing parents of children who have been exposed to IPV
Intervention	• Identification of children' exposure to IPV by any methods(s) – screening, case-finding, notification by other services, self-disclosure OR<br>• Initial response to children's exposure to IPV that followed the identification and occurred before referral to another professional or service
Phenomena of interest	• Views of and direct experiences with identification and initial response to children's exposure to IPV
Types of studies	• Publication date: database inception to April 28th, 2016 AND<br>• English language AND<br>• Empirical qualitative (stand alone or components of mixed methods research) AND<br>• Qualitative methods for data collection and analysis (e.g. interviews, focus groups, observations) AND<br>• Verbatim quotations from participants AND<br>• Papers that have undergone formal peer-review

Note. Social services professionals cover a range of services provided to advance adult and child welfare including child protection services.

Multiple papers from the same study were included if they each reported new data relevant to the research questions. Exclusion of non-English papers and papers that have not been through the formal peer review system (e.g., books, conference papers, editorials, letters, general comment papers) was justified by limited resources and concerns about validity and reliability of non-peer-reviewed sources, respectively.

For peer review only - http://bmjopen.bmj.com/site/about/guidelines.xhtml

Two reviewers independently screened titles and abstracts of all references. The first reviewer screened all full text papers, the second reviewers screened a 10% subset, and disagreements (27%) were resolved through discussion and consensus.

Analysis

Quality appraisal. Two reviewers independently assessed each study for methodological validity using the Critical Appraisal Skills Programme Qualitative checklist[21] modified for the purpose of the VEGA Project (M-CASP, score range 0-20).[22] Disagreements not resolvable between reviewers were resolved by a third reviewer. We did not exclude studies on reporting quality;[23] we conducted a secondary sensitivity analysis by restricting the synthesis to studies in the top tertile of methodological quality (M-CASP score ≥15).[24]

Data extraction. We adapted a data extraction form from IMPROVE.[20] The first reviewer extracted study details; second reviewers checked the extracts. The papers were then treated as primary text transcripts. Where studies included varied participants, only data relevant to our inclusion criteria were considered. Two reviewers independently extracted raw qualitative data[25] relevant to the views and direct experience of identification and initial response to children's exposure to IPV from Results sections of the included papers. These data could be in the form of participants' quotes or authors' interpretations of participants' voices. For each study, the reviewer entered data extracts into the form separately for children, parents and professionals.

Synthesis. Our choice of synthesis method was guided by the practical aims of this review, the "thickness" of the data reported in the primary studies, and the expertise of the team.[26 27] We applied thematic synthesis[25 28] in three stages:

I. *Line-by-line coding* alongside data extraction. Two reviewers independently coded the data extracts for themes relevant to the acceptability of the identification approach and initial response to children's exposure to IPV[29] subsequently meeting to compare and combine their codes. The first reviewer produced a final table of codes with supporting verbatim text for each participant group in each study.
II. *Developing descriptive themes.* The first reviewer grouped the codes into themes and sub-themes with accompanying verbatim to capture consistency and range of views within each participant group and across the studies.[29] Second reviewers commented on the table leading to the final version.
III. *Generating analytical themes.* The first reviewer used the constant comparison method[30] to integrate perspectives across child, parent and professional groups. The integrated stakeholder

For peer review only - http://bmjopen.bmj.com/site/about/guidelines.xhtml

perspectives were categorised by level of agreement within and across the groups.[31] When perspectives on a theme were consistent, it was categorised as convergent. When stakeholders' views on a theme were consistent within groups, but differed between them, it was categorised as divergent. Finally, themes with wide variation, within and between the groups, were categorised as conflicting. This integration through categorisation produced interim analytical themes which were further refined in relation to the research questions. Our approach allowed us to: (i) integrate perspectives of all participant groups and generate new interpretations beyond findings from primary studies, (ii) identify gaps in evidence within and across participant groups; and (ii) establish areas of conflicting perspectives which can be targeted in future research and interventions. Throughout this stage, the first reviewer developed diagrams and tables with interim analytical themes which were refined during group discussions at three meetings with all second reviewers.

RESULTS

Sixteen papers reporting 11 studies were included (Figure 1); three were reported in multiple papers. Two papers were from a study on parents' experiences with Irish child protection services.[32 33] Two papers drew on a study of police and children's social services responses to IPV incidents where children were present or resided in the household.[34 35] Four papers were from the RESPONDS study on general practice clinicians' perspectives on child safeguarding in IPV cases.[36-39] All papers were published between 2008 and 2015.

Studies characteristics and methodological quality

The 11 included studies are summarised in Table 2; online Supplementary file 2 contains detailed studies characteristics. Ten studies were based in high-income countries: UK,[35 39 40] US,[41-43] Australia,[44 45] Ireland,[32] and Canada.[46] One study was based in a middle-income country, Brazil.[47]

Table 2. Summary characteristics of the 11 studies (16 papers) synthesised (in chronological order)

Study	Country	Topic	Methodology	Stakeholder group, study			
				Children	Parents	HCPs	SSPs
Buckley, 2008[32 33]	Ireland	Experiences of child protection services	Interviews		X		
Black,	US	Interventions	Interviews			X	X

For peer review only - http://bmjopen.bmj.com/site/about/guidelines.xhtml

Study	Country	Topic	Methodology	Stakeholder group, study			
				Children	Parents	HCPs	SSPs
2010[41]		for IPV					
Stanley, 2010[34 35]	UK	Police IPV notifications of children's social services	Interviews	X	X		X
Meyer, 2011[44]	Australia	Help-seeking of IPV victims with children	Interviews		X		
Randell, 2012[43]	US	IPV information in healthcare setting	Focus groups		X		
Davidov, 2012[42]	US	Mandatory reporting of children's exposure to IPV	Secondary analysis of interviews and focus groups		X	X	X
Angelo, 2013[47]	Brazil	Experiences of providing care to children exposed to IPV	Interviews			X	
Jenney, 2014[46]	Canada	Communication between providers and recipients of child protection service	Interviews and focus groups		X		X
Szilassy, 2015[36 -39]	UK	Experiences of responding to children's exposure to IPV	Interviews			X	
Clarke,	UK	Experiences of	Interviews,		X	X	X

For peer review only

Study	Country	Topic	Methodology	Stakeholder group, study			
				Children	Parents	HCPs	SSPs
2015[40]		responding to children's exposure to IPV	focus groups, observations				
Morris, 2015[45]	Australia	Safety and resilience of children exposed to IPV	Interviews and focus groups	X	X		

Note. US – United States. UK – United Kingdom. IPV – intimate partner violence. HCPs – health care professionals. SSPs – social service professionals.

Voices of children were reported in two studies,[35 45] parents in eight studies[32 35 40 42-46] and professionals in seven studies.[35 39-42 46 47] Overall, the studies involved 42 children and young people aged 8 to 24 (19 from IPV and social services, 23 from general practice), 220 parents (212 mothers), and 251 professionals (113 health care, 42 social services, and 96 mixed samples). All parents were IPV survivors. HCPs included physicians[37 41] and nurses[37 42 47] from primary and secondary health care. SSPs were drawn from children's social services,[35] child protection services[46] and unspecified settings.[40 41]

Of 11 studies, seven [30 39-44] scored ≥15 out of 20 on the M-CASP indicating their overall good methodological quality (Figure 2, online Supplementary file 3). The main shortcomings identified were in the M-CASP domains of reliability and objectivity. Thus the authors did not justify their choices of study design,[35 37 40 41 44 47] research methods,[35 37 40-43 46 47] participant selection[35 40 41 43 44 46 47] and recruitment.[33 35 37 40 42 43 47] Only two studies[41 45] described strategies for establishing neutrality.

Synthesis

Line-by-line coding and descriptive themes

Initial line-by-line coding generated 75 codes. We grouped them into 22 descriptive themes with 13 subthemes related to: (1) experiences of identifying children's exposure to IPV; (2) experiences of the initial response to children's exposure; (3) factors enabling identification and initial response to children's exposure ; (4) reasons for not identifying or disclosing children's exposure or not engaging with services; (5) psychological consequences of individuals' involvement in identification and initial

For peer review only - http://bmjopen.bmj.com/site/about/guidelines.xhtml

response to children's exposure; and (6) suggested training and resources (see online Supplementary file 4). All relevant parents' quotations were from mothers who have experienced IPV.

Analytical themes

Categorisation of participant views by agreement/disagreement within and across the three stakeholder groups showed converging, diverging and conflicting perspectives on satisfactory identification and responses to children's exposure to IPV. These perspectives were expressed in five interim analytical themes (see online Supplementary file 5) which were refined further, when possible resolving conflicting perspectives within and across the studies. Finally, we articulated the final analytical themes as descriptions of an ideal identification and initial response:

1. Precursors for acceptable identification and response,
2. Acceptable identification,
3. Acceptable initial response, and
4. Conflicting perspectives on engagement with children and management of safety (Table 3).

Not all participant groups contributed equally to the final analytical themes. Thus, professionals' perspectives were presented across 15/17 subthemes, while mothers informed 13/17 and children 9/17. Children's quotes were not available for most subthemes covering an acceptable initial response. Quotes from each stakeholder group supporting the final analytical themes are collated in the online Supplementary file 6.

I. Precursors for acceptable identification and response. This theme was developed from converging perspectives on facilitators and barriers to satisfactory identification and response. It captures areas of agreement within and between stakeholder groups on the enabling processes and conditions required before children's exposure to IPV is disclosed or discussed. Mothers and children know and trust professionals who are non-judgemental and non-threatening, have good communication skills, can ensure confidentiality, and can offer practical help.[32 35 37 41 42 44-46] Good communication and trusting patient-professional relationship make mothers and children feel comfortable and safe in discussing sensitive issues and to return for support, if needed.[32 45] Professionals match their approaches to the individual mother's readiness to disclose and engage with services and work with the mother towards increasing her readiness.[35 41 43 44 46] Culturally sensitive materials in different languages on IPV and children's exposure to IPV are displayed in healthcare settings to prepare patients for enquiry and provide information for those who are not ready to disclose and engage.[41 43]

For peer review only - http://bmjopen.bmj.com/site/about/guidelines.xhtml

Professionals receive sufficient training and guidance built upon local policies on children's exposure to IPV and inter-agency work with children's social services and IPV services.[37 41 42] Training fits into daily practice of HCPs and SSPs and prepares them to better communicate with children.[37] Training and guidance clarify: (i) what constitutes children's exposure to IPV, especially psychological and non-direct physical, (ii) what are professionals' roles and reporting duties, (iii) how and where to document children's exposure to IPV, and (iv) how and where to signpost mothers and children.[32 35-37 41-46] Training and guidance increase professional awareness, skills and confidence, which lead to more satisfactory identification and responses.

Mothers, children and professionals receive emotional support and help with managing the emotional burden of involvement in identifying and responding to IPV. For patients/clients, such support is provided through non-judgemental responses and confidentiality (or being clear when reports must be made).[32 34 35 43 45] For professionals, ongoing emotional support and supervision need to be arranged.[35 37 41 47] This helps to maintain well-being and mental health of patients and professionals and leads to increased satisfaction.

The acceptability of any work related to identification of and response to children's exposure to IPV can be undermined by systems-level constraints, such as high demand on services, lack of resources and system support, and poor inter-agency collaboration.[35 37 40 41] Therefore, the approaches to identification and responses must be aligned with the capabilities of under-resourced health care and social services.

II. Acceptable identification This theme was developed from converging perspectives on the acceptable approaches to identification. It captures areas of agreement within and between stakeholders on how professionals should ask about children's exposure to IPV. The enquiry is built upon a trusting patient-professional relationship, takes place in a safe and supportive environment and is integrated into the context of the consultation or visit.[37 41 42 45 47] Acceptable identification involves a trained and well-supported HCP who is non-judgemental and has good communication skills.[32 35 37 41 42 44-46] HCP uses a phased approach, i.e. starts from the presenting symptoms, then moves to general safety and well-being of the child and finally asks about feeling safe at home.[37 41 42 45 47]

III. Acceptable initial responses. This theme was developed from converging perspectives on what the acceptable initial response should include and how it should be delivered. It also captures conflicting professional perspectives on the focus of their responses, reported within and between three studies[35 37 41] and resolved though justifying that child safety is prioritised over mother's safety and confidentiality. Due to the overlap between IPV and children's exposure to IPV, most

For peer review only - http://bmjopen.bmj.com/site/about/guidelines.xhtml

HCPs first target their initial response to the mother-child dyad.[37 41] However, their focus shifts towards the child if they are at risk of harm.[35 37] Some professionals feel conflicted when prioritising the child's needs over the adult's and require assistance with managing the emotional burden.[41] Professionals educate the mother about the impact of IPV on children, as well as the roles and duties of different providers.[35 37 41-43 46] This helps to relieve mothers' fears and increase their readiness to engage with services. Importantly, the approach to mothers education should not jeopardise their safety (e.g., by sending materials home).[35] Mothers and children must also be given information about local IPV services.[35 37 46]

IV. Conflicting perspectives on engagement with children and management of safety. This theme captures divergent and conflicting perspectives on the appropriateness of engaging directly with children[35 37 40 45] and satisfactory management of safety.[35 37 40 45 46] The variations were reported within and between studies and remain un-resolved in our review. While most children and mothers are positive about professionals talking directly to children and addressing their individual needs, a few held the opposite view.[35 45] In contrast, most professionals do not feel competent in communicating directly with children and prefer to assess children's needs through a proxy adult,[37 39] while some report always engaging with children and some think they should engage more.[35 40] Furthermore, all stakeholder groups have different views on the appropriate age for talking directly to children about IPV.[37 39 45] For example, some mothers think that HCPs can talk to their children about IPV from the age of five, while some doubt the acceptability of such conversations even with adolescents.[45] Finally, children and HCPs do not agree on the appropriate age for seeing a doctor alone. Most children prefer their mother or trusted adult to be with them at the consultation and indicate that at least teenagers should be seen alone.[45] Similarly, most HCPs indicate they would not see children alone, with some suggesting that if they were to do so, they would need to be adolescents. A few HCPs suggest that they would seek a parent's and child's permission to see the child alone, but they do not elaborate on the practicalities of obtaining such permission.[37]

Next, mothers and SSPs have divergent and conflicting views on the management of child safety and both perceive current practices as unsatisfactory. First, safety is understood differently by mothers and professionals. Actions perceived by mothers as increasing child's safety (e.g., staying with perpetrator for financial reasons, not seeking help to prevent escalation of abuse) are seen by SSPs as increasing the risk for the child.[44 46] While most mothers think that involvement of children's social services increases the risk for the child through potential escalation of abuse and child removal, most SSPs believe that their involvement results in greater safety. Whereas most SSPs think that women and children are safer out of the abusive relationship and feel frustrated when women

For peer review only - http://bmjopen.bmj.com/site/about/guidelines.xhtml

do not follow their advice to leave, women do not feel safer after leaving the perpetrator because of potential escalation of abuse, child contact with perpetrator, and lack of support post-separation. However, mothers feel intimidated by SSPs and that they must adhere to their instructions to keep children in the home and minimise involvement of children's social services.[32 44 46]

Second, stakeholders' perspectives on acceptable risk assessment and safety planning vary and diverge. Children's preferences for the assessment of risk in all environments are represented by one boy's voice which does not match any of the professional or parent viewpoints.[45] Mothers report not being included in safety planning with SSPs and want their views to be considered.[46] Professionals have polarised perspectives on the acceptability of the current risk assessment and safety planning with most feeling dissatisfied with the inconsistent approaches and bureaucracy involved.[35 37 40 41 46] A few SSPs suggest engaging with mothers and children more without elaborating on how this should be done.[40] All stakeholders report feeling conflicted about the existing management of safety, but have to comply due to the imbalance of power and system requirements.[35 37 40 41 46]

For peer review only

For peer review only - http://bmjopen.bmj.com/site/about/guidelines.xhtml

Table 3. Final analytical themes and their definitions

Final analytical themes with subthemes	Definition	Stakeholder group, study		
		Children	Mothers	Professionals
**1. Precursors for acceptable identification and response**				
**1.1. Satisfying and sustainable relationship**	Patients know and trust professionals with whom they develop good long-term relationships. Trusting relationships enable patients to feel safe and comfortable to discuss sensitive issues.	[45]	[32 44-46]	[37 41 46]
**1.2. Desired professional attitudes and skills**	When interacting with patients/clients, professionals demonstrate non-judgemental, non-threatening attitudes, show respect, actively listen, validate patient's accounts, reassure confidentiality, and provide practical help.	[35 45]	[32 35 42 44-46]	[37 41 42]
**1.3. Considering mother's readiness**	Professionals acknowledge individual mothers' readiness to disclose IPV and engage with services, work towards increasing mothers' readiness and match their approaches to the stage of mothers' readiness.		[43 44]	[35 41 46]
**1.4. Patient materials**	Culturally sensitive materials on IPV and children's exposure to IPV in different languages are displayed in healthcare settings.		[43]	[41]
**1.5. Professional training**	Professionals receive adequate training on communication with children, indicators of children's exposure to IPV, especially psychological and non-direct physical IPV, professionals' role in identifying and responding, documenting and reporting, inter-agency work.	[35 45]	[32 35 43-46]	[35-37 41 42]
**1.6. Professional resources**	Professionals have clear guidance on local IPV resources, what constitutes children's exposure to IPV, what is reportable, and how to document children's exposure to IPV in a way that keeps the			[37 41 42]

For peer review only - http://bmjopen.bmj.com/site/about/guidelines.xhtml

Final analytical themes with subthemes	Definition	Stakeholder group, study		
		Children	Mothers	Professionals
	child safe and ensures the safety and confidentiality of the mother.			
**1.7. Professional supervision and support**	Professionals have skilled supervision and ongoing support for coping with psychological consequences of working with children and mothers exposed to IPV and preventing vicarious trauma.			[35 37 41 47]
**1.8. Addressing systems' barriers**	Professionals' work of identifying and responding to children's exposure to IPV fits into the organisational, local and national context of increased demands on health care and social services without commensurate resources.			[35 37 40 41]
**2. Acceptable identification**				
**2.1. Space and time**	It is ideal to give patients permission, space and time to discuss sensitive matters.	[45]	[45]	
**2.2. Vocabulary**	It is preferable for HCPs to phrase questions about children's exposure to IPV as a "safety-at-home" matter.	[45]	[45]	
**2.2. Phased approach**	When asking about children's exposure to IPV, it is ideal for HCPs to initiate the enquiry, adapt it to the context of the consultation, and use a phased approach – from presenting symptoms to general safety and well-being, then to safety at home.	[45]	[45]	[37 41 42 47]
**3. Acceptable initial response**				
**3.1. Shifting focus**	Professionals first focus their responses on the mother-child dyad and shift to the child if he/she is at risk of harm. Professionals need assistance with managing emotional burdens caused by the shift.			[35 37 41]
**3.2. Emotional support**	When responding to disclosure, it is ideal to provide children and parents with encouragement		[46]	[37 39 47]

For peer review only - http://bmjopen.bmj.com/site/about/guidelines.xhtml

Final analytical themes with subthemes	Definition	Stakeholder group, study		
		Children	Mothers	Professionals
	and emotional support.			
**3.3. Education**	It is acceptable to educate mothers about the impact of IPV on children, IPV dynamics, professionals' roles and duties in responding. However, education should not jeopardise patient safety (e.g., through sending materials home where the perpetrator can find them).		[43]	[35 37 41 42 46]
**3.4. Signposting**	It is acceptable for professionals to give children and mothers information about local IPV services.	[35]	[35]	[35 37 46]
**4. Conflicting perspectives on engagement with children and management of safety**				
**4.1. Engaging directly with children**	Stakeholders' perspectives on the acceptability of talking directly to children exposed to IPV and seeing them alone are conflicting. Children are absent in the patient-professional communication. Mothers and children want direct engagement with children. Professionals do not see children as patients on their own and feel ill equipped for communicating with children about IPV.	[34 35 45]	[45]	[35 37 40]
**4.2. Management of safety**	Stakeholder preferences regarding risk assessment and safety planning are conflicting. Mothers and children are absent in the management of safety and want to be involved. Professionals are not satisfied with current risk assessment and safety planning approaches and want them to change.	[45]	[46]	[35 37 40 41]

Note. IPV – intimate partner violence. HCPs – health care professionals. SSPs – social care professionals.

For peer review only - http://bmjopen.bmj.com/site/about/guidelines.xhtml

Sensitivity analysis

Exclusion of four studies scoring below 15 on the M-CASP in sensitivity analysis[35 40 41 47] did not change the final analytical themes.

DISCUSSION

Main findings

This synthesis included 11 qualitative studies with 513 providers and recipients of health care and social services. We identified enabling precursors, ideal approaches, and areas of disagreement among children, mothers and professionals regarding the acceptable identification and initial response to children's exposure to IPV. Enabling precursor processes were linked to patient/client-professional relationship building, creating a safe and supporting environment, and changing and matching responses according to individual mothers' readiness to disclose and engage with services. Enabling conditions included sufficient training and multiple language-versions materials and embedding the work of identifying and responding to children's exposure to IPV into the context of under-resourced services. Acceptable identification involved a phased approach to enquiry. An ideal initial response included emotional support, education about IPV and signposting to IPV services. Areas of disagreement were related to the acceptability of engaging directly with children and managing child's safety.

An important finding of this synthesis is that participants' views on many factors related to satisfactory identification and initial response to children's exposure to IPV are strikingly consistent. Our first theme suggests that mothers and children need to have a trusting relationship with a professional who demonstrates certain attitudes and skills before enquiry and identification occurs. Combined with the theme of a phased approach to enquiry, our findings support a case-finding approach to identifying IPV. Our third theme supports the acceptability of providing mothers and children with emotional support, education about IPV and signposting to local IPV services. These essential components of good clinical practice on identification and response to IPV are supported in the literature[2 24 48] and highlight the importance of safe environments and trusting relationships as conditions for recognition of and response to mothers' and children's exposure to IPV.[49] Furthermore, the acceptability of a case-finding approach is consistent with the approach outlined by WHO on identification of child maltreatment in the recent mhGAP Intervention Guide.[50] The ideal initial responses to children's exposure to IPV are in line with the WHO LIVES principles for IPV response (**L**isten, **I**nquire about needs and concerns, **V**alidate, **E**nhance safety, and **S**upport).[1]

For peer review only - http://bmjopen.bmj.com/site/about/guidelines.xhtml

Another notable finding is the role of the wider context. Although most of the included studies were based in high-income countries, all professionals described how their ability to identify and respond to children's exposure to IPV was heavily influenced by healthcare and social service systems constraints. Specifically, they felt they lacked time to engage with their patients about sensitive issues; they felt burdened by constant cuts and restructuring of health care and social services; and they expressed frustration about poor access to referral pathways. These systemic factors were compounded by poor communication and coordination across organisations and absence of a single referral pathway, consistent with findings in research on child protection services.[51 52] This highlights the importance of targeting interventions on identification and initial response to IPV at both individual and system levels,[53-55] with professional guidance adaptable to the changing landscape of local services.

Our findings about the emotional burden of IPV work suggest that identifying and responding to children's exposure to IPV can have negative psychological impact on both providers and recipients of care (e.g., disempowerment, compassion fatigue and vicarious trauma). The common causes of distress are patients'/clients' feelings of shame and guilt linked to the acknowledgement of IPV and disclosure, professionals' ambiguous feelings towards mothers who did not follow their advice, tensions when shifting the focus from mother-child dyad to the child, and frustration with system-level obstacles. These findings, which are consistent with previous research,[56-58] emphasise the importance of assisting both patients/clients and professionals with managing psychological symptoms and preventing vicarious trauma.[58-60] The results also supports a trauma- and violence-informed approach to care of adults and children exposed to violence.[59-62]

One of the most striking findings of this review is the gap between including children's own needs and preferences, and the lack of attention paid by professionals to children as patient/clients on their own. Although professionals recognised the importance of working with a mother-child dyad exposed to IPV, most did not perceive children as individual patients/clients and used a proxy adult to assess and respond to the child's needs. In contrast, mothers and children were in favour of HCPs talking directly to children, treating each one as an individual and addressing the child's needs directly. The invisibility of children's own views has been previously reported[63-67] and can be explained by ethical and methodological challenges of undertaking research with children,[68] rigid professional attitudes, and lack of knowledge/competence on communicating with children. However, the finding is still concerning, especially given growing recognition of children's rights[69] and agency.[70] The gap between patient and professional preferences for talking directly to children suggests that professionals need training and guidance on communicating with children about sensitive issues. With regards to children's exposure to IPV specifically, professionals need

For peer review only - http://bmjopen.bmj.com/site/about/guidelines.xhtml

help understanding safety requirements, such as asking children in a private, confidential environment about exposure to IPV.[50]

Patients/clients and professionals held conflicting views about the acceptable management of safety. Neither patients nor professionals were satisfied with current risk assessment and safety planning. Addressing the needs of mother and child involves aligning professionals' and mothers' diverging perspectives about safety and risk. While professionals may think that leaving an abusive partner is a pre-requisite for safety, this is the time when women are at greatest risk of homicide;[71] furthermore, women themselves know when it is safest to leave.[72] In our synthesis, professionals favoured targeting the mother-child dyad, but found it acceptable to switch the focus to the child when they are at risk of harm. This is consistent with literature which suggests a hierarchy of needs for children and caregivers, recognising the needs of both mother and child, but prioritising the child given children's inherent vulnerability.[73]

While not the focus of this synthesis, it is important to note that mandatory reporting of child maltreatment laws may complicate or intertwine with strategies for inquiring about exposure to IPV, as children's exposure to IPV is a reportable exposure in some jurisdictions.[74 75] We found that most HCPs were confused as to whether or not children's exposure to IPV was reportable in their jurisdiction. They felt anxiety about the reporting duties and thresholds. This finding is in line with the recent meta-synthesis on mandated reporters' experiences with reporting child maltreatment.[22] The authors offer recommendations for mitigating potential harms associated with the reporting processes. The strategies include disclosing reporting duties and the limits of confidentiality when providers start a relationship, consulting with child protection services in an anonymous manner when a provider is unsure if the suspected maltreatment is reportable, and – if the suspected maltreatment is reportable – discussing with the child/family how the provider will file a report (when it is safe for the child to do so) and likely child protection service responses to the report. Discussing reporting duties and limits of confidentiality should ideally occur before inquiry, to minimise feelings of betrayal that may emerge when a provider realises they must report. These recommended strategies are applicable to the reportable children's exposure to IPV.

Strengths and limitations

We used comprehensive strategies for retrieving papers, including systematic searches of bibliographic databases, citation searching, reference checking, and emailing topic experts. Involvement of two reviewers throughout screening, data abstraction and critical appraisal reduced potential bias. Reviewers' backgrounds in different disciplines broadened and enriched data interpretation. Bringing together perspectives of children, mothers and professionals gave the

For peer review only - http://bmjopen.bmj.com/site/about/guidelines.xhtml

recipients of care a voice equal to the providers. Our analytical themes are easily understood and can be used by practitioners and policy makers as targets for interventions on identification and response to children's exposure to IPV.

This review has several limitations. Only papers published in English were included due to limited resources. This limitation alongside the exclusion of books and conference abstracts could result in missing studies relevant to the review questions. However, our decision to focus on papers that have been peer-reviewed and potentially of better methodological quality, increases the robustness of our findings.

The evidence we produced should be interpreted with caution, taking into consideration the following limitations of the primary data. It is supported by only 11 studies from high- and middle-income countries, four of which had methodological shortcomings regarding reliability and nine were lacking objectivity. The selective nature of the stakeholders' sample makes our findings relevant to HCPs and children's social service providers, mothers who have experienced IPV and their children. All evidence supported by children's voices came from two studies. Although small, the sample of children covered varied ages and settings.

Future studies should explore children's own experiences of encounters with health care and social services around identification and initial responses to IPV. Children's voices will provide important information on their values and preferences. Future studies should also recruit fathers who have experienced IPV. Our fourth theme of conflicting perspectives should guide future research on the acceptable approaches to talking directly to children about IPV, assessing risk and undertaking risk assessment and safety planning.

Conclusion

The present analysis adds to the evidence base important, client-centred considerations drawn from qualitative research to enhance approaches to the identification of, and response to, children exposed to IPV, and their caregivers. Health care and social service professionals should receive sufficient training and on-going individual and system-level support to recognise and respond safely to children's exposure to IPV. Ideal identification and response should be based on the WHO LIVES principles,[12] integrated into a trauma- and violence-informed model of care.[76] In Canada, findings from the present analysis have been integrated into professional guidance and curriculum in the VEGA Project.[13]

For peer review only - http://bmjopen.bmj.com/site/about/guidelines.xhtml

FUNDING

This work was supported by the Public Health Agency of Canada, through funding to the VEGA Project (HLM, NW). HLM was supported by the Chedoke Health Chair in Child Psychiatry. NVL was also supported by the Avon Primary Care Research Collaborative and National Institute for Health Research (NIHR) Collaboration for Leadership in Applied Health Research and Care North Thames at Bart's Health NHS Trust (NIHR CLAHRC North Thames). The views expressed in this article are those of the author(s) and not necessarily those of the NHS, the NIHR, or the Department of Health and Social Care.

COMPETING INTERESTS

We have read and understood BMJ policy on declaration of interests and declare that we have no competing interests.

ACKNOWLEDGEMENTS

The authors thank Dr Lisa Arai for performing the electronic searches and for helping with title and abstract screening. The authors are grateful to Khabo Piggott and Eleanor Wright who volunteered to help with data extraction, coding and critical appraisal.

CONTRIBUTORS

HLM and NW conceived the idea. GSF, NVL, EH, ES designed the study. NVL identified additional studies, carried out title and abstract screening, full text screening, quality appraisal, data extraction, coding and synthesis. She reported the research and drafted the paper. EH, ES, GSF conducted double full text screening, data extraction, coding and quality appraisal. NVL, EH, ES, GSF, JRM contributed to the development of descriptive and analytical themes. All authors contributed to the interpretation of the data and five revisions of the paper.

DATA SHARING STATEMENT

Data extracted from each of the 16 papers are stored in Word files and are available from the corresponding author upon reasonable request. Raw syntheses of these data are stored in Excel and Word files and are available from the corresponding author upon reasonable request.

For peer review only - http://bmjopen.bmj.com/site/about/guidelines.xhtml

REFERENCES

1. WHO. Health care for women subjected to intimate partner violence or sexual violence. http://www.who.int/reproductivehealth/publications/violence/vaw-clinical-handbook/en/: WHO, 2014.
2. Feder G, MacMillan HL. Intimate partner violence. In: Goldman L, Schafer AI, eds. Goldman's Cecil Medicine (25th ed). Philadelphia, PA: Elsevier Saunders, 2015.
3. WHO. Global and regional estimates of violence against women: prevalence and health effects of intimate partner violence and nonpartner sexual violence. Italy: WHO, 2013:51.
4. García-Moreno C, Jansen H, Ellsberg M, Heise L, Watts C. WHO multi-country study on women's health and domestic violence against women : initial results on prevalence, health outcomes and women's responses. Geneva, Switzerland: WHO, 2005:118.
5. MacMillan HL, Wathen CN. Children's exposure to intimate partner violence. Child Adolesc Psychiatr Clin N Am 2014;**23**(2):295-308, viii-ix doi: 10.1016/j.chc.2013.12.008[published Online First: Epub Date]|.
6. Radford L, Hester M. *Mothering through domestic violence*. London ; Philadelphia: Jessica Kingsley Publishers, 2006.
7. Finkelhor D, Turner HA, Shattuck A, Hamby SL. Prevalence of Childhood Exposure to Violence, Crime, and Abuse: Results From the National Survey of Children's Exposure to Violence. JAMA Pediatr 2015;**169**(8):746-54 doi: 10.1001/jamapediatrics.2015.0676[published Online First: Epub Date]|.
8. McTavish JR, MacGregor JC, Wathen CN, MacMillan HL. Children's exposure to intimate partner violence: an overview. Int Rev Psychiatry 2016:1-15 doi: 10.1080/09540261.2016.1205001[published Online First: Epub Date]|.
9. Holden GW. Children exposed to domestic violence and child abuse: terminology and taxonomy. Clin Child Fam Psychol Rev 2003;**6**(3):151-60
10. Government HM. Working together to safeguard children. A guide to inter-agency working to safeguard and promote the welfare of children: HM Government, 2015:109.
11. NICE. Domestic violence and abuse: How social care, health services and those they work with can respond effectively. London: NICE, 2014.
12. WHO. Responding to Intimate Partner Violence and Sexual Violence Against Women: WHO Clinical and Policy Guidelines. Geneva: WHO, 2013.
13. PreVAiL. VEGA (Violence, Evidence, Guidance and Action) Project: a public health response to family violence. Secondary VEGA (Violence, Evidence, Guidance and Action) Project: a public health response to family violence. https://projectvega.ca/.
14. Green J, Thorogood N. *Qualitative methods for health research*. Third edition. ed. Los Angeles: SAGE, 2014.
15. Malterud K. Qualitative research: standards, challenges, and guidelines. Lancet 2001;**358**(9280):483-8 doi: 10.1016/S0140-6736(01)05627-6[published Online First: Epub Date]|.
16. CRD. Systematic reviews: CRD's guidance for undertaking reviews in health care. In: Dissemination CfRa, ed. Centre for Reviews and Dissemination: University of York, 2009:294.
17. Liberati A, Altman DG, Tetzlaff J, et al. The PRISMA statement for reporting systematic reviews and meta-analyses of studies that evaluate health care interventions: explanation and elaboration. J Clin Epidemiol 2009;**62**(10):e1-34 doi: 10.1016/j.jclinepi.2009.06.006[published Online First: Epub Date]|.
18. Tong A, Flemming K, McInnes E, Oliver S, Craig J. Enhancing transparency in reporting the synthesis of qualitative research: ENTREQ. BMC Med Res Methodol 2012;**12**:181 doi: 10.1186/1471-2288-12-181[published Online First: Epub Date]|.
19. Booth A. Searching for qualitative research for inclusion in systematic reviews: a structured methodological review. Syst Rev 2016;**5**(74)

For peer review only - http://bmjopen.bmj.com/site/about/guidelines.xhtml

20. Howarth E, Moore T, Welton N, et al. IMPRoving Outcomes for children exposed to domestic ViolencE (IMPROVE): an evidence synthesis. Public Health Res 2016;**4**(10)
21. CASP. Critical Appraisal Skills Programme. CASP Qualitative Research Checklist 31.05.13. Secondary Critical Appraisal Skills Programme. CASP Qualitative Research Checklist 31.05.13 2013. http://www.casp-uk.net/checklists.
22. McTavish J, Kimber M, Devries K, et al. Mandated reporters' experiences with reporting child maltreatment: a meta-synthesis of qualitative studies. BMJ Open 2017 Oct 16;**7**(10):e013942 doi: 10.1136/bmjopen-2016-013942[published Online First: Epub Date]|.
23. Carroll C, Booth A. Quality assessment of qualitative evidence for systematic review and synthesis: Is it meaningful, and if so, how should it be performed? Res Synth Methods 2015;**6**(2):149-54 doi: 10.1002/jrsm.1128[published Online First: Epub Date]|.
24. Feder GS, Hutson M, Ramsay J, Taket AR. Women exposed to intimate partner violence: expectations and experiences when they encounter health care professionals: a meta-analysis of qualitative studies. Arch Intern Med 2006;**166**(1):22-37 doi: 10.1001/archinte.166.1.22[published Online First: Epub Date]|.
25. Thomas J, Harden A. Methods for the thematic synthesis of qualitative research in systematic reviews. BMC Med Res Methodol 2008;**8**:45 doi: 10.1186/1471-2288-8-45[published Online First: Epub Date]|.
26. Hannes K, Lockwood C. *Synthesising qualitative research : choosing the right approach*. Chichester, West Sussex, UK: John Wiley & Sons, 2012.
27. Noyes J, Hannes K, Booth A, et al. Chapter 20: Qualitative research and Cochrane reviews. In: Higgins J, Green S, eds. Cochrane handbook for systematic reviews of interventions. Version 5.3.0 (updated October 2015): The Cochrane Collaboration, 2015.
28. Barnett-Page E, Thomas J. Methods for the synthesis of qualitative research: a critical review. BMC Med Res Methodol 2009;**9**:59 doi: 10.1186/1471-2288-9-59[published Online First: Epub Date]|.
29. Braun V, Clarke V. Using thematic analysis in psychology. Qualitative Research in Psychology 2006;**3**(2):77-101 doi: 10.1191/1478088706qp063oa[published Online First: Epub Date]|.
30. Boeije H. A purposeful approach to the constant comparative method in the analysis of qualitative interviews. Qual Quant 2002;**36**(4):391-409
31. Shim WS, Haight WL. Supporting battered women and their children: Perspectives of battered mothers and child welfare professionals. Child Youth Serv Rev 2006;**28**(6):620-37 doi: 10.1016/j.childyouth.2005.06.005[published Online First: Epub Date]|.
32. Buckley H, Whelan S, Carr N. 'Like waking up in a Franz Kafka novel': Service users' experiences of the child protection system when domestic violence and acrimonious separations are involved. Child Youth Serv Rev 2011;**33**(1):126-33
33. Buckley H, Whelan S, Carr N, Murphy C. Service users' perceptions of the Irish Child Protection System. The Stationery Office, Dublin: Office of the Minister for Children and Youth Affairs, 2008:96.
34. Stanley N, Miller P, Richardson Foster H. Engaging with children's and parents' perspectives on domestic violence. Child & Family Social Work 2012;**17**(2):192-201
35. Stanley N, Miller P, Richardson Foster H, Thompson G. Children and families experiencing domestic violence: Police and children's social services' responses. London: NSPCC University of Lancashire, 2010:268.
36. Drinkwater J, Stanley N, Szilassy E, Larkins C, Hester M, Feder G. Juggling confidentiality and safety: A qualitative study of how general practice clinicians document domestic violence in families with children. BJGP 2017 doi: 10.3399/bjgp17X689353[published Online First: Epub Date]|.
37. Szilassy E, Das J, Drinkwater J, et al. Researching Education to Strengthen Primary care ON Domestic violence & Safeguarding (RESPONDS). Final Report for the Department of Health, Policy Research Programme Project Bristol: University of Bristol, 2015.

For peer review only - http://bmjopen.bmj.com/site/about/guidelines.xhtml

38. Szilassy E, Drinkwater J, Hester M, et al. Making the links between domestic violence and child safeguarding: an evidence-based pilot training for general practice. Health Soc Care Community 2016 doi: 10.1111/hsc.12401[published Online First: Epub Date]|.
39. Larkins C, Drinkwater J, Hester M, Stanley N, Szilassy E, Feder G. General practice clinicians' perspectives on involving and supporting children and adult perpetrators in families experiencing domestic violence and abuse. Family practice 2015;**32**(6):701-05 doi: 10.1093/fampra/cmv070[published Online First: Epub Date]|.
40. Clarke A, Wydall S. From 'Rights to Action': practitioners' perceptions of the needs of children experiencing domestic violence. Child & Family Social Work 2015;**20**(2):181-90 doi: 10.1111/cfs.12066[published Online First: Epub Date]|.
41. Black S, Dempsey SH, Davis MB. Practitioner-recommended policies and procedures for children exposed to domestic violence. Health Promotion Practice 2010;**11**(6):900-7
42. Davidov DM, Jack SM, Frost SS, Coben JH. Mandatory reporting in the context of home visitation programs: intimate partner violence and children's exposure to intimate partner violence. Violence Against Women 2012;**18**(5):595-610
43. Randell KA, Bledsoe LK, Shroff PL, Pierce MC. Educational Interventions for Intimate Partner Violence Guidance From Survivors. Pediatric Emergency Care 2012;**28**(11):1190-96 doi: 10.1097/PEC.0b013e318271be7f[published Online First: Epub Date]|.
44. Meyer S. 'Acting in the children's best interest?': Examining victims' responses to intimate partner violence. Journal of Child and Family Studies 2011;**20**(4):436-43
45. Morris A. Safety and resiliency at home: Voices of children from a primary care population (Unpublished PhD thesis). The University of Melbourne, 2015.
46. Jenney A, Mishna F, Alaggia R, Scott K. Doing the right thing? (Re) Considering risk assessment and safety planning in child protection work with domestic violence cases. Child Youth Serv Rev 2014;**47**:92-101 doi: 10.1016/j.childyouth.2014.07.015[published Online First: Epub Date]|.
47. Angelo M, Isidoro Prado S, Cascaes Cruz Ai, Oliveira Ribeiro M. Nurses' experiences caring for child victims of domestic violence: a phenomenological analysis. Texto & Contexto Enfermagem 2013;**22**(3):585-92 8p
48. MacMillan HL, Fleming J, Jamieson E. Psychiatric assessment of children and adolescents. In: Goldbloom R, ed. Pediatric Clinical Skills. (4th ed). New York: Churchill Linvingstone, 2010:2062–14.
49. VEGA. Trauma- and violence-informed care. Briefing note. Secondary Trauma- and violence-informed care. Briefing note. 2016. http://projectvega.ca/wp-content/uploads/2016/10/VEGA-TVIC-Briefing-Note-2016.pdf.
50. WHO. Update of the Mental Health Gap Action Programme (mhGAP) guidelines for mental, neurological and substance use disorders. http://www.who.int/mental_health/mhgap/guideline_2015/en/: WHO, 2015:72.
51. Stanley N. *Children experiencing domestic violence: a research review*: Dartington: Research in practice, 2011.
52. Stanley N, Humphreys C. *Domestic violence and protecting children : new thinking and approaches*. London ; Philadelphia: Jessica Kingsley Publishers, 2015.
53. Turner W, Hester M, Broad J, et al. Interventions to Improve the Response of Professionals to Children Exposed to Domestic Violence and Abuse: A Systematic Review. Child Abuse Rev 2017;**26**(1):19-39 doi: 10.1002/car.2385[published Online First: Epub Date]|.
54. Zaher E, Keogh K, Ratnapalan S. Effect of domestic violence training: systematic review of randomized controlled trials. Can Fam Physician 2014;**60**(7):618-24, e340-7
55. Garcia-Moreno C, Hegarty K, d'Oliveira AF, Koziol-McLain J, Colombini M, Feder G. The health-systems response to violence against women. Lancet 2015;**385**(9977):1567-79 doi: 10.1016/S0140-6736(14)61837-7[published Online First: Epub Date]|.

For peer review only - http://bmjopen.bmj.com/site/about/guidelines.xhtml

56. Dmytryshyn AL, Jack SM, Ballantyne M, Wahoush O, MacMillan HL. Long-term home visiting with vulnerable young mothers: an interpretive description of the impact on public health nurses. Bmc Nurs 2015;**14**(12)
57. Conrad D, Kellar-Guenther Y. Compassion fatigue, burnout, and compassion satisfaction among Colorado child protection workers. Child Abuse Neglect 2006;**30**(10):1071-80 doi: 10.1016/j.chiabu.2006.03.009[published Online First: Epub Date]|.
58. Slattery SM, Goodman LA. Secondary traumatic stress among domestic violence advocates: workplace risk and protective factors. Violence Against Women 2009;**15**(11):1358-79 doi: 10.1177/1077801209347469[published Online First: Epub Date]|.
59. Munro E. Department for Education. The Munro review of child protection: Final Report. A child-centred system. The Stationery Office Limited, 2011:189.
60. Tehrani N. *Managing trauma in the workplace : supporting workers and organisations*. Hove, East Sussex ; New York: Routledge, 2011.
61. Phillips H, Lyon E, Fabri M, Warshaw C. Promising practices and model programs: trauma-informed approaches to working with survivors of domestic and sexual violence and other trauma http://www.nationalcenterdvtraumamh.org/publications-products/promising-practices-and-model-programs/: National Center on Domestic Violence, Trauma & Mental Health, 2015:39.
62. Ponic P, Varcoe C, Smutylo T. Trauma- (and violence-) informed approaches to supporting victims of violence: policy and practice considerations. http://www.justice.gc.ca/eng/rp-pr/cj-jp/victim/rd9-rr9/p2.html: Government of Canada, Department of Justice, 2016.
63. Hester M. Children protection and domestic violence: findings from a Rowntree/ NSPCC study. In: Hanmer J, Itzin C, Quaid S, Wigglesworth D, eds. Home thruth about domestic violence: feminist influences on policy and practice: a reader. London and New York: Routledge, 2000:96-112.
64. McGee C. *Childhood experiences of domestic violence*. London ; Philadelphia: Jessica Kingsley Publishers, 2000.
65. Mullender A. *Children's perspectives on domestic violence*. London ; Thousand Oaks, Calif.: SAGE, 2002.
66. Radford L, Aitken R, Miller P, Ellis J, Roberts J, Firkic A. Meeting the needs of children living with domestic violence in London. Research report. London: NSPCC and Refuge, 2011:258.
67. Overlien C. Children Exposed to Domestic Violence Conclusions from the Literature and Challenges Ahead. J Soc Work 2010;**10**(1):80-97 doi: 10.1177/1468017309350663[published Online First: Epub Date]|.
68. Cater A, Øverlien C. Children exposed to domestic violence: a discussion about research ethics and researchers' responsibilities. Nordic Social Work Research 2014

4(1):67-79
69. UN. Convention on the Rights of the Child. Treaty Series 20 November 1989; 1577. http://www.refworld.org/docid/3ae6b38f0.html (accessed 21 June 2017).
70. Mayall B. *Towards a sociology for childhood : thinking from children's lives*. Buckingham ; Philadelphia: Open University Press, 2002.
71. Garcia L, Soria C, Hurwitz EL. Homicides and intimate partner violence. Trauma, Vilence & Abuse 2007;**8**(4):370-83 doi: 10.1177/1524838007307294[published Online First: Epub Date]|.
72. Reisenhofer S, Taft A. Women's journey to safety – the transtheoretical model in clinical practice when working with women experiencing intimate partner violence: a scientific review and clinical guidance. Patient Education and Counseling 2013;**93**(3): 536-48 doi: 10.1016/j.pec.2013.08.004[published Online First: Epub Date]|.
73. Humphreys C. Problems in the system of mandatory reporting of children living with domestic violence. Journal of Family Studies 2008;**14**(2-3)
74. Dubowitz H. World perspectives on child abuse: International Society for the Prevention of Child Abuse and Neglect, 2014.

For peer review only - http://bmjopen.bmj.com/site/about/guidelines.xhtml

75. Mathews B. Mandatory reporting laws: their origin, nature, and development over time. In: Mathews B, Bross DC, eds. Mandatory reporting laws and the identification of severe child abuse and neglect. Dordrecht: Springer, 2015:3-25.
76. Varcoe C, Ford-Gilboe M, Smye V, Wathen C, Browne A. VEGA Briefing Note on Trauma- and Violence-Informed Care. 2016. (accessed 09.01.2018).

List of figures

Figure 1. Flow of studies through the review.

Figure 2. Methodological quality of the studies as assessed by the modified Critical Appraisal Skills Programme (M-CASP, score range 0-20). Studies scored >=15 are in the top tertile.

List of online Supplementary files

Supplementary file 1. Search strategy.

Supplementary file 2. Characteristics of the 11 studies (16 papers) synthesised (in chronological order).

Supplementary file 3. Quality appraisal for the included studies.

Supplementary file 4. Descriptive themes.

Supplementary file 5. Interim analytical themes and their definitions.

Supplementary file 6. Final analytical themes supported by raw qualitative data from included studies.

For peer review only - http://bmjopen.bmj.com/site/about/guidelines.xhtml

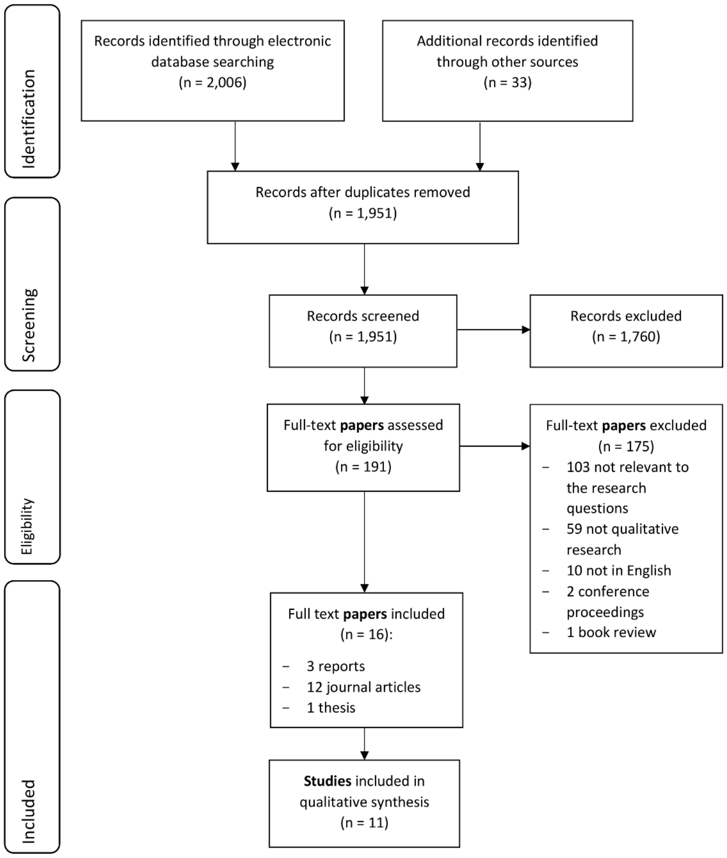

Figure 1. Flow of studies through the review.

210x297mm (300 x 300 DPI)

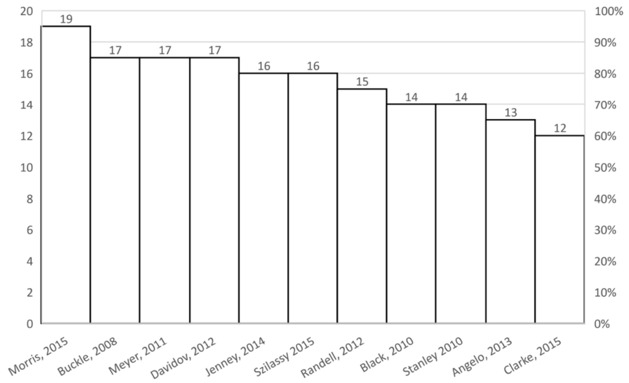

Figure 2. Methodological quality of the studies as assessed by the modified Critical Appraisal Skills Programme (M-CASP, score range 0-20).

Studies scored ≥15 are in the top tertial.

210x297mm (300 x 300 DPI)

Supplementary file 1

Search strategy

Full Boolean search strategy for MEDLINE

Date range of search 1 April 2013 to 28 April 2016.

Date of search 28 April 2016.

----

1 Child Welfare/ or Child, Preschool/ or Mother-Child Relations/ or Father-Child Relations/ or Child Behavior/ or "Child of Impaired Parents"/ or Child/ or Parent-Child Relations/ or Child Psychology/ or Child Reactive Disorders/ or Child Psychiatry/ or Adolescent Psychiatry/ or Adolescent Behavior/ or Adolescent/ or Adolescent Health Services/ or Adolescent Psychology/ or Adolescent Development/ (2612601)

2 (adolesc* or preadolesc* or pre-adolesc* or boy* or girl* or child* or infant* or preschool* or juvenil* or minors or school* or pediatri* or paediatri* or pubescen* or pre-pubescen* or puberty or student* or teen* or young or youth* or school* or high-school or "high school" or college or undergrad* or campus* or classroom*).mp. [mp=title, abstract, original title, name of substance word, subject heading word, keyword heading word, protocol supplementary concept word, rare disease supplementary concept word, unique identifier] (4168331)

3 1 or 2 (4168331)

4 Domestic Violence/ or Spouse Abuse/ or Battered Women/ (12097)

5 (abuse* adj3 (wom*n or partner or spous* or m*n or wife or wives or husband*)).tw. (5458)

6 (battered adj3 (wom*n or partner or spous* or m*n or wife or wives or husband*)).tw. (695)

7 (violen* adj3 (wom*n or partner or spous* or m*n or wife or wives or husband*)).tw. (7703)

8 (marital adj3 (violen* or abuse*)).tw. (297)

9 (famil* adj3 (violen* or abuse*)).tw. (2930)

10 domestic violen*.tw. (4517)

11 (intimate adj3 partner adj3 (violen* or abuse*)).tw. (4273)

12 (interparental adj3 (violen* or abuse*)).tw. (97)

For peer review only

13 (violen* adj2 (home*1 or household*)).tw. (206)

14 (parent* adj3 (violen* or abuse*)).tw. (1868)

15 or/4-14 (22293)

16 (expose* or exposure).mp. (927096)

17 witnes*.mp. (18418)

18 growing up.tw. (1791)

19 ((child* or adolesc*) adj3 "living with").tw. (1380)

20 16 or 17 or 18 or 19 (946981)

21 3 and 15 and 20 (2401)

22 limit 1 to ed=20130401-20160428 (284370)

23 21 and 22 (426)

For peer review only

Supplementary file 2

Characteristics of the 11 studies (16 papers) synthesised (in chronological order)

N	Study Paper(s) Country	Aim	Setting	Participants, n	Intervention(s)	Data collection	Data analysis	Primary themes relevant to the synthesis	M-CASP score/ Yes-No-Not sure
1	Buckley 2008[a]<br>Buckley 2008 (report) [33]<br>Buckley 2011 (article)<br>[32]<br>Republic of Ireland	Service users' experiences of child protection services	Women's refuges, advocacy groups, family support services, treatment services, counselling service, public redress bodies, community youth project, and statutory child protection service in 16 counties	27 parents<br>20 female, 7 male<br>18 survivors of IPV	Notification by other services<br>Self-disclosure<br>Self-referral<br>First contact and initial response from child protection services	Interviews	Grounded-theory	Positive and negative experiences with child protection services.<br>Impact of those experiences on parents and children.	17-3-0

For peer review only - http://bmjopen.bmj.com/site/about/guidelines.xhtml

N	Study Paper(s) Country	Aim	Setting	Participants, n	Intervention(s)	Data collection	Data analysis	Primary themes relevant to the synthesis	M-CASP score/ Yes-No-Not sure
2	Black 2010 [41], US	Recommended practices and obstacles to practices to improve interventions for families suffering from IPV	Varied health care services in one large urban area	Mixed sample of professionals including 24 HCPs and SSPs<br>20 female, 4 male	IPV screening<br>case finding of children's exposure to IPV<br>Initial care of the mother–child dyad	Interviews	Not stated	Individual, social, and system levels obstacles to identifying and responding to children's exposure to IPV.<br>Participants recommendations on how to improve identification and response to children's exposure to IPV.<br>Focus on supporting the mother–child dyad.<br>Challenges of focusing on the	14-6-0

For peer review only

N	Study<br>Paper(s)<br>Country	Aim	Setting	Participants, n	Intervention(s)	Data collection	Data analysis	Primary themes relevant to the synthesis	M-CASP score/ Yes-No-Not sure
								mother-child dyad.	
3	Stanley 2010[a]<br><br>Stanley 2010 (report) [35]<br><br>Stanley 2012 (article) [34]<br><br>UK	Experiences and views of police notifications to children's social services of IPV incidents where children were present/resided in the household	IPV services for women, services for children affected by IPV, children's social services in two local authorities in the North and South of England	19 children: age 10-19; 8 male, 11 female; 16 white British, 1 white/ Asian, 1 white/ black Caribbean, 1 white/ black African.<br><br>11 parents survivors of IPV: 1 male, 10 female; mean age 38 (25-48); most black minority ethnic (BME), 4 white British.<br><br>25 SSPs: age 23-65; 4 male, 21 female; 14 front-line, 22 white British, 1 black British, 1 black African, 1 "other";	Notification by police<br><br>Response from children's social services to the police notifications	Focus groups; interviews	Grounded theory	Clients' positive and negative experiences with children's social services and health professionals when disclosing IPV.<br><br>Barriers and facilitators to disclosing and acknowledging IPV.<br><br>Individual, organisational and system level barriers to responding to the police	14-6-0

For peer review only

N	Study Paper(s) Country	Aim	Setting	Participants, n	Intervention(s)	Data collection	Data analysis	Primary themes relevant to the synthesis	M-CASP score/ Yes-No-Not sure
				job experience 1.5-30 years				notifications of children's exposure to IPV.	
4	Meyer 2011 [44] Australia	Help-seeking decisions of IPV victims with dependent children	IPV services, family counselling services in three areas in Southeast Queensland	29 parents survivors of IPV<br>All female<br>Age 21-62 (mean 38, SD 11.3).<br>21 Australian-born, 2 New Zealand-born, 3 UK-born, 1 South African, 1 Malaysian, 1 Filipino.<br>27 had children. 25 children had witnessed the abuse.<br>16 secondary education, 3 in tertiary education, 10 completed tertiary education.	Initial response from child protection services	Interviews	Grounded theory	Gap between client and professional understanding of the dynamics of IPV and response needed.<br>Parents' fear of harm and loss of custody.	17-3-0

For peer review only

N	Study Paper(s) Country	Aim	Setting	Participants, n	Intervention(s)	Data collection	Data analysis	Primary themes relevant to the synthesis	M-CASP score/ Yes-No-Not sure
				7 employed, 2 unemployed, 2 volunteers, 14 home duties, 3 students, 1 retired.<br>26 received government benefits.					
5	Randell 2012 [43]<br>US	Important information to communicate about IPV and how it should be presented in a pediatric emergency department	IPV service in a racially and ethnically mixed city	99 parents survivors of IPV<br>All female<br>36 White, 32 Hispanic, 22 African American, 5 other, 4 not reported.<br>52 high school, 21 degree, 26 not reported.<br>45 currently employed	Identification and response in health care setting	Focus groups	Grounded theory	Content and format of posters about children's exposure to IPV.	15-5-0

For peer review only

N	Study Paper(s) Country	Aim	Setting	Participants, n	Intervention(s)	Data collection	Data analysis	Primary themes relevant to the synthesis	M-CASP score/ Yes-No-Not sure
6	Davidov 2012 [42] US	Issues related to the mandatory reporting of children's exposure to IPV	Community based Nurse-Family Partnership (NFP) programme in four sites	20 NFP mothers survivors of IPV; mean age 21.3; 50% White, 25% Black, 15% Hispanic; 90% single/ never married; years of school 11.7; 80% income<$24,000<br>47 HCPs:<br>25 NFP nurses; mean age 46.2; 92%>=bachelor's degree; nursing experience 19.7 (5-38); years in NFP 4.4.<br>22 community stakeholders including 4 from hospital or public health services; work	Mandatory reporting of children's exposure to IPV by NFP nurses	A secondary analysis of focus groups and interviews	Retrospective interpretation	Professional and patient uncertainty regarding reportable children's exposure to IPV.<br>Mothers' fears of losing custody of children.<br>Strategies nurses can use to ease mothers' fear.<br>Negative client experiences with child protection services.	17-3-0

For peer review only

N	Study Paper(s) Country	Aim	Setting	Participants, n	Intervention(s)	Data collection	Data analysis	Primary themes relevant to the synthesis	M-CASP score/ Yes-No-Not sure
				experience 9.2 years; mean age 50.5 years.					
7	Angelo 2013 [47] Brazil	Experience of the nurses in their care of child victims of IPV	Pediatric emergency, intensive care and inpatient units in São Paulo	15 HCPs<br>14 female, 1 male; ages 27-48, time since graduation 3-12 years	Case finding<br>Initial response	Interviews	Motivational theory	Identification of children's exposure to IPV through attention to signs and symptoms followed by verification.<br>Ambivalent feelings towards parents of children exposed to IPV.<br>Protective professional attitude towards children.	13-7-0
8	Jenney 2014 [46] Canada	How the voices of women with experience of	Child protection services in five Ontario child	17 CPS workers: 12 females, 5 males; job experience >6 years;	Notification by other services	Focus group; interviews	Grounded theory	The process of interaction between worker	16-4-0

For peer review only

N	Study<br>Paper(s)<br>Country	Aim	Setting	Participants, n	Intervention(s)	Data collection	Data analysis	Primary themes relevant to the synthesis	M-CASP score/ Yes-No-Not sure
		IPV are translated into child protection service workers' interventions	welfare agencies	qualifications - BA (3), BSW (8), CYW (1), and MSW (5); 9 did not have DVA training, 4 had training in both risk assessment and safety planning.<br>11 parent survivors of IPV: all female; age 26-49 years; 5 immigrant/refugee; 8 on < $25,000 annually; 5 College/University education, 2 high school, 4 grades 9–11.	Self-referral<br>Response from child protection services			and client during child protection service investigation.<br>Gap between client and professional understanding of the concept of safety and the impact of IPV on children.	
9	Interview study within the RESPONDS Project[a]	Responses to disclosure of IPV when children are exposed and the dilemmas	General practices in six sites across the north and south	69 HCPs<br>42 general practitioners, 12 practice nurses and	Case-finding<br>Notification by other services	Interviews	Thematic analysis using framework	Engaging directly with children at medical consultation.	16-4-0

For peer review only

N	Study Paper(s) Country	Aim	Setting	Participants, n	Intervention(s)	Data collection	Data analysis	Primary themes relevant to the synthesis	M-CASP score/ Yes-No-Not sure
	Szilassy 2015 (report) [37] Larkins 2015 (paper) [39] Szilassy 2016 (paper) [38] Drinkwater 2016 (paper) [36]<br><br>UK	and challenges general practice clinicians face when confronted with children's exposure to IPV	of England and the Midlands	15 practice managers<br>48 female, 21 male<br>Age: 21-34 – 10; 35-44 – 13; 45-54 – 30; 55-64 – 10; not known – 10<br>Experience managing domestic violence (number of cases): >5 – 5; a few – 14; 1 – 2, none – 26; none but aware of case at surgery - 7<br>IPV service provision: sparse – 28; established – 41<br>Location: metropolitan – 19; urban – 27; semi-rural – 23	Initial response from general practice clinicians			Professional uncertainty about responding to children's exposure to IPV.<br>Poor inter-agency work.	

For peer review only

N	Study Paper(s) Country	Aim	Setting	Participants, n	Intervention(s)	Data collection	Data analysis	Primary themes relevant to the synthesis	M-CASP score/ Yes-No-Not sure
				Region: north – 21; Midlands – 33; south – 33					
10	Clarke 2015 [40] UK	Practitioners' perceptions of children's experiences of IPV. Experiences of responding to the needs of children	Varied services in Wales	Mixed sample of 54 professionals from social services, health, education, police, probation, housing, IPV services and third sector organisations 5 parent survivors of IPV: all female	HCPs and SSPs responses to disclosure	Focus groups; interviews; case studies; meetings observations	Thematic analysis	Organisational and system level barriers to engaging directly with children and inter-agency work.	12-8-0
11	Morris 2015 (thesis) [45] Australia	Perspectives on children's safety and resilience when they live with IPV	General practices in Victoria	18 parent survivors of IPV: all female; mean age 43 (28-56) years; 2 born overseas. 23 children to 16 mothers survivors of IPV: mean age 14 (8-	Screening Case-finding Initial response from general practice clinicians	Interviews; focus groups	Hermeneutic Phenomenological analysis	Raising matters of safety or psychological well-being in the consultation. The type of relationships children and mothers expect	19-1-0

For peer review only

N	Study Paper(s) Country	Aim	Setting	Participants, n	Intervention(s)	Data collection	Data analysis	Primary themes relevant to the synthesis	M-CASP score/ Yes-No-Not sure
				24) years; 2 born overseas				to have with health professionals if they are to feel comfortable discussing family violence.<br>When, with whom and how safety conversations could be initiated.	

Note. a Indicates where more than one paper relates to a single study. IPV – intimate partner violence. HCPs – health care professionals. SSPs – social service professionals; NFP – Nurse-Family Partnership. CPS – child protection service; M-CASP – the modified version of the Critical Appraisal Skill Programme.[21 22] For studies reported in multiple papers, the total score was derived based on data extracted from all papers.

For peer review only

Supplementary file 3

Quality appraisal for the included studies (n = 11)

**Quality appraisal criteria**	**Yes**	**Studies**
A. Appropriateness of research methodology and design		
1. Does the research seek to interpret or illuminate the actions and/or subjective experiences of research participants?	11	[33 35 37 40-47]
2. Is qualitative research the right methodology for addressing the research goal?	11	[33 35 37 40-47]
3. Is the research design appropriate to address the aims of the research?	11	[33 35 37 40-47]
4. Has the researcher justified the research design	5	[33 42 43 45 46]
B. Ethical considerations		
5. Did the researcher use two or more of the following strategies to ensure ethical issues have been taken into consideration (is there are sufficient details of):<br>a. how the research was explained to participants for the reader to assess whether ethical standards were maintained;<br>b. did the researcher discuss issues raised by the study, such as issues around informed consent or confidentiality or how they have handled the effects of the study on the participants during and after the study;<br>c. was approval sought from an ethics committee)?	8	[33 35 37 41-45]
C. Credibility (akin to internal validity)<br>Do participants and those with similar experiences recognize the experiences contained with the study?		
6. Did the research use one or more of the following strategies to establish credibility:<br>a. has the researcher discussed saturation of data;<br>b. attempt to triangulate data by using different data collection methods;<br>c. member checking to see if participants agreed with the interpretations of the researcher;<br>d. peers or consultants experienced in qualitative research review their coding process;<br>e. full descriptions of member's words in their final paper?	11	[33 35 37 40-47]
D. Transferability (akin to external validity)		

For peer review only

Quality appraisal criteria	Yes	Studies
How does one determine the extent to which the findings of the study are applicability in other contexts or with other participant types?		
7. Did the researchers use any of the following strategies to establish transferability (use of dense description of the population studied through descriptions of demographics and geographic boundaries of the study? Note: the author must describe at least two specific sample descriptors (e.g. age range, gender, setting from which sample was selected, SES, etc.)	8	[33 35 37 42-44 46 47]
E. Consistency (akin to reliability)<br>Can another researcher follow the decision trail used by the researcher?		
8. Did the researcher use any of the following strategies to establish the purpose of the research:<br>a. was there a clear statement of aims of the research;<br>b. what was the goal of the research;<br>c. why was it thought important<br>d. its relevance?	11	[33 35 37 40-47]
9. Was the recruitment strategy appropriate to the aims of the research? (e.g. does the population from which the sample was selected resonate with the research objectives, was the sample selection ethically implemented).	10	[33 35 37 40-42 44-47]
10. Did the researcher explain how participants were selected?	9	[32 37 40 42-47]
11. Did they explain why the participants they selected were the most appropriate to provide access to the type of knowledge sought by the study	4	[32 37 42 45]
12. Were there any discussions around recruitment (e.g. why some people chose not to take part)?	4	[41 44-46]
13. Was the setting for data collection justified?	8	[35 37 40 42-46]
14. Is it clear how data were collected (e.g. focus group, semi-structured interview etc.)?	11	[33 35 37 40-47]
15. Did the researcher justify the methods chosen?	3	[33 44 45]
16. Did the researcher make the methods explicit (e.g. for interview method, is there an indication of how interviews were conducted, or did they use a topic guide)?	10	[33 35 37 41-47]
17. If the form of data is clear (e.g. tape recordings, video material, notes etc.)?	11	[33 35 37 40-47]
18. Did the researcher explain how the data were reduced or transformed for analysis?	11	[33 35 37 40-47]

For peer review only

Quality appraisal criteria	Yes	Studies
19. Did they discuss their interpretation and presentation of their findings?	11	[33 35 37 40-47]
F. Neutrality (akin to objectivity)<br>Did overall credibility, transferability, and consistency occur?		
20. Did the researcher use one or more of the following strategies to ensure neutrality (has the relationship between researcher and participants been adequately considered; has the researcher critically examined their own role, potential bias and influence during the formulation of the research questions or data collection, including sample recruitment and choice of location; did the researcher discuss how they responded to events during the study and whether they considered the implications of any changes in the research design; did the researcher employ field notes to record their personal reactions and biases after each interview/focus group; did they make a conscious effort to follow rather than lead the direction of interviews/focus groups)?	2	[41 45]

Note. Quality was appraised using the modified version of the Critical Appraisal Skill Programme (M-CASP), score range 0-20.[17]

For peer review only

Supplementary file 4

Descriptive themes

Descriptive theme with subthemes		Stakeholder group, study		
		Children	Parents	Professionals
**1. Experiences of identification of children’s exposure to IPV**				
1.1. Approach	1.1.1. How to ask	All children felt that HCPs should initiate a conversation about safety and well-being by giving permission, space and time to discuss sensitive matters.[45]	All mothers felt that HCPs should initiate a conversation about safety and well-being by giving permission, space and time to discuss sensitive matters.[45]	
	1.1.2. When to ask	Most children found it acceptable when HCP initiates enquiry about ‘safety at home’ from the presenting problem.[45]		Most HCPs described a positive experience of enquiring from the presenting symptoms of children’s exposure to IPV or following the disclosure of IPV by mother.[37 41 42 47]
		One young man preferred when HCP asks about ‘safety at home’ regardless of the presenting problem.[45]		Several practitioners suggested screening for IPV as part of routine pediatric exam.[41]
	1.1.4. Police notification to children’s social services of IPV incidents when		Most mothers were not aware of the police notification.[35]	Some SSPs from children’s social services thought that not all incidents of IPV with children present/ resided in the household need to be referred to

Descriptive theme with subthemes		Stakeholder group, study		
		Children	Parents	Professionals
	children were present/ resided in the household			them. However, they thought that they should be informed of such incidents.[35]
				Some SSPs from children's social services found police notifications inadequate due to the lack of details on children's exposure to IPV.[35]
				SSPs from children's social services found that "filtering out" police notifications to assess whether the family requires further intervention is too time- and labour-consuming.[35]
**2. Experiences of initial response to children's exposure to IPV**				
	2.1. Focus			Most HCPs felt that they should treat the mother-child dyad as a single entity where the needs of the mother were consistent with the child's health and safety needs.[37 41]
				Some HCPs felt conflicted when they had to prioritise the child's needs over mother's needs and confidentiality.[37 41]
				Some HCPs and most SSPs felt that if a child is at risk, they have to prioritise his/ her safety over the mothers' needs and confidentiality.[35 37]
	2.2. Emotional support		Mothers valued when SSPs provided a lot of encouragement.[46]	Some HCPs were positive about providing emotional support and 'leaving the door open' to

Descriptive theme with subthemes		Stakeholder group, study		
		Children	Parents	Professionals
				children to come back with any problems.[37 39 47]
	2.3. Education		Mothers valued information materials on the impact of IPV on children and IPV resources.[43]	HCPs and SSPs emphasised the importance of educating mothers about the impact of IPV on children and professional responsibilities in cases of children's exposure to IPV. Professionals used education to increase mother's engagement in services.[35 37 41 42 46]
				HCPs emphasised the importance of educating mothers about the purpose of the home visitation programme and children's social services. Professionals saw education as a pathway to easing mothers' fears of child removal as a result of professionals' involvement.[42]
				SSPs expressed mixed views on the acceptability of sending parents materials about impact of IPV on children in response to the police notification without any other intervention to families.[35]
2.4. Safety	2.4.1. Understanding of safety		Mothers thought that involvement of SSPs resulted in increased risk of abuse escalation and child removal.[32 44 46]	SSPs understood women's and children's safety as being out of the abusive relationship and believed that their involvement resulted in increased safety for both mothers and children.[44 46]

Descriptive theme with subthemes	Stakeholder group, study		
	Children	Parents	Professionals
		Some actions that mothers perceived as preventing abuse escalation and increasing child safety (e.g., staying with perpetrator, not seeking help) were seen by SSPs as increasing risk for children.[44 46]	Some actions that mothers perceived as increasing child safety were seen by SSPs as increasing risk for children.[44 46]
		Mothers did not feel safer after they complied with SSPs' requirements and left the abusive partner.[44 46]	
		Women wanted to talk with children's social workers about safety and safety planning.[46]	
2.4.2. Assessment of risk	One child suggested that HCPs need to ask children about their safety in all the environments that a child inhabits (e.g., all households post-separation, school).[45]		
			SSPs and HCPs favoured varied approaches to risk assessment (e.g., observation, information gathering from mother and other professionals, gut feeling, previous experiences).[35 37 46]

Descriptive theme with subthemes	Stakeholder group, study		
	Children	Parents	Professionals
			Some SSPs found standardised tools helpful for risk assessment (e.g., the Barnardo's Domestic Violence Risk toll).[35]
			Some SSPs saw the existing risk assessment as a bureaucratic exercise and believed that it does not measure real risk from the perpetrator[35 40 46]
2.4.3. Safety planning		Most mothers were not involved in safety planning with their SSPs therefore could not comment on its acceptability.[46]	SSPs found undertaking safety planning with both children and mothers to be acceptable.[35]
			Although SSPs understood that they could not change women, they believed that leaving an abusive partner is the most effective form of safety planning.[46]
			Some SSPs felt that they monitor the risk instead of doing direct safety planning with mothers and children and wanted more direct engagement with them.[35 40]
			Some HCPs found it acceptable to undertake safety planning with women who were not ready to leave the perpetrator.[41]

Descriptive theme with subthemes		Stakeholder group, study		
		Children	Parents	Professionals
	2.5. Signposting	Children appreciated when social workers signposted them to other services.[35]	Mothers appreciated when health visitors signposted them to other services.[35]	Two GPs felt comfortable when giving children information about services they could access.[37]
				Some HCPs and SSPs felt comfortable providing mothers with information about IPV resources. They saw signposting as a mechanism to facilitate engagement in services.[35 37 46]
	2.6. Documenting			Most HCPs reported uncertainty and confusion about recording policies and practices on children's exposure to IPV. The uncertainty was reported at local, region and national levels.[36 37 41 42]
				HCPs feared possible breach confidentiality of parents exposed to IPV by recording children's exposure in child or parent medical records.[36 37]
	2.7. Reporting			HCPs described uncertainty and confusion about their reporting responsibilities especially in cases of child exposure to psychological IPV and non-direct physical IPV.[37 41 42]

Descriptive theme with subthemes	Stakeholder group, study		
	Children	Parents	Professionals
		Mothers felt uncertain about reporting responsibilities of HCPs and feared that HCPs could initiate child removal.[42]	HCPs emphasised the importance of explaining their reporting responsibilities and procedures to mothers. Professionals use such education to ease mothers' fears of child removal, to increase engagement with services, and to justify breach of the mothers' confidentiality after IPV disclosure.[37 42]
**3. Factors that facilitated identification and initial response to children's exposure to IPV**			
3.1. Mother's readiness to disclose and engage		Mothers reported past experiences when they were not ready to acknowledge the impact of IPV on their children, disclose IPV and engage with services.[35 43 44]	HCPs and SSPs believed that the approach to identification and initial response should match mother's readiness to disclose IPV and engage with services.[35 41 46]
3.2. Patient-professional relationship	Most children emphasised the importance of 'knowing and being known' to the doctor, which made them feel comfortable to talk about personal issues.[45]	Most mothers emphasised the importance of a long-term trusting relationship with the same professional for IPV disclosure and engagement with services.[32 44-46]	Most HCPs and SSPs emphasised the importance of a long-term trusting relationship with mother for IPV disclosure and engagement with services.[41 46]
		Mothers suggested that they could be role models for their children in developing trust and building	Only one HCP suggested that existing relationships could enable proactive and direct engagement with children.[37 39]

Descriptive theme with subthemes		Stakeholder group, study		
		Children	Parents	Professionals
			relationship between child and doctor.[45]	
				Some HCPs thought that referral to children's social service can worsen their relationship with a parent who discloses IPV.[37 39 41]
				Some HCPs feared that professionals with strong relationships with their patients are more likely to develop vicarious trauma.[41]
	3.3. Positive professional attitude	Children described preferred professionals as good at listening, making them feel that they were welcome back, helping them practically.[34 35 45]	Mother appreciated HCPs and SSPs who took them seriously, validated their accounts, ensured confidentiality and helped practically.[32-35 42 44 46]	HCPs emphasised the importance of nonthreatening and non-judgemental attitudes with frequent reassurance of confidentiality when responding to IPV disclosure.[37 41 42]
3.4. Engaging directly with [illegible]	3.4.1. Talking to children at consultation	Children appreciated when professionals talk directly to them, instead of to their mothers.[34 35 45]	Mothers stressed the importance of giving each child a voice and addressing each child's needs at a medical consultation.[45]	Most HCPs expressed concerns in children's competence as informants and believed parents more than children.[37 39]
				Some HCPs expressed concern about their lack of competence in communicating directly with children.[37]

Descriptive theme with subthemes	Stakeholder group, study		
	Children	Parents	Professionals
			A few HCPs who directly engaged with children were female general practitioners in affluent areas with high levels of IPV service provision.[39]
			SSPs felt confident talking to children when assessing and intervening in families with IPV.[35]
			SSPs felt that they should listen to the child more when assessing and intervening in families with IPV.[40]
3.4.2. Appropriate age for talking to children at consultation	Most children regardless of age preferred their mother (trusted adult) to be present in a medical consultation.[45]	Mothers found it acceptable if doctors speak to children from the age of five. They believed that children exposed to IPV mature quicker than non-exposed children.[45]	There was no age above which clinicians consistently agreed it would be acceptable to talk to children about IPV; some were hesitant about even talking to teenagers.[37 39]
		Mothers preferred doctors to use non-verbal methods when communicating with young children.[45]	
		Mothers of adolescents believed that their children would find it difficult to talk to doctors because of teenage behavioural traits.[45]	

Descriptive theme with subthemes		Stakeholder group, study		
		Children	Parents	Professionals
	3.4.3. Seeing children alone	Some children felt that they should be of adolescent-age before speaking alone with the doctor about their safety and well-being.[45]		Most general practice clinicians felt that they should not see children alone at consultation.[37 39]
		Most children wanted their mothers (trusted adult) to be present at the medical consultation.[45]		Few general practice clinicians who would see children alone thought that it is acceptable to seek permission from the non-abusive parent and from the child without describing how it should be done.[37]
				SSPs regularly saw children alone when assessing risk, although they found it challenging to talk to children alone.[35]
**4. Reasons for not identifying/ disclosing children's exposure to IPV and not engaging with services**				
	4.1. Awareness of children's exposure to IPV		Mothers recognised that it took long time to acknowledge that IPV had an impact on their children.[32 35 43 44 46]	Most HCPs felt uncertain and confused about what constitutes children's exposure to IPV in cases of psychological IPV and non-direct physical IPV and how to respond to such cases.[37 42]
			Mothers felt that SSPs are unaware of the dynamics of IPV and risks involved.[32 34 35 44 46]	SSPs believed that mothers' underestimating of the impact of IPV on children was a barrier to disclosure and engagement with services.[34 35 46]

Descriptive theme with subthemes		Stakeholder group, study		
		Children	Parents	Professionals
4.2. Perceived role	4.2.1. Someone else's	Most children believed that HCPs' role is to treat illness and monitor their health and well-being, although health was narrowly defined as physical (e.g., injuries, vaccination).[45]	Mothers believed that HCPs' role is to treat illness and monitor their health and well-being, although health was narrowly defined as physical (e.g., injuries, vaccination).[45]	HCPs believed that Identifying and responding to children's exposure to IPV is someone else's role (e.g., health visitor, social worker, school nurse).[35 37]
		Two children recognised a potential role for HCPs in caring about children beyond physical illness.[45]		
				HCPs and SSPs believed that it is a mother's role to seek help and stop IPV.[35 37 40 42 44 46 47]
	4.2.2. Threatening and punitive	Children perceived children's social service's role as threatening and punitive.[34 35]	Mothers perceived children's social service's role as threatening and punitive.[34 35 42 46]	HCPs and SSPs perceived children's social service's role as threatening and punitive and recognised that mothers and children felt the same.[34 35 42]
				SSPs perceived health visitors as less threatening and more acceptable to families.[35]
	4.2.3. Uncertainty		Some mothers felt confused and uncertain about the role of children's social services which led to anxiety and avoidance of HCPs and SSPs.[34 35]	

Descriptive theme with subthemes	Stakeholder group, study		
	Children	Parents	Professionals
4.2.4. Mandatory reporting			HCPs felt confused and uncertain about their role as mandated reporters of children's exposure to IPV, especially in cases of psychological IPV and non-direct physical IPV.[42]
		Mothers perceived all HCPs as mandated reporters of children's exposure to IPV which led to anxiety and avoidance of HCPs.[42]	HCPs recognised that their role as mandated reporters prevented mothers from disclosing IPV and engaging with services.[42]
4.3. Fears	Children feared being removed from the home and taken into care.[34 35 40]	Mothers feared the removal of children by social services.[32-35 42 44 46]	HCPs and SSPs recognised that varied fears prevented mothers and children from disclosing IPV and engaging with services.[35 42 46]
	Children feared the negative consequences of IPV disclosure (e.g., escalation of abuse, involvement of children's social services).[40 45]	Mothers feared the negative consequences of IPV disclosure (e.g., escalation of abuse, involvement of police and children's social service).[44 45]	HCPs feared the negative consequences of identifying and responding to children's exposure to IPV (e.g., escalation of abuse, misunderstanding, involvement of children's social service, threats from parents, damage to the doctor-patient relationship, legal consequences).[35-37 39 41 47]
		Mothers feared HCPs because of their mandated reporter responsibilities.[42]	HCPs recognised that mothers feared them because of their mandated reporter responsibilities.[42]

Descriptive theme with subthemes		Stakeholder group, study		
		Children	Parents	Professionals
	4.4. Stigma	Children recognised the stigma, shame, and embarrassment associated with disclosing IPV.[34 35]	Mothers recognised the stigma, shame and embarrassment associated with disclosing IPV.[34 35 43 45]	
4.5. Influences from the wider environment	4.5.1 Social and cultural factors		Mothers who were non-native speakers expressed the need for information on children's exposure to IPV in their native language.[43]	HCPs and SSPs recognised that language and cultural norms and stereotypes make it difficult to engage with mothers from ethnic minorities.[35 41]
			Immigrant mothers wanted materials on children's exposure to IPV that address the immigration issues and culture-specific fears of IPV disclosure.[43]	
	4.5.2. Healthcare and social care system factors			HCPs and SSPs perceived the lack of contact time with patients/ clients as a major barrier to identifying and responding to children's exposure to IPV.[37 40 41]
				HCPs did not perceive children's exposure to IPV as their top priority.[37]
				HCPs and SSPs highlighted that their work with children was negatively affected by the cutbacks and constant reforms in home visitation services and social services.[35 37]

Descriptive theme with subthemes		Stakeholder group, study		
		Children	Parents	Professionals
				HCPs and SSPs highlighted the lack of services for all family members which prevented them from asking about and responding to children's exposure to IPV.[35 37 41]
		A few children suggested that police and social workers should communicate better in cases of IPV to ensure children's safety and adequate support.[34 35]		HCPs and SSPs were concerned with the lack of communication between the different providers and expressed a need for better communication and a more coordinated response to children's exposure to IPV.[35 37 40 41]
**5. Psychological consequences of identification and initial response to children's exposure to IPV**				
	5.1. Emotional burden	Children felt disappointed and powerless as a result of negative experiences with SSPs.[34 35]	Parents felt disappointed, stressed, hopeless and frustrated as a result of negative experiences with SSPs.[32-35 44]	
		Children thought that involvement with SSPs was 'stressful' for their mothers.[35]		
			Mothers felt embarrassed and guilty when acknowledging the impact of IPV on their children and when disclosing IPV to professionals.[34 35 43 45]	HCPs and SSPs felt frustrated and conflicted towards mothers who did not follow their advice.[41 46 47]

Descriptive theme with subthemes	Stakeholder group, study		
	Children	Parents	Professionals
			HCPs felt conflicted when changing the focus from the mother-child dyad to the individual child.[37 41]
			HCPs and SSPs felt uncomfortable about pressuring mothers to leave the perpetrator to protect children, but thought that they had few options in situations where mothers and children continued to live with the perpetrator.[37 41]
5.2. Vicarious trauma			HCPs feared the possibility of burnout and hardening towards women and children.[41 47]
	**6. Training and resources suggested**		
6.1. Professional training			HCPs preferred interactive training built upon real cases.[37 38]
			HCPs asked for training on communication with children.[37 38]
			HCPs and SSPs wanted to learn about other services' roles and responsibilities in responding to children's exposure to IPV.[35 37 38]
6.2. Information materials		Mothers favoured single posters in pediatric settings about signs of IPV, effect of IPV on children and local IPV services.[43]	HCPs found IPV posters in health care settings to be acceptable in facilitating IPV disclosure and self-referral to IPV services.[41]

Descriptive theme with subthemes	Stakeholder group, study		
	Children	Parents	Professionals
		Mothers who were non-native speakers favoured information materials in their native language.[43]	HCPs wanted local signposting materials on IPV services.[37 38]
		Mothers preferred information materials with positive non-judgemental message, representing ethnic and social diversity[43]	HCPs wanted materials on policies and procedures about mandatory reporting of children's exposure to IPV.[42]

Note. IPV – intimate partner violence. HCPs – health care professionals. SSPs – social service professionals….. – converging perspectives. …. – diverging perspectives. … - conflicting perspectives.

For peer review only

Supplementary file 5

Interim analytical themes and their definitions

Interim analytical theme with subthemes	Stakeholder group, study			
	Children	Mothers	HCPs	SSPs
**1. Converging perspectives on satisfactory strategies for identification and initial response to children's exposure to IPV**				
**1.1. Facilitators of acceptable identification and initial response**				
**1.1.1. Patient-professional relationship.** Some children, most mothers and most professionals highlighted that central to any work around children's exposure to IPV were aspects of the patient-professional relationship. When describing a good relationship, stakeholders talked about the long-term process of relationship building, continuity of contact, trust and effective communication.	[45]	[32 44-46]	[37 41]	[46]
**1.1.2. Positive professional attitudes and skills.** Patients/ clients and HCPs described how a non-judgemental non-threatening attitude, showing respect, validation of patients' accounts and reassurance of confidentiality facilitated disclosure and engagement with services.	[35 45]	[32 35 42 44 46]	[37 41 42]	
**1.1.3. Safety & comfort.** Good patient-professional relationships enabled children and mothers to feel safe and comfortable when discussing sensitive issues.	[45]	[45]		
**1.1.4. Individual mother's readiness.** Mothers and professionals suggested that the individual mother's readiness to disclose IPV and seek help should be considered when asking about IPV and responding to disclosure.		[43 44]	[41]	[35 46]
**1.1.5. Patient materials.** Mothers and HCPs thought that printed materials for patients about the effect of IPV on children and local IPV resources should be displayed in health-care settings to facilitate IPV disclosure by mothers and provide information for self-help.		[43]	[41]	

For peer review only - http://bmjopen.bmj.com/site/about/guidelines.xhtml

Interim analytical theme with subthemes	Stakeholder group, study			
	Children	Mothers	HCPs	SSPs
**1.2. Identification process**				
**1.2.1. Consultation atmosphere.** Children and mothers desired permission, space and time to discuss sensitive issues with HCPs.	[45]	[45]		
**1.2.2. Approach to enquiry.** Most children and HCPs preferred a case-based approach (from presenting symptoms).	[45]		[37 41 42 45 47]	
**1.2.3. Phased inquiry.** Patients and professionals were positive about HCPs initiating the enquiry. They preferred professionals to ask about children's exposure to IPV as a 'safety-at-home' issue – starting from the presenting symptoms, through questions about general well-being and the family situation to questions about feeling safe at home.	[45]	[45]	[37 41 42 47]	
**1.3. Initial response process**				
**1.3.1. Emotional support.** Mothers and professionals were positive about providing a lot of encouragement and emotional support to both children and mothers.		[46]	[37 47]	
**1.3.2. Patient/client education.** Mothers and professionals were positive about patient/client education about the impact of IPV on children, IPV dynamics, professional roles and duties. Professionals highlighted the importance of considering women's safety when sending letters about the impact of IPV on children.		[43]	[37 41 42]	[35 46]
**1.3.3. Signposting.** Mothers, children, and professionals were positive about signposting mothers and children to IPV services.	[35]	[35]	[37]	[35 46]
**2. Converging perspectives on barriers to satisfactory identification and initial response to children's exposure to IPV and proposed solutions**				
**2.1. Fears.** All stakeholder groups shared varied fears. The fear of abuse escalation, involvement of children's social services and child removal were mentioned by all groups. Additional professional fears of	[35 40 45]	[32 35 42 44 46]	[37 41 42 47]	[35 46]

For peer review only

Interim analytical theme with subthemes	Stakeholder group, study			
	Children	Mothers	HCPs	SSPs
negative consequences included threats from parents, negative impact on the therapeutic relationship with parents and legal consequences. Professionals used education to ease mothers' fears.				
**2.2. Gaps in knowledge and skills**				
**2.1.1. Low awareness.** All stakeholder groups demonstrated low awareness about children's exposure to IPV, especially psychological and non-direct physical IPV and delegated responsibilities of identifying and responding to somebody else.	[35 45]	[32 35 43-46]	[37 42]	[35 46]
**2.1.2. Communicating with children.** HCPs wanted to improve their communication skills in talking directly to children during medical consultations.			[37]	
**2.1.3. Documenting.** Professional described uncertainty and confusion regarding how and where to document children's exposure to IPV.			[36 41 42]	
**2.1.4. Mandatory reporting.** Mothers' uncertainty and confusion about HCPs' duties as mandated reporters mirrored professional uncertainty and confusion, especially in cases of children's exposure to psychological and non-direct physical IPV.		[42]	[37 41 42]	
**2.2. Emotional burdens.** All stakeholders were emotionally involved in the work of identifying and responding to IPV. Clients reported feeling threatened and pressured by SSPs to leave the perpetrator without adequate support provided. Professionals described psychological distress caused by ambivalent feelings towards mothers when dealing with children's exposure to IPV and were concerned that involvement in helping abused women and their children could cause vicarious trauma.	[35]	[32 35 46]	[37 41 47]	[35]
**2.3. System level factors**				
**2.3.1. Language and culture.** Stakeholders described mothers' immigration status, community norms and poor English as additional barriers to identifying and responding to children's exposure to IPV.		[43]	[41]	[35]

For peer review only

Interim analytical theme with subthemes	Stakeholder group, study			
	Children	Mothers	HCPs	SSPs
**2.3.2. Inter-agency work.** Stakeholders were similarly concerned with the lack of communication between different providers and expressed a need for a better communication, information sharing and a more coordinated approach when responding to children's exposure to IPV.	[35]		[37 41]	[35 40]
**2.3.3. Stretched services.** Professionals repeatedly mentioned: (i) lack of contact time with patients/clients, (ii) high demand and competing priorities, (iii) constant reforms to health care and social services, and (iv) shortage of services for all family members.			[37 41]	[35 40]
**3. Suggested training and resources** Professional training and resources. Professionals favoured a case-based training, wanted to improve their communication skills, learn about the roles of and responsibilities of other services and highlighted the importance of inter-agency collaboration. HCPs requested clear instructions on mandatory reporting and local signposting materials			[36 37 41 42]	[35]
**4. Diverging perspectives on satisfactory initial response**				
**4.1. Safety.** Mothers and professionals disagreed on the understanding of safety for the child. Actions perceived by mothers as increasing children's safety were seen by SSPs as jeopardising it. Most SSPs believed that their involvement increased children's safety and required mothers to leave the perpetrator. In contrast, most mothers thought that SSPs' involvement posed a threat to the child through leading to IPV escalation and child removal. Women did not feel safer after leaving the perpetrator because of potential escalation of abuse and child contact.		[32 44 46]		[44 46]
**4.2. Talking directly to children.** While most recipients of care were positive about professionals talking directly to children from a young age and addressing their individual needs, most HCPs did not see children as patients on their own and assessed children's needs through a proxy adult. Children and HCPs had	[34 35 45]	[45]	[37]	[35 40]

For peer review only

Interim analytical theme with subthemes	Stakeholder group, study			
	Children	Mothers	HCPs	SSPs
conflicting views about the appropriate age for talking to children about IPV and about appropriateness of seeing children alone.				
**5. Conflicting perspectives on satisfactory identification and initial response**				
**5.1 Shifting focus.** HCPs and SSPs had conflicting perspectives on the focus of their responses – child vs mother-child dyad/family. Although all agreed that the focus should switch to the child when he/she is at risk of harm, some felt conflicted when prioritising children's safety and needs over mother's' safety.			[37 41]	[35]
**5.2. Assessment of risk.** While children wanted to be involved in risk assessment and some SSPs found it acceptable to talk to children when assessing the risk, most professionals were not satisfied with the existing risk assessment process and saw it as a ticking box exercise.	[45]		[37]	[35 40 46]
**5.3. Safety planning.** While some HCPs found it acceptable to undertake safety planning with women who were not ready to leave the abusive partner, most SSPs saw leaving the abusive partner as the best safety planning for the child. SSPs had mixed views on the current safety planning process. Mothers wanted better communication with SSPs when undertaking safety planning.			[41]	[35 40 46]

Note. IPV – intimate partner violence. HCPs – health care professionals, SSPs – social service professionals.

For peer review only

Supplementary file 6

Final analytical themes supported by raw qualitative data from included studies

Theme	Subtheme	Participant quotation	Authors interpretation quotation
1. Precursors for acceptable identification and response to children's exposure to IPV	1.1. Satisfying and sustainable relationship	"Have confidence around them...I guess it depends how much you know your doctor. It's just depending on how well you know 'em. (Zoe, 12)"[45]<br><br>"Well in domestic violence I find that it's always the relationship that we have with the client, with the mom in this case — the stronger our relationship with the mom and the more we can understand her, understand her world through her eyes instead of imposing our view, we get them more on board. Once we can get that trust, then we can work together. [Worker #11]"[46]	"In common with participants in the wider study, they [parents] placed a lot of value on the type of relationship they could build up with workers [child protection workers] and the degree of trust that developed thereafter."[32]
	1.2. Desired professional attitudes and skills	"My doctor was really good, like once I went there and she was like really concerned and she sent me to someone else and then she told me to come back in a week so she could check on me and then, you know...like she kept making appointments so she could see me again and again. So it's good when doctors care like that...Makes you want to then	"Where service users reported positive experiences with the child protection services, it was normally when they felt they had been listened to fairly and believed. They also appreciated it when workers took direct action."[32]

For peer review only - http://bmjopen.bmj.com/site/about/guidelines.xhtml

Theme	Subtheme	Participant quotation	Authors interpretation quotation
		maybe go and um always go to them. (Claire, 21)"[45]<br><br>"I'm grateful that they actually heard me, heard my voice and listened to me (Client#11)"[46]	
	1.3. Considering mother's readiness	"[I] lost the children—my three youngest girls plus my son. Because the Department saw that I—which, looking back now, I understand what they're saying— that I put my children in a dangerous situation … they could very well have gotten hurt. But I don't think I deserved to lose them—I needed help to get out of that situation and I asked for help to get out of that situation."[44]	"It was suggested that a comprehensive system of support could be achieved through coordination of education, safety, and support services with the stage of change of the caregiver (P 1 and 14). Whereas victims in the early stages needed information, victims in later stages needed shelter, support groups, or legal services (P 11 and 16)."[41]
	1.4. Patient materials	"Doctors should ask about exposure. We can invite the conversation through literature and posters. Some believe that we should not ask. It is not the doctor's role. Some people [victims] are not ready to do anything. For those people, you just say, "Here is some info. (P 14)"[41]	"We found that women with histories of IPV support positive educational interventions in the PED [Pediatric Emergency Department] and recommend interventions that educate about resources, signs of IPV, and the effects of childhood IPV exposure in a non-judgmental and positive manner."[43]
	1.5. Professional training	"…other agencies, they don't actually understand what our role is, you know…what would be really, really good is people should shadow…and they should do some training on what the role, the roles	"Participants voiced a need to deconstruct the misperception that CEDV [children's exposure to domestic violence] is harmless and to educate

For peer review only

Theme	Subtheme	Participant quotation	Authors interpretation quotation
		of different agencies are and what different agencies can actually offer. (Child protection social worker 4)"[35]	professionals, health care providers, judges, police officers, and school staff on the hazards of CEDV (P 19)."[41]
	1.6. Professional resources	"I think you need to have in your literature about mandated reporting because, as nurses, we are mandated reporters and we need to know, again, with that role it needs to be spelled out very clearly because in a court of law that's what would be thrown at us, if we have fulfilled our responsibilities in what way. (Nurse)"[42]	
	1.7. Professional supervision	"[...] I don't feel much impact, because, it's like, we get so many [children], recently there have been a lot, and so before we used to get more [impacted], but these days it is hard to go through one month in which you don't have one or two cases of poor treatment, sexual abuse [...] I have already had so many [cases] that we are beginning to become accustomed with the situation [...] (N15)."[47]	"Conversely, providers with strong relationships with their patients may be more susceptible to frustration, burnout, or hardening toward victims who are not ready to leave abusive relationships." (P 19).[41]
	1.8. Addressing systems' barriers	"I know people can be very protective about information sharing. 'Well *you're* working with the adult' and '*You're* working with the child', so there is	"Some of the reasons given for not exploring the possibility of DVA [domestic violence and abuse] when child safeguarding concerns arose included DVA not being 'first on your radar or list of things to

Theme	Subtheme	Participant quotation	Authors interpretation quotation
		not always that joined-up thinking about risks as a whole. (Specialist Social Worker: 1)"[40]	ask about' (GP31) and the problem of 'finding the time to do [it] all' (GP28)."[37]
2. Acceptable identification	2.1. Space and time	"It makes it a lot easier when they say that you can take your time. Like even for the first session…you don't have to do anything, you can just try and get used to being there and stuff. (Amelia, 15)"[45]<br><br>"Five years, same woman, all same—then I start to tell them everything then they said why you didn't tell me before? Then I tell them you know I was so scared that time like I feel safe with them to talk about everything then my children aid worker she find out shelter for me. [Client #9]"[46]<br><br>"The sense that it's okay to come and talk to you about anything that worries them. (GP21)"[37]	
	2.2. Vocabulary	"Ask the kids if they're OK…(Lisa, Claire's mother) Yeah, I mean that's exactly right (agreeing with Lisa in the focus group).<br>(Doctor) 'Are_you_OK?' (Patient) 'Oh no'. (Doctor) 'Why? Would you like to talk about it?' I think, and they're simple little words…(Penelope, mother)"[45]	

For peer review only

Theme	Subtheme	Participant quotation	Authors interpretation quotation
	2.3. Phased approach	"I brought up the fact that I wasn't sleeping like normal people and that it was affecting my school work because I really, really want to do well at school. So he kind of asked a few questions and stuff to find out what else is affecting me… (Amelia, 15)"[45]<br><br>"But there might be other things like sleeping issues, so then the doctor might go, 'OK, so why aren't you sleeping, do you think?' And then that might lead to the child opening up, 'Well this is happening and…Mum and Dad are fighting all the time and…(Lisa, mother)"[45]<br><br>"If they start talking about domestic violence then at that point engage them in the discussion about, "Well, do you realize that that is a form of child abuse? Not that I'm going to report you today or anything but I want you to be aware of that. (Community stakeholder)"[42]	
3. Acceptable initial response	3.1. Shifting focus	"We are child focused so this is what you need to do to keep your children safe, but obviously you need to be safe to do that, so it sort of interlinks, but	"Throughout the interviews, it became apparent that although the project focused on children, practitioners treated the mother–child dyad as a

For peer review only

Theme	Subtheme	Participant quotation	Authors interpretation quotation
		certainly the children are our first concern. I mean, you know, if the victim is unable to protect their child, then we need to look at whether there is more action that we need to do, something a bit sort of formal as opposed to giving advice. (Initial assessment manager 4)"[35]<br><br>"I'd have to tell her that, because of the children, I would basically be needing to involve child protection services. I would reassure and inform her as much as possible as to what that would involve, but yes, I'd have to make her aware that, you know, it wasn't actually up to her whether I, because I often get into arguments about 'oh no, but they'll take the children... (GP30)"[37]	single entity where the needs of the mother were consistent with the child's health and safety needs."[41]
	3.2. Emotional support	"But, within my capabilities, what I did was intensify surveillance, and try my best to help the child emotionally [...] (N4)."[47]<br><br>"she used to give me a lot of encouragement, tell me I was doing a good job in spite of you know all the circumstances?" "just thinking of her words does	

For peer review only

Theme	Subtheme	Participant quotation	Authors interpretation quotation
		help you know, when I'm being a bit hard on myself as a mom (Client #8)"[46]	
	3.3. Education	"Initial care of the mother–child consisted of gathering information, helping the mother to view the relationship honestly and objectively, educating the mother on potentially dangerous situations and ways to avoid or prevent abuse. Documenting provider efforts in the patient chart facilitated follow-up. (P 15)"[41]<br><br>"There's already a lot of anxiety if you know talk about Children Services. . . . Hopefully [CPS] could be seen as more of a positive support for this mom and the children. . . . One thing is just to educate and talk to moms about what role Children Services can play, and seeing it more as a positive not just a negative experience. . . .Educate that, you know, Children Services is here to help, Children's Services isn't here to remove your kids. (Community stakeholder)"[42]	
	3.4. Signposting	"She was really helpful, …, she was the one that gave us the number for the NSPCC. She was just good at	"Two examples were given of GPs overcoming the lack of time and giving young people information

For peer review only

Theme	Subtheme	Participant quotation	Authors interpretation quotation
		listening to us and that. (Nicola, Young People's Focus Group 1)" [35]	directly about relevant services they could access."[37]
4. Conflicting perspectives on acceptable identification and response	4.1. Engaging directly with children	"It depends on how they (doctors) approach them. And you've got to treat them as an individual, not as a (sibling) group." (Focus group participants all agree).(Charlie, mother)"[45]<br><br>". . . for me it's opening the opportunities for children to speak. Because key to it is understanding the children's view of it. The parents can say, 'Oh I love him, I'll never do it again'. . . . What's happening for the kid within all this? (Senior Social Worker: 2)"[40]<br><br>"When you complete initial assessments you do see the children alone and often they are quite young, and direct questions are very difficult. You are only in the house for an hour, an hour and a half doing your initial assessment. They usually know why this lady is coming to the house and they have been told not to say anything. You always ask if there is anything they would like to talk about, how are things at home, you know, what happens if they are naughty, do they go	"Lisa and Penelope suggest five as a suitable age to engage directly with children about their safety based on the level of awareness that their own children had at that age."[45]<br><br>"Whilst not precluding the doctor from addressing a child directly in a consultation where their mother is present, all the children and young people suggested an older age range [15-18] for being seen on their own."[45]<br><br>"Few clinicians would routinely seek to directly engage with the children concerned. Some might in some circumstance, after undertaking other actions such as talking to others (e.g. health visitors); or if an opportunity arose or excuse could be made. More than half would not seek to engage directly with children."[37]

For peer review only

Theme	Subtheme	Participant quotation	Authors interpretation quotation
		on the naughty stair, or, you know, but it's very difficult. (Initial assessment worker 3)"[35]	
	4.2. Management of safety	"AM: And in what way can doctors and nurses help families?<br>By checking if they're sick. Checking… if they're OK, feeling safe and all that.<br>AM: OK, what would be the best way that they could ask you if you were feeling OK?<br>Um…are you safe at home and at school and that…and at Dad's. (Fred, 9)"[45]<br><br>"We talk a lot about options if she is ready to leave [the abusive situation]. The options, if she is not ready to leave, a lot has to do with education, what are the dynamics of domestic violence, helping her to understand what is going on in her relationship, helping her to look at the cycle of violence, and to begin to recognize what is going on. A lot of what we do, if somebody is not ready to leave, has to do with safety planning and not safety planning about leaving, but safety planning while you are in the relationship. . . . If a violent incident happened, where can you tell the kids to go? Can the kids go	"Although formally asked about safety plans, many women either had not been involved in safety planning (or did not recall doing so) with their workers."[46]<br><br>"Similar to the victims who decided against leaving after approaching CPS [child protection service] for support, most victims who remained silent altogether believed they were acting in the best interest of their children at the time…. Some of the non-help-seeking decisions of victims are therefore well-informed decisions, often based on victims' individual risk assessment and their perception of how they can best ensure their children's safety at that particular point in time."[44]<br><br>"A common theme across the interviews with practitioners was the need for a shift towards more direct working with children and their parents. There was a widespread belief that current policy focused on monitoring risk, rather than addressing risk

For peer review only

Theme	Subtheme	Participant quotation	Authors interpretation quotation
		upstairs? Can you teach them to dial 911? Can you teach them to leave and go to a neighbor's house? Who can the kids talk to? How do you talk to your kids about what is going on? And then, how do you get yourself safe? Not staying in the kitchen, not being in the bathroom, not staying in a place where you don't have an exit, not wearing scarves around your neck because they can be used to choke you. There are lots and lots . . . and for every person the safety plan is going to look a little bit different. (P 2D)"[41]	through systematic safety planning with adult victims and direct safety planning with children."[40]

For peer review only

BMJ Open

IDENTIFICATION AND INITIAL RESPONSE TO CHILDREN'S EXPOSURE TO INTIMATE PARTNER VIOLENCE: A QUALITATIVE SYNTHESIS OF THE PERSPECTIVES OF CHILDREN, MOTHERS AND PROFESSIONALS

Journal:	*BMJ Open*
Manuscript ID	bmjopen-2017-019761.R2
Article Type:	Research
Date Submitted by the Author:	07-Mar-2018
Complete List of Authors:	Lewis, Natalia; University of Bristol, Centre for Academic Primary Care, Population Health Sciences, Bristol Medical School; Queen Mary University of London, Centre for Primary Care and Public Health, Blizard Institute, Barts and the London School of Medicine and Dentistry<br>Feder, Gene; University of Bristol, Centre for Academic Primary Care, Population Health Sciences, Bristol Medical School<br>Howarth, Emma; University of Cambridge, NIHR Collaboration for Leadership in Applied Health Research & Care (CLAHRC) East of England<br>Szilassy, Eszter; University of Bristol, Centre for Academic Primary Care, Population Health Sciences, Bristol Medical School<br>McTavish, Jill; McMaster University, Department of Psychiatry and Behavioural Neurosciences<br>MacMillan, Harriet; McMaster University, Departments of Psychiatry and Behavioural Neurosciences, and of Pediatrics<br>Wathen, Nadine; Western University, Faculty of Information and Media Studies, and Centre for Research & Education on Violence Against Women & Children
**Primary Subject Heading**:	Public health
Secondary Subject Heading:	Health services research, Qualitative research
Keywords:	Child protection < PAEDIATRICS, PUBLIC HEALTH, QUALITATIVE RESEARCH, SOCIAL MEDICINE

SCHOLARONE™
Manuscripts

IDENTIFICATION AND INITIAL RESPONSE TO CHILDREN'S EXPOSURE TO INTIMATE PARTNER VIOLENCE: A QUALITATIVE SYNTHESIS OF THE PERSPECTIVES OF CHILDREN, MOTHERS AND PROFESSIONALS

Corresponding author: Natalia V. Lewis, Canynge Hall, 39 Whatley Road, Bristol, BS8 2PS, UK; (44)1179287246, Nat.Lewis@bristol.ac.uk

Natalia V. Lewis, Centre for Academic Primary Care, Population Health Sciences, Bristol Medical School, University of Bristol, Bristol, UK and Centre for Primary Care and Public Health, Blizard Institute, Barts and the London School of Medicine and Dentistry, Queen Mary University of London, UK

Gene S. Feder, Centre for Academic Primary Care, Population Health Sciences, Bristol Medical School, University of Bristol, Bristol, UK

Emma Howarth, National Institute for Health Research Collaboration for Leadership in Applied Health Research and Care East of England, Cambridge Institute of Public Health, University of Cambridge, Cambridge, UK

Eszter Szilassy, Centre for Academic Primary Care, Population Health Sciences, Bristol Medical School, University of Bristol, UK

Jill R. McTavish, Department of Psychiatry and Behavioural Neurosciences, McMaster University, Hamilton, ON, Canada

Harriet L. MacMillan, Departments of Psychiatry and Behavioural Neurosciences, and of Pediatrics, McMaster University, Hamilton, ON, Canada

Nadine Wathen, Faculty of Information and Media Studies, and Centre for Research & Education on Violence Against Women & Children, Western University, London, ON, Canada

Word count: 4740 (excluding title page, abstract, tables, figures & references).

For peer review only

For peer review only - http://bmjopen.bmj.com/site/about/guidelines.xhtml

ABSTRACT

Objectives. To synthesise evidence on the acceptable identification and initial response to children's exposure to intimate partner violence (IPV) from the perspectives of providers and recipients of healthcare and social services.

Design. We conducted a thematic synthesis of qualitative research, appraised the included studies with the modified Critical Appraisal Skills Programme checklist and undertook a sensitivity analysis of the studies scored above 15.

Data sources. We searched eight electronic databases, checked references and citations, and contacted authors of the included studies.

Eligibility criteria. We included qualitative studies with children, parents and providers of healthcare or social services about their experiences of identification or initial responses to children's exposure to IPV. Papers that have not been peer-reviewed were excluded as well as non-English papers.

Results: Searches identified 2039 records; 11 studies met inclusion criteria. Integrated perspectives of 42 children, 212 mothers and 251 professionals showed that sufficient training and support for professionals, good patient-professional relationship and supportive environment for patient/clients need to be in place before enquiry/disclosure of children's exposure to IPV should occur. Providers and recipients of care favour a phased enquiry about IPV initiated by healthcare professionals, which focuses on "safety at home" and is integrated into the context of the consultation or visit. Participants agreed that an acceptable initial response prioritises child safety and includes emotional support, education about IPV and signposting to IPV services. Participants had conflicting perspectives on what constitutes acceptable engagement with children and management of safety. Sensitivity analysis produced similar results.

Conclusions: Healthcare and social service professionals should receive sufficient training and on-going individual and system-level support to provide acceptable identification of and initial response to children's exposure to IPV. Ideal identification and responses should use a phased approach to enquiry and WHO LIVES principles integrated into a trauma- and violence-informed model of care.

For peer review only - http://bmjopen.bmj.com/site/about/guidelines.xhtml

STRENGTHS AND LIMITATIONS OF THIS STUDY

- This is the first synthesis of qualitative studies focusing on the integrated perspectives of patients/clients and healthcare and social service professionals on the acceptable identification and initial response to children's exposure to IPV.
- We retrieved relevant studies through a comprehensive search strategy, including electronic searches, citation and reference checking and contacting experts.
- Involvement of two reviewers throughout screening, data abstraction and critical appraisal of each study ensured methodological rigour of this review. Reviewers' backgrounds in different disciplines broadened and enriched the interpretation of data.
- Thematic synthesis allowed us to: (i) integrate perspectives of all participant groups and generate new interpretations going beyond the findings from primary studies, (ii) identify gaps in evidence within and across participant groups; (ii) establish areas of conflicting perspectives which can be targeted in development of interventions and future research.
- Exclusion of non-English papers and non-peer reviewed reports could result in missing some relevant studies; methodological limitations of the included studies weakened the reliability and objectivity of the evidence.

For peer review only

For peer review only - http://bmjopen.bmj.com/site/about/guidelines.xhtml

INTRODUCTION

Intimate partner violence (IPV) is a violation of human rights and widespread public health problem that is associated with impairment throughout the lifespan. It is defined as any behaviour by a current or former intimate partner associated with physical, sexual or psychological harm, including acts of physical aggression, sexual coercion, psychological abuse and controlling behaviours.[1] Although IPV is experienced by both men and women, the morbidity and mortality related to IPV is highest among women.[2] IPV has detrimental effects on physical, mental and reproductive health of women and has a negative impact on their children.[3 4] Children's exposure to IPV can occur through direct involvement and witnessing or through indirect exposure (e.g., being aware of the violence between parents/caregivers, financial consequences, parenting affected by IPV).[5 6] The prevalence of witnessing a parent assaulting another parent in the United States is 5.8% in the past year and 25% over the life-time.[7] Children's exposure to IPV is strongly associated with a broad range of emotional and behavioural problems, including internalising and externalising symptoms, as well as increased risk-taking behaviour and academic problems. Furthermore, such exposure among children can lead to physical health consequences, including injuries and death, when physical violence between caregivers directly involves children.[8 9] Health care professionals (HCPs) and social service professionals (SSPs) have an important role in identifying and responding to adult patients/clients and their children exposed to IPV.[10-12]

Identification of children's exposure to IPV in healthcare or social service settings can occur when the abused parent or caregiver seeks help, when children undergo assessment for behavioural problems, or when other services notify healthcare and social service providers about IPV occurrence. The WHO IPV guidelines recommend a case-finding approach to identifying women exposed to IPV: healthcare providers asking those women who present with indicators or clinical associations of IPV about safety in their relationship and at home.[1] There is no equivalent guidance on effective and acceptable approaches to identifying and responding to children's exposure to IPV and limited evidence on which to base that guidance.[8]

This review is one of a series undertaken for the **V**iolence, **E**vidence, **G**uidance and **A**ction (VEGA) Project, informing pan-Canadian public health guidance on family violence.[13] The objectives of the present systematic review were to identify, appraise and synthesise research evidence on the acceptability of the identification and initial responses to children's exposure to IPV in healthcare and social service settings. The synthesis addressed the following research questions:

1. What approaches to identification of children's exposure to IPV are acceptable to children, non-abusing parents and professionals?

For peer review only - http://bmjopen.bmj.com/site/about/guidelines.xhtml

2. What initial responses to children identified as being exposed to IPV are acceptable to children, non-abusing parents and professionals?

METHODS

We focused on qualitative evidence because we wanted to understand how providers and recipients of care perceive approaches to identification and responses to children's exposure to IPV and why they find them acceptable or otherwise.[14]. Qualitative research explores peoples' own experiences and perspectives through analysing textual or visual material obtained while talking to people or observing them.[15] This systematic review follows the Centre for Reviews and Dissemination (CRD)[16] and PRISMA[17] guidance and adheres to the ENTREQ checklist for reporting synthesis of qualitative research.[18]

Search methods

We aimed to retrieve relevant studies in the field through a comprehensive search and sampling strategy,[19] building on an earlier review by Howarth and colleagues in the IMPROVE 2013 evidence synthesis.[20] First, we retrieved full-text reports assessed in the IMPROVE synthesis. Second, we re-ran IMPROVE searches (28.04.2016) in eight medical, social science, social care and nursing databases (see online Supplementary file 1). Finally, the first reviewer completed forward and backward citation chaining of all included papers and emailed corresponding authors of the included papers (12.08.2016, 24.08.2016) to confirm peer-reviewed status of reports and signpost to additional relevant papers.

Studies selection

Inclusion criteria are summarised in Table 1.

Table 1. Inclusion criteria for selecting studies

Category	Inclusion criteria
Population (further called stakeholders)	• Providers of health care (health care professionals (HCPs)) or social services (social service professionals (SSPs)) OR<br>• Recipients of health care or social services (further called patients or clients respectively):<br>– Children (however defined in primary studies) who have been exposed to IPV OR

For peer review only

Category	Inclusion criteria
	– Non-abusing parents of children who have been exposed to IPV
Intervention	• Identification of children' exposure to IPV by any methods(s) – screening, case-finding, notification by other services, self-disclosure OR<br>• Initial response to children's exposure to IPV that followed the identification and occurred before referral to another professional or service
Phenomena of interest	• Views of and direct experiences with identification and initial response to children's exposure to IPV
Types of studies	• Publication date: database inception to April 28th, 2016 AND<br>• English language AND<br>• Empirical qualitative (stand alone or components of mixed methods research) AND<br>• Qualitative methods for data collection and analysis (e.g. interviews, focus groups, observations) AND<br>• Verbatim quotations from participants AND<br>• Papers that have undergone formal peer-review

Note. Social services professionals cover a range of services provided to advance adult and child welfare including child protection services.

Multiple papers from the same study were included if they each reported new data relevant to the research questions. Exclusion of non-English papers and papers that have not been through the formal peer review system (e.g., books, conference papers, editorials, letters, general comment papers) was justified by limited resources and concerns about validity and reliability of non-peer-reviewed sources, respectively.

Two reviewers independently screened titles and abstracts of all references. The first reviewer screened all full text papers, the second reviewers screened a 10% subset, and disagreements (27%) were resolved through discussion and consensus.

For peer review only

For peer review only - http://bmjopen.bmj.com/site/about/guidelines.xhtml

Analysis

Quality appraisal. Two reviewers independently assessed each study for methodological validity using the Critical Appraisal Skills Programme Qualitative checklist[21] modified for the purpose of the VEGA Project (M-CASP, score range 0-20).[22] Disagreements not resolvable between reviewers were resolved by a third reviewer. We did not exclude studies on reporting quality;[23] we conducted a secondary sensitivity analysis by restricting the synthesis to studies in the top tertile of methodological quality (M-CASP score $\geq$15).[24]

Data extraction. We adapted a data extraction form from IMPROVE.[20] The first reviewer extracted study details; second reviewers checked the extracts. The papers were then treated as primary text transcripts. Where studies included varied participants, only data relevant to our inclusion criteria were considered. Two reviewers independently extracted raw qualitative data[25] relevant to the views and direct experience of identification and initial response to children's exposure to IPV from Results sections of the included papers. These data could be in the form of participants' quotes or authors' interpretations of participants' voices. For each study, the reviewer entered data extracts into the form separately for children, parents and professionals.

Synthesis. Our choice of synthesis method was guided by the practical aims of this review, the "thickness" of the data reported in the primary studies, and the expertise of the team.[26 27] We applied thematic synthesis[25 28] in three stages:

I. *Line-by-line coding* alongside data extraction. Two reviewers independently coded the data extracts for themes relevant to the acceptability of the identification approach and initial response to children's exposure to IPV[29] subsequently meeting to compare and combine their codes. The first reviewer produced a final table of codes with supporting verbatim text for each participant group in each study.

II. *Developing descriptive themes.* The first reviewer grouped the codes into themes and sub-themes with accompanying verbatim to capture consistency and range of views within each participant group and across the studies.[29] Second reviewers commented on the table leading to the final version.

III. *Generating analytical themes.* The first reviewer used the constant comparison method[30] to integrate perspectives across child, parent and professional groups. The integrated stakeholder perspectives were categorised by level of agreement within and across the groups.[31] When perspectives on a theme were consistent, it was categorised as convergent. When stakeholders' views on a theme were consistent within groups, but differed between them, it was categorised as divergent. Finally, themes with wide variation, within and between the groups, were

For peer review only - http://bmjopen.bmj.com/site/about/guidelines.xhtml

categorised as conflicting. This integration through categorisation produced interim analytical themes which were further refined in relation to the research questions. Our approach allowed us to: (i) integrate perspectives of all participant groups and generate new interpretations beyond findings from primary studies, (ii) identify gaps in evidence within and across participant groups; and (ii) establish areas of conflicting perspectives which can be targeted in future research and interventions. Throughout this stage, the first reviewer developed diagrams and tables with interim analytical themes which were refined during group discussions at three meetings with all second reviewers.

Patient and Public Involvement

Patients and public were not involved in the design and conduct of this secondary analysis of published research.

RESULTS

Sixteen papers reporting 11 studies were included (Figure 1); three were reported in multiple papers. Two papers were from a study on parents' experiences with Irish child protection services.[32 33] Two papers drew on a study of police and children's social services responses to IPV incidents where children were present or resided in the household.[34 35] Four papers were from the RESPONDS study on general practice clinicians' perspectives on child safeguarding in IPV cases.[36-39] All papers were published between 2008 and 2015.

Studies characteristics and methodological quality

The 11 included studies are summarised in Table 2; online Supplementary file 2 contains detailed studies characteristics. Ten studies were based in high-income countries: UK,[35 39 40] US,[41-43] Australia,[44 45] Ireland,[32] and Canada.[46] One study was based in a middle-income country, Brazil.[47]

Table 2. Summary characteristics of the 11 studies (16 papers) synthesised (in chronological order)

Study	Country	Topic	Methodology	Stakeholder group			
				Children	Parents	HCPs	SSPs
Buckley, 2008[32 33]	Ireland	Experiences of child protection services	Interviews		X		
Black,	US	Interventions	Interviews			X	X

For peer review only - http://bmjopen.bmj.com/site/about/guidelines.xhtml

Study	Country	Topic	Methodology	Stakeholder group			
				Children	Parents	HCPs	SSPs
2010[41]		for IPV					
Stanley, 2010[34 35]	UK	Police IPV notifications of children's social services	Interviews	X	X		X
Meyer, 2011[44]	Australia	Help-seeking of IPV victims with children	Interviews		X		
Randell, 2012[43]	US	IPV information in healthcare setting	Focus groups		X		
Davidov, 2012[42]	US	Mandatory reporting of children's exposure to IPV	Secondary analysis of interviews and focus groups		X	X	X
Angelo, 2013[47]	Brazil	Experiences of providing care to children exposed to IPV	Interviews			X	
Jenney, 2014[46]	Canada	Communication between providers and recipients of child protection service	Interviews and focus groups		X		X
Szilassy, 2015[36-39]	UK	Experiences of responding to children's exposure to IPV	Interviews			X	
Clarke,	UK	Experiences of	Interviews,		X	X	X

For peer review only

For peer review only - http://bmjopen.bmj.com/site/about/guidelines.xhtml

Study	Country	Topic	Methodology	Stakeholder group			
				Children	Parents	HCPs	SSPs
2015[40]		responding to children's exposure to IPV	focus groups, observations				
Morris, 2015[45]	Australia	Safety and resilience of children exposed to IPV	Interviews and focus groups	X	X		

Note. US – United States. UK – United Kingdom. IPV – intimate partner violence. HCPs – health care professionals. SSPs – social service professionals.

Voices of children were reported in two studies,[35 45] parents in eight studies[32 35 40 42-46] and professionals in seven studies.[35 39-42 46 47] Overall, the studies involved 42 children and young people aged 8 to 24 (19 from IPV and social services, 23 from general practice), 220 parents (212 mothers), and 251 professionals (113 health care, 42 social services, and 96 mixed samples). All parents were IPV survivors. HCPs included physicians[37 41] and nurses[37 42 47] from primary and secondary health care. SSPs were drawn from children's social services,[35] child protection services[46] and unspecified settings.[40 41]

Of 11 studies, seven [30 39-44] scored ≥15 out of 20 on the M-CASP indicating their overall good methodological quality (Figure 2, online Supplementary file 3). The main shortcomings identified were in the M-CASP domains of reliability and objectivity. Thus the authors did not justify their choices of study design,[35 37 40 41 44 47] research methods,[35 37 40-43 46 47] participant selection[35 40 41 43 44 46 47] and recruitment.[33 35 37 40 42 43 47] Only two studies[41 45] described strategies for establishing neutrality.

Synthesis

Line-by-line coding and descriptive themes

Initial line-by-line coding generated 75 codes. We grouped them into 22 descriptive themes with 13 subthemes related to: (1) experiences of identifying children's exposure to IPV; (2) experiences of the initial response to children's exposure; (3) factors enabling identification and initial response to children's exposure ; (4) reasons for not identifying or disclosing children's exposure or not engaging with services; (5) psychological consequences of individuals' involvement in identification and initial

For peer review only - http://bmjopen.bmj.com/site/about/guidelines.xhtml

response to children's exposure; and (6) suggested training and resources (see online Supplementary file 4). All relevant parents' quotations were from mothers who have experienced IPV.

Analytical themes

Categorisation of participant views by agreement/disagreement within and across the three stakeholder groups showed converging, diverging and conflicting perspectives on satisfactory identification and responses to children's exposure to IPV. These perspectives were expressed in five interim analytical themes (see online Supplementary file 5) which were refined further, when possible resolving conflicting perspectives within and across the studies. Finally, we articulated the final analytical themes as descriptions of an ideal identification and initial response:

1. Precursors for acceptable identification and response,
2. Acceptable identification,
3. Acceptable initial response, and
4. Conflicting perspectives on engagement with children and management of safety (Table 3).

Not all participant groups contributed equally to the final analytical themes. Thus, professionals' perspectives were presented across 15/17 subthemes, while mothers informed 13/17 and children 9/17. Children's quotes were not available for most subthemes covering an acceptable initial response. Quotes from each stakeholder group supporting the final analytical themes are collated in the online Supplementary file 6.

I. Precursors for acceptable identification and response. This theme was developed from converging perspectives on facilitators and barriers to satisfactory identification and response. It captures areas of agreement within and between stakeholder groups on the enabling processes and conditions required before children's exposure to IPV is disclosed or discussed. Mothers and children know and trust professionals who are non-judgemental and non-threatening, have good communication skills, can ensure confidentiality, and can offer practical help.[32 35 37 41 42 44-46] Good communication and trusting patient-professional relationship make mothers and children feel comfortable and safe in discussing sensitive issues and to return for support, if needed.[32 45] Professionals match their approaches to the individual mother's readiness to disclose and engage with services and work with the mother towards increasing her readiness.[35 41 43 44 46] Culturally sensitive materials in different languages on IPV and children's exposure to IPV are displayed in healthcare settings to prepare patients for enquiry and provide information for those who are not ready to disclose and engage.[41 43]

For peer review only - http://bmjopen.bmj.com/site/about/guidelines.xhtml

Professionals receive sufficient training and guidance built upon local policies on children's exposure to IPV and inter-agency work with children's social services and IPV services.[37 41 42] Training fits into daily practice of HCPs and SSPs and prepares them to better communicate with children.[37] Training and guidance clarify: (i) what constitutes children's exposure to IPV, especially psychological and non-direct physical, (ii) what are professionals' roles and reporting duties, (iii) how and where to document children's exposure to IPV, and (iv) how and where to signpost mothers and children.[32 35-37 41-46] Training and guidance increase professional awareness, skills and confidence, which lead to more satisfactory identification and responses.

Mothers, children and professionals receive emotional support and help with managing the emotional burden of involvement in identifying and responding to IPV. For patients/clients, such support is provided through non-judgemental responses and confidentiality (or being clear when reports must be made).[32 34 35 43 45] For professionals, ongoing emotional support and supervision need to be arranged.[35 37 41 47] This helps to maintain well-being and mental health of patients and professionals and leads to increased satisfaction.

The acceptability of any work related to identification of and response to children's exposure to IPV can be undermined by systems-level constraints, such as high demand on services, lack of resources and system support, and poor inter-agency collaboration.[35 37 40 41] Therefore, the approaches to identification and responses must be aligned with the capabilities of under-resourced health care and social services.

II. Acceptable identification This theme was developed from converging perspectives on the acceptable approaches to identification. It captures areas of agreement within and between stakeholders on how professionals should ask about children's exposure to IPV. The enquiry is built upon a trusting patient-professional relationship, takes place in a safe and supportive environment and is integrated into the context of the consultation or visit.[37 41 42 45 47] Acceptable identification involves a trained and well-supported HCP who is non-judgemental and has good communication skills.[32 35 37 41 42 44-46] HCP uses a phased approach, i.e. starts from the presenting symptoms, then moves to general safety and well-being of the child and finally asks about feeling safe at home.[37 41 42 45 47]

III. Acceptable initial responses. This theme was developed from converging perspectives on what the acceptable initial response should include and how it should be delivered. It also captures conflicting professional perspectives on the focus of their responses, reported within and between three studies[35 37 41] and resolved though justifying that child safety is prioritised over mother's safety and confidentiality. Due to the overlap between IPV and children's exposure to IPV, most

For peer review only - http://bmjopen.bmj.com/site/about/guidelines.xhtml

HCPs first target their initial response to the mother-child dyad.[37 41] However, their focus shifts towards the child if they are at risk of harm.[35 37] Some professionals feel conflicted when prioritising the child's needs over the adult's and require assistance with managing the emotional burden.[41] Professionals educate the mother about the impact of IPV on children, as well as the roles and duties of different providers.[35 37 41-43 46] This helps to relieve mothers' fears and increase their readiness to engage with services. Importantly, the approach to mothers education should not jeopardise their safety (e.g., by sending materials home).[35] Mothers and children must also be given information about local IPV services.[35 37 46]

IV. Conflicting perspectives on engagement with children and management of safety. This theme captures divergent and conflicting perspectives on the appropriateness of engaging directly with children[35 37 40 45] and satisfactory management of safety.[35 37 40 45 46] The variations were reported within and between studies and remain un-resolved in our review. While most children and mothers are positive about professionals talking directly to children and addressing their individual needs, a few held the opposite view.[35 45] In contrast, most professionals do not feel competent in communicating directly with children and prefer to assess children's needs through a proxy adult,[37 39] while some report always engaging with children and some think they should engage more.[35 40] Furthermore, all stakeholder groups have different views on the appropriate age for talking directly to children about IPV.[37 39 45] For example, some mothers think that HCPs can talk to their children about IPV from the age of five, while some doubt the acceptability of such conversations even with adolescents.[45] Finally, children and HCPs do not agree on the appropriate age for seeing a doctor alone. Most children prefer their mother or trusted adult to be with them at the consultation and indicate that at least teenagers should be seen alone.[45] Similarly, most HCPs indicate they would not see children alone, with some suggesting that if they were to do so, they would need to be adolescents. A few HCPs suggest that they would seek a parent's and child's permission to see the child alone, but they do not elaborate on the practicalities of obtaining such permission.[37]

Next, mothers and SSPs have divergent and conflicting views on the management of child safety and both perceive current practices as unsatisfactory. First, safety is understood differently by mothers and professionals. Actions perceived by mothers as increasing child's safety (e.g., staying with perpetrator for financial reasons, not seeking help to prevent escalation of abuse) are seen by SSPs as increasing the risk for the child.[44 46] While most mothers think that involvement of children's social services increases the risk for the child through potential escalation of abuse and child removal, most SSPs believe that their involvement results in greater safety. Whereas most SSPs think that women and children are safer out of the abusive relationship and feel frustrated when women

For peer review only - http://bmjopen.bmj.com/site/about/guidelines.xhtml

do not follow their advice to leave, women do not feel safer after leaving the perpetrator because of potential escalation of abuse, child contact with perpetrator, and lack of support post-separation. However, mothers feel intimidated by SSPs and that they must adhere to their instructions to keep children in the home and minimise involvement of children's social services.[32 44 46]

Second, stakeholders' perspectives on acceptable risk assessment and safety planning vary and diverge. Children's preferences for the assessment of risk in all environments are represented by one boy's voice which does not match any of the professional or parent viewpoints.[45] Mothers report not being included in safety planning with SSPs and want their views to be considered.[46] Professionals have polarised perspectives on the acceptability of the current risk assessment and safety planning with most feeling dissatisfied with the inconsistent approaches and bureaucracy involved.[35 37 40 41 46] A few SSPs suggest engaging with mothers and children more without elaborating on how this should be done.[40] All stakeholders report feeling conflicted about the existing management of safety, but have to comply due to the imbalance of power and system requirements.[35 37 40 41 46]

For peer review only

For peer review only - http://bmjopen.bmj.com/site/about/guidelines.xhtml

Table 3. Final analytical themes and their definitions

Final analytical themes with subthemes	Definition	Stakeholder group, study		
		Children	Mothers	Professionals
**1. Precursors for acceptable identification and response**				
**1.1. Satisfying and sustainable relationship**	Patients know and trust professionals with whom they develop good long-term relationships. Trusting relationships enable patients to feel safe and comfortable to discuss sensitive issues.	[45]	[32 44-46]	[37 41 46]
**1.2. Desired professional attitudes and skills**	When interacting with patients/clients, professionals demonstrate non-judgemental, non-threatening attitudes, show respect, actively listen, validate patient's accounts, reassure confidentiality, and provide practical help.	[35 45]	[32 35 42 44-46]	[37 41 42]
**1.3. Considering mother's readiness**	Professionals acknowledge individual mothers' readiness to disclose IPV and engage with services, work towards increasing mothers' readiness and match their approaches to the stage of mothers' readiness.		[43 44]	[35 41 46]
**1.4. Patient materials**	Culturally sensitive materials on IPV and children's exposure to IPV in different languages are displayed in healthcare settings.		[43]	[41]
**1.5. Professional training**	Professionals receive adequate training on communication with children, indicators of children's exposure to IPV, especially psychological and non-direct physical IPV, professionals' role in identifying and responding, documenting and reporting, inter-agency work.	[35 45]	[32 35 43-46]	[35-37 41 42]
**1.6. Professional resources**	Professionals have clear guidance on local IPV resources, what constitutes children's exposure to IPV, what is reportable, and how to document children's exposure to IPV in a way that keeps the			[37 41 42]

For peer review only - http://bmjopen.bmj.com/site/about/guidelines.xhtml

Final analytical themes with subthemes	Definition	Stakeholder group, study		
		Children	Mothers	Professionals
	child safe and ensures the safety and confidentiality of the mother.			
**1.7. Professional supervision and support**	Professionals have skilled supervision and ongoing support for coping with psychological consequences of working with children and mothers exposed to IPV and preventing vicarious trauma.			[35 37 41 47]
**1.8. Addressing systems' barriers**	Professionals' work of identifying and responding to children's exposure to IPV fits into the organisational, local and national context of increased demands on health care and social services without commensurate resources.			[35 37 40 41]
**2. Acceptable identification**				
**2.1. Space and time**	It is ideal to give patients permission, space and time to discuss sensitive matters.	[45]	[45]	
**2.2. Vocabulary**	It is preferable for HCPs to phrase questions about children's exposure to IPV as a "safety-at-home" matter.	[45]	[45]	
**2.2. Phased approach**	When asking about children's exposure to IPV, it is ideal for HCPs to initiate the enquiry, adapt it to the context of the consultation, and use a phased approach – from presenting symptoms to general safety and well-being, then to safety at home.	[45]	[45]	[37 41 42 47]
**3. Acceptable initial response**				
**3.1. Shifting focus**	Professionals first focus their responses on the mother-child dyad and shift to the child if he/she is at risk of harm. Professionals need assistance with managing emotional burdens caused by the shift.			[35 37 41]
**3.2. Emotional support**	When responding to disclosure, it is ideal to provide children and parents with encouragement		[46]	[37 39 47]

For peer review only - http://bmjopen.bmj.com/site/about/guidelines.xhtml

Final analytical themes with subthemes	Definition	Stakeholder group, study		
		Children	Mothers	Professionals
	and emotional support.			
**3.3. Education**	It is acceptable to educate mothers about the impact of IPV on children, IPV dynamics, professionals' roles and duties in responding. However, education should not jeopardise patient safety (e.g., through sending materials home where the perpetrator can find them).		[43]	[35 37 41 42 46]
**3.4. Signposting**	It is acceptable for professionals to give children and mothers information about local IPV services.	[35]	[35]	[35 37 46]
**4. Conflicting perspectives on engagement with children and management of safety**				
**4.1. Engaging directly with children**	Stakeholders' perspectives on the acceptability of talking directly to children exposed to IPV and seeing them alone are conflicting. Children are absent in the patient-professional communication. Mothers and children want direct engagement with children. Professionals do not see children as patients on their own and feel ill equipped for communicating with children about IPV.	[34 35 45]	[45]	[35 37 40]
**4.2. Management of safety**	Stakeholder preferences regarding risk assessment and safety planning are conflicting. Mothers and children are absent in the management of safety and want to be involved. Professionals are not satisfied with current risk assessment and safety planning approaches and want them to change.	[45]	[46]	[35 37 40 41]

Note. IPV – intimate partner violence. HCPs – health care professionals. SSPs – social care professionals.

For peer review only - http://bmjopen.bmj.com/site/about/guidelines.xhtml

Sensitivity analysis

Exclusion of four studies scoring below 15 on the M-CASP in sensitivity analysis[35 40 41 47] did not change the final analytical themes.

DISCUSSION

Main findings

This synthesis included 11 qualitative studies with 513 providers and recipients of health care and social services. We identified enabling precursors, ideal approaches, and areas of disagreement among children, mothers and professionals regarding the acceptable identification and initial response to children's exposure to IPV. Enabling precursor processes were linked to patient/client-professional relationship building, creating a safe and supporting environment, and changing and matching responses according to individual mothers' readiness to disclose and engage with services. Enabling conditions included sufficient training and multiple language-versions materials and embedding the work of identifying and responding to children's exposure to IPV into the context of under-resourced services. Acceptable identification involved a phased approach to enquiry. An ideal initial response included emotional support, education about IPV and signposting to IPV services. Areas of disagreement were related to the acceptability of engaging directly with children and managing child's safety.

An important finding of this synthesis is that participants' views on many factors related to satisfactory identification and initial response to children's exposure to IPV are strikingly consistent. Our first theme suggests that mothers and children need to have a trusting relationship with a professional who demonstrates certain attitudes and skills before enquiry and identification occurs. Combined with the theme of a phased approach to enquiry, our findings support a case-finding approach to identifying IPV. Our third theme supports the acceptability of providing mothers and children with emotional support, education about IPV and signposting to local IPV services. These essential components of good clinical practice on identification and response to IPV are supported in the literature[2 24 48] and highlight the importance of safe environments and trusting relationships as conditions for recognition of and response to mothers' and children's exposure to IPV.[49] Furthermore, the acceptability of a case-finding approach is consistent with the approach outlined by WHO on identification of child maltreatment in the recent mhGAP Intervention Guide.[50] The ideal initial responses to children's exposure to IPV are in line with the WHO LIVES principles for IPV response (**L**isten, **I**nquire about needs and concerns, **V**alidate, **E**nhance safety, and **S**upport).[1]

For peer review only - http://bmjopen.bmj.com/site/about/guidelines.xhtml

Another notable finding is the role of the wider context. Although most of the included studies were based in high-income countries, all professionals described how their ability to identify and respond to children's exposure to IPV was heavily influenced by healthcare and social service systems constraints. Specifically, they felt they lacked time to engage with their patients about sensitive issues; they felt burdened by constant cuts and restructuring of health care and social services; and they expressed frustration about poor access to referral pathways. These systemic factors were compounded by poor communication and coordination across organisations and absence of a single referral pathway, consistent with findings in research on child protection services.[51 52] This highlights the importance of targeting interventions on identification and initial response to IPV at both individual and system levels,[53-55] with professional guidance adaptable to the changing landscape of local services.

Our findings about the emotional burden of IPV work suggest that identifying and responding to children's exposure to IPV can have negative psychological impact on both providers and recipients of care (e.g., disempowerment, compassion fatigue and vicarious trauma). The common causes of distress are patients'/clients' feelings of shame and guilt linked to the acknowledgement of IPV and disclosure, professionals' ambiguous feelings towards mothers who did not follow their advice, tensions when shifting the focus from mother-child dyad to the child, and frustration with system-level obstacles. These findings, which are consistent with previous research,[56-58] emphasise the importance of assisting both patients/clients and professionals with managing psychological symptoms and preventing vicarious trauma.[58-60] The results also supports a trauma- and violence-informed approach to care of adults and children exposed to violence.[59-62]

One of the most striking findings of this review is the gap between including children's own needs and preferences, and the lack of attention paid by professionals to children as patient/clients on their own. Although professionals recognised the importance of working with a mother-child dyad exposed to IPV, most did not perceive children as individual patients/clients and used a proxy adult to assess and respond to the child's needs. In contrast, mothers and children were in favour of HCPs talking directly to children, treating each one as an individual and addressing the child's needs directly. The invisibility of children's own views has been previously reported[63-67] and can be explained by ethical and methodological challenges of undertaking research with children,[68] rigid professional attitudes, and lack of knowledge/competence on communicating with children. However, the finding is still concerning, especially given growing recognition of children's rights[69] and agency.[70] The gap between patient and professional preferences for talking directly to children suggests that professionals need training and guidance on communicating with children about sensitive issues. With regards to children's exposure to IPV specifically, professionals need

For peer review only - http://bmjopen.bmj.com/site/about/guidelines.xhtml

help understanding safety requirements, such as asking children in a private, confidential environment about exposure to IPV.[50]

Patients/clients and professionals held conflicting views about the acceptable management of safety. Neither patients nor professionals were satisfied with current risk assessment and safety planning. Addressing the needs of mother and child involves aligning professionals' and mothers' diverging perspectives about safety and risk. While professionals may think that leaving an abusive partner is a pre-requisite for safety, this is the time when women are at greatest risk of homicide;[71] furthermore, women themselves know when it is safest to leave.[72] In our synthesis, professionals favoured targeting the mother-child dyad, but found it acceptable to switch the focus to the child when they are at risk of harm. This is consistent with literature which suggests a hierarchy of needs for children and caregivers, recognising the needs of both mother and child, but prioritising the child given children's inherent vulnerability.[73]

While not the focus of this synthesis, it is important to note that mandatory reporting of child maltreatment laws may complicate or intertwine with strategies for inquiring about exposure to IPV, as children's exposure to IPV is a reportable exposure in some jurisdictions.[74 75] We found that most HCPs were confused as to whether or not children's exposure to IPV was reportable in their jurisdiction. They felt anxiety about the reporting duties and thresholds. This finding is in line with the recent meta-synthesis on mandated reporters' experiences with reporting child maltreatment.[22] The authors offer recommendations for mitigating potential harms associated with the reporting processes. The strategies include disclosing reporting duties and the limits of confidentiality when providers start a relationship, consulting with child protection services in an anonymous manner when a provider is unsure if the suspected maltreatment is reportable, and – if the suspected maltreatment is reportable – discussing with the child/family how the provider will file a report (when it is safe for the child to do so) and likely child protection service responses to the report. Discussing reporting duties and limits of confidentiality should ideally occur before inquiry, to minimise feelings of betrayal that may emerge when a provider realises they must report. These recommended strategies are applicable to the reportable children's exposure to IPV.

Strengths and limitations

We used comprehensive strategies for retrieving papers, including systematic searches of bibliographic databases, citation searching, reference checking, and emailing topic experts. Involvement of two reviewers throughout screening, data abstraction and critical appraisal reduced potential bias. Reviewers' backgrounds in different disciplines broadened and enriched data interpretation. Bringing together perspectives of children, mothers and professionals gave the

For peer review only - http://bmjopen.bmj.com/site/about/guidelines.xhtml

recipients of care a voice equal to the providers. Our analytical themes are easily understood and can be used by practitioners and policy makers as targets for interventions on identification and response to children's exposure to IPV.

This review has several limitations. Only papers published in English were included due to limited resources. This limitation alongside the exclusion of books and conference abstracts could result in missing studies relevant to the review questions. However, our decision to focus on papers that have been peer-reviewed and potentially of better methodological quality, increases the robustness of our findings.

The evidence we produced should be interpreted with caution, taking into consideration the following limitations of the primary data. It is supported by only 11 studies from high- and middle-income countries, four of which had methodological shortcomings regarding reliability and nine were lacking objectivity. Some important information (e.g., mandatory reporting status of professionals) was not specified. The selective nature of the stakeholders' sample makes our findings relevant to HCPs and children's social service providers, mothers who have experienced IPV and their children. All evidence supported by children's voices came from two studies. Although small, the sample of children covered varied ages and settings.

Future studies should explore children's own experiences of encounters with health care and social services around identification and initial responses to IPV. Children's voices will provide important information on their values and preferences. Future studies should also recruit fathers who have experienced IPV. Our fourth theme of conflicting perspectives should guide future research on the acceptable approaches to talking directly to children about IPV, assessing risk and undertaking risk assessment and safety planning.

Conclusion

The present analysis adds to the evidence base important, client-centred considerations drawn from qualitative research to enhance approaches to the identification of, and response to, children exposed to IPV, and their caregivers. Health care and social service professionals should receive sufficient training and on-going individual and system-level support to recognise and respond safely to children's exposure to IPV. Ideal identification and response should be based on the WHO LIVES principles,[12] integrated into a trauma- and violence-informed model of care.[76] In Canada, findings from the present analysis have been integrated into professional guidance and curriculum in the VEGA Project.[13]

For peer review only - http://bmjopen.bmj.com/site/about/guidelines.xhtml

FUNDING

This work was supported by the Public Health Agency of Canada, through funding to the VEGA Project (HLM, NW). HLM was supported by the Chedoke Health Chair in Child Psychiatry. NVL was also supported by the Avon Primary Care Research Collaborative and the National Institute for Health Research (NIHR) Collaboration for Leadership in Applied Health Research and Care North Thames at Bart's Health NHS Trust (NIHR CLAHRC North Thames). The views expressed in this article are those of the author(s) and not necessarily those of the NHS, the NIHR, or the Department of Health and Social Care.

COMPETING INTERESTS

We have read and understood BMJ policy on declaration of interests and declare that we have no competing interests.

ACKNOWLEDGEMENTS

The authors thank Dr Lisa Arai for performing the electronic searches and for helping with title and abstract screening. The authors are grateful to Khabo Piggott and Eleanor Wright who volunteered to help with data extraction, coding and critical appraisal.

CONTRIBUTORS

HLM and NW conceived the idea. GSF, NVL, EH, ES designed the study. NVL identified additional studies, carried out title and abstract screening, full text screening, quality appraisal, data extraction, coding and synthesis. She reported the research and drafted the paper. EH, ES, GSF conducted double full text screening, data extraction, coding and quality appraisal. NVL, EH, ES, GSF, JRM contributed to the development of descriptive and analytical themes. All authors contributed to the interpretation of the data and five revisions of the paper.

DATA SHARING STATEMENT

Data extracted from each of the 16 papers are stored in Word files and are available from the corresponding author upon reasonable request. Raw syntheses of these data are stored in Excel and Word files and are available from the corresponding author upon reasonable request.

For peer review only - http://bmjopen.bmj.com/site/about/guidelines.xhtml

REFERENCES

1. WHO. Health care for women subjected to intimate partner violence or sexual violence. http://www.who.int/reproductivehealth/publications/violence/vaw-clinical-handbook/en/: WHO, 2014.
2. Feder G, MacMillan HL. Intimate partner violence. In: Goldman L, Schafer AI, eds. Goldman's Cecil Medicine (25th ed). Philadelphia, PA: Elsevier Saunders, 2015.
3. WHO. Global and regional estimates of violence against women: prevalence and health effects of intimate partner violence and nonpartner sexual violence. Italy: WHO, 2013:51.
4. García-Moreno C, Jansen H, Ellsberg M, Heise L, Watts C. WHO multi-country study on women's health and domestic violence against women : initial results on prevalence, health outcomes and women's responses. Geneva, Switzerland: WHO, 2005:118.
5. MacMillan HL, Wathen CN. Children's exposure to intimate partner violence. Child Adolesc Psychiatr Clin N Am 2014;**23**(2):295-308, viii-ix doi: 10.1016/j.chc.2013.12.008[published Online First: Epub Date]|.
6. Radford L, Hester M. *Mothering through domestic violence*. London ; Philadelphia: Jessica Kingsley Publishers, 2006.
7. Finkelhor D, Turner HA, Shattuck A, Hamby SL. Prevalence of Childhood Exposure to Violence, Crime, and Abuse: Results From the National Survey of Children's Exposure to Violence. JAMA Pediatr 2015;**169**(8):746-54 doi: 10.1001/jamapediatrics.2015.0676[published Online First: Epub Date]|.
8. McTavish JR, MacGregor JC, Wathen CN, MacMillan HL. Children's exposure to intimate partner violence: an overview. Int Rev Psychiatry 2016:1-15 doi: 10.1080/09540261.2016.1205001[published Online First: Epub Date]|.
9. Holden GW. Children exposed to domestic violence and child abuse: terminology and taxonomy. Clin Child Fam Psychol Rev 2003;**6**(3):151-60
10. Government HM. Working together to safeguard children. A guide to inter-agency working to safeguard and promote the welfare of children: HM Government, 2015:109.
11. NICE. Domestic violence and abuse: How social care, health services and those they work with can respond effectively. London: NICE, 2014.
12. WHO. Responding to Intimate Partner Violence and Sexual Violence Against Women: WHO Clinical and Policy Guidelines. Geneva: WHO, 2013.
13. PreVAiL. VEGA (Violence, Evidence, Guidance and Action) Project: a public health response to family violence. Secondary VEGA (Violence, Evidence, Guidance and Action) Project: a public health response to family violence. https://projectvega.ca/.
14. Green J, Thorogood N. *Qualitative methods for health research*. Third edition. ed. Los Angeles: SAGE, 2014.
15. Malterud K. Qualitative research: standards, challenges, and guidelines. Lancet 2001;**358**(9280):483-8 doi: 10.1016/S0140-6736(01)05627-6[published Online First: Epub Date]|.
16. CRD. Systematic reviews: CRD's guidance for undertaking reviews in health care. In: Dissemination CfRa, ed. Centre for Reviews and Dissemination: University of York, 2009:294.
17. Liberati A, Altman DG, Tetzlaff J, et al. The PRISMA statement for reporting systematic reviews and meta-analyses of studies that evaluate health care interventions: explanation and elaboration. J Clin Epidemiol 2009;**62**(10):e1-34 doi: 10.1016/j.jclinepi.2009.06.006[published Online First: Epub Date]|.
18. Tong A, Flemming K, McInnes E, Oliver S, Craig J. Enhancing transparency in reporting the synthesis of qualitative research: ENTREQ. BMC Med Res Methodol 2012;**12**:181 doi: 10.1186/1471-2288-12-181[published Online First: Epub Date]|.
19. Booth A. Searching for qualitative research for inclusion in systematic reviews: a structured methodological review. Syst Rev 2016;**5**(74)

For peer review only - http://bmjopen.bmj.com/site/about/guidelines.xhtml

20. Howarth E, Moore T, Welton N, et al. IMPRoving Outcomes for children exposed to domestic ViolencE (IMPROVE): an evidence synthesis. Public Health Res 2016;**4**(10)
21. CASP. Critical Appraisal Skills Programme. CASP Qualitative Research Checklist 31.05.13. Secondary Critical Appraisal Skills Programme. CASP Qualitative Research Checklist 31.05.13 2013. http://www.casp-uk.net/checklists.
22. McTavish J, Kimber M, Devries K, et al. Mandated reporters' experiences with reporting child maltreatment: a meta-synthesis of qualitative studies. BMJ Open 2017 Oct 16;**7**(10):e013942 doi: 10.1136/bmjopen-2016-013942[published Online First: Epub Date]|.
23. Carroll C, Booth A. Quality assessment of qualitative evidence for systematic review and synthesis: Is it meaningful, and if so, how should it be performed? Res Synth Methods 2015;**6**(2):149-54 doi: 10.1002/jrsm.1128[published Online First: Epub Date]|.
24. Feder GS, Hutson M, Ramsay J, Taket AR. Women exposed to intimate partner violence: expectations and experiences when they encounter health care professionals: a meta-analysis of qualitative studies. Arch Intern Med 2006;**166**(1):22-37 doi: 10.1001/archinte.166.1.22[published Online First: Epub Date]|.
25. Thomas J, Harden A. Methods for the thematic synthesis of qualitative research in systematic reviews. BMC Med Res Methodol 2008;**8**:45 doi: 10.1186/1471-2288-8-45[published Online First: Epub Date]|.
26. Hannes K, Lockwood C. *Synthesising qualitative research : choosing the right approach*. Chichester, West Sussex, UK: John Wiley & Sons, 2012.
27. Noyes J, Hannes K, Booth A, et al. Chapter 20: Qualitative research and Cochrane reviews. In: Higgins J, Green S, eds. Cochrane handbook for systematic reviews of interventions. Version 5.3.0 (updated October 2015): The Cochrane Collaboration, 2015.
28. Barnett-Page E, Thomas J. Methods for the synthesis of qualitative research: a critical review. BMC Med Res Methodol 2009;**9**:59 doi: 10.1186/1471-2288-9-59[published Online First: Epub Date]|.
29. Braun V, Clarke V. Using thematic analysis in psychology. Qualitative Research in Psychology 2006;**3**(2):77-101 doi: 10.1191/1478088706qp063oa[published Online First: Epub Date]|.
30. Boeije H. A purposeful approach to the constant comparative method in the analysis of qualitative interviews. Qual Quant 2002;**36**(4):391-409
31. Shim WS, Haight WL. Supporting battered women and their children: Perspectives of battered mothers and child welfare professionals. Child Youth Serv Rev 2006;**28**(6):620-37 doi: 10.1016/j.childyouth.2005.06.005[published Online First: Epub Date]|.
32. Buckley H, Whelan S, Carr N. 'Like waking up in a Franz Kafka novel': Service users' experiences of the child protection system when domestic violence and acrimonious separations are involved. Child Youth Serv Rev 2011;**33**(1):126-33
33. Buckley H, Whelan S, Carr N, Murphy C. Service users' perceptions of the Irish Child Protection System. The Stationery Office, Dublin: Office of the Minister for Children and Youth Affairs, 2008:96.
34. Stanley N, Miller P, Richardson Foster H. Engaging with children's and parents' perspectives on domestic violence. Child & Family Social Work 2012;**17**(2):192-201
35. Stanley N, Miller P, Richardson Foster H, Thompson G. Children and families experiencing domestic violence: Police and children's social services' responses. London: NSPCC University of Lancashire, 2010:268.
36. Drinkwater J, Stanley N, Szilassy E, Larkins C, Hester M, Feder G. Juggling confidentiality and safety: A qualitative study of how general practice clinicians document domestic violence in families with children. BJGP 2017 doi: 10.3399/bjgp17X689353[published Online First: Epub Date]|.
37. Szilassy E, Das J, Drinkwater J, et al. Researching Education to Strengthen Primary care ON Domestic violence & Safeguarding (RESPONDS). Final Report for the Department of Health, Policy Research Programme Project Bristol: University of Bristol, 2015.

For peer review only - http://bmjopen.bmj.com/site/about/guidelines.xhtml

38. Szilassy E, Drinkwater J, Hester M, et al. Making the links between domestic violence and child safeguarding: an evidence-based pilot training for general practice. Health Soc Care Community 2016 doi: 10.1111/hsc.12401[published Online First: Epub Date]|.
39. Larkins C, Drinkwater J, Hester M, Stanley N, Szilassy E, Feder G. General practice clinicians' perspectives on involving and supporting children and adult perpetrators in families experiencing domestic violence and abuse. Family practice 2015;**32**(6):701-05 doi: 10.1093/fampra/cmv070[published Online First: Epub Date]|.
40. Clarke A, Wydall S. From 'Rights to Action': practitioners' perceptions of the needs of children experiencing domestic violence. Child & Family Social Work 2015;**20**(2):181-90 doi: 10.1111/cfs.12066[published Online First: Epub Date]|.
41. Black S, Dempsey SH, Davis MB. Practitioner-recommended policies and procedures for children exposed to domestic violence. Health Promotion Practice 2010;**11**(6):900-7
42. Davidov DM, Jack SM, Frost SS, Coben JH. Mandatory reporting in the context of home visitation programs: intimate partner violence and children's exposure to intimate partner violence. Violence Against Women 2012;**18**(5):595-610
43. Randell KA, Bledsoe LK, Shroff PL, Pierce MC. Educational Interventions for Intimate Partner Violence Guidance From Survivors. Pediatric Emergency Care 2012;**28**(11):1190-96 doi: 10.1097/PEC.0b013e318271be7f[published Online First: Epub Date]|.
44. Meyer S. 'Acting in the children's best interest?': Examining victims' responses to intimate partner violence. Journal of Child and Family Studies 2011;**20**(4):436-43
45. Morris A. Safety and resiliency at home: Voices of children from a primary care population (Unpublished PhD thesis). The University of Melbourne, 2015.
46. Jenney A, Mishna F, Alaggia R, Scott K. Doing the right thing? (Re) Considering risk assessment and safety planning in child protection work with domestic violence cases. Child Youth Serv Rev 2014;**47**:92-101 doi: 10.1016/j.childyouth.2014.07.015[published Online First: Epub Date]|.
47. Angelo M, Isidoro Prado S, Cascaes Cruz Ai, Oliveira Ribeiro M. Nurses' experiences caring for child victims of domestic violence: a phenomenological analysis. Texto & Contexto Enfermagem 2013;**22**(3):585-92 8p
48. MacMillan HL, Fleming J, Jamieson E. Psychiatric assessment of children and adolescents. In: Goldbloom R, ed. Pediatric Clinical Skills. (4th ed). New York: Churchill Linvingstone, 2010:2062–14.
49. VEGA. Trauma- and violence-informed care. Briefing note. Secondary Trauma- and violence-informed care. Briefing note. 2016. http://projectvega.ca/wp-content/uploads/2016/10/VEGA-TVIC-Briefing-Note-2016.pdf.
50. WHO. Update of the Mental Health Gap Action Programme (mhGAP) guidelines for mental, neurological and substance use disorders. http://www.who.int/mental_health/mhgap/guideline_2015/en/: WHO, 2015:72.
51. Stanley N. *Children experiencing domestic violence: a research review*: Dartington: Research in practice, 2011.
52. Stanley N, Humphreys C. *Domestic violence and protecting children : new thinking and approaches*. London ; Philadelphia: Jessica Kingsley Publishers, 2015.
53. Turner W, Hester M, Broad J, et al. Interventions to Improve the Response of Professionals to Children Exposed to Domestic Violence and Abuse: A Systematic Review. Child Abuse Rev 2017;**26**(1):19-39 doi: 10.1002/car.2385[published Online First: Epub Date]|.
54. Zaher E, Keogh K, Ratnapalan S. Effect of domestic violence training: systematic review of randomized controlled trials. Can Fam Physician 2014;**60**(7):618-24, e340-7
55. Garcia-Moreno C, Hegarty K, d'Oliveira AF, Koziol-McLain J, Colombini M, Feder G. The health-systems response to violence against women. Lancet 2015;**385**(9977):1567-79 doi: 10.1016/S0140-6736(14)61837-7[published Online First: Epub Date]|.

For peer review only - http://bmjopen.bmj.com/site/about/guidelines.xhtml

56. Dmytryshyn AL, Jack SM, Ballantyne M, Wahoush O, MacMillan HL. Long-term home visiting with vulnerable young mothers: an interpretive description of the impact on public health nurses. Bmc Nurs 2015;**14**(12)
57. Conrad D, Kellar-Guenther Y. Compassion fatigue, burnout, and compassion satisfaction among Colorado child protection workers. Child Abuse Neglect 2006;**30**(10):1071-80 doi: 10.1016/j.chiabu.2006.03.009[published Online First: Epub Date]|.
58. Slattery SM, Goodman LA. Secondary traumatic stress among domestic violence advocates: workplace risk and protective factors. Violence Against Women 2009;**15**(11):1358-79 doi: 10.1177/1077801209347469[published Online First: Epub Date]|.
59. Munro E. Department for Education. The Munro review of child protection: Final Report. A child-centred system. The Stationery Office Limited, 2011:189.
60. Tehrani N. *Managing trauma in the workplace : supporting workers and organisations*. Hove, East Sussex ; New York: Routledge, 2011.
61. Phillips H, Lyon E, Fabri M, Warshaw C. Promising practices and model programs: trauma-informed approaches to working with survivors of domestic and sexual violence and other trauma http://www.nationalcenterdvtraumamh.org/publications-products/promising-practices-and-model-programs/: National Center on Domestic Violence, Trauma & Mental Health, 2015:39.
62. Ponic P, Varcoe C, Smutylo T. Trauma- (and violence-) informed approaches to supporting victims of violence: policy and practice considerations. http://www.justice.gc.ca/eng/rp-pr/cj-jp/victim/rd9-rr9/p2.html: Government of Canada, Department of Justice, 2016.
63. Hester M. Children protection and domestic violence: findings from a Rowntree/ NSPCC study. In: Hanmer J, Itzin C, Quaid S, Wigglesworth D, eds. Home thruth about domestic violence: feminist influences on policy and practice: a reader. London and New York: Routledge, 2000:96-112.
64. McGee C. *Childhood experiences of domestic violence*. London ; Philadelphia: Jessica Kingsley Publishers, 2000.
65. Mullender A. *Children's perspectives on domestic violence*. London ; Thousand Oaks, Calif.: SAGE, 2002.
66. Radford L, Aitken R, Miller P, Ellis J, Roberts J, Firkic A. Meeting the needs of children living with domestic violence in London. Research report. London: NSPCC and Refuge, 2011:258.
67. Overlien C. Children Exposed to Domestic Violence Conclusions from the Literature and Challenges Ahead. J Soc Work 2010;**10**(1):80-97 doi: 10.1177/1468017309350663[published Online First: Epub Date]|.
68. Cater A, Øverlien C. Children exposed to domestic violence: a discussion about research ethics and researchers' responsibilities. Nordic Social Work Research 2014

4(1):67-79
69. UN. Convention on the Rights of the Child. Treaty Series 20 November 1989; 1577. http://www.refworld.org/docid/3ae6b38f0.html (accessed 21 June 2017).
70. Mayall B. *Towards a sociology for childhood : thinking from children's lives*. Buckingham ; Philadelphia: Open University Press, 2002.
71. Garcia L, Soria C, Hurwitz EL. Homicides and intimate partner violence. Trauma, Vilence & Abuse 2007;**8**(4):370-83 doi: 10.1177/1524838007307294[published Online First: Epub Date]|.
72. Reisenhofer S, Taft A. Women's journey to safety – the transtheoretical model in clinical practice when working with women experiencing intimate partner violence: a scientific review and clinical guidance. Patient Education and Counseling 2013;**93**(3): 536-48 doi: 10.1016/j.pec.2013.08.004[published Online First: Epub Date]|.
73. Humphreys C. Problems in the system of mandatory reporting of children living with domestic violence. Journal of Family Studies 2008;**14**(2-3)
74. Dubowitz H. World perspectives on child abuse: International Society for the Prevention of Child Abuse and Neglect, 2014.

For peer review only - http://bmjopen.bmj.com/site/about/guidelines.xhtml

75. Mathews B. Mandatory reporting laws: their origin, nature, and development over time. In: Mathews B, Bross DC, eds. Mandatory reporting laws and the identification of severe child abuse and neglect. Dordrecht: Springer, 2015:3-25.

76. Varcoe C, Ford-Gilboe M, Smye V, Wathen C, Browne A. VEGA Briefing Note on Trauma- and Violence-Informed Care. 2016. (accessed 09.01.2018).

List of figures

Figure 1. Flow of studies through the review.

Figure 2. Methodological quality of the studies as assessed by the modified Critical Appraisal Skills Programme (M-CASP, score range 0-20). Studies scored >=15 are in the top tertile.

List of online Supplementary files

Supplementary file 1. Search strategy.

Supplementary file 2. Characteristics of the 11 studies (16 papers) synthesised (in chronological order).

Supplementary file 3. Quality appraisal for the included studies.

Supplementary file 4. Descriptive themes.

Supplementary file 5. Interim analytical themes and their definitions.

Supplementary file 6. Final analytical themes supported by raw qualitative data from included studies.

For peer review only

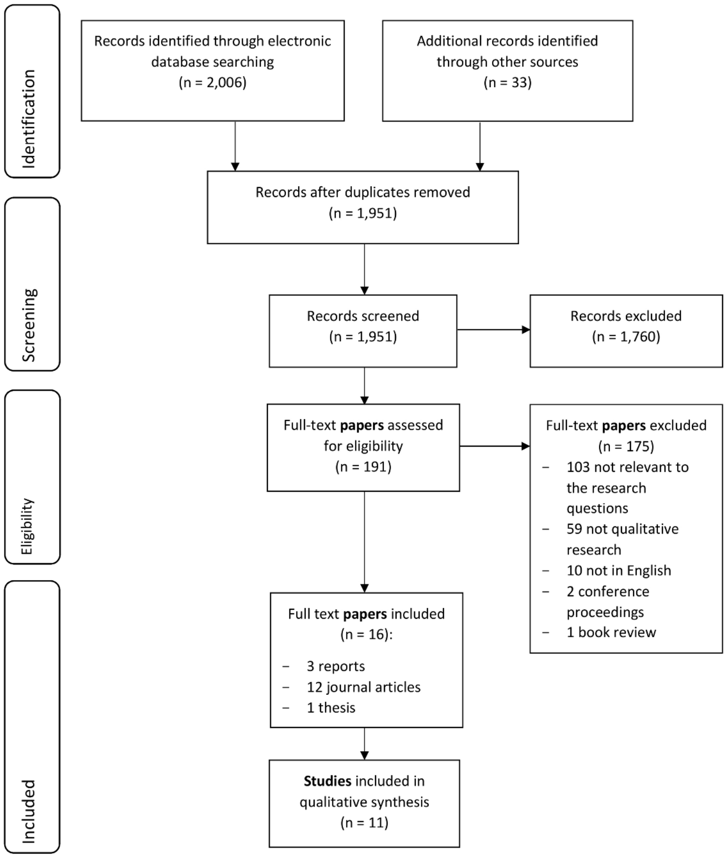

Figure 1. Flow of studies through the review.

210x297mm (300 x 300 DPI)

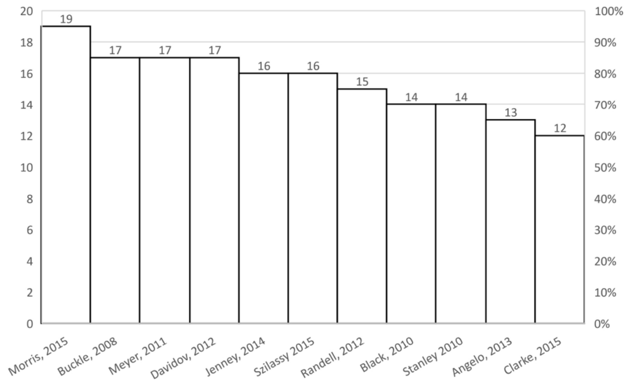

Figure 2. Methodological quality of the studies as assessed by the modified Critical Appraisal Skills Programme (M-CASP, score range 0-20).

Studies scored ≥15 are in the top tertial.

210x297mm (300 x 300 DPI)

Supplementary file 1

Search strategy

Full Boolean search strategy for MEDLINE

Date range of search 1 April 2013 to 28 April 2016.

Date of search 28 April 2016.

----

1 Child Welfare/ or Child, Preschool/ or Mother-Child Relations/ or Father-Child Relations/ or Child Behavior/ or "Child of Impaired Parents"/ or Child/ or Parent-Child Relations/ or Child Psychology/ or Child Reactive Disorders/ or Child Psychiatry/ or Adolescent Psychiatry/ or Adolescent Behavior/ or Adolescent/ or Adolescent Health Services/ or Adolescent Psychology/ or Adolescent Development/ (2612601)

2 (adolesc* or preadolesc* or pre-adolesc* or boy* or girl* or child* or infant* or preschool* or juvenil* or minors or school* or pediatri* or paediatri* or pubescen* or pre-pubescen* or puberty or student* or teen* or young or youth* or school* or high-school or "high school" or college or undergrad* or campus* or classroom*).mp. [mp=title, abstract, original title, name of substance word, subject heading word, keyword heading word, protocol supplementary concept word, rare disease supplementary concept word, unique identifier] (4168331)

3 1 or 2 (4168331)

4 Domestic Violence/ or Spouse Abuse/ or Battered Women/ (12097)

5 (abuse* adj3 (wom*n or partner or spous* or m*n or wife or wives or husband*)).tw. (5458)

6 (battered adj3 (wom*n or partner or spous* or m*n or wife or wives or husband*)).tw. (695)

7 (violen* adj3 (wom*n or partner or spous* or m*n or wife or wives or husband*)).tw. (7703)

8 (marital adj3 (violen* or abuse*)).tw. (297)

9 (famil* adj3 (violen* or abuse*)).tw. (2930)

10 domestic violen*.tw. (4517)

11 (intimate adj3 partner adj3 (violen* or abuse*)).tw. (4273)

12 (interparental adj3 (violen* or abuse*)).tw. (97)

For peer review only - http://bmjopen.bmj.com/site/about/guidelines.xhtml

13 (violen* adj2 (home*1 or household*)).tw. (206)

14 (parent* adj3 (violen* or abuse*)).tw. (1868)

15 or/4-14 (22293)

16 (expose* or exposure).mp. (927096)

17 witnes*.mp. (18418)

18 growing up.tw. (1791)

19 ((child* or adolesc*) adj3 "living with").tw. (1380)

20 16 or 17 or 18 or 19 (946981)

21 3 and 15 and 20 (2401)

22 limit 1 to ed=20130401-20160428 (284370)

23 21 and 22 (426)

For peer review only

Supplementary file 2

Characteristics of the 11 studies (16 papers) synthesised (in chronological order)

N	Study<br>Paper(s)<br>Country	Aim	Setting	Participants, n	Intervention(s)	Data collection	Data analysis	Primary themes relevant to the synthesis	M-CASP score/ Yes-No-Not sure
1	Buckley 2008[a]<br>Buckley 2008 (report) [33]<br>Buckley 2011 (article)<br>[32]<br>Republic of Ireland	Service users' experiences of child protection services	Women's refuges, advocacy groups, family support services, treatment services, counselling service, public redress bodies, community youth project, and statutory child protection service in 16 counties	27 parents<br>20 female, 7 male<br>18 survivors of IPV	Notification by other services<br>Self-disclosure<br>Self-referral<br>First contact and initial response from child protection services	Interviews	Grounded-theory	Positive and negative experiences with child protection services.<br>Impact of those experiences on parents and children.	17-3-0

For peer review only

N	Study Paper(s) Country	Aim	Setting	Participants, n	Intervention(s)	Data collection	Data analysis	Primary themes relevant to the synthesis	M-CASP score/ Yes-No-Not sure
2	Black 2010 [41], US	Recommended practices and obstacles to practices to improve interventions for families suffering from IPV	Varied health care services in one large urban area	Mixed sample of professionals including 24 HCPs and SSPs<br>20 female, 4 male	IPV screening<br>case finding of children's exposure to IPV<br>Initial care of the mother–child dyad	Interviews	Not stated	Individual, social, and system levels obstacles to identifying and responding to children's exposure to IPV.<br>Participants recommendations on how to improve identification and response to children's exposure to IPV.<br>Focus on supporting the mother–child dyad.<br>Challenges of focusing on the	14-6-0

For peer review only

N	Study Paper(s) Country	Aim	Setting	Participants, n	Intervention(s)	Data collection	Data analysis	Primary themes relevant to the synthesis	M-CASP score/ Yes-No-Not sure
								mother-child dyad.	
3	Stanley 2010[a]<br>Stanley 2010 (report) [35]<br>Stanley 2012 (article) [34]<br>UK	Experiences and views of police notifications to children's social services of IPV incidents where children were present/resided in the household	IPV services for women, services for children affected by IPV, children's social services in two local authorities in the North and South of England	19 children: age 10-19; 8 male, 11 female; 16 white British, 1 white/ Asian, 1 white/ black Caribbean, 1 white/ black African.<br>11 parents survivors of IPV: 1 male, 10 female; mean age 38 (25-48); most black minority ethnic (BME), 4 white British.<br>25 SSPs: age 23-65; 4 male, 21 female; 14 front-line, 22 white British, 1 black British, 1 black African, 1 "other";	Notification by police<br>Response from children's social services to the police notifications	Focus groups; interviews	Grounded theory	Clients' positive and negative experiences with children's social services and health professionals when disclosing IPV.<br>Barriers and facilitators to disclosing and acknowledging IPV.<br>Individual, organisational and system level barriers to responding to the police	14-6-0

For peer review only

N	Study Paper(s) Country	Aim	Setting	Participants, n	Intervention(s)	Data collection	Data analysis	Primary themes relevant to the synthesis	M-CASP score/ Yes-No-Not sure
				job experience 1.5-30 years				notifications of children's exposure to IPV.	
4	Meyer 2011 [44] Australia	Help-seeking decisions of IPV victims with dependent children	IPV services, family counselling services in three areas in Southeast Queensland	29 parents survivors of IPV<br>All female<br>Age 21-62 (mean 38, SD 11.3).<br>21 Australian-born, 2 New Zealand-born, 3 UK-born, 1 South African, 1 Malaysian, 1 Filipino.<br>27 had children. 25 children had witnessed the abuse.<br>16 secondary education, 3 in tertiary education, 10 completed tertiary education.	Initial response from child protection services	Interviews	Grounded theory	Gap between client and professional understanding of the dynamics of IPV and response needed.<br>Parents' fear of harm and loss of custody.	17-3-0

For peer review only

N	Study Paper(s) Country	Aim	Setting	Participants, n	Intervention(s)	Data collection	Data analysis	Primary themes relevant to the synthesis	M-CASP score/ Yes-No-Not sure
				7 employed, 2 unemployed, 2 volunteers, 14 home duties, 3 students, 1 retired.<br>26 received government benefits.					
5	Randell 2012 [43]<br>US	Important information to communicate about IPV and how it should be presented in a pediatric emergency department	IPV service in a racially and ethnically mixed city	99 parents survivors of IPV<br>All female<br>36 White, 32 Hispanic, 22 African American, 5 other, 4 not reported.<br>52 high school, 21 degree, 26 not reported.<br>45 currently employed	Identification and response in health care setting	Focus groups	Grounded theory	Content and format of posters about children's exposure to IPV.	15-5-0

For peer review only

N	Study Paper(s) Country	Aim	Setting	Participants, n	Intervention(s)	Data collection	Data analysis	Primary themes relevant to the synthesis	M-CASP score/ Yes-No-Not sure
6	Davidov 2012 [42]<br>US	Issues related to the mandatory reporting of children's exposure to IPV	Community based Nurse-Family Partnership (NFP) programme in four sites	20 NFP mothers survivors of IPV; mean age 21.3; 50% White, 25% Black, 15% Hispanic; 90% single/ never married; years of school 11.7; 80% income<$24,000<br>47 HCPs:<br>25 NFP nurses; mean age 46.2; 92%>=bachelor's degree; nursing experience 19.7 (5-38); years in NFP 4.4.<br>22 community stakeholders including 4 from hospital or public health services; work	Mandatory reporting of children's exposure to IPV by NFP nurses	A secondary analysis of focus groups and interviews	Retrospective interpretation	Professional and patient uncertainty regarding reportable children's exposure to IPV.<br>Mothers' fears of losing custody of children.<br>Strategies nurses can use to ease mothers' fear.<br>Negative client experiences with child protection services.	17-3-0

For peer review only

N	Study Paper(s) Country	Aim	Setting	Participants, n	Intervention(s)	Data collection	Data analysis	Primary themes relevant to the synthesis	M-CASP score/ Yes-No-Not sure
				experience 9.2 years; mean age 50.5 years.					
7	Angelo 2013 [47] Brazil	Experience of the nurses in their care of child victims of IPV	Pediatric emergency, intensive care and inpatient units in São Paulo	15 HCPs<br>14 female, 1 male; ages 27-48, time since graduation 3-12 years	Case finding<br>Initial response	Interviews	Motivational theory	Identification of children's exposure to IPV through attention to signs and symptoms followed by verification.<br>Ambivalent feelings towards parents of children exposed to IPV.<br>Protective professional attitude towards children.	13-7-0
8	Jenney 2014 [46] Canada	How the voices of women with experience of	Child protection services in five Ontario child	17 CPS workers: 12 females, 5 males; job experience >6 years;	Notification by other services	Focus group; interviews	Grounded theory	The process of interaction between worker	16-4-0

For peer review only

N	Study Paper(s) Country	Aim	Setting	Participants, n	Intervention(s)	Data collection	Data analysis	Primary themes relevant to the synthesis	M-CASP score/ Yes-No-Not sure
		IPV are translated into child protection service workers' interventions	welfare agencies	qualifications - BA (3), BSW (8), CYW (1), and MSW (5); 9 did not have DVA training, 4 had training in both risk assessment and safety planning.<br>11 parent survivors of IPV: all female; age 26-49 years; 5 immigrant/refugee; 8 on < $25,000 annually; 5 College/University education, 2 high school, 4 grades 9–11.	Self-referral<br>Response from child protection services			and client during child protection service investigation.<br>Gap between client and professional understanding of the concept of safety and the impact of IPV on children.	
9	Interview study within the RESPONDS Project[a]	Responses to disclosure of IPV when children are exposed and the dilemmas	General practices in six sites across the north and south	69 HCPs<br>42 general practitioners, 12 practice nurses and	Case-finding<br>Notification by other services	Interviews	Thematic analysis using framework	Engaging directly with children at medical consultation.	16-4-0

For peer review only

N	Study Paper(s) Country	Aim	Setting	Participants, n	Intervention(s)	Data collection	Data analysis	Primary themes relevant to the synthesis	M-CASP score/ Yes-No-Not sure
	Szilassy 2015 (report) [37] Larkins 2015 (paper) [39] Szilassy 2016 (paper) [38] Drinkwater 2016 (paper) [36]<br><br>UK	and challenges general practice clinicians face when confronted with children's exposure to IPV	of England and the Midlands	15 practice managers<br>48 female, 21 male<br>Age: 21-34 – 10; 35-44 – 13; 45-54 – 30; 55-64 – 10; not known – 10<br>Experience managing domestic violence (number of cases): >5 – 5; a few – 14; 1 – 2, none – 26; none but aware of case at surgery - 7<br>IPV service provision: sparse – 28; established – 41<br>Location: metropolitan – 19; urban – 27; semi-rural – 23	Initial response from general practice clinicians			Professional uncertainty about responding to children's exposure to IPV.<br>Poor inter-agency work.	

For peer review only

N	Study Paper(s) Country	Aim	Setting	Participants, n	Intervention(s)	Data collection	Data analysis	Primary themes relevant to the synthesis	M-CASP score/ Yes-No-Not sure
				Region: north – 21; Midlands – 33; south – 33					
10	Clarke 2015 [40] UK	Practitioners' perceptions of children's experiences of IPV. Experiences of responding to the needs of children	Varied services in Wales	Mixed sample of 54 professionals from social services, health, education, police, probation, housing, IPV services and third sector organisations 5 parent survivors of IPV: all female	HCPs and SSPs responses to disclosure	Focus groups; interviews; case studies; meetings observations	Thematic analysis	Organisational and system level barriers to engaging directly with children and inter-agency work.	12-8-0
11	Morris 2015 (thesis) [45] Australia	Perspectives on children's safety and resilience when they live with IPV	General practices in Victoria	18 parent survivors of IPV: all female; mean age 43 (28-56) years; 2 born overseas. 23 children to 16 mothers survivors of IPV: mean age 14 (8-	Screening Case-finding Initial response from general practice clinicians	Interviews; focus groups	Hermeneutic Phenomenological analysis	Raising matters of safety or psychological well-being in the consultation. The type of relationships children and mothers expect	19-1-0

N	Study Paper(s) Country	Aim	Setting	Participants, n	Intervention(s)	Data collection	Data analysis	Primary themes relevant to the synthesis	M-CASP score/ Yes-No-Not sure
				24) years; 2 born overseas				to have with health professionals if they are to feel comfortable discussing family violence.<br>When, with whom and how safety conversations could be initiated.	

Note. a Indicates where more than one paper relates to a single study. IPV – intimate partner violence. HCPs – health care professionals. SSPs – social service professionals; NFP – Nurse-Family Partnership. CPS – child protection service; M-CASP – the modified version of the Critical Appraisal Skill Programme.[21 22] For studies reported in multiple papers, the total score was derived based on data extracted from all papers.

For peer review only

Supplementary file 3

Quality appraisal for the included studies (n = 11)

**Quality appraisal criteria**	**Yes**	**Studies**
A. Appropriateness of research methodology and design		
1. Does the research seek to interpret or illuminate the actions and/or subjective experiences of research participants?	11	[33 35 37 40-47]
2. Is qualitative research the right methodology for addressing the research goal?	11	[33 35 37 40-47]
3. Is the research design appropriate to address the aims of the research?	11	[33 35 37 40-47]
4. Has the researcher justified the research design	5	[33 42 43 45 46]
B. Ethical considerations		
5. Did the researcher use two or more of the following strategies to ensure ethical issues have been taken into consideration (is there are sufficient details of):<br>a. how the research was explained to participants for the reader to assess whether ethical standards were maintained;<br>b. did the researcher discuss issues raised by the study, such as issues around informed consent or confidentiality or how they have handled the effects of the study on the participants during and after the study;<br>c. was approval sought from an ethics committee)?	8	[33 35 37 41-45]
C. Credibility (akin to internal validity)<br>Do participants and those with similar experiences recognize the experiences contained with the study?		
6. Did the research use one or more of the following strategies to establish credibility:<br>a. has the researcher discussed saturation of data;<br>b. attempt to triangulate data by using different data collection methods;<br>c. member checking to see if participants agreed with the interpretations of the researcher;<br>d. peers or consultants experienced in qualitative research review their coding process;<br>e. full descriptions of member's words in their final paper?	11	[33 35 37 40-47]
D. Transferability (akin to external validity)		

For peer review only

Quality appraisal criteria	Yes	Studies
How does one determine the extent to which the findings of the study are applicability in other contexts or with other participant types?		
7. Did the researchers use any of the following strategies to establish transferability (use of dense description of the population studied through descriptions of demographics and geographic boundaries of the study? Note: the author must describe at least two specific sample descriptors (e.g. age range, gender, setting from which sample was selected, SES, etc.)	8	[33 35 37 42-44 46 47]
E. Consistency (akin to reliability)<br>Can another researcher follow the decision trail used by the researcher?		
8. Did the researcher use any of the following strategies to establish the purpose of the research:<br>a. was there a clear statement of aims of the research;<br>b. what was the goal of the research;<br>c. why was it thought important<br>d. its relevance?	11	[33 35 37 40-47]
9. Was the recruitment strategy appropriate to the aims of the research? (e.g. does the population from which the sample was selected resonate with the research objectives, was the sample selection ethically implemented).	10	[33 35 37 40-42 44-47]
10. Did the researcher explain how participants were selected?	9	[32 37 40 42-47]
11. Did they explain why the participants they selected were the most appropriate to provide access to the type of knowledge sought by the study	4	[32 37 42 45]
12. Were there any discussions around recruitment (e.g. why some people chose not to take part)?	4	[41 44-46]
13. Was the setting for data collection justified?	8	[35 37 40 42-46]
14. Is it clear how data were collected (e.g. focus group, semi-structured interview etc.)?	11	[33 35 37 40-47]
15. Did the researcher justify the methods chosen?	3	[33 44 45]
16. Did the researcher make the methods explicit (e.g. for interview method, is there an indication of how interviews were conducted, or did they use a topic guide)?	10	[33 35 37 41-47]
17. If the form of data is clear (e.g. tape recordings, video material, notes etc.)?	11	[33 35 37 40-47]
18. Did the researcher explain how the data were reduced or transformed for analysis?	11	[33 35 37 40-47]

For peer review only

Quality appraisal criteria	Yes	Studies
19. Did they discuss their interpretation and presentation of their findings?	11	[33 35 37 40-47]
F. Neutrality (akin to objectivity)<br>Did overall credibility, transferability, and consistency occur?		
20. Did the researcher use one or more of the following strategies to ensure neutrality (has the relationship between researcher and participants been adequately considered; has the researcher critically examined their own role, potential bias and influence during the formulation of the research questions or data collection, including sample recruitment and choice of location; did the researcher discuss how they responded to events during the study and whether they considered the implications of any changes in the research design; did the researcher employ field notes to record their personal reactions and biases after each interview/focus group; did they make a conscious effort to follow rather than lead the direction of interviews/focus groups)?	2	[41 45]

Note. Quality was appraised using the modified version of the Critical Appraisal Skill Programme (M-CASP), score range 0-20.[17]

For peer review only

Supplementary file 4

Descriptive themes

Descriptive theme with subthemes		Stakeholder group, study		
		**Children**	**Parents**	**Professionals**
		**1. Experiences of identification of children's exposure to IPV**		
1.1. Approach	1.1.1. How to ask	All children felt that HCPs should initiate a conversation about safety and well-being by giving permission, space and time to discuss sensitive matters.[45]	All mothers felt that HCPs should initiate a conversation about safety and well-being by giving permission, space and time to discuss sensitive matters.[45]	
	1.1.2. When to ask	Most children found it acceptable when HCP initiates enquiry about 'safety at home' from the presenting problem.[45]		Most HCPs described a positive experience of enquiring from the presenting symptoms of children's exposure to IPV or following the disclosure of IPV by mother.[37 41 42 47]
		One young man preferred when HCP asks about 'safety at home' regardless of the presenting problem.[45]		Several practitioners suggested screening for IPV as part of routine pediatric exam.[41]
	1.1.4. Police notification to children's social services of IPV incidents when		Most mothers were not aware of the police notification.[35]	Some SSPs from children's social services thought that not all incidents of IPV with children present/ resided in the household need to be referred to

Descriptive theme with subthemes		Stakeholder group, study		
		Children	Parents	Professionals
	children were present/ resided in the household			them. However, they thought that they should be informed of such incidents.[35]
				Some SSPs from children's social services found police notifications inadequate due to the lack of details on children's exposure to IPV.[35]
				SSPs from children's social services found that "filtering out" police notifications to assess whether the family requires further intervention is too time- and labour-consuming.[35]
**2. Experiences of initial response to children's exposure to IPV**				
	2.1. Focus			Most HCPs felt that they should treat the mother-child dyad as a single entity where the needs of the mother were consistent with the child's health and safety needs.[37 41]
				Some HCPs felt conflicted when they had to prioritise the child's needs over mother's needs and confidentiality.[37 41]
				Some HCPs and most SSPs felt that if a child is at risk, they have to prioritise his/ her safety over the mothers' needs and confidentiality.[35 37]
	2.2. Emotional support		Mothers valued when SSPs provided a lot of encouragement.[46]	Some HCPs were positive about providing emotional support and 'leaving the door open' to

Descriptive theme with subthemes		Stakeholder group, study		
		Children	Parents	Professionals
				children to come back with any problems.[37 39 47]
	2.3. Education		Mothers valued information materials on the impact of IPV on children and IPV resources.[43]	HCPs and SSPs emphasised the importance of educating mothers about the impact of IPV on children and professional responsibilities in cases of children's exposure to IPV. Professionals used education to increase mother's engagement in services.[35 37 41 42 46]
				HCPs emphasised the importance of educating mothers about the purpose of the home visitation programme and children's social services. Professionals saw education as a pathway to easing mothers' fears of child removal as a result of professionals' involvement.[42]
				SSPs expressed mixed views on the acceptability of sending parents materials about impact of IPV on children in response to the police notification without any other intervention to families.[35]
2.4. Safety	2.4.1. Understanding of safety		Mothers thought that involvement of SSPs resulted in increased risk of abuse escalation and child removal.[32 44 46]	SSPs understood women's and children's safety as being out of the abusive relationship and believed that their involvement resulted in increased safety for both mothers and children.[44 46]

Descriptive theme with subthemes	Stakeholder group, study		
	Children	Parents	Professionals
		Some actions that mothers perceived as preventing abuse escalation and increasing child safety (e.g., staying with perpetrator, not seeking help) were seen by SSPs as increasing risk for children.[44 46]	Some actions that mothers perceived as increasing child safety were seen by SSPs as increasing risk for children.[44 46]
		Mothers did not feel safer after they complied with SSPs' requirements and left the abusive partner.[44 46]	
		Women wanted to talk with children's social workers about safety and safety planning.[46]	
2.4.2. Assessment of risk	One child suggested that HCPs need to ask children about their safety in all the environments that a child inhabits (e.g., all households post-separation, school).[45]		
			SSPs and HCPs favoured varied approaches to risk assessment (e.g., observation, information gathering from mother and other professionals, gut feeling, previous experiences).[35 37 46]

Descriptive theme with subthemes	Stakeholder group, study		
	Children	Parents	Professionals
			Some SSPs found standardised tools helpful for risk assessment (e.g., the Barnardo's Domestic Violence Risk toll).[35]
			Some SSPs saw the existing risk assessment as a bureaucratic exercise and believed that it does not measure real risk from the perpetrator[35 40 46]
2.4.3. Safety planning		Most mothers were not involved in safety planning with their SSPs therefore could not comment on its acceptability.[46]	SSPs found undertaking safety planning with both children and mothers to be acceptable.[35]
			Although SSPs understood that they could not change women, they believed that leaving an abusive partner is the most effective form of safety planning.[46]
			Some SSPs felt that they monitor the risk instead of doing direct safety planning with mothers and children and wanted more direct engagement with them.[35 40]
			Some HCPs found it acceptable to undertake safety planning with women who were not ready to leave the perpetrator.[41]

Descriptive theme with subthemes	Stakeholder group, study		
	Children	Parents	Professionals
2.5. Signposting	Children appreciated when social workers signposted them to other services.[35]	Mothers appreciated when health visitors signposted them to other services.[35]	Two GPs felt comfortable when giving children information about services they could access.[37]
			Some HCPs and SSPs felt comfortable providing mothers with information about IPV resources. They saw signposting as a mechanism to facilitate engagement in services.[35 37 46]
2.6. Documenting			Most HCPs reported uncertainty and confusion about recording policies and practices on children's exposure to IPV. The uncertainty was reported at local, region and national levels.[36 37 41 42]
			HCPs feared possible breach confidentiality of parents exposed to IPV by recording children's exposure in child or parent medical records.[36 37]
2.7. Reporting			HCPs described uncertainty and confusion about their reporting responsibilities especially in cases of child exposure to psychological IPV and non-direct physical IPV.[37 41 42]

Descriptive theme with subthemes	Stakeholder group, study		
	Children	Parents	Professionals
		Mothers felt uncertain about reporting responsibilities of HCPs and feared that HCPs could initiate child removal.[42]	HCPs emphasised the importance of explaining their reporting responsibilities and procedures to mothers. Professionals use such education to ease mothers' fears of child removal, to increase engagement with services, and to justify breach of the mothers' confidentiality after IPV disclosure.[37 42]
**3. Factors that facilitated identification and initial response to children's exposure to IPV**			
3.1. Mother's readiness to disclose and engage		Mothers reported past experiences when they were not ready to acknowledge the impact of IPV on their children, disclose IPV and engage with services.[35 43 44]	HCPs and SSPs believed that the approach to identification and initial response should match mother's readiness to disclose IPV and engage with services.[35 41 46]
3.2. Patient-professional relationship	Most children emphasised the importance of 'knowing and being known' to the doctor, which made them feel comfortable to talk about personal issues.[45]	Most mothers emphasised the importance of a long-term trusting relationship with the same professional for IPV disclosure and engagement with services.[32 44-46]	Most HCPs and SSPs emphasised the importance of a long-term trusting relationship with mother for IPV disclosure and engagement with services.[41 46]
		Mothers suggested that they could be role models for their children in developing trust and building	Only one HCP suggested that existing relationships could enable proactive and direct engagement with children.[37 39]

Descriptive theme with subthemes		Stakeholder group, study		
		**Children**	**Parents**	**Professionals**
			relationship between child and doctor.[45]	
				Some HCPs thought that referral to children's social service can worsen their relationship with a parent who discloses IPV.[37 39 41]
				Some HCPs feared that professionals with strong relationships with their patients are more likely to develop vicarious trauma.[41]
	3.3. Positive professional attitude	Children described preferred professionals as good at listening, making them feel that they were welcome back, helping them practically.[34 35 45]	Mother appreciated HCPs and SSPs who took them seriously, validated their accounts, ensured confidentiality and helped practically.[32-35 42 44 46]	HCPs emphasised the importance of nonthreatening and non-judgemental attitudes with frequent reassurance of confidentiality when responding to IPV disclosure.[37 41 42]
3.4. Engaging directly with [illegible]	3.4.1. Talking to children at consultation	Children appreciated when professionals talk directly to them, instead of to their mothers.[34 35 45]	Mothers stressed the importance of giving each child a voice and addressing each child's needs at a medical consultation.[45]	Most HCPs expressed concerns in children's competence as informants and believed parents more than children.[37 39]
				Some HCPs expressed concern about their lack of competence in communicating directly with children.[37]

Descriptive theme with subthemes	Stakeholder group, study		
	Children	Parents	Professionals
			A few HCPs who directly engaged with children were female general practitioners in affluent areas with high levels of IPV service provision.[39]
			SSPs felt confident talking to children when assessing and intervening in families with IPV.[35]
			SSPs felt that they should listen to the child more when assessing and intervening in families with IPV.[40]
3.4.2. Appropriate age for talking to children at consultation	Most children regardless of age preferred their mother (trusted adult) to be present in a medical consultation.[45]	Mothers found it acceptable if doctors speak to children from the age of five. They believed that children exposed to IPV mature quicker than non-exposed children.[45]	There was no age above which clinicians consistently agreed it would be acceptable to talk to children about IPV; some were hesitant about even talking to teenagers.[37 39]
		Mothers preferred doctors to use non-verbal methods when communicating with young children.[45]	
		Mothers of adolescents believed that their children would find it difficult to talk to doctors because of teenage behavioural traits.[45]	

Descriptive theme with subthemes		Stakeholder group, study		
		Children	Parents	Professionals
	3.4.3. Seeing children alone	Some children felt that they should be of adolescent-age before speaking alone with the doctor about their safety and well-being.[45]		Most general practice clinicians felt that they should not see children alone at consultation.[37 39]
		Most children wanted their mothers (trusted adult) to be present at the medical consultation.[45]		Few general practice clinicians who would see children alone thought that it is acceptable to seek permission from the non-abusive parent and from the child without describing how it should be done.[37]
				SSPs regularly saw children alone when assessing risk, although they found it challenging to talk to children alone.[35]
**4. Reasons for not identifying/ disclosing children's exposure to IPV and not engaging with services**				
	4.1. Awareness of children's exposure to IPV		Mothers recognised that it took long time to acknowledge that IPV had an impact on their children.[32 35 43 44 46]	Most HCPs felt uncertain and confused about what constitutes children's exposure to IPV in cases of psychological IPV and non-direct physical IPV and how to respond to such cases.[37 42]
			Mothers felt that SSPs are unaware of the dynamics of IPV and risks involved.[32 34 35 44 46]	SSPs believed that mothers' underestimating of the impact of IPV on children was a barrier to disclosure and engagement with services.[34 35 46]

Descriptive theme with subthemes		Stakeholder group, study		
		Children	Parents	Professionals
4.2. Perceived role	4.2.1. Someone else's	Most children believed that HCPs' role is to treat illness and monitor their health and well-being, although health was narrowly defined as physical (e.g., injuries, vaccination).[45]	Mothers believed that HCPs' role is to treat illness and monitor their health and well-being, although health was narrowly defined as physical (e.g., injuries, vaccination).[45]	HCPs believed that Identifying and responding to children's exposure to IPV is someone else's role (e.g., health visitor, social worker, school nurse).[35 37]
		Two children recognised a potential role for HCPs in caring about children beyond physical illness.[45]		
				HCPs and SSPs believed that it is a mother's role to seek help and stop IPV.[35 37 40 42 44 46 47]
	4.2.2. Threatening and punitive	Children perceived children's social service's role as threatening and punitive.[34 35]	Mothers perceived children's social service's role as threatening and punitive.[34 35 42 46]	HCPs and SSPs perceived children's social service's role as threatening and punitive and recognised that mothers and children felt the same.[34 35 42]
				SSPs perceived health visitors as less threatening and more acceptable to families.[35]
	4.2.3. Uncertainty		Some mothers felt confused and uncertain about the role of children's social services which led to anxiety and avoidance of HCPs and SSPs.[34 35]	

Descriptive theme with subthemes	Stakeholder group, study		
	Children	Parents	Professionals
4.2.4. Mandatory reporting			HCPs felt confused and uncertain about their role as mandated reporters of children's exposure to IPV, especially in cases of psychological IPV and non-direct physical IPV.[42]
		Mothers perceived all HCPs as mandated reporters of children's exposure to IPV which led to anxiety and avoidance of HCPs.[42]	HCPs recognised that their role as mandated reporters prevented mothers from disclosing IPV and engaging with services.[42]
4.3. Fears	Children feared being removed from the home and taken into care.[34 35 40]	Mothers feared the removal of children by social services.[32-35 42 44 46]	HCPs and SSPs recognised that varied fears prevented mothers and children from disclosing IPV and engaging with services.[35 42 46]
	Children feared the negative consequences of IPV disclosure (e.g., escalation of abuse, involvement of children's social services).[40 45]	Mothers feared the negative consequences of IPV disclosure (e.g., escalation of abuse, involvement of police and children's social service).[44 45]	HCPs feared the negative consequences of identifying and responding to children's exposure to IPV (e.g., escalation of abuse, misunderstanding, involvement of children's social service, threats from parents, damage to the doctor-patient relationship, legal consequences).[35-37 39 41 47]
		Mothers feared HCPs because of their mandated reporter responsibilities.[42]	HCPs recognised that mothers feared them because of their mandated reporter responsibilities.[42]

Descriptive theme with subthemes		Stakeholder group, study		
		Children	Parents	Professionals
	4.4. Stigma	Children recognised the stigma, shame, and embarrassment associated with disclosing IPV.[34 35]	Mothers recognised the stigma, shame and embarrassment associated with disclosing IPV.[34 35 43 45]	
4.5. Influences from the wider environment	4.5.1 Social and cultural factors		Mothers who were non-native speakers expressed the need for information on children's exposure to IPV in their native language.[43]	HCPs and SSPs recognised that language and cultural norms and stereotypes make it difficult to engage with mothers from ethnic minorities.[35 41]
			Immigrant mothers wanted materials on children's exposure to IPV that address the immigration issues and culture-specific fears of IPV disclosure.[43]	
	4.5.2. Healthcare and social care system factors			HCPs and SSPs perceived the lack of contact time with patients/ clients as a major barrier to identifying and responding to children's exposure to IPV.[37 40 41]
				HCPs did not perceive children's exposure to IPV as their top priority.[37]
				HCPs and SSPs highlighted that their work with children was negatively affected by the cutbacks and constant reforms in home visitation services and social services.[35 37]

Descriptive theme with subthemes		Stakeholder group, study		
		Children	Parents	Professionals
				HCPs and SSPs highlighted the lack of services for all family members which prevented them from asking about and responding to children's exposure to IPV.[35 37 41]
		A few children suggested that police and social workers should communicate better in cases of IPV to ensure children's safety and adequate support.[34 35]		HCPs and SSPs were concerned with the lack of communication between the different providers and expressed a need for better communication and a more coordinated response to children's exposure to IPV.[35 37 40 41]
**5. Psychological consequences of identification and initial response to children's exposure to IPV**				
	5.1. Emotional burden	Children felt disappointed and powerless as a result of negative experiences with SSPs.[34 35]	Parents felt disappointed, stressed, hopeless and frustrated as a result of negative experiences with SSPs.[32-35 44]	
		Children thought that involvement with SSPs was 'stressful' for their mothers.[35]		
			Mothers felt embarrassed and guilty when acknowledging the impact of IPV on their children and when disclosing IPV to professionals.[34 35 43 45]	HCPs and SSPs felt frustrated and conflicted towards mothers who did not follow their advice.[41 46 47]

Descriptive theme with subthemes	Stakeholder group, study		
	Children	Parents	Professionals
			HCPs felt conflicted when changing the focus from the mother-child dyad to the individual child.[37 41]
			HCPs and SSPs felt uncomfortable about pressuring mothers to leave the perpetrator to protect children, but thought that they had few options in situations where mothers and children continued to live with the perpetrator.[37 41]
5.2. Vicarious trauma			HCPs feared the possibility of burnout and hardening towards women and children.[41 47]
	**6. Training and resources suggested**		
6.1. Professional training			HCPs preferred interactive training built upon real cases.[37 38]
			HCPs asked for training on communication with children.[37 38]
			HCPs and SSPs wanted to learn about other services' roles and responsibilities in responding to children's exposure to IPV.[35 37 38]
6.2. Information materials		Mothers favoured single posters in pediatric settings about signs of IPV, effect of IPV on children and local IPV services.[43]	HCPs found IPV posters in health care settings to be acceptable in facilitating IPV disclosure and self-referral to IPV services.[41]

Descriptive theme with subthemes	Stakeholder group, study		
	Children	Parents	Professionals
		Mothers who were non-native speakers favoured information materials in their native language.[43]	HCPs wanted local signposting materials on IPV services.[37 38]
		Mothers preferred information materials with positive non-judgemental message, representing ethnic and social diversity[43]	HCPs wanted materials on policies and procedures about mandatory reporting of children's exposure to IPV.[42]

Note. IPV – intimate partner violence. HCPs – health care professionals. SSPs – social service professionals….. – converging perspectives. …. – diverging perspectives. … - conflicting perspectives.

For peer review only

Supplementary file 5

Interim analytical themes and their definitions

Interim analytical theme with subthemes	Stakeholder group, study			
	Children	Mothers	HCPs	SSPs
**1. Converging perspectives on satisfactory strategies for identification and initial response to children's exposure to IPV**				
**1.1. Facilitators of acceptable identification and initial response**				
**1.1.1. Patient-professional relationship.** Some children, most mothers and most professionals highlighted that central to any work around children's exposure to IPV were aspects of the patient-professional relationship. When describing a good relationship, stakeholders talked about the long-term process of relationship building, continuity of contact, trust and effective communication.	[45]	[32 44-46]	[37 41]	[46]
**1.1.2. Positive professional attitudes and skills.** Patients/ clients and HCPs described how a non-judgemental non-threatening attitude, showing respect, validation of patients' accounts and reassurance of confidentiality facilitated disclosure and engagement with services.	[35 45]	[32 35 42 44 46]	[37 41 42]	
**1.1.3. Safety & comfort.** Good patient-professional relationships enabled children and mothers to feel safe and comfortable when discussing sensitive issues.	[45]	[45]		
**1.1.4. Individual mother's readiness.** Mothers and professionals suggested that the individual mother's readiness to disclose IPV and seek help should be considered when asking about IPV and responding to disclosure.		[43 44]	[41]	[35 46]
**1.1.5. Patient materials.** Mothers and HCPs thought that printed materials for patients about the effect of IPV on children and local IPV resources should be displayed in health-care settings to facilitate IPV disclosure by mothers and provide information for self-help.		[43]	[41]	

For peer review only

Interim analytical theme with subthemes	Stakeholder group, study			
	Children	Mothers	HCPs	SSPs
**1.2. Identification process**				
**1.2.1. Consultation atmosphere.** Children and mothers desired permission, space and time to discuss sensitive issues with HCPs.	[45]	[45]		
**1.2.2. Approach to enquiry.** Most children and HCPs preferred a case-based approach (from presenting symptoms).	[45]		[37 41 42 45 47]	
**1.2.3. Phased inquiry.** Patients and professionals were positive about HCPs initiating the enquiry. They preferred professionals to ask about children's exposure to IPV as a 'safety-at-home' issue – starting from the presenting symptoms, through questions about general well-being and the family situation to questions about feeling safe at home.	[45]	[45]	[37 41 42 47]	
**1.3. Initial response process**				
**1.3.1. Emotional support.** Mothers and professionals were positive about providing a lot of encouragement and emotional support to both children and mothers.		[46]	[37 47]	
**1.3.2. Patient/client education.** Mothers and professionals were positive about patient/client education about the impact of IPV on children, IPV dynamics, professional roles and duties. Professionals highlighted the importance of considering women's safety when sending letters about the impact of IPV on children.		[43]	[37 41 42]	[35 46]
**1.3.3. Signposting.** Mothers, children, and professionals were positive about signposting mothers and children to IPV services.	[35]	[35]	[37]	[35 46]
**2. Converging perspectives on barriers to satisfactory identification and initial response to children's exposure to IPV and proposed solutions**				
**2.1. Fears.** All stakeholder groups shared varied fears. The fear of abuse escalation, involvement of children's social services and child removal were mentioned by all groups. Additional professional fears of	[35 40 45]	[32 35 42 44 46]	[37 41 42 47]	[35 46]

For peer review only

Interim analytical theme with subthemes	Stakeholder group, study			
	Children	Mothers	HCPs	SSPs
negative consequences included threats from parents, negative impact on the therapeutic relationship with parents and legal consequences. Professionals used education to ease mothers' fears.				
**2.2. Gaps in knowledge and skills**				
**2.1.1. Low awareness.** All stakeholder groups demonstrated low awareness about children's exposure to IPV, especially psychological and non-direct physical IPV and delegated responsibilities of identifying and responding to somebody else.	[35 45]	[32 35 43-46]	[37 42]	[35 46]
**2.1.2. Communicating with children.** HCPs wanted to improve their communication skills in talking directly to children during medical consultations.			[37]	
**2.1.3. Documenting.** Professional described uncertainty and confusion regarding how and where to document children's exposure to IPV.			[36 41 42]	
**2.1.4. Mandatory reporting.** Mothers' uncertainty and confusion about HCPs' duties as mandated reporters mirrored professional uncertainty and confusion, especially in cases of children's exposure to psychological and non-direct physical IPV.		[42]	[37 41 42]	
**2.2. Emotional burdens.** All stakeholders were emotionally involved in the work of identifying and responding to IPV. Clients reported feeling threatened and pressured by SSPs to leave the perpetrator without adequate support provided. Professionals described psychological distress caused by ambivalent feelings towards mothers when dealing with children's exposure to IPV and were concerned that involvement in helping abused women and their children could cause vicarious trauma.	[35]	[32 35 46]	[37 41 47]	[35]
**2.3. System level factors**				
**2.3.1. Language and culture.** Stakeholders described mothers' immigration status, community norms and poor English as additional barriers to identifying and responding to children's exposure to IPV.		[43]	[41]	[35]

For peer review only

Interim analytical theme with subthemes	Stakeholder group, study			
	Children	Mothers	HCPs	SSPs
**2.3.2. Inter-agency work.** Stakeholders were similarly concerned with the lack of communication between different providers and expressed a need for a better communication, information sharing and a more coordinated approach when responding to children's exposure to IPV.	[35]		[37 41]	[35 40]
**2.3.3. Stretched services.** Professionals repeatedly mentioned: (i) lack of contact time with patients/clients, (ii) high demand and competing priorities, (iii) constant reforms to health care and social services, and (iv) shortage of services for all family members.			[37 41]	[35 40]
**3. Suggested training and resources** Professional training and resources. Professionals favoured a case-based training, wanted to improve their communication skills, learn about the roles of and responsibilities of other services and highlighted the importance of inter-agency collaboration. HCPs requested clear instructions on mandatory reporting and local signposting materials			[36 37 41 42]	[35]
**4. Diverging perspectives on satisfactory initial response**				
**4.1. Safety.** Mothers and professionals disagreed on the understanding of safety for the child. Actions perceived by mothers as increasing children's safety were seen by SSPs as jeopardising it. Most SSPs believed that their involvement increased children's safety and required mothers to leave the perpetrator. In contrast, most mothers thought that SSPs' involvement posed a threat to the child through leading to IPV escalation and child removal. Women did not feel safer after leaving the perpetrator because of potential escalation of abuse and child contact.		[32 44 46]		[44 46]
**4.2. Talking directly to children.** While most recipients of care were positive about professionals talking directly to children from a young age and addressing their individual needs, most HCPs did not see children as patients on their own and assessed children's needs through a proxy adult. Children and HCPs had	[34 35 45]	[45]	[37]	[35 40]

For peer review only

Interim analytical theme with subthemes	Stakeholder group, study			
	Children	Mothers	HCPs	SSPs
conflicting views about the appropriate age for talking to children about IPV and about appropriateness of seeing children alone.				
**5. Conflicting perspectives on satisfactory identification and initial response**				
**5.1 Shifting focus.** HCPs and SSPs had conflicting perspectives on the focus of their responses – child vs mother-child dyad/family. Although all agreed that the focus should switch to the child when he/she is at risk of harm, some felt conflicted when prioritising children's safety and needs over mother's' safety.			[37 41]	[35]
**5.2. Assessment of risk.** While children wanted to be involved in risk assessment and some SSPs found it acceptable to talk to children when assessing the risk, most professionals were not satisfied with the existing risk assessment process and saw it as a ticking box exercise.	[45]		[37]	[35 40 46]
**5.3. Safety planning.** While some HCPs found it acceptable to undertake safety planning with women who were not ready to leave the abusive partner, most SSPs saw leaving the abusive partner as the best safety planning for the child. SSPs had mixed views on the current safety planning process. Mothers wanted better communication with SSPs when undertaking safety planning.			[41]	[35 40 46]

Note. IPV – intimate partner violence. HCPs – health care professionals, SSPs – social service professionals.

For peer review only

Supplementary file 6

Final analytical themes supported by raw qualitative data from included studies

Theme	Subtheme	Participant quotation	Authors interpretation quotation
1. Precursors for acceptable identification and response to children's exposure to IPV	1.1. Satisfying and sustainable relationship	"Have confidence around them...I guess it depends how much you know your doctor. It's just depending on how well you know 'em. (Zoe, 12)"[45]<br><br>"Well in domestic violence I find that it's always the relationship that we have with the client, with the mom in this case — the stronger our relationship with the mom and the more we can understand her, understand her world through her eyes instead of imposing our view, we get them more on board. Once we can get that trust, then we can work together. [Worker #11]"[46]	"In common with participants in the wider study, they [parents] placed a lot of value on the type of relationship they could build up with workers [child protection workers] and the degree of trust that developed thereafter."[32]
	1.2. Desired professional attitudes and skills	"My doctor was really good, like once I went there and she was like really concerned and she sent me to someone else and then she told me to come back in a week so she could check on me and then, you know...like she kept making appointments so she could see me again and again. So it's good when doctors care like that...Makes you want to then	"Where service users reported positive experiences with the child protection services, it was normally when they felt they had been listened to fairly and believed. They also appreciated it when workers took direct action."[32]

For peer review only - http://bmjopen.bmj.com/site/about/guidelines.xhtml

Theme	Subtheme	Participant quotation	Authors interpretation quotation
		maybe go and um always go to them. (Claire, 21)"[45]<br><br>"I'm grateful that they actually heard me, heard my voice and listened to me (Client#11)"[46]	
	1.3. Considering mother's readiness	"[I] lost the children—my three youngest girls plus my son. Because the Department saw that I—which, looking back now, I understand what they're saying— that I put my children in a dangerous situation … they could very well have gotten hurt. But I don't think I deserved to lose them—I needed help to get out of that situation and I asked for help to get out of that situation."[44]	"It was suggested that a comprehensive system of support could be achieved through coordination of education, safety, and support services with the stage of change of the caregiver (P 1 and 14). Whereas victims in the early stages needed information, victims in later stages needed shelter, support groups, or legal services (P 11 and 16)."[41]
	1.4. Patient materials	"Doctors should ask about exposure. We can invite the conversation through literature and posters. Some believe that we should not ask. It is not the doctor's role. Some people [victims] are not ready to do anything. For those people, you just say, "Here is some info. (P 14)"[41]	"We found that women with histories of IPV support positive educational interventions in the PED [Pediatric Emergency Department] and recommend interventions that educate about resources, signs of IPV, and the effects of childhood IPV exposure in a non-judgmental and positive manner."[43]
	1.5. Professional training	"…other agencies, they don't actually understand what our role is, you know…what would be really, really good is people should shadow…and they should do some training on what the role, the roles	"Participants voiced a need to deconstruct the misperception that CEDV [children's exposure to domestic violence] is harmless and to educate

For Peer review Only

Theme	Subtheme	Participant quotation	Authors interpretation quotation
		of different agencies are and what different agencies can actually offer. (Child protection social worker 4)"[35]	professionals, health care providers, judges, police officers, and school staff on the hazards of CEDV (P 19)."[41]
	1.6. Professional resources	"I think you need to have in your literature about mandated reporting because, as nurses, we are mandated reporters and we need to know, again, with that role it needs to be spelled out very clearly because in a court of law that's what would be thrown at us, if we have fulfilled our responsibilities in what way. (Nurse)"[42]	
	1.7. Professional supervision	"[...] I don't feel much impact, because, it's like, we get so many [children], recently there have been a lot, and so before we used to get more [impacted], but these days it is hard to go through one month in which you don't have one or two cases of poor treatment, sexual abuse [...] I have already had so many [cases] that we are beginning to become accustomed with the situation [...] (N15)."[47]	"Conversely, providers with strong relationships with their patients may be more susceptible to frustration, burnout, or hardening toward victims who are not ready to leave abusive relationships." (P 19).[41]
	1.8. Addressing systems' barriers	"I know people can be very protective about information sharing. 'Well *you're* working with the adult' and '*You're* working with the child', so there is	"Some of the reasons given for not exploring the possibility of DVA [domestic violence and abuse] when child safeguarding concerns arose included DVA not being 'first on your radar or list of things to

For peer review only

Theme	Subtheme	Participant quotation	Authors interpretation quotation
		not always that joined-up thinking about risks as a whole. (Specialist Social Worker: 1)"[40]	ask about' (GP31) and the problem of 'finding the time to do [it] all' (GP28)."[37]
2. Acceptable identification	2.1. Space and time	"It makes it a lot easier when they say that you can take your time. Like even for the first session…you don't have to do anything, you can just try and get used to being there and stuff. (Amelia, 15)"[45]<br><br>"Five years, same woman, all same—then I start to tell them everything then they said why you didn't tell me before? Then I tell them you know I was so scared that time like I feel safe with them to talk about everything then my children aid worker she find out shelter for me. [Client #9]"[46]<br><br>"The sense that it's okay to come and talk to you about anything that worries them. (GP21)"[37]	
	2.2. Vocabulary	"Ask the kids if they're OK…(Lisa, Claire's mother) Yeah, I mean that's exactly right (agreeing with Lisa in the focus group).<br>(Doctor) 'Are_you_OK?' (Patient) 'Oh no'. (Doctor) 'Why? Would you like to talk about it?' I think, and they're simple little words…(Penelope, mother)"[45]	

For peer review only

Theme	Subtheme	Participant quotation	Authors interpretation quotation
	2.3. Phased approach	"I brought up the fact that I wasn't sleeping like normal people and that it was affecting my school work because I really, really want to do well at school. So he kind of asked a few questions and stuff to find out what else is affecting me… (Amelia, 15)"[45]<br><br>"But there might be other things like sleeping issues, so then the doctor might go, 'OK, so why aren't you sleeping, do you think?' And then that might lead to the child opening up, 'Well this is happening and…Mum and Dad are fighting all the time and…(Lisa, mother)"[45]<br><br>"If they start talking about domestic violence then at that point engage them in the discussion about, "Well, do you realize that that is a form of child abuse? Not that I'm going to report you today or anything but I want you to be aware of that. (Community stakeholder)"[42]	
3. Acceptable initial response	3.1. Shifting focus	"We are child focused so this is what you need to do to keep your children safe, but obviously you need to be safe to do that, so it sort of interlinks, but	"Throughout the interviews, it became apparent that although the project focused on children, practitioners treated the mother–child dyad as a

For peer review only

Theme	Subtheme	Participant quotation	Authors interpretation quotation
		certainly the children are our first concern. I mean, you know, if the victim is unable to protect their child, then we need to look at whether there is more action that we need to do, something a bit sort of formal as opposed to giving advice. (Initial assessment manager 4)"[35]<br><br>"I'd have to tell her that, because of the children, I would basically be needing to involve child protection services. I would reassure and inform her as much as possible as to what that would involve, but yes, I'd have to make her aware that, you know, it wasn't actually up to her whether I, because I often get into arguments about 'oh no, but they'll take the children… (GP30)"[37]	single entity where the needs of the mother were consistent with the child's health and safety needs."[41]
	3.2. Emotional support	"But, within my capabilities, what I did was intensify surveillance, and try my best to help the child emotionally [...] (N4)."[47]<br><br>"she used to give me a lot of encouragement, tell me I was doing a good job in spite of you know all the circumstances?" "just thinking of her words does	

For peer review only

Theme	Subtheme	Participant quotation	Authors interpretation quotation
		help you know, when I'm being a bit hard on myself as a mom (Client #8)"[46]	
	3.3. Education	"Initial care of the mother–child consisted of gathering information, helping the mother to view the relationship honestly and objectively, educating the mother on potentially dangerous situations and ways to avoid or prevent abuse. Documenting provider efforts in the patient chart facilitated follow-up. (P 15)"[41]<br><br>"There's already a lot of anxiety if you know talk about Children Services. . . . Hopefully [CPS] could be seen as more of a positive support for this mom and the children. . . . One thing is just to educate and talk to moms about what role Children Services can play, and seeing it more as a positive not just a negative experience. . . .Educate that, you know, Children Services is here to help, Children's Services isn't here to remove your kids. (Community stakeholder)"[42]	
	3.4. Signposting	"She was really helpful, …, she was the one that gave us the number for the NSPCC. She was just good at	"Two examples were given of GPs overcoming the lack of time and giving young people information

Theme	Subtheme	Participant quotation	Authors interpretation quotation
		listening to us and that. (Nicola, Young People's Focus Group 1)" [35]	directly about relevant services they could access."[37]
4. Conflicting perspectives on acceptable identification and response	4.1. Engaging directly with children	"It depends on how they (doctors) approach them. And you've got to treat them as an individual, not as a (sibling) group." (Focus group participants all agree).(Charlie, mother)"[45]<br><br>". . . for me it's opening the opportunities for children to speak. Because key to it is understanding the children's view of it. The parents can say, 'Oh I love him, I'll never do it again'. . . . What's happening for the kid within all this? (Senior Social Worker: 2)"[40]<br><br>"When you complete initial assessments you do see the children alone and often they are quite young, and direct questions are very difficult. You are only in the house for an hour, an hour and a half doing your initial assessment. They usually know why this lady is coming to the house and they have been told not to say anything. You always ask if there is anything they would like to talk about, how are things at home, you know, what happens if they are naughty, do they go	"Lisa and Penelope suggest five as a suitable age to engage directly with children about their safety based on the level of awareness that their own children had at that age."[45]<br><br>"Whilst not precluding the doctor from addressing a child directly in a consultation where their mother is present, all the children and young people suggested an older age range [15-18] for being seen on their own."[45]<br><br>"Few clinicians would routinely seek to directly engage with the children concerned. Some might in some circumstance, after undertaking other actions such as talking to others (e.g. health visitors); or if an opportunity arose or excuse could be made. More than half would not seek to engage directly with children."[37]

For peer review only

Theme	Subtheme	Participant quotation	Authors interpretation quotation
		on the naughty stair, or, you know, but it's very difficult. (Initial assessment worker 3)"[35]	
	4.2. Management of safety	"AM: And in what way can doctors and nurses help families?<br>By checking if they're sick. Checking… if they're OK, feeling safe and all that.<br>AM: OK, what would be the best way that they could ask you if you were feeling OK?<br>Um…are you safe at home and at school and that…and at Dad's. (Fred, 9)"[45]<br><br>"We talk a lot about options if she is ready to leave [the abusive situation]. The options, if she is not ready to leave, a lot has to do with education, what are the dynamics of domestic violence, helping her to understand what is going on in her relationship, helping her to look at the cycle of violence, and to begin to recognize what is going on. A lot of what we do, if somebody is not ready to leave, has to do with safety planning and not safety planning about leaving, but safety planning while you are in the relationship. . . . If a violent incident happened, where can you tell the kids to go? Can the kids go	"Although formally asked about safety plans, many women either had not been involved in safety planning (or did not recall doing so) with their workers."[46]<br><br>"Similar to the victims who decided against leaving after approaching CPS [child protection service] for support, most victims who remained silent altogether believed they were acting in the best interest of their children at the time…. Some of the non-help-seeking decisions of victims are therefore well-informed decisions, often based on victims' individual risk assessment and their perception of how they can best ensure their children's safety at that particular point in time."[44]<br><br>"A common theme across the interviews with practitioners was the need for a shift towards more direct working with children and their parents. There was a widespread belief that current policy focused on monitoring risk, rather than addressing risk

For Peer review only

Theme	Subtheme	Participant quotation	Authors interpretation quotation
		upstairs? Can you teach them to dial 911? Can you teach them to leave and go to a neighbor's house? Who can the kids talk to? How do you talk to your kids about what is going on? And then, how do you get yourself safe? Not staying in the kitchen, not being in the bathroom, not staying in a place where you don't have an exit, not wearing scarves around your neck because they can be used to choke you. There are lots and lots . . . and for every person the safety plan is going to look a little bit different. (P 2D)"[41]	through systematic safety planning with adult victims and direct safety planning with children."[40]

For peer review only

Research checklist

ENTREQ checklist (Enhancing transparency in reporting the synthesis of qualitative research

No.	Item	Guide and description	Reported on Page #
1	Aim	State the research question the synthesis addresses	p.4, para 3
2	Synthesis methodology	Identify the synthesis methodology or theoretical framework which underpins the synthesis, and describe the rationale for choice of methodology (e.g. meta-ethnography, thematic synthesis, critical interpretive synthesis, grounded theory synthesis, realist synthesis, meta-aggregation, meta-study, framework synthesis)	p. 7, paragraph 3
3	Approach to searching	Indicate whether the search was pre-planned (comprehensive search strategies to seek all available studies) or iterative (to seek all available concepts until they theoretical saturation is achieved)	p. 5, paragraph 2
4	Inclusion criteria	Specify the inclusion/exclusion criteria (e.g. in terms of population, language, year limits, type of publication, study type)	p. 5-6, Table 1
5	Data sources	Describe the information sources used (e.g. electronic databases (MEDLINE, EMBASE, CINAHL, psycINFO), grey literature databases (digital thesis, policy reports), relevant organisational websites, experts, information specialists, generic web searches (Google Scholar) hand searching, reference lists) and when the searches conducted; provide the rationale for using the data sources	p. 5, paragraph 2
6	Electronic search strategy	Describe the literature search (e.g. provide electronic search strategies with population terms, clinical or health topic terms, experiential or social phenomena related terms, filters for qualitative research, and search limits)	p. 5, paragraph 2
7	Study screening	Describe the process of study screening and sifting (e.g. title, abstract and full text review, number of	p. 6, paragraph 2

For peer review only

No.	Item	Guide and description	Reported on Page #
	methods	independent reviewers who screened studies)	
8	Study characteristics	Present the characteristics of the included studies (e.g. year of publication, country, population, number of participants, data collection, methodology, analysis, research questions)	p. 8, paragraph 3<br>p. 8-10, Table 2<br>online Supplementary file 2
9	Study selection results	Identify the number of studies screened and provide reasons for study exclusion (e,g, for comprehensive searching, provide numbers of studies screened and reasons for exclusion indicated in a figure/flowchart; for iterative searching describe reasons for study exclusion and inclusion based on modifications to the research question and/or contribution to theory development)	p. 8, paragraph 2<br>Figure 1
10	Rationale for appraisal	Describe the rationale and approach used to appraise the included studies or selected findings (e.g. assessment of conduct (validity and robustness), assessment of reporting (transparency), assessment of content and utility of the findings)	p. 7, paragraph 1
11	Appraisal items	State the tools, frameworks and criteria used to appraise the studies or selected findings (e.g. Existing tools: CASP, QARI, COREQ, Mays and Pope [25]; reviewer developed tools; describe the domains assessed: research team, study design, data analysis and interpretations, reporting)	p. 7, paragraph 1
12	Appraisal process	Indicate whether the appraisal was conducted independently by more than one reviewer and if consensus was required	p. 7, paragraph 1
13	Appraisal results	Present results of the quality assessment and indicate which articles, if any, were weighted/excluded based on the assessment and give the rationale	p. 10, paragraph 2
14	Data extraction	Indicate which sections of the primary studies were	p. 7, paragraph 2

For peer review only

No.	Item	Guide and description	Reported on Page #
		analysed and how were the data extracted from the primary studies? (e.g. all text under the headings "results /conclusions" were extracted electronically and entered into a computer software)	
15	Software	State the computer software used, if any	NA
16	Number of reviewers	Identify who was involved in coding and analysis	p. 7, paragraph 2-3
17	Coding	Describe the process for coding of data (e.g. line by line coding to search for concepts)	p. 7, paragraph 3
18	Study comparison	Describe how were comparisons made within and across studies (e.g. subsequent studies were coded into pre-existing concepts, and new concepts were created when deemed necessary)	p. 7, paragraph 3
19	Derivation of themes	Explain whether the process of deriving the themes or constructs was inductive or deductive	p. 7, paragraph 3
20	Quotations	Provide quotations from the primary studies to illustrate themes/constructs, and identify whether the quotations were participant quotations of the author's interpretation	Online Supplementary file 6
21	Synthesis output	Present rich, compelling and useful results that go beyond a summary of the primary studies (e.g. new interpretation, models of evidence, conceptual models, analytical framework, development of a new theory or construct)	p. 11-14<br>p. 15-17, Table 3<br>Online supplementary files 4-6

For peer review only
